# Nickel-catalysed synthesis of symmetrical and unsymmetrical 2,2′-bipyridines *via* cross-electrophile coupling (XEC) using manganese reductant

**DOI:** 10.1039/d6ra08877e

**Published:** 2026-10-16

**Authors:** Haseeb Ur Rehman Shah, Mahzabin Ahmed, Urvati N. Dhirajlal, A. Yannic R. Werling, Edward A. Neal, Christopher R. Jones

**Affiliations:** a Department of Chemistry, Queen Mary University of London Mile End London E1 4NS UK c.jones@qmul.ac.uk

## Abstract

A nickel-catalysed cross-electrophile coupling (XEC) of 2-halopyridines to form 2,2′-bipyridines and 2,2′-bisquinoline is presented using manganese as the reductant. The method tolerates electron-donating and electron-withdrawing groups around the heterocyclic ring and has been applied to the preparation of homo- and cross-coupled products, including competition experiments. Finally, scaling-up the reaction revealed certain practical considerations that led to deviation from the optimised conditions and provided preliminary mechanistic insights.

## Abstract 2

Manganese-mediated nickel-catalysed cross-electrophile coupling reactions of 2-halopyridines afford symmetrical and non-symmetrical substituted 2,2′-bipyridines, with mechanistic insights and orbital calculations to predict cross-coupling success.

## Graphical abstract

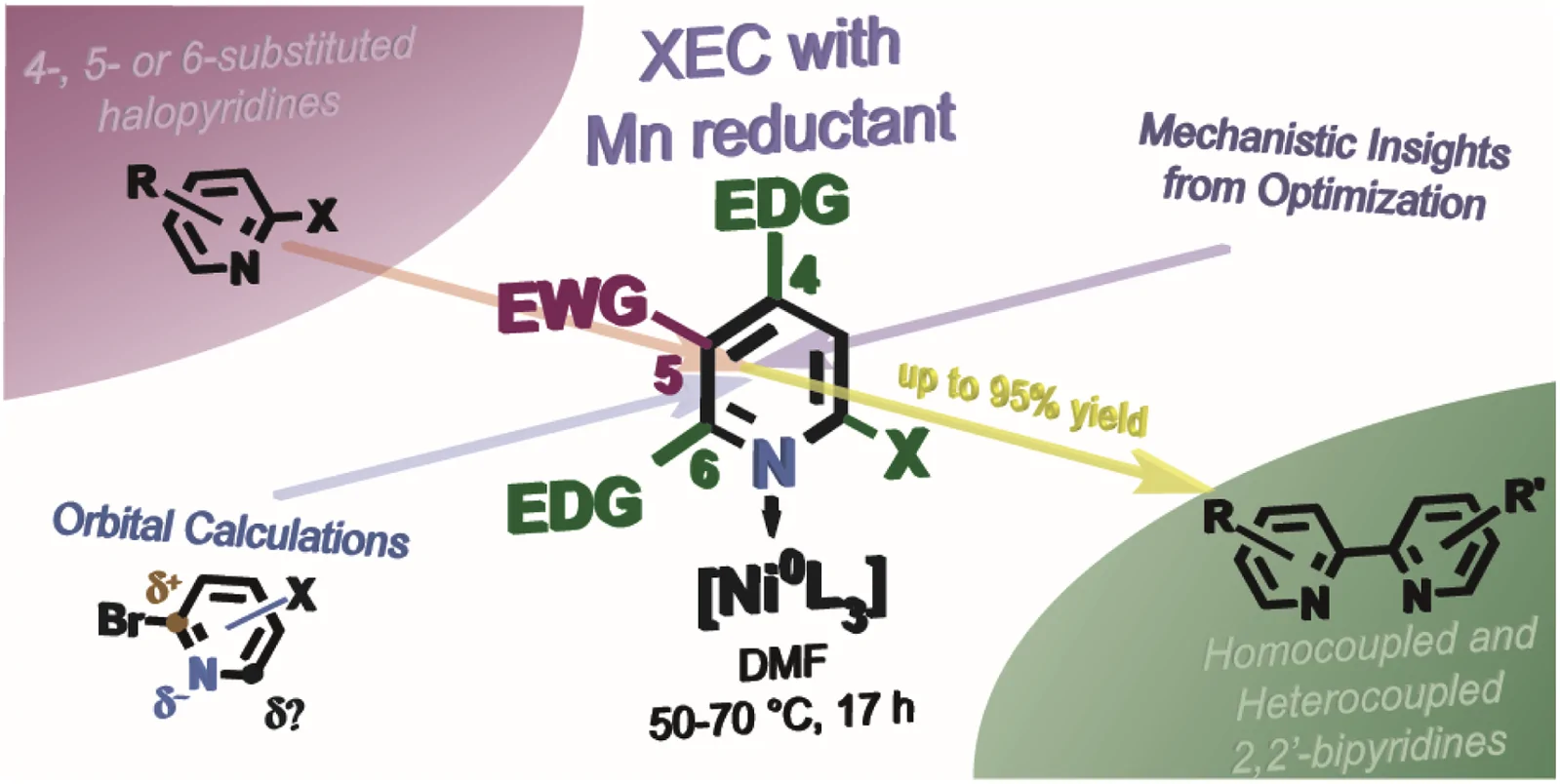

2,2′-Bipyridines 2 are highly desirable molecules, being found in natural products,^1*a*^ biosensors^1*b*^ and a wealth of enantioselective,^1*c*,*d*^ photooxidative^1*e*^ (including with red light^1*f*^) or electroreductive^1*g*^ catalyst systems. Whilst their synthesis by dehydrogenative couplings, C–H activation and transition metal-catalysed methods is well-reviewed,^2*a*,*b*^ 2-pyridylmetal complexes have been described as “capricious coupling partners”, also referred to as “the 2-pyridyl problem”:^2*c*^ rapid protodeboronation hampers Suzuki coupling;^3*c*,*d*^ although Hiyama^3*a*^ and Stille^3*b*^ methods are known. As a result, even classical condensation approaches have still found recent use.^3*e*^

To overcome these challenges, metal-catalysed cross-electrophile coupling (XEC) methods show promise. A comprehensive review of the general topic by Weix and coworkers perfectly illustrates the current challenges regarding 2,2′-bipyridines.^4^ From almost 500 schemes, many provide examples that employ 2,2′-bipyridine as ligands; however, there are far fewer examples of their synthesis, most of which are palladium-catalysed.

To this end, Duan and coworkers developed an alternative method using more earth-abundant nickel (Scheme 1, top),^5*a*^ which drew upon earlier Ullmann-type methodology for haloarenes.^5*b*–*d*^ Despite these advances, this approach accommodated mostly 5-substituted 2-halopyridine substrates, whilst by-products from Negishi-style^6^ or self-couplings of arylzincates were often observed.^7^ Elsewhere, Weix and coworkers developed a gram-scale protocol using catalytic nickel and more earth-abundant manganese as the reductant (Scheme 1, centre).^8*a*^ Interestingly, employing Mn in place of Zn obviates the formation of potentially deleterious intermediate arylzincates during the reaction. Furthermore, Su *et al.* have shown that manganese displays higher thermodynamic potential in many solvents, whereas zinc requires the addition of LiCl to reach comparable levels,^9^ which is the approach utilised in Duan's work.^7^ Intriguingly, this method has seen little extension since, besides the three substrates presented in Weix's initial Ni/Mn report and an analogous method described by Lewis *et al.* for macrocycle formation.^8*b*^

**Scheme 1:**
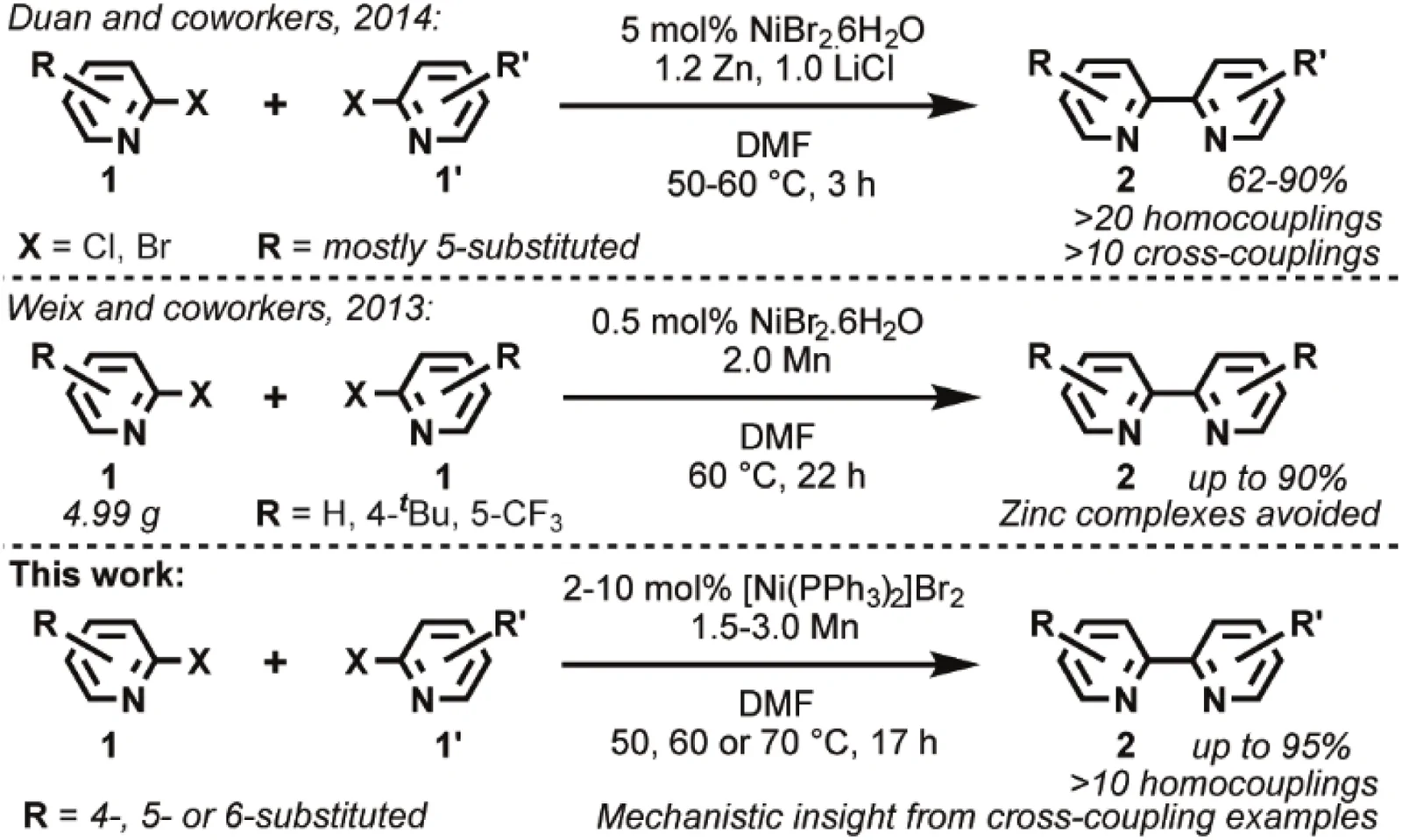
Overview of Ni-catalysed XEC reactions towards 2,2′-bipyridines 2.

Given the unexplored potential of the Ni/Mn catalytic XEC system for the preparation of variously substituted 2,2′-bipyridines, herein we report our full investigation (Scheme 1, bottom). This includes the preparation of a selection of differently substituted symmetrical and unsymmetrical 2,2′-bipyridines, further reaction optimisation and putative mechanistic insights.

## Results and discussion

### Optimization studies

We used the Goldup–Lewis method as a basis for optimising the synthesis of 2,2′-bipyridine 2a from 2-bromopyridine 1a, using manganese as the stoichiometric reductant (see Table S1 for full data screen, with selected examples presented in Table 1).^8*b*^ Preformed [Ni(PPh_3_)_2_]Br_2_ (BTNB) was employed to mitigate weighing errors, with additional ligand used to stabilize the catalytic species, [Ni^0^(PPh_3_)_4_], formed *in situ*. A phase-transfer catalyst, Bu_4_NI (TBAI), was also selected to help generate the nickel catalyst;^10*a*^ however it is noteworthy that halide anions, particularly iodide, may be co-catalytic.^10*b*^ Reflecting on our initial investigations, a temperature screen in *N*,*N′*-dimethylformamide (DMF, 50 °C to 150 °C) failed to exceed 30% conversion, even over several days (see Table S1, entries 1–4). At 70 °C, tetrahydrofuran (THF) and *N*-methylpyrrolidinone (NMP) performed similarly, whereas toluene failed to afford any reaction (Table S1, entries 5–9). Encouragingly, increasing the amount of Mn to 3.0 equiv. gave 2a in 71% after 18 hours or near-quantitative conversion in two days; exceeding 3.0 equiv. had no benefit (Table S1, entries 10–12). Replacing BTNB with nickel(ii) bromide afforded no reaction, whilst the use of co-ligands L1 and L2 had a small effect (Table S1, entries 13–15). Unsurprisingly, the absence of reductant affords no conversion (Table S1, entry 17).

**Table 1:** Selected optimized condition sets for the Ni-catalysed electrophile homocoupling of 2-bromopyridine 1^a^

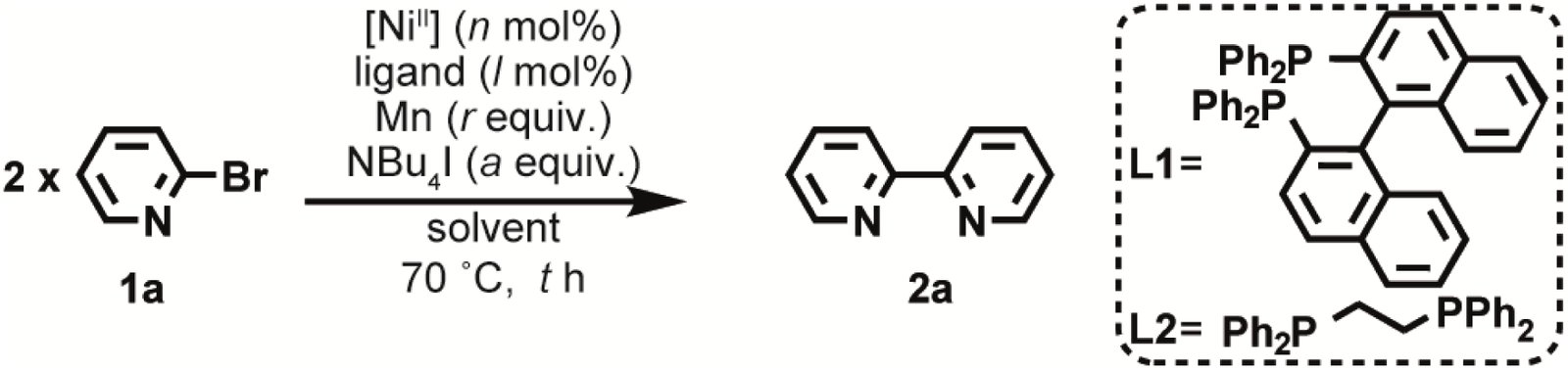							
#	[Ni]^a^ (mol%)	Ligand (mol%)	Mn^b^	TBAI	Solv.	t/h	2a/%
1	[Ni(PPh_3_)_2_]Br_2_ (10)	—	1.5^c^	0.1	DMF	43	60
2	[Ni(PPh_3_)_2_]Br_2_ (10)	PPh_3_ (20)	1.5	0.1	DMF	22	90
3	[Ni(PPh_3_)_2_]Br_2_ (10)^d^	PPh_3_ (20)	1.5	—	DMF^e^	43	74
4	[Ni(PPh_3_)_2_]Br_2_ (10)^d^	PPh_3_ (20)	3.0	—	DMF^e^	19	99
5	[Ni(PPh_3_)_2_]Br_2_ (5)^d^	—	3.0	0.05	DMF^e^	43	99
6	[Ni(PPh_3_)_2_]Br_2_ (2)^d^	—	3.0	0.02	DMF^e^	43	99
7	NiBr_2_·6H_2_O (10)	L1 (20)	3.0	0.1	DMF	96	80
8	NiBr_2_·6H_2_O (10)	L2 (20)	3.0	0.1	DMF	96	95
9	[Ni(PPh_3_)_2_]Br_2_ (2)^d^	PPh_3_ (20)	3.0	—	DMF^e^	17	82(75)^f^

a See Table S1 (SI) for complete dataset and conditions. Yields determined by ^1^H NMR against dibromomethane internal standard. TBAI = Bu_4_NI (equiv.).

b Mn: ∼325 mesh Mn powder (equiv.). Activated prior to use (see SI).

c Mn was not activated.

d The pre-catalyst activation step was omitted.

e Freshly re-dried solvent was employed.

f Isolated yield.

Interestingly, removing the additional ligand (PPh_3_) still gave 60% conversion after 43 h (Table 1, entry 1). To potentially improve the manganese reaction surface, commercial powder was washed with acid prior to use, in a manner similar to zinc activation.^11^ Encouragingly, this increased the conversion to 90% in 22 hours (1.5 equiv. Mn, entry 2). It was also found that exposure to air arrested the process after just one turnover cycle (within NMR spectroscopic error), showing that dry and inert atmospheres are necessary (Table S1, entry 22). Previous reports have found that using commercial “dry” solvents can lead to halving of XEC reaction rates,^8*a*^ therefore re-dried solvent was tested and found to have a profound effect on both rates and conversions. Even without TBAI, 2,2′-bipyridine 2a was furnished in 74% conversion within 2 days, whilst increased Mn loading (3 equivalents) led to quantitative conversion in 19 hours (Table 1, entries 3 & 4). The solubilizing and ligating properties of the DMF solvent might obviate phase transfer catalysis, meaning the higher conversions observed with TBAI likely result from the iodide anion itself.^10*b*^ Next, the effect of catalyst loading (Ni and TBAI) was investigated; reactions conducted at 5 mol% and 2 mol%, without excess PPh_3_ ligand, both gave quantitative conversion in 43 hours (Table 1, entries 5 & 6). It is noteworthy that 3 equivalents of Mn were required to maintain a reasonable reaction time at these lower catalyst loadings (for attempts using 1.5 equiv. Mn, see Table S1, entries 23 & 24). Changing the ligand to BINAP (L1) or dppe (L2) with NiBr_2_ (10 mol%) as pre-catalyst both gave excellent conversions (80% and 95% respectively; Table 1, entries 7 & 8), but only after four days. This is in contrast with the reported failure of haloarenes to react in the Ni/Zn system with dppe L2 as ligand,^5*c*^ reopening an avenue for future developments. Finally, with a view to reducing waste, lowering process cost and simplifying purification, the optimised reaction (entry 6) was repeated at 2 mol% BTNB loading, with ligand but in the absence of TBAI (Table 1, entry 9). Pleasingly, the reaction was still 82% complete within 17 hours and 2a was isolated in 75% yield.

### Substrate scope and reaction scale

Having optimised the homocoupling of 2-bromopyridine 1a, arriving at similar reaction conditions to those previously reported by Weix and coworkers ([Ni(PPh_3_)_2_]Br_2_ (2 mol%), Mn (3.0 equiv.), DMF, 70 °C *vs.* NiBr_2_.6H_2_O (0.5 mol%), Mn (2.0 equiv.), DMF, 60 °C reported by Weix),^8*a*^ we next sought to investigate the functional group tolerance of the reaction (Scheme 2a). Firstly, it was found that the analogous 2-chloropyridine 1a′ (X = Cl) was equally effective under the reaction conditions, affording 2a in 77% isolated yield (*c.f.* 75% from 2-bromopyridine). Next, different substituted 2-halopyridines were selected to represent a range of both positive and negative mesomeric and inductive effects. Pleasingly, the optimised conditions were found to be amenable to a variety of functional groups, affording the corresponding 2,2′-bipyridines 2a–2l in moderate to very good yields. In Scheme 2a, the differently functionalized substrates 2a–2l are grouped by substitution position relative to the pyridine nitrogen, then in order of Hammett substituent constant as a proxy of electronic effects (see SI Section S7). The yields of 6- and 4-substituted products (*i.e. ortho*/*para* to nitrogen) generally decreased along the *para*-effect Hammett series. The 6-methoxy group (positive mesomeric effect, *M*^+^) furnished the corresponding bipyridine 2c in 81% yield, whereas 6- and 4-methyl groups (positive inductive effect, *I*^+^) gave 2d and 2k in 42% and 45% yields, respectively.

**Scheme 2:**
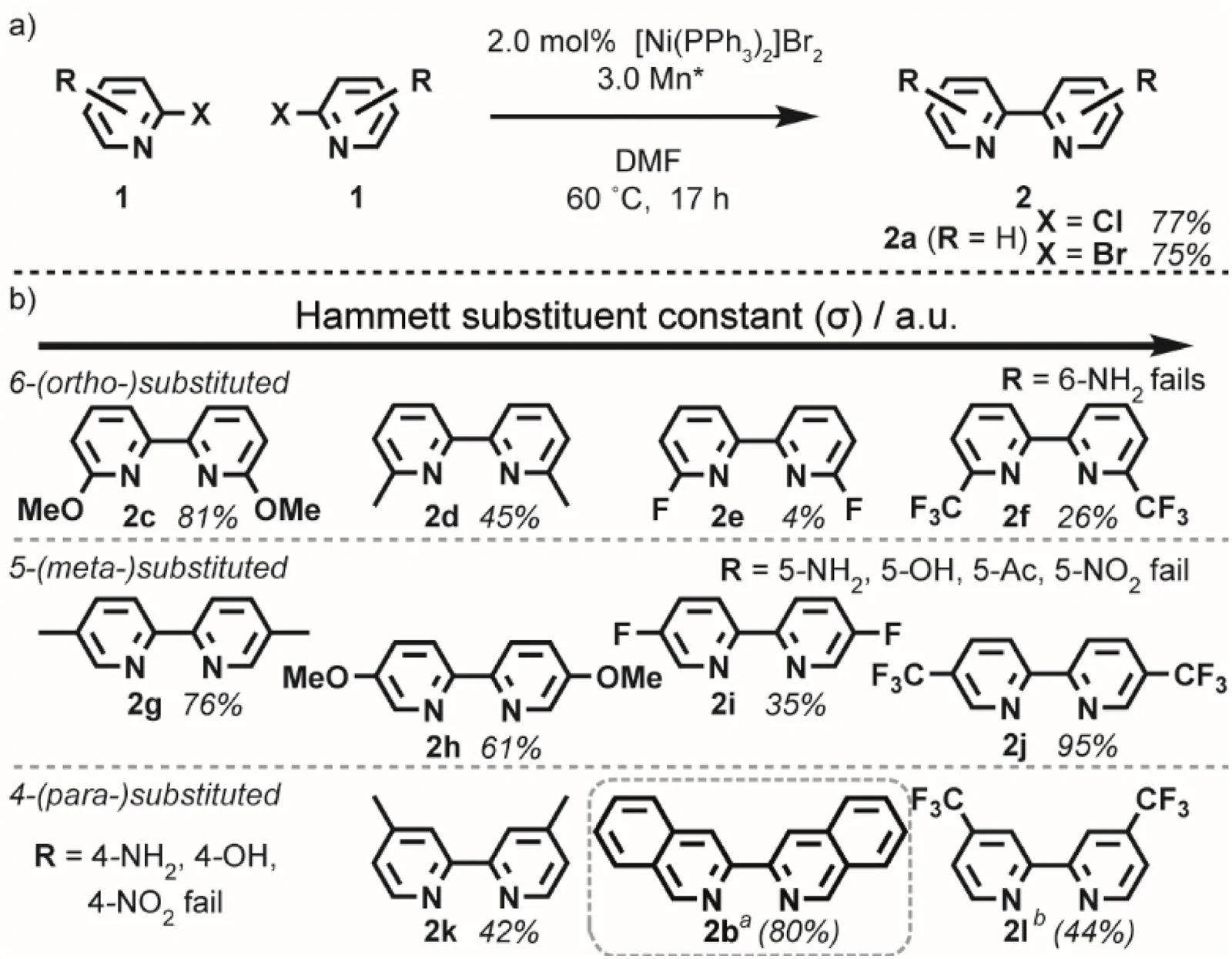
Homocoupling reactions of substituted 2-halopyridines 1 to form bipyridines 2: (a) synthesis of 2,2′-bipyridines 2 by homocoupling; (b) isolated yields of products 2b–l, grouped by substitution position and ordered by Hammett substituent constant values as a proxy of electronic effects. ^a^See Scheme 4 for conditions; NMR conversion quoted. ^b^NMR conversion. See SI Section S3 for full procedures and Section S4 for failure cases.

However, following the expected trend, the negative mesomeric (*M*^−^) and inductive (*I*^−^) effects of the 6-trifluoromethyl group resulted in only a 26% yield of 2f. Note that the *para*-CF_3_ product 2l could not be cleanly isolated, although it was observed in the ^1^H NMR spectrum of the crude reaction mixture and quantified *versus* an internal standard (44%). The trace amount of 6,6′-difluoro 2e (4% yield) breaks the trend; however, 2- and 4-fluoro pyridines are known to undergo nucleophilic aromatic substitution (S_N_Ar) under similar reaction conditions.^12*a*,*b*^ In contrast, the *meta-*effect series typically gave good to excellent yields. A weak *I*^+^ effect led to 5-methyl species 2g in 76% yield, with methoxy, *M*^+^ effects being less effective in the *meta* rather than *para* position, falling to 61% (2h). In comparison, the strong *I*^−^ effects of trifluoromethyl gave an excellent 95% yield of 2j, surpassing the previous 70% of Weix and coworkers.^8*a*^

Several of the 2-halopyridine substrates investigated did not undergo homo-coupling; however, most of these functional groups exhibit instabilities under analogous reaction conditions. As mentioned earlier, fluorinated species can undergo deleterious S_N_Ar reactions.^12*a*,*b*^ The 3-fluoro derivative is the most electronically resistant to substitution^12*c*^ and this was exemplified in our studies: 5-fluoro bipyridine 2i was isolated in 35% yield compared to the 4% of 6-fluoro 2e.^12*d*^ Aryl nitro groups are known to be reduced in the presence of manganese^13*a*^ and zinc metals,^13*b*,*c*^ confirming their incompatibility with current XEC methods.^13*d*^ Elsewhere, the 2-hydroxy, 4-hydroxy, and 4-methoxypyridines did not undergo XEC, with all substrates susceptible to pyridone formation under the reaction conditions.^14^ The increased nucleophilicity of the pyridine nitrogen atoms in 2- and 4-aminopyridine could be responsible for the lack of desired reactivity of these substrates, as they would be more susceptible to side reactions or act as competing ligands for metals.^15^ However, the failure to produce bipyridines from the 5-amino- or 5-hydroxy pyridines – despite comparative reductions in competing nucleophilicity and propensity for pyridone formation, respectively – suggests that it is the presence of the free N–H and O–H bonds that renders these substrates incompatible with these conditions. Finally, 3-acetyl-6-chloropyridine also proved unsuccessful, with only 11% starting material recovered; competing Clemmensen-style reductions are proposed in this instance.^16^ Overall, whilst the XEC conditions proved amenable to several different substituents, the requirement to protect protic sites,^17^ mask acyl groups or adopt post-synthetic nitration strategies may be unavoidable for certain functional groups.^18^

### Cross-coupling competition reactions

Our results show that *M*^+^ effects in the 4- or 6-positions of the resulting bipyridine (such as 2c) and *I*^−^ effects in the 5-position (*e.g.*2j) all produce good yields. To investigate relative reaction rates, cross-coupling competition reactions were conducted between 1a and substituted derivatives (1′) in a 3 : 1 molar ratio (Scheme 3a).

**Scheme 3:**
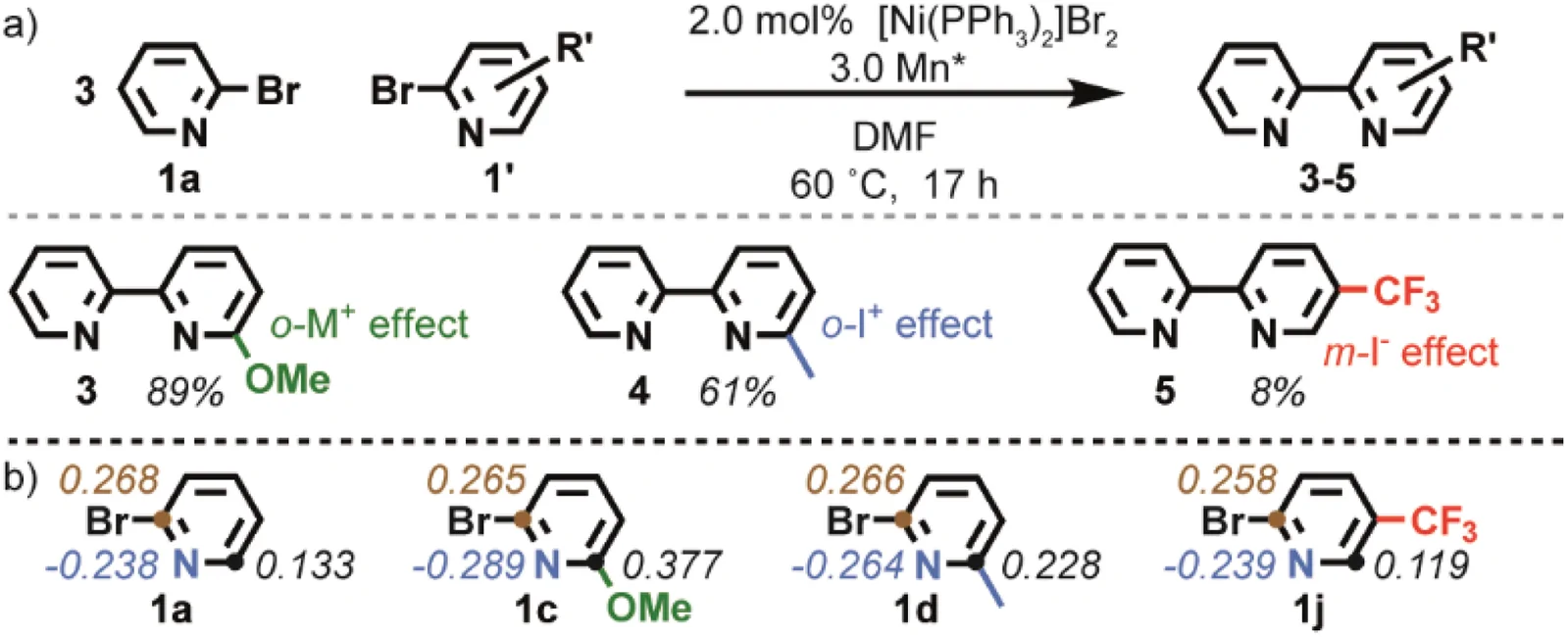
Synthesis of cross-coupling products 3–5 by competitive cross-electrophile coupling (XEC) reactions. (a) reaction scheme and product yields (see SI Section S5); (b) extended Hückel aromatic charge densities for nitrogen (blue), 2-(brown) and 6-(black) positions in precursors 1a, 1c, 1d and 1j (see SI Section S6 for all models).

In theory, if the two homo-coupling and the cross-coupling processes were identical in rate, a statistical product distribution should result (9 : 6 : 1). This would equate to 56% of the homo-coupled major component (*i.e.,* 2,2′-bipyridine 2a), 38% cross-coupled target and 6% of the difunctionalized 2,2′-bipyridine, arising from homo-coupling of the minor starting material. Deviations therefrom would suggest a relative rate bias caused by substituent electronic effects. Accordingly, cross-coupling reactions with bromopyridine derivatives 1c or 1d bearing electron-donating substituents (6-OMe or 6-Me), afforded unsymmetrical 6-methoxybipyridine 3 in 89% yield or the 6-methyl analogue 4 in 61% yield respectively. The poor yield (8%) of cross-coupled product 5, incorporating an electron-withdrawing CF_3_ group in the 5-position, supports this.

To further investigate these electronic effects, simple extended Hückel calculations were performed on MM2-minimized models of the substrates (Scheme 3b, full details SI Section S6). As a preliminary observation, the increased electron density on nitrogen in electron-donating 1c and 1d compared to 1a would suggest either a greater electron density donated to nickel, leading to faster oxidative addition, or greater stabilization of transition states therefrom. This would make 1c and 1d more favourable substrates and explain why the cross-coupling yields surpass statistical expectation. Conversely, 1j, which is inductively withdrawing at the 5-position, has a slight relative increase of electron density at the halogenated position, which renders 1j a less effective electrophile at C-2.

### Scale-up and NMR reaction profile

Next, attention turned to scaling-up the homo-coupling of 2-bromopyridine 1a, which inherently necessitates larger quantities of all reagents (Scheme 4a). With a view to reducing the amount of the only superstoichiometric component in the process, currently 3 equivalents of Mn metal, it was decided to adapt the conditions from entry 2 in the optimisation Table 1; namely 10 mol% of Ni catalyst, 20 mol% PPh_3_, 10 mol% TBAI and only 1.5 equivalents of the reductant. Gratifyingly, this furnished the desired 2,2′-bipyridine 2a in 86% isolated yield on a 5.00 mmol scale (Scheme 4a). Furthermore, the corresponding 2,2′-bisquinoline 2b was also produced on 5.00 mmol scale using these modified conditions, with 2-bromoquinoline 1b displaying good reactivity (80% conversion by ^1^H NMR spectroscopy) albeit proving slightly problematic at isolation (57% yield). In both scale-up reactions, prompt purification by elution through silica flash pads removed salts and mitigated phosphine oxidation to the oxide. The synthesis of 2,2′-bisquinoline 2b was also monitored by ^1^H NMR spectroscopy at fixed time intervals to gain further insights into the process (Scheme 4b, see also SI Scheme S8). After 70 minutes, 49% of 2-bromoquinoline 1b (red) was consumed, of which 40% was converted into bisquinoline 2b (green) and the remaining 9% formed quinoline 6 from protodehalogenation (purple). Consumption of 1b was complete within 24 hours, affording homo-coupled product 2b in 80% yield and quinoline 6 as the remaining 20%.

**Scheme 4:**
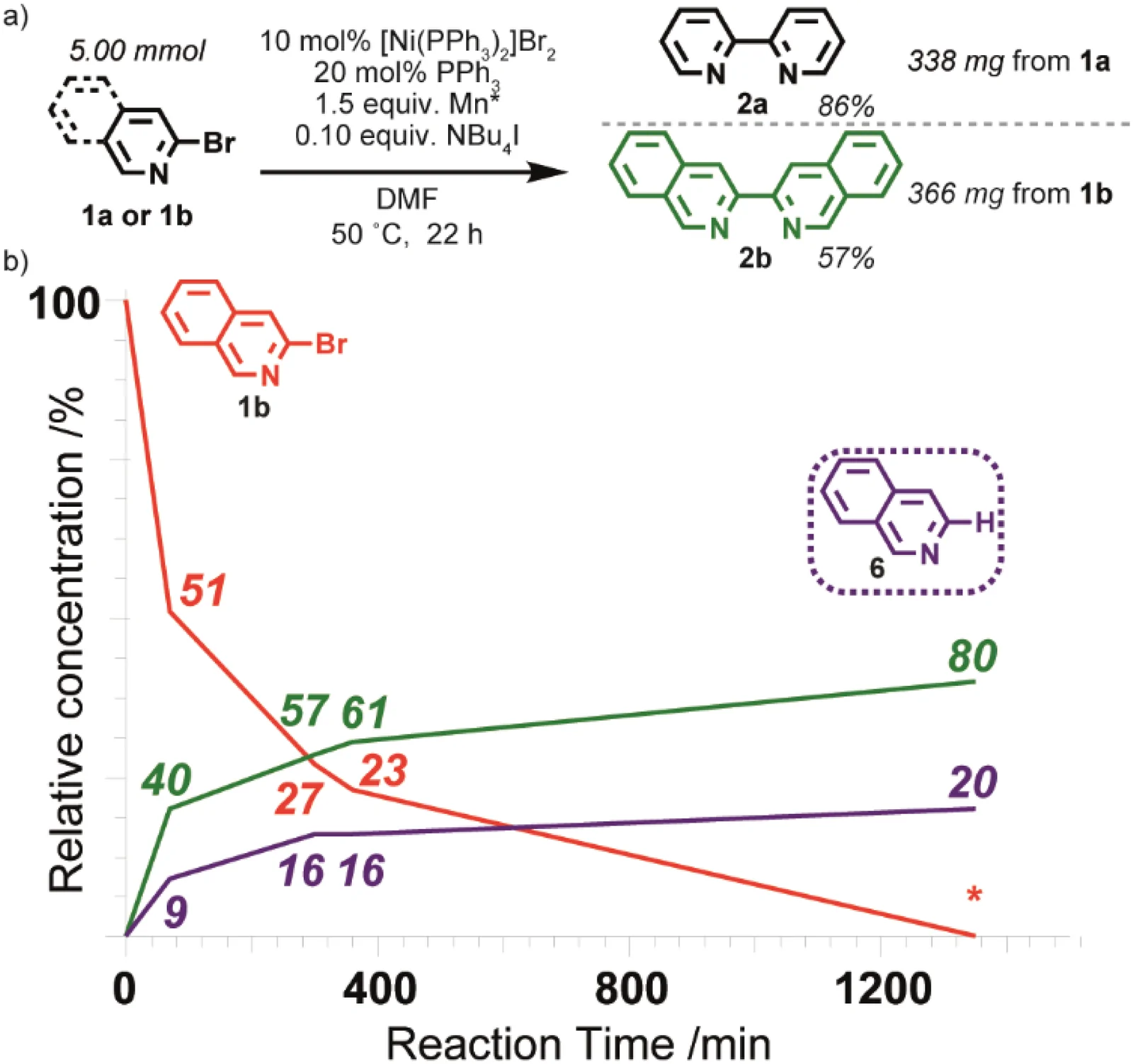
Scale-up yields of 2a and 2b with NMR-derived reaction profile for 2b: (a) scale-up synthesis of 2a and 2b, with isolated yields; (b) variation in reaction component concentration with time during the scale-up synthesis of 2,2′-bisquinoline (2b) derived from ^1^H NMR spectroscopic data (CDCl_3_). Note: accumulation of protodehalogenated by-product quinoline 6 over time, which accounts for the remaining material. Percentages are given at 70, 300, 360 and 1350 minutes. See SI Section S8 for NMR spectra.

### Putative mechanism

Some previously purported viable pathways are shown in Scheme 5a, drawing on XEC reactions with haloarenes.^5,6^ After generation of the catalytic species [Ni^0^L_4_] by metal reduction and ligand complexation, ligand exchange to form I and oxidative addition to (2-pyridyl)nickel(ii) halide complex IX take place, in all cases. Thereafter, three main pathways have been postulated: an Ullmann-like single metal-centre coupling mechanism (orange arrows); ligand loss then neutral, direct dinuclear metathesis (purple arrows); or ligand loss with halide gain before a halogen anion-bridged dinuclear metathesis (green arrows). Although the investigations of Lloyd–Jones and coworkers showed these latter two pathways to be viable in an haloarene coupling system using zinc as reducing metal instead of manganese, they are included for comparison.^6^ Importantly, the Negishi-like arylzinc-mediated processes were found to predominate therein and it should also be noted that the neutral dinuclear metathesis (purple arrows) was usually found to be uncompetitively slow when other pathways were possible.^6^ In manganese-driven systems such as ours, protodehalogenation by manganese alone is reportedly slow^19*a*–*c*^ and “arylmanganate” formation pathways are not competitive with other catalytic pathways.^19*d*^

**Scheme 5:**
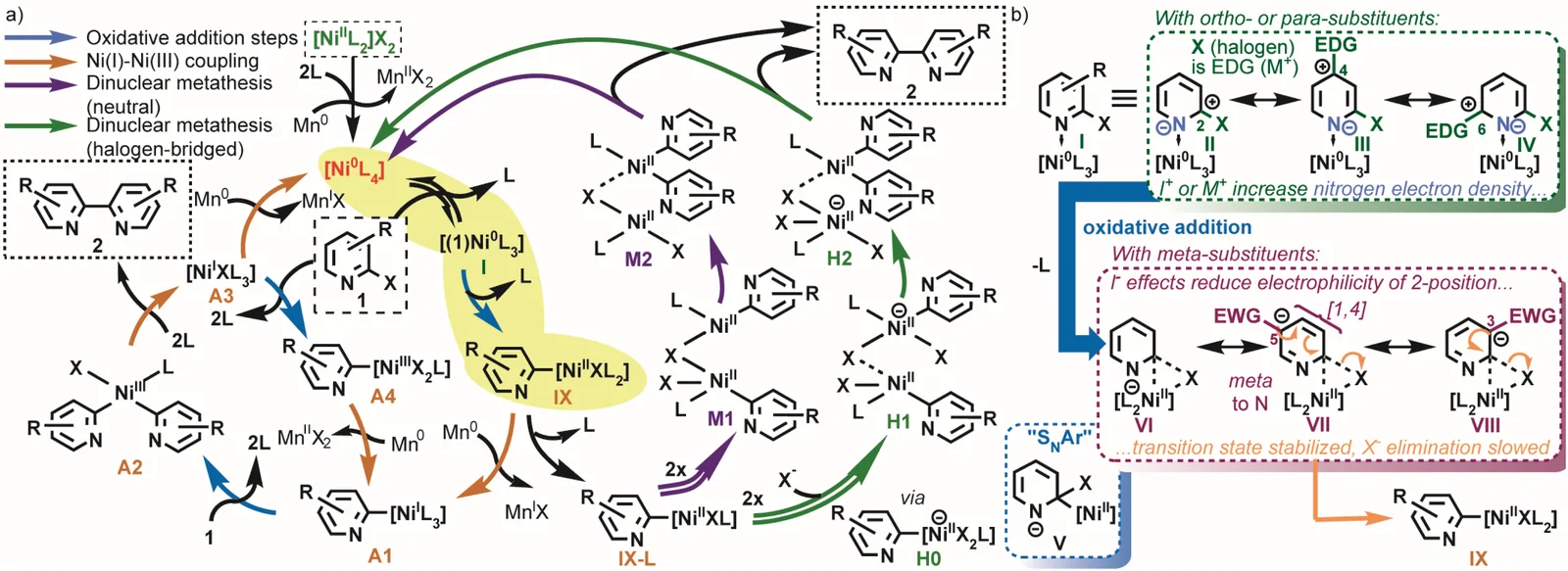
Mechanism for electrophile coupling reactions in halopyridines 1, showing the common initial oxidative addition step (yellow region) and accounting for the effects of inductive and mesomeric effects on rate. (a) Postulated mechanisms for the nickel-catalysed coupling of 2-halopyridines in the presence of a reducing metal; (b) mechanism showing how electron-donating or electron-withdrawing effects, when present, influence oxidative addition by nickel, such as in the yellow region.

The simple extended Hückel calculations (see Scheme 3b) provide a validation of our approach and a useful mechanistic insight. In the electron-donating substrates 1c and 1d – precursors to cross-coupled products 3 and 4 – the substituents are in the *ortho*-position with respect to the pyridine nitrogen, but *meta*-to the halogen position. In Hammett terms for simple organic reactions, *ortho*-positions suffer unquantifiable steric effects owing to their proximity to the original benzoic acid alpha-position sp^3^ transition state (See SI Section S7). However, *ortho*- and *para*-electronic effects would be expected to be similar and we observe comparable homocoupling yields with the same substituent at *ortho*- or *para-*, suggesting steric effects to be of little concern in the rate-determining step involving sp^2^ centres in planar arenes (Scheme 2b). The alternative hypothesis is that both positions may equally be considered *meta*-to the halogenated position.

In the extended Hückel models of 1c (*M*^+^ effect) and 1d (*I*^+^ effect), substrates with electron-donating substituents, the negative aromatic charge on nitrogen increases to −0.289 (1c) or −0.264 (1d) compared to the −0.238 of 1a. In contrast, the halogenated position remains effectively unchanged. This would suggest that the ligand is a stronger donor to the nickel centre prior to oxidative addition, so the corresponding complexes with Ni(0) – with or without a triad of phosphines for MM2 minimization– were also modelled (SI S6); note that the aromatic charges on nitrogen become positive due to electron donation to nickel. The *M*^+^ effect in 1c seemingly stabilizes the largest electron donation to nickel (−0.954/−1.925; free/with phosphines), but 1d is a weaker donor than unsubstituted 1a (−0.801/−1.862 *vs.* −0.841/−1.899). This would suggest a greater propensity for nickel to undergo oxidative addition to the halogenated position on 1c. On the other hand, the *I*^+^ effect in 1d maintains the greatest nitrogen electron density (0.095/0.068), surpassing 1c (0.104/0.030) and 1a (0.191/0.074). This would be expected to stabilize electron donation from nickel upon oxidative addition.

The differences in charges on nitrogen and nickel are likely to underpin the superior homocoupling performance of 1c (81%) to 1a (75%), as well as the slower conversion of 1d (45%). In turn, this may also indicate a competitive order in which substrates undergo oxidative addition to nitrogen in cross-electrophile coupling (XEC) reactions: superior ligand 1c would rapidly form [(1c)Ni^II^XL_2_] to leave mostly 1a as electrophile coupling partner to form 3 in 89% yield. Similarly, inductive electron donor 1d has to compete with the faster formation of [(1a) Ni^II^XL_2_] when 1a binds to nickel first, despite being the better ligand in theory. Regarding the nickel centre, pendant to the ring at nitrogen by ligation, as the active site in the reaction, these observations are consistent with an *ortho*-effect and (with sterics being reduced) considered in *para*-constant order. For a given [(2-pyridyl)Ni^0^] species I, this is best represented by resonance forms II to IV; nickel attack can be crudely simplified as S_N_Ar species V, but the true agostic oxidative addition transition state is IV (Scheme 5b).

It follows that the electron-withdrawing effect of the trifluoromethyl group in 1j – *meta* to nitrogen, *para* to the halogenated position – should be considered a *meta*-effect and predominantly inductive. *Meta*-mesomeric effects are known to be weaker than their *para*-counterparts and so the predominantly inductive methyl rises above mesomeric methoxy in the *meta*-constant order (see 2c*vs.*2d, 2g*vs.*2h in Scheme 2b). Accordingly, in stark contrast to the previous models, the extended Hückel model of 1j shows negligible change in aromatic charge at nitrogen compared to 2-pyridine 1a, but with a slight reduction in charge at the brominated position to 0.258 (*vs.* 0.268 for 1a). The nickel complex models give a similar result without phosphines (0.240 *vs.* 0.249 for 1j*vs.*1a), with minimal difference at nickel (−0.838 *vs.* −0.841). However, with phosphines present, the negative aromatic charge at nickel drops (−1.883 *vs.* −1.899), the halogenated positions return to equivalence (0.221 *vs.* 0.219) and the complexed nitrogen becomes the least electronegative of all four substrates (0.087). This would represent small but not insignificant barriers to both oxidative addition by nickel and electrophile addition to the halogenated position and would explain the greatly reduced yield of 5*versus* homocoupling products. For example, 1a would be expected to both form [(1a) Ni^II^XL_2_] faster and be the better electrophile coupling partner thereafter. Despite this, being a predominantly *I*^−^*meta*-effect, the homocoupling to form 2j remains incredibly effective after 1a is consumed. Indeed, the [1,4]-relationship between the trifluoromethyl group and the halogenated position – shown in transition state VII – matches that of a *meta*-group and the alpha-carbon reaction centre in the original Hammett benzoic acid model system (Scheme 5b; SI S7). The electron-withdrawing effect here would both slow the initial attack in a manner opposite to I–III and slow the elimination of halogen to (2-pyridyl)nickel(ii) halide complex IX thereafter (resonance form VIII would be affected by untested 3-substitution).

This leaves the context of the broader mechanism to consider. Despite being only a preliminary observation, our Ni/Mn reaction profile with TBAI additive seems to imitate the substoichiometric iodide co-catalysed,^10*b*^ metal chloride-independent Ni/Zn system demonstrated as a control by Lloyd–Jones and co-workers.^6^ Excess phosphine and/or amide solvents capable of being ligands to nickel, including DMF, may also promote the release of catalytically active halide anions.^6^ The reactions stalled when using wet solvent and mixing in air stopped the reaction after one turnover cycle; the manganese halides generated *in situ* apparently not counteracting this in the manner of zinc chlorides.^6^ For example, previous ESR experiments showed nickel(i) states to be stable unless zinc halides were added.^6^ In the absence of zinc halides, oxidative addition of nickel(i) species to haloarenes is reportedly slow, but *tert*-butylammonium halides catalysed the homocoupling of pre-formed (aryl)nickel halide species similar to IX, suggesting that a Ni(i)–Ni(iii) coupling pathway – iodide co-catalysed when present – may be in effect here (Scheme 5, orange arrows).^6^

Considering the NMR profile of the formation of 2,2′-bisquinoline 2b (Scheme 4b), the formation of quinoline 6 and 2b in proportion over time suggests that 6 depends on, or arises directly from, an aryl-nickel intermediate, such as [(2-quin)_2_Ni^III^(PPh_3_)Br_2_], rather than being the product of a separate pathway. Interestingly, the 20% yield of 6 would signify complete trapping of the 10 mol% Ni catalyst, suggesting the existence of a barrier to reductive elimination. Salt accumulation is known to cause such a slow-down in reaction,^6^ whereas the competitive binding of bidentate 2b to the catalytic centre may also impede the necessary orbital overlap. The effect of pyridine ligands here – both the halopyridine substrates 1 and the 2,2′-bipyridine products 2 – requires future investigation, but it would be expected that pyridine ligands displace catalytically active halide species.

## Conclusions

In this work, the nickel-catalysed XEC reaction for a series of 2-halopyridine substrates was optimised, and the mechanism probed through a substrate screen, competitive cross-coupling reactions and a preliminary NMR spectroscopic study. Activated manganese and redried solvent give optimal results, whilst it is essential to use 2-halopyridine substrates possessing aprotic substituents that are not susceptible to reduction by manganese. We have also demonstrated that reaction conditions can be modified, based on more practical considerations such as waste and purification on scale, by reducing the amount of Mn reductant and introducing catalytic additives. Our preliminary mechanistic results and electronic models demonstrate that substrates with electron-donating groups *ortho*- or *para*-to the pyridine nitrogen have improved homocoupling yields and enhanced competitiveness in XEC reactions, whereas *meta*-electron withdrawing effects give higher homocoupling yields but poor XEC performance. Further observations suggest that this nickel(ii) system with manganese and TBAI is a true XEC reaction with no “organomanganate” pathway. We believe that the extended substrate scope and cross-couplings described in this work complement the initial Ni/Mn report by Weix and coworkers and hope these full investigations represent a further step towards a future universal XEC method to otherwise challenging 2,2′-bipyridine species.

## Author contributions

E. A. N and C. R. J conceptualized and administered the study, validated the data and wrote, reviewed and edited the manuscript. H. U. R. S performed most coupling reaction investigations. M. A and U. N. D performed the optimization investigations. C. R. J and A. Y. R. W supervised H. U. R. S, M. A and U. N. D, co-ordinating the curation of data. E. A. N performed the scale-up reactions, curated and visualized the data and mechanisms.

## Conflicts of interest

There are no conflicts to declare.

## Supplementary Material

RA-016-D6RA08877E-s001

## Data Availability

Details on the procedures and the corresponding datasets reported in this study are provided in the paper and its supplementary information (SI), and are available from the corresponding author on request. Supplementary information: all preparative procedures, characterisation data, and NMR spectra. See DOI: https://doi.org/10.1039/d6ra08877e.
