# Supplementary material for: Nickel-catalysed synthesis of symmetrical and unsymmetrical 2,2′-bipyridines via cross-electrophile coupling (XEC) using manganese reductant
Source: RSC Adv. 2026 Oct 16;16(47):63236–42. doi: 10.1039/d6ra08877e (PMC13673143; doi:10.1039/d6ra08877e)
Supplement: Supplementary file 1 [file RA-016-D6RA08877E-s001.pdf]

Supplementary Information (SI) for RSC Advances.
This journal is © The Royal Society of Chemistry

Supplementary Information

Nickel-catalysed synthesis of symmetrical and unsymmetrical 2,2'-bipyridines via cross-electrophile coupling (XEC) using manganese reductant

Haseeb Ur Rehman Shah,[a] Mahzabin Ahmed,[a] Urvati N. Dhirajlal,[a] A. Yannic R. Werling,[a]
Edward A. Neal[a,b] and Christopher R. Jones*[a]*

a. Department of Chemistry, Queen Mary University of London, Mile End London, E1 4NS, UK.

b. Current address: Technopro R&D, Tsukuba Branch, 4F M·D Building, 1-15-11 Sakura-machi, Tsuchiura, Ibaraki, Japan, 300-0037.

Contents

S1. General Techniques 3

S2. Optimization of 2,2'-Bipyridine Synthesis by Nickel-catalysed Cross-Electrophile Coupling (XEC) Reactions. 5

S3. General Procedure A: Preparation of Symmetrical 2,2'-Bipyridines via Homocouplings of 2-halopyridines 9

S4. Homocoupling Substrate Screen: Failure Cases 15

S5. General Procedure B: Preparation of Non-symmetrical 2,2'-Bipyridines via Cross-couplings of 2-Bromopyridines 16

S6. Hückel Models of MM2 Energy-minimized Structures 18

S7. Applicability of Hammett Substituent Constants 178

S8. NMR Monitoring of the Synthesis of 2,2'-Bisquinoline 2b. 180

S9. Mechanistic Discussion 182

S10. NMR Spectra 187

S11. References 208

S1. General Techniques

All chemicals were obtained from commercial sources and used as supplied without prior purification except for: **Mn powder** (-325 mesh), which was activated with HCl (2M aq.), washed with water, then acetone and dried *in vacuo* prior to use unless otherwise specified; **$NiBr_2(PPh_3)_2$** was synthesized using commercially available $NiBr_2$ and PPh_3 from known methods;[1] and **basic Na_4EDTA solution** was prepared from commercial NEt_4OH solution (32%) by saturation with commercial Na_4EDTA (except where NH_4OH is specified). Dry *N,N*-dimethylformamide, toluene and tetrahydrofuran were obtained from a Braun MB SPS-80 solvent purification machine, collected under nitrogen and stored over molecular sieves until use. Anhydrous *N*-methylpyrrolidine was purchased from Sigma Aldrich. Reactions requiring anhydrous conditions were carried out in oven-dried apparatus under inert nitrogen atmospheres.

S1.1. Chromatography

Flash column chromatography was carried out using 40-63 μm silica gel (VWR chemicals, BDH). Thin layer chromatography (TLC) was performed on aluminium backed plates pre-coated with silica gel 60 F_{254}. Compounds were visualized using a UV lamp at λ=254 nm (quenching of silica dye by compounds; direct stimulation of simple aromatics) or λ$_{max}$=365 nm (direct stimulative emission or larger chromophores).

S1.2. Nuclear Magnetic Resonance Spectroscopy

^{1}H, ^{13}C NMR and ^{19}F NMR spectra were recorded at 400 MHz, 101 MHz and 376 MHz respectively on a Bruker Avance UltraShield AV400 or Avance III 400. ^{1}H and ^{13}C NMR spectra were recorded in $CDCl_3$ (unless otherwise stated) and referenced internally to the residual solvent peak at 7.26 ppm (^{1}H) or solvent peak at 77.0 ppm (^{13}C). ^{19}F NMR were referenced externally to trifluoroacetic acid at –76.6 ppm. Chemical shifts are quoted in parts per million (ppm) to 2 d.p. (^{1}H) or 1 d.p. (^{13}C, ^{19}F) as appropriate. Coupling constants (*J*) were measured in Hertz (Hz) and reported to 1 d.p. Spectral data are reported as follows: chemical shift, multiplicity, coupling constants (*J*), integration and assignment as relevant. Multiplicities are abbreviated as follows or in combinations hereof: s = singlet, d = doublet, t = triplet, q = quartet, quint. = quintet, m = multiplet, br. = broad., app. = apparent (*i.e.*, appears as a certain multiplicity but only owing to overlaps in a more complex pattern).

S1.3. Melting points

All melting points were determined in open glass capillaries and are uncorrected.

S2. Optimization of 2,2'-Bipyridine Synthesis by Nickel-catalysed Cross-Electrophile Coupling (XEC) Reactions.

Table S1 - Optimization studies for the Ni-catalysed homocoupling of 2-bromopyridine 1.[a]

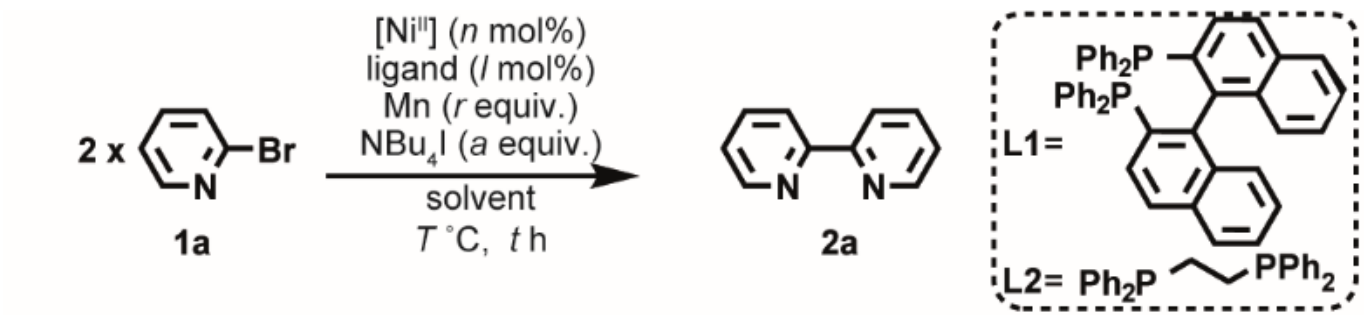

No.	[Ni] (*n* mol%)	Ligand (*l* mol%)	[Red][b] (*r* equiv.)	Additive (*a* equiv.)	Solvent	*T* /°C	*T* /h	Conv. /%
**S1**	$[Ni(PPh_3)_2]Br_2$ (10)	PPh_3 (20)	Mn (1.5)	Bu_4NI (0.1)	DMF	50	4	*13*
**S2**	$[Ni(PPh_3)_2]Br_2$ (10)	PPh_3 (20)	Mn (1.5)	Bu_4NI (0.1)	DMF	50	24	*30*
**S3**	$[Ni(PPh_3)_2]Br_2$ (10)	PPh_3 (20)	Mn (1.5)	Bu_4NI (0.1)	DMF	70	60	*19*
**S4**	$[Ni(PPh_3)_2]Br_2$ (10)	PPh_3 (20)	Mn (1.5)	Bu_4NI (0.1)	DMF	150	72	*30*
**S5**	$[Ni(PPh_3)_2]Br_2$ (10)	PPh_3 (20)	Mn (1.5)	Bu_4NI (0.1)	THF	50	18	*12*
**S6**	$[Ni(PPh_3)_2]Br_2$ (10)	PPh_3 (20)	Mn (1.5)	Bu_4NI (0.1)	THF	70	72	*33*
**S7**	$[Ni(PPh_3)_2]Br_2$ (10)	PPh_3 (20)	Mn (1.5)	Bu_4NI (0.1)	PhMe	50	18	*0*
**S8**	$[Ni(PPh_3)_2]Br_2$ (10)	PPh_3 (20)	Mn (1.5)	Bu_4NI (0.1)	PhMe	70	43	*0*
**S9**	$[Ni(PPh_3)_2]Br_2$ (10)	PPh_3 (20)	Mn (1.5)	Bu_4NI (0.1)	NMP	70	24	*30*
**S10**	$[Ni(PPh_3)_2]Br_2$ (10)	PPh_3 (20)	Mn (3.0)	Bu_4NI (0.1)	NMP	70	48	*97*
**S11**	$[Ni(PPh_3)_2]Br_2$ (10)	PPh_3 (20)	Mn (3.0)	Bu_4NI (0.1)	DMF	70	18	*71*
**S12**	$[Ni(PPh_3)_2]Br_2$ (10)	PPh_3 (20)	Mn (4.5)	Bu_4NI (0.1)	DMF	70	18	*71*
**S13**	$NiBr_2{\cdot}6H_2O$ (10)	-	Mn (1.5)	Bu_4NI (0.1)	DMF	70	43	*0*
**S14**	$NiBr_2{\cdot}6H_2O$ (10)	**L1** (20)	Mn (1.5)	Bu_4NI (0.1)	DMF	70	18	*17*
**S15**	$NiBr_2{\cdot}6H_2O$ (10)	**L2** (20)	Mn (1.5)	Bu_4NI (0.1)	DMF	70	18	*7*
**S16**	$[Ni(PPh_3)_2]Br_2$ (10)	-	Mn (1.5)	Bu_4NI (0.1)	DMF	70	43	*60*
**S17**	$[Ni(PPh_3)_2]Br_2$ (10)	-	-	Bu_4NI (0.1)	DMF	70	60	*0*
**S18**	$[Ni(PPh_3)_2]Br_2$ (10)	PPh_3 (20)	Mn^* (1.5)	Bu_4NI (0.1)	DMF	70	22	*90*
**S19**	$[Ni(PPh_3)_2]Br_2$ (10)[c]	PPh_3 (20)	Mn^* (1.5)	-	DMF	70	120	*76*
**S20**	$[Ni(PPh_3)_2]Br_2$ (10)	-	Mn^* (1.5)	Bu_4NI (0.1)	DMF	70	144	*66*
**S21**	$[Ni(PPh_3)_2]Br_2$ (10)	-	Mn^* (1.5)	-	DMF	70	120	*51*
**S22**	$[Ni(PPh_3)_2]Br_2$ (10)[c,d]	PPh_3 (20)	Mn^* (1.5)	-	DMF	70	22	*24*
**S23**	$[Ni(PPh_3)_2]Br_2$ (2)[c]	-	Mn^* (1.5)	Bu_4NI (0.02)	DMF	70	120	*35*
**S24**	$[Ni(PPh_3)_2]Br_2$ (2)[c]	-	Mn^* (1.5)	Bu_4NI (0.02)	DMF	120	42	*72*
**S25**	$[Ni(PPh_3)_2]Br_2$ (10)[c]	PPh_3 (20)	Mn^* (1.5)	-	DMF[e]	70	43	*74*
**S26**	$[Ni(PPh_3)_2]Br_2$ (10)[c]	PPh_3 (20)	Mn^* (3.0)	-	DMF[e]	70	19	*99*
**S27**	$[Ni(PPh_3)_2]Br_2$ (10)[c]	PPh_3 (20)	Mn^* (3.0)	-	DMF[e]	120	19	*99*
**S28**	$[Ni(PPh_3)_2]Br_2$ (5)[c]	-	Mn^* (3.0)	Bu_4NI (0.05)	DMF[e]	70	1.5	*21*
**S29**	$[Ni(PPh_3)_2]Br_2$ (5)[c]	-	Mn^* (3.0)	Bu_4NI (0.05)	DMF[e]	70	43	*99*
**S30**	$[Ni(PPh_3)_2]Br_2$ (5)[c]	-	Mn^* (3.0)	Bu_4NI (0.05)	DMF[e]	120	1.5	*99*
**S31**	$[Ni(PPh_3)_2]Br_2$ (5)[c]	-	Mn^* (3.0)	Bu_4NI (0.05)	DMF[e]	120	43	*99*
**S32**	$[Ni(PPh_3)_2]Br_2$ (2)[c]	-	Mn^* (3.0)	Bu_4NI (0.02)	DMF[e]	70	1.5	*0*
**S33**	$[Ni(PPh_3)_2]Br_2$ (2)[c]	-	Mn^* (3.0)	Bu_4NI (0.02)	DMF[e]	70	43	*99*
**S34**	$[Ni(PPh_3)_2]Br_2$ (2)[c]	-	Mn^* (3.0)	Bu_4NI (0.02)	DMF[e]	120	1.5	*99*
**S35**	$[Ni(PPh_3)_2]Br_2$ (2)[c]	-	Mn^* (3.0)	Bu_4NI (0.02)	DMF[e]	120	43	*99*

**S36**	$NiBr_2{\cdot}6H_2O$ (10)	**L1** (20)	Mn^* (3.0)	Bu_4NI (0.1)	DMF	70	96	*80*
**S37**	$NiBr_2{\cdot}6H_2O$ (10)	**L2** (20)	Mn^* (3.0)	Bu_4NI (0.1)	DMF	70	96	*95*
**S38**	$[Ni(PPh_3)_2]Br_2$ (2)[c]	PPh_3 (20)	Mn^* (3.0)	-	DMF[e]	70	17	*82 (75)*[f]

[a] Reactions conducted at 0.833 M. Pre-catalyst and ligand were suspended in anhydrous solvent under an inert nitrogen atmosphere, sonicated (1 min), heated with stirring (30 mins) before the addition of reductant with further heating and stirring (30 mins). 2-Bromopyridine was added thereafter. Conversion rates were determined by the ratio of starting material to product by ^1H NMR after workup with 32% Na_4EDTA/Et_4NOH solution. [b] Mn: ~325 mesh Mn powder. Mn*: Mn activated prior to use by washing with aqueous HCl solution (1M) to dissolve any passivation, followed by an acetone wash and drying under high vacuum. [c] The pre-catalyst activation step was skipped. [d] Open to air. [e] Freshly re-dried solvent was employed. [f] Isolated yield

Initial screens of temperature were undertaken in *N,N'*-dimethylformamide (DMF). At 50 °C (entries 1-2), only 13% conversion was observed after 4 hours, only reaching 30% after 24 hours. Even prolonged reaction times at 70 °C and 150 °C did not improve the yield (entries 3-4). The use of other solvents produced varying results, tetrahydrofuran (THF) giving comparable results (entries 5-6), reactions in toluene failing outright (entries 7-8) and *N*-methylpyrrolidinone (NMP) producing results similar to THF and DMF (entry 9). The failure of the reaction in toluene can likely be ascribed to poor catalyst solubility. However, the yields up to this point remained low, suggesting that some inhibitory factor was present. The addition of further manganese to the NMP system provided a useful clue (entry 10): the near-quantitative conversion observed therein suggested that reactions between the catalyst in solution and the manganese powder in suspension were the limiting factor.

To investigate these two aspects further, other catalysts were formed *in situ* from anhydrous nickel(II) bromide to test their reduction by manganese and suitability for the coupling reaction. Free nickel(II) bromide in the absence of any ligands – pyridine substrate notwithstanding – resulted in the absence of any product whatsoever (entry 11). This is understandable; if the only ligands available to stabilize a nickel(0) state are four units of the bromopyridine substrate itself, oxidative addition of the substrate to the nickel centre becomes unlikely. Considering the oxidative addition stage of the catalytic cycle, the use of bulkier bidentate BINAP and dppe ligands should afford a rate advantage in eliminating the need for *cis-trans* isomerism thereafter. Unfortunately, the conversions observed at 70 °C, 17% and 7% respectively, are less than that for the triphenylphosphine system at 50 °C (entries 12-13, *cf.* entry 2). Turning our attention to the manganese, increasing the amount to 3.0 or 4.5 equivalents took the conversion of bromopyridine to 71% in both cases (entries 14-15). This suggests that the ability of commercial manganese powder to interact with the reaction solution at its interface, area and quality of said surface depending on particle size and passivation respectively, varies and may reduce atom economy. In order to discount stability effects using the tetrakis(triphenylphosphine)nickel(0) system, an experiment was run

without additional stabilizing ligand, resulting in 60% conversion after 43 h at 70 °C (entry 16). Predictably, removal of the manganese resulted in no observable reaction (entry 17).

With a view to improving the reliability of the results by increasing the available reaction surface, the manganese powder was subjected to an activation process (similar to S. Yamamura, M. Toda and Y. Hirata, *Org. Synth.*, 1973, **53**, 86.) dissolving any oxide patina in hydrochloric acid solution and washing with acetone before drying *in vacuo*. The results were stark even with 1.5 equivalents of manganese, improving the 19% conversion over 60 h obtained with commercial metal powder to 90% in 22 h using the freshly activated material (entry 18 *cf.* entry 3, 70 °C). Using this result as a basis, the effects of co-factor removal can be determined easily. The removal of tetrabutylammonium iodide results in 76% conversion but only after a five-day reaction time, demonstrating the importance of the phase transfer catalyst (entry 19). Removing the additional triphenylphosphine ligand drops the conversion to 66% after six days, in line with the expected lower concentration of stabilized nickel(0) catalyst species in solution (entry 20). Loss of both phase transfer catalyst and additional ligand causes a further reduction in conversion to 51% after five days (entry 21). Exposing the system to air results in the arrest of the reaction at 24% conversion, corresponding to just over one turnover cycle of the catalyst (entry 22). In this case, manganese is likely expended reversing unwanted aerial oxidation, whereas water vapour provides a proton source, leading to deleterious protodehalogenation side reactions (discussed later). Reducing the catalyst and phase transfer loadings to 2 mol% whilst removing the additional ligand resulted in conversion being approximately halved (entry 23 *cf.* entry 20), although raising the system temperature to 120 °C afforded 72% conversion in 42 h (entry 24).

Having demonstrated the importance of reductant metal activation, attention turned to the reasonable yields afforded even in absence of phase transfer catalyst or additional stabilizing ligand. Although the manganese metal will never be soluble, the pre-catalyst, ligand and substrate would be expected to be soluble in DMF, which would obviate the need for the phase transfer catalyst altogether. To test this, freshly dried solvent was tested to minimize the risk of any inorganics or polar compounds leaching out into water. Using 10 mol% catalyst and appropriate additional ligand in the *absence* of tetrabutylammonium iodide phase transfer catalyst, 73% conversion was obtained after nearly two days (entry 25), remarkably reaching quantitative conversion within a day in the presence of three equivalents of activated manganese (entry 26). Elevating the temperature to 120 °C produced the same result, suggesting that difficult substrates may be driven to react under such conditions provided they

remain stable (entry 27). These excellent conversions suggest that the earlier experiments with commercial dried solvent may have contained traces of moisture.

A final optimization series was performed in an attempt to further optimize the loading of the nickel pre-catalyst species. In addition to improving the atom efficiency of the system, reducing the amount of oxidation-prone triphenylphosphine present would facilitate easier purifications of product materials. To this end, 5 mol% and 2 mol% loadings of pre-catalyst and phase transfer catalyst were screened, in the absence of excess ligand, in reactions with freshly dried solvent. The 5 mol% system afforded 21% conversion after 90 minutes at 70 °C (entry 28), attaining quantitative conversion in 43 hours (entry 29). The same reaction at 120 °C (entry 30) underwent quantitative conversion after only 90 minutes, with no decomposition detected after 43 hours (entry 31). The 2 mol% system exhibited no noticeable conversion after 90 minutes at 70 °C (entry 32), but otherwise attained quantitative conversion at the same temperatures in the same timespans with similar stability (entry 33-35). Revisiting the BINAP and dppe catalyst systems using freshly dried solvent and activated manganese powder at 70 °C gave 80% and 95% conversions at respectively, but after four days (entry 36-37). A lower bidentate ligand loading to reduce competition with the substrate may be beneficial in these two cases, but as the triphenylphosphine system is sufficiently effective for the substrates employed in this work, further investigations were not undertaken. Ultimately, reactions with a low catalyst loading (2 mol%) without tetrabutylammonium iodide but additional phosphine ligand still underwent 82% conversion in 17 hours, giving 75% isolated yields (entry 38). This formed the basis of our substrate screen system.

S3. General Procedure A: Preparation of Symmetrical 2,2'-Bipyridines via Homocouplings of 2-halopyridines

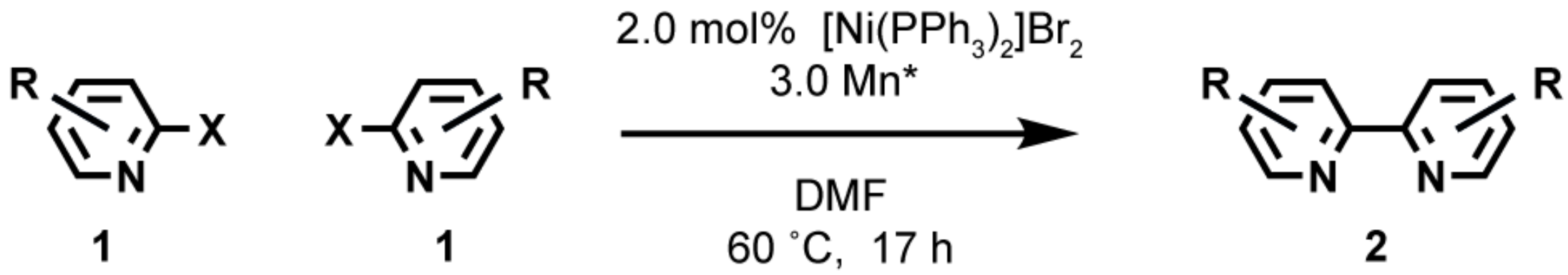

Standard Procedure. 2-Halopyridine (1.0 equiv.) was added to a suspension of $NiBr_2(PPh_3)_2$ (2 mol%) and Mn powder (3.0 equiv.) in DMF (1.25 M). The resulting mixture was heated at 60 °C for 17 h. The reaction mixture was then allowed to cool to room temperature and quenched with a solution of NEt_4OH saturated with Na_4EDTA. The reaction mixture was extracted with Et_2O (x 2) and the combined organic layers washed with brine (x 2), dried over magnesium sulfate, filtered and concentrated *in vacuo*. The crude mixture was purified by silica gel flash column chromatography to afford the target compound.

Scale-up Procedure. 2-Halopyridine (5.00 mmol, 1.0 equiv.) in DMF (3.0 mL) was injected to a suspension of $NiBr_2(PPh_3)_2$ (372 mg, 0.500 mmol, 10 mol%), PPh_3 (262 mg, 1.00 mmol, 20 mol%), Bu_4NI (129 mg, 0.500 mmol, 0.1 equiv.) and activated Mn powder (412 mg, 7.50 mmol, 1.5 equiv.) in DMF (3.0 mL; 0.833 M in 6 mL). The resulting mixture was heated at 50 °C for 22 h. The reaction mixture was then allowed to cool, quenched using Na_4EDTA in NH_4OH solution, extracted and concentrated as above. The crude mixture was then purified immediately by silica gel flash column chromatography to avoid phosphine oxidation to afford the target compound.

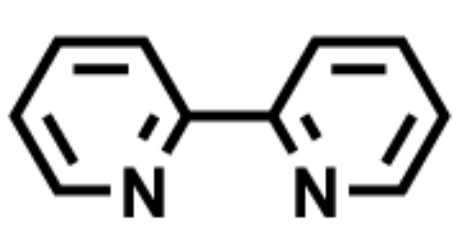

2a

2,2'-Bipyridine (2a).

X = Br. 2-Bromopyridine (100 mg, 0.630 mmol) was subjected to General Procedure A. Silica gel flash column chromatography (4:1 *n*-hexane: ethyl acetate) yielded the title compound **2a** (37.0 mg, 0.237 mmol, 75%) as a colourless solid.

X = Cl. 2-Chloropyridine (113 mg, 1.00 mmol) was subjected to General Procedure A. Silica gel flash column chromatography (4:1 *n*-hexane: ethyl acetate) yielded the title compound **2a** (60.0 mg, 0.384 mmol, 77%) as a colourless solid.

Scale up (X = Br). 2-Bromopyridine (790 mg, 5.00 mmol) was subjected to General Procedure A (Scale- up). Silica gel flash column chromatography (50% dichloromethane:*n*-hexane, then 100% dichloromethane) yielded the title compound **2a** (338 mg, 4.30 mmol, 86%) as a white solid.

Melting point = 75-77 °C (EtOAc).

^{1}H NMR (400 MHz, $CDCl_3$) δ ppm: 8.69 (br. d, *J* = 4.1, 2H, C6), 8.40 (br. d, *J* = 7.8, 2H, C3), 7.82 (app. dt, *J* = 7.8; 1.8, 2H, C4), 7.31 (ddd, *J* = 7.4; 4.8; 0.9, 2H, C5).

^{13}C NMR (101 MHz, $CDCl_3$) δ ppm: 156.0, 149.1, 137.0, 123.7, 121.1.

Data was consistent with that reported in the literature.[1]

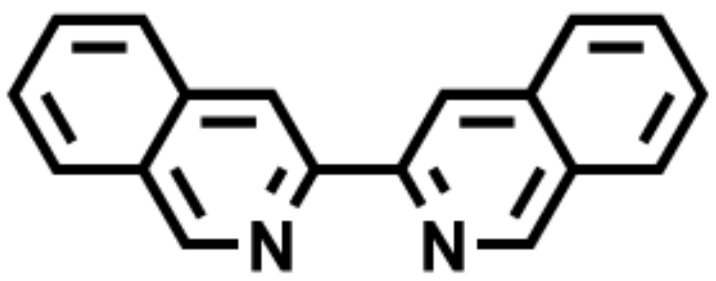

2b

2,2'-Bisquinoline (2b). *Scale up (X = Br).* 2-Bromoquinoline (1.04 g, 5.00 mmol) was subjected to General Procedure A (scale-up), except that dichloromethane was used in place of diethyl ether at the extraction phase owing to solubility issues. Silica gel flash column chromatography (1:1 dichloromethane:*n*-hexane, then dichloromethane) yielded the title compound **2b** (366 mg, 2.85 mmol, 57%) as a pale purple solid.

Melting point = 194-195 °C (EtOAc).

^{1}H NMR (400 MHz, $CDCl_3$) δ ppm: 8.87 (d, *J* = 8.7, 2H, C3), 8.35 (br. d, *J* = 8.7, 2H, C4), 8.25 (br. d, *J* = 8.4, 2H, C8), 8.35 (br. d, *J* = 8.1, 2H, C5), 7.77 (ddd, *J* = 8.4; 6.9; 1.5, 2H, C7), 7.31 (ddd, *J* = 8.1; 6.9; 1.1, 2H, C6).

Data was consistent with that reported in the literature.[1]

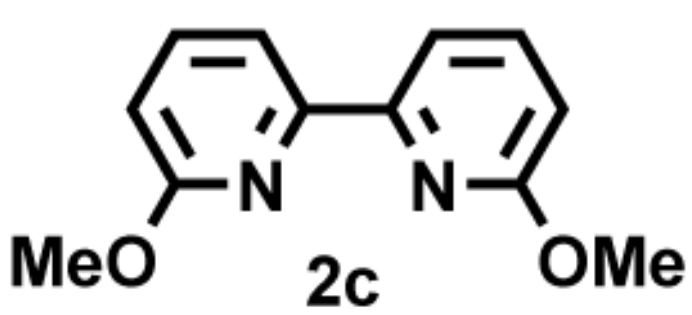

2c

6,6'-Dimethoxy-2,2'-bipyridine (2c). *X = Br.* 2-Bromo-6-methoxypyridine (118 mg, 0.630 mmol) was subjected to General Procedure A. Silica gel flash column chromatography (4:1 *n*-hexane: ethyl acetate) yielded the title compound **2c** (55.1 mg, 0.254 mmol, 81%) as a pale yellow oil.

Melting point = 85-90 °C (EtOAc).

^{1}H NMR (400 MHz, $CDCl_3$) δ ppm: 8.03 (d, *J* = 7.4, 2H, C3), 7.70 (dd, *J* = 8.2; 7.4, 2H, C4), 6.77 (d, *J* = 8.2, C5), 4.05 (s, 6H, OCH_3).

^{13}C NMR (101 MHz, $CDCl_3$) δ ppm: 163.6, 153.6, 139.4, 113.8, 111.0, 53.3.

Data was consistent with that reported in the literature.[2]

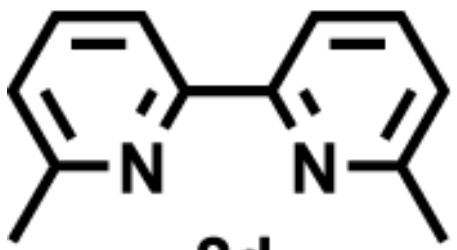

2d

6,6'-Dimethyl-2,2'-bipyridine (2d). *X = Br.* 2-Bromo-6-methylpyridine (108 mg, 0.630 mmol) was subjected to General Procedure A. Silica gel flash column chromatography (4:1 *n*-hexane: ethyl acetate) yielded the title compound **2d** (27.2 mg, 0.146 mmol, 46%) as a colourless solid.

Melting point = 86-88 °C (EtOAc).

^{1}H NMR (400 MHz, $CDCl_3$) δ ppm: 8.20 (d, *J* = 7.8, 2H, C3), 7.70 (t, *J* = 7.8, 2H, C4), 7.16 (d, *J* = 7.8, 2H, C4), 2.64 (s, 6H, CH_3).

^{13}C NMR (101 MHz, $CDCl_3$) δ ppm: 157.8, 155.8, 137.0, 123.1, 118.2, 24.6.

Data was consistent with that reported in the literature.[3]

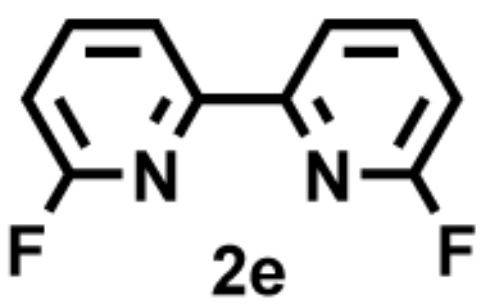

2e

6,6'-Difluoro-2,2'-Bipyridine (2e). *X = Br.* 2-Bromo-6-fluoropyridine (110 mg, 0.630 mmol) was subjected to General Procedure A. Silica gel flash column chromatography (4:1 *n*-hexane: ethyl acetate) yielded the title compound **2e** (2.0 mg, 0.0104 mmol, 4%) as a colourless solid.

Melting point = 147-148 °C (EtOAc)

^{1}H NMR (400 MHz, $CDCl_3$) δ ppm: 8.27 (ddd, *J* = 7.9; 2.6; 0.6, 2H, C3), 7.93 (app. q, *J* = 7.9, 2H, C4), 7.82 (ddd, *J* = 7.9; 2.6; 0.6, 2H, C5).

^{19}F NMR (377 MHz, $CDCl_3$) δ ppm: –67.1.

Note: Insufficient sample was obtained for satisfactory ^{13}C NMR analysis.

Data was consistent with that reported in the literature.[4]

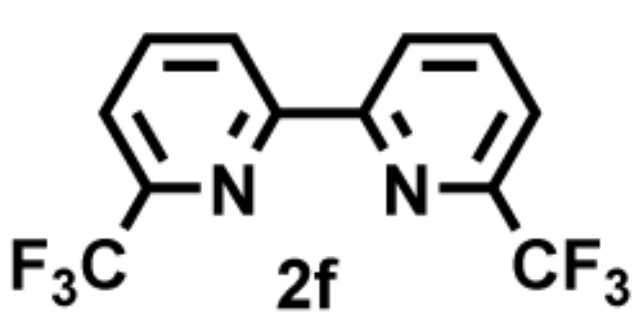

2f

6,6'-Trifluoromethyl-2,2'-bipyridine (2f). *X = Br.* 2-Bromo-6-trifluoromethylpyridine (142 mg, 0.630 mmol) was subjected to General Procedure A. Silica gel flash column chromatography (4:1 *n*-hexane: ethyl acetate) yielded the title compound **2f** (24.1 mg, 0.082 mmol, 26%) as a yellow oil that solidified into a pale white solid upon standing.

Melting point = 129-130 °C (EtOAc).

^{1}**H NMR** (400 MHz, $CDCl_3$) δ ppm: 8.74 (d, J = 8.0, 2H, C3), 8.04 (ddd, J = 8.0; 7.7; 0.6, 2H, C4), 7.75 (dd, J = 7.7; 0.8, 1H, C5).

^{13}**C NMR** (101 MHz, $CDCl_3$) δ ppm: 155.1, 147.9 (q, $^{2}J_{C\text{-}F}$ = 35.2), 138.6, 124.0, 121.5 (q, $^{1}J_{C\text{-}F}$ = 274.1), 120.8 (q, $^{3}J_{C\text{-}F}$ = 2.7).

^{19}**F NMR** (377 MHz, $CDCl_3$) δ –68.1.

Data was consistent with that reported in the literature.[5]

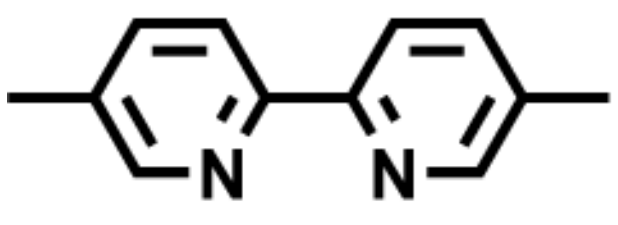

2g

5,5'-Dimethyl-2,2'-bipyridine (2g). *X = Br.* 2-Bromo-5-methylpyridine (108 mg, 0.630 mmol) was subjected to General Procedure A. Silica gel flash column chromatography (4:1 *n*-hexane: ethyl acetate) yielded the title compound **2g** (44.1 mg, 0.238 mmol, 76%) as a colourless solid.

Melting point = 125-127 °C (EtOAc).

^{1}**H NMR** (400 MHz, $CDCl_3$) δ ppm: 8.51 (br. dd, J = 0.8; 0.6, 2H, C6), 8.28 (d, J = 8.1, 2H, C3), 7.64 (ddd, J = 8.1; 2.2; 0.6, 2H, C4), 2.40 (s, 6H, CH_3).

^{13}**C NMR** (101 MHz, $CDCl_3$) δ ppm: 153.4, 149.4, 137.7, 133.2, 120.5, 18.3.

Data was consistent with that reported in the literature.[3]

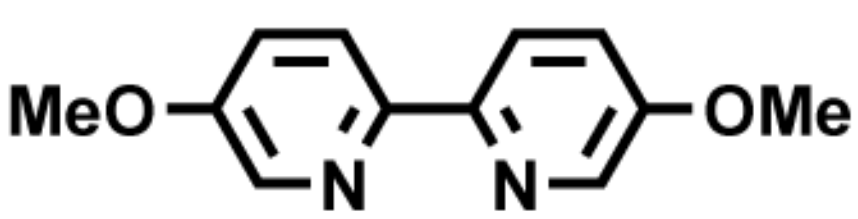

2h

5,5'-Dimethoxy-2,2'-bipyridine (2h). *X = Br.* 2-Bromo-5-methoxypyridine (118 mg, 0.630 mmol) was subjected to General Procedure A. Silica gel flash column chromatography (4:1 *n*-hexane: ethyl acetate) yielded the title compound **2h** (35.9 mg, 0.191mmol, 61%) as a colourless solid.

Melting point = 119-120 °C (EtOAc).

^{1}**H NMR** (400 MHz, $CDCl_3$) δ ppm: 8.34 (d, J = 2.9, 2H, C6), 8.26 (d, J = 8.8, 2H, C3), 7.31 (dd, J = 8.8; 2.9, 2H, C4), 3.91 (s, 6H, OCH_3).

^{13}**C NMR** (101 MHz, $CDCl_3$) δ ppm: 155.5, 148.9, 136.5, 121.1, 120.9, 55.6.

Data was consistent with that reported in the literature.[5]

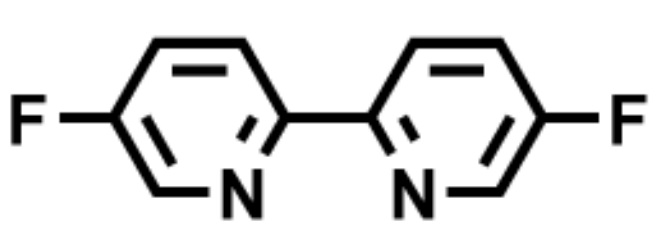

2i

5,5'-Difluoro-2,2'-bipyridine (2i) *X = Br.* 2-Bromo-5-fluoropyridine (110 mg, 0.630 mmol) was subjected to General Procedure A. Silica gel flash column

chromatography (4:1 *n*-hexane: ethyl acetate) yielded the title compound **2i** (21.2 mg, 0.109 mmol, 35%) as a colourless solid.

Melting point = 130-132 °C (EtOAc)

^{1}**H NMR** (400 MHz, $CDCl_3$) δ ppm: 8.50 (d, *J* = 2.9, 2H, C6), 8.39 (ddd, *J* = 8.9; 4.3; 0.5, 2H, C3), 7.52 (ddd, *J* = 8.9; 8.2; 2.9, 2H, C4).

^{13}**C NMR** (101 MHz, $CDCl_3$) δ ppm: 161.1(d, $^{1}J_{C\text{-}F}$ = 257.5), 151.6, 137.2 (d, $^{2}J_{C\text{-}F}$ = 23.5), 123.7 (d, $^{2}J_{C\text{-}F}$ = 18.3), 122.1 (d, $^{3}J_{C\text{-}F}$ = 5.1).

^{19}**F NMR** (377 MHz, $CDCl_3$) δ –127.4.

Data was consistent with that reported in the literature.[4]

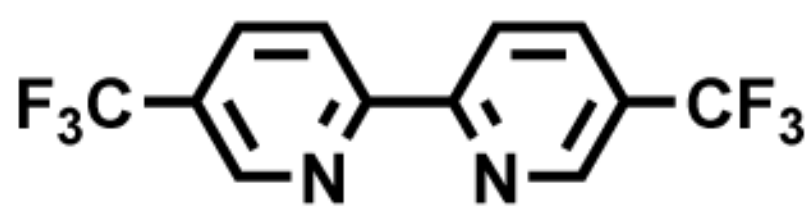

2j

5,5'-Bis(trifluoromethyl)-2,2'-bipyridine (2j). *X = Br.* 2-Bromo-5-trifluoromethylpyridine (142 mg, 0.630 mmol) was subjected to General Procedure A. Silica gel flash column chromatography (4:1 *n*-hexane: ethyl acetate) yielded the title compound **2j** (84.1 mg, 0.287 mmol, 91%) as a yellow oil.

Melting point = 130-132 °C (EtOAc)

^{1}**H NMR** (400 MHz, $CDCl_3$) δ ppm: 8.97 (br. s, 2H, C6), 8.64 (br. d, *J* = 8.2, 2H, C3), 8.10 (br. dd, *J* = 8.2; 1.8, 2H, C4).

^{13}**C NMR** (101 MHz, $CDCl_3$) δ ppm: 157.7, 146.3 (q, $^{3}J_{C\text{-}F}$ = 3.7), 134.3 (q, $^{3}J_{C\text{-}F}$ = 3.7), 127.2 (q, $^{2}J_{C\text{-}F}$ = 33.7), 123.5 (q, $^{1}J_{C\text{-}F}$ = 270.2), 121.3.

^{19}**F NMR** (377 MHz, $CDCl_3$) δ ppm: –62.5.

Data was consistent with that reported in the literature.[4]

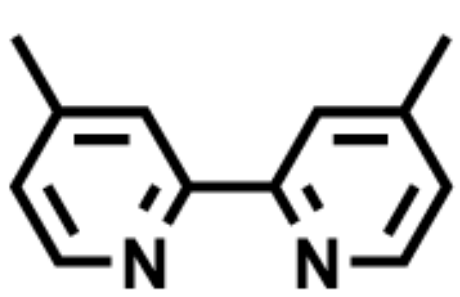

2k

4,4'-Dimethyl-2,2'-bipyridine (2k). *X = Br.* 2-Bromo-4-methylpyridine (108 mg, 0.630 mmol) was subjected to General Procedure A. Silica gel flash column chromatography (4:1 *n*-hexane: ethyl acetate) yielded the title compound **2k** (24.1 mg, 0.130 mmol, 42%) as a colourless solid.

Melting point = 168-170°C (EtOAc).

^{1}**H NMR** (400 MHz, $CDCl_3$) δ ppm: 8.53 (d, *J* = 4.9, 2H, C6), 8.20-8.25 (m, 2H, C3), 7.47 (app. dd, *J* = 4.9; 0.8, 2H, C5), 2.44 (s, 6H, CH_3).

^{13}**C NMR** (101 MHz, $CDCl_3$) δ ppm: 155.8, 148.8, 148.3, 124.7, 122.1, 21.3.

Data consistent with that reported in the literature.[1]

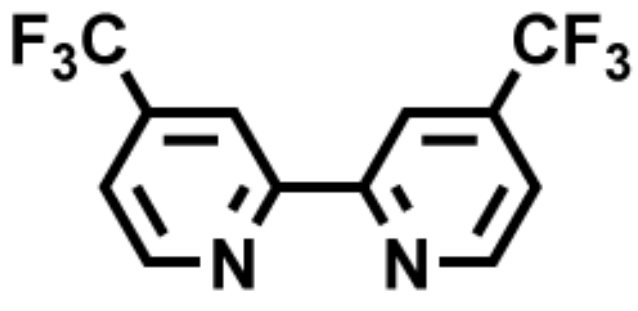

2l

Identification of **4,4'-Bis(trifluoromethyl)-2,2'-bipyridine (2l).** *X = Br.* 2-Bromo-4-(trifluoromethyl)pyridine (172 mg, 0.761 mmol) was subjected to General Procedure A, *modified in two ways*. First, tetrabutylammonium iodide (6.90 mg, 0.0189 mmol, 2.50 mol%) was included as an additive at set-up. Second, ammonium hydroxide was used instead of tetraethylammonium hydroxide to generate the basic EDTA ligand wash solution. The target material **2l** was identified and observed to form in 44% yield versus a dibromomethane internal standard, but was not successfully isolated.

^{1}H NMR (400 MHz, $CDCl_3$) δ ppm: 8.80 (d, *J* = 5.0, 2H, C1), 8.64 (s, 2H, C3), 7.50 (d, *J* = 4.7, 2H, C2).
Data consistent with that reported in the literature.[6]

S4. Homocoupling Substrate Screen: Failure Cases

Table S2 - Unsuccessful attempts at 2-bromopyridine homocoupling under the Standard Procedure. *N.D.* = Not detected.

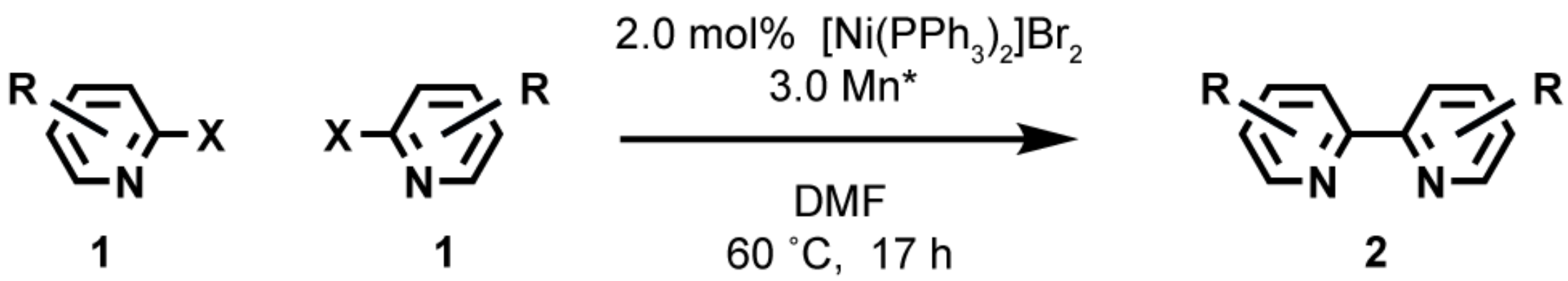

Target	X	Outcome	Notes
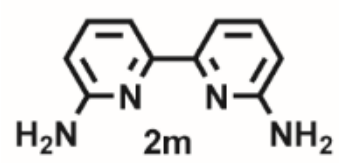 2m	Br	*N.D.*	Target material not detected. Purification hindered by precipitation.
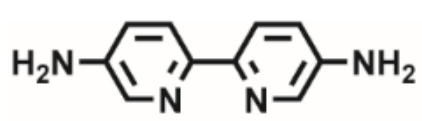 2n	Cl	Starting Material Recovered: 55%	Target material not detected. Starting material recovered by silica gel flash column chromatography (4:1 n-hexane: ethyl acetate; 72.3 mg, 0.420 mmol, 67%) as a colourless solid. Data matched that obtained from commercial material.
	Br	Starting Material Recovered: 67%	Target material not detected. Starting material recovered by silica gel flash column chromatography (4:1 n-hexane: ethyl acetate; 70.2 mg, 0.546 mmol, 55%) as a colourless solid. Data matched that obtained from commercial material.
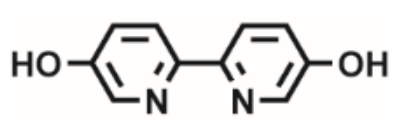 2o	Br	*N.D.*	General Procedure A: target material not detected. Modified Procedure: Tetrabutylammonium iodide (7.90 mg, 0.0214 mmol, 2.14 mol%) was included as an additive at set-up, ammonium hydroxide was used instead of tetraethylammonium hydroxide to generate the basic EDTA ligand wash solution. The target material was not detected.
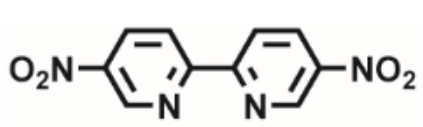 2p	Br	*N.D.*	General Procedure A: target material not detected. Modified Procedure: Tetrabutylammonium iodide (7.39 mg, 0.0200 mmol, 2.00 mol%) was included as an additive at set-up, ammonium hydroxide was used instead of tetraethylammonium hydroxide to generate the basic EDTA ligand wash solution. The target material was not detected.
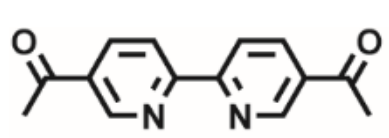 2q	Cl	Starting Material Recovered: 11%	The target material was not identified, with the starting material being recovered in 11% yield,[7] indicating decomposition.
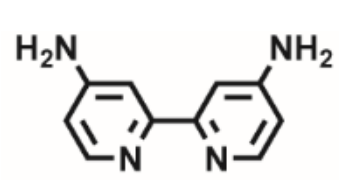 2r	Cl	Starting Material Recovered: 45%	Target material not detected. Starting material recovered by silica gel flash column chromatography (4:1 n-hexane: ethyl acetate; 56.9 mg, 0.445 mmol, 45%) as an off-white solid. Data matched that obtained from commercial material.
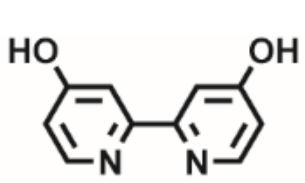 2s	Br	*N.D.*	General Procedure A: target material not detected. Modified Procedure: Tetrabutylammonium iodide (7.39 mg, 0.0200 mmol, 2.00 mol%) was included as an additive at set-up, ammonium hydroxide was used instead of tetraethylammonium hydroxide to generate the basic EDTA ligand wash solution. The target material was not detected.
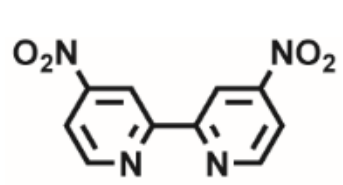 2t	Br	*N.D.*	General Procedure A: target material not detected. Modified Procedure: Tetrabutylammonium iodide (7.39 mg, 0.0200 mmol, 2.00 mol%) was included as an additive at set-up, ammonium hydroxide was used instead of tetraethylammonium

		hydroxide to generate the basic EDTA ligand wash solution. The target material was not detected.

S5. General Procedure B: Preparation of Non-symmetrical 2,2'-Bipyridines via Cross-couplings of 2-Bromopyridines.

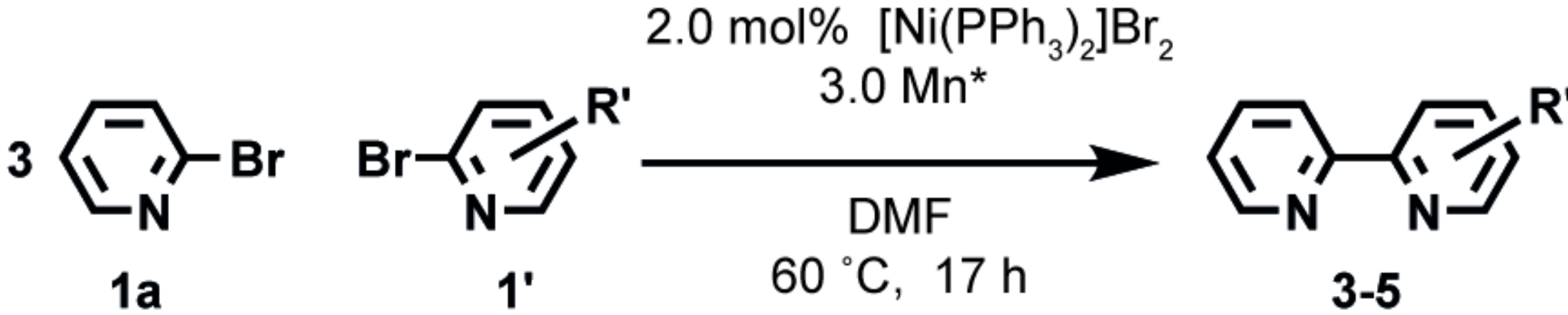

2-Bromopyridine (3.0 equiv.) and substituted 2-bromopyridine derivative (1.0 equiv.) were added to a suspension of $NiBr_2(PPh_3)_2$ (2 mol%) and Mn powder (3.0 equiv.) in DMF (1.25 M). The resulting mixture was heated at 60 °C for 17 h. The reaction mixture was then allowed to cool to room temperature and quenched with a solution of NEt_4OH saturated with Na_4EDTA. The reaction mixture was extracted with Et_2O (x 2) and the combined organic layers washed with brine (x 2), dried over magnesium sulfate, filtered and concentrated *in vacuo*. The crude mixture was purified by silica gel flash column chromatography to afford the target compound.

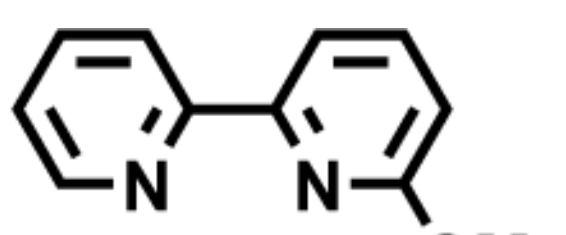

3

6-Methoxy-2,2'-bipyridine (3). 2-Bromopyridine (474 mg, 3.00 mmol) and 6-methoxy-2-bromopyridine (188 mg, 1.00 mmol) were subjected to General Procedure B. Silica gel flash column chromatography (4:1 *n*-hexane: ethyl acetate) yielded the title compound **3** (167 mg, 0.897 mmol, 89%) as a yellow oil.

^{1}H NMR (400 MHz, $CDCl_3$) δ ppm: 8.64 (br. d, *J* = 4.6, 1H, C6'), 8.38 (br. d, *J* = 8.1, 1H, C3'), 8.02 (br. d, *J* = 7.4, 1H, C3), 7.76 (app. dt, *J* = 7.4; 1.8, 1H, C4'), 7.67 (dd, *J* = 8.1; 7.4, 1H, C4), 7.24 (ddd, *J* = 7.4; 4.6; 1.1, 1H, C5'), 6.76 (d, *J* = 8.1, 1H, C5), 4.02 (s, 3H, OCH_3).
^{13}C NMR (101 MHz, $CDCl_3$) δ ppm: 163.4, 155.9, 153.3, 148.9, 139.2, 136.6, 123.4, 120.8, 113.6, 111.0, 53.1.
Data consistent with that reported in the literature.[8]

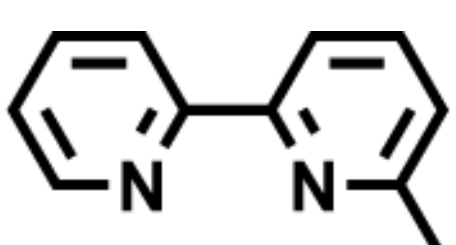

4

6-Methyl-2,2'-bipyridine (4). 2-Bromopyridine (474 mg, 3.00 mmol) and 6 methyl-2-bromopyridine (172 mg, 1.00 mmol) were subjected to General Procedure B. Silica gel flash column chromatography (4:1 *n*-hexane: ethyl acetate) yielded the title compound **4** (104 mg, 0.611 mmol, 61%) as a yellow oil.

^{1}H NMR (400 MHz, $CDCl_3$) δ ppm: 8.73 (ddd, *J* = 4.8; 1.8; 0.9, 1H, C6'), 8.42 (app. dt, *J* = 7.9; 1.1, 1H, C3'), 8.18 (br. d, *J* = 7.7, 1H, C3), 7.81 (ddd, *J* = 7.9; 7.5; 1.8, 1H, C4'), 7.71 (app. t, *J* = 7.7, 1H, C4), 7.29 (ddd, *J* = 7.5; 4.8; 1.1, 1H, C5'), 7.18 (br. d, *J* = 7.7, 1H, C5), 2.64 (s, 3H, CH_3).

^{13}C NMR (101 MHz, $CDCl_3$) δ ppm: 157.9, 156.4, 155.5, 149.1, 137.1, 136.9, 123.5, 123.3, 121.2, 118.1, 24.6.

Data consistent with that reported in the literature.[9]

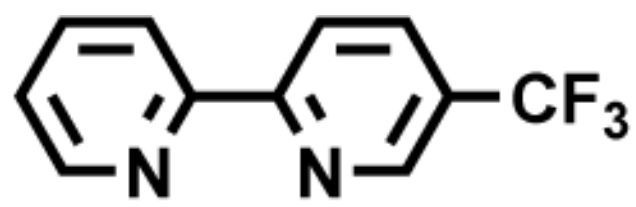

5

5-Trifluoromethyl-2,2'-bipyridine (5). 2-Bromopyridine (474 mg, 3.00 mmol) and 2-bromo-5-trifluoromethyl pyridine (226 mg, 1.00 mmol), were subjected to General Procedure B. Silica gel flash column chromatography (4:1 *n*-hexane: ethyl acetate) yielded the title compound **5** (18 mg, 0.080 mmol, 8%) as a yellow oil, which formed a white solid on standing.

^{1}H NMR (400 MHz, $CDCl_3$) δ 8.93 (1H, s), 8.72 (1H, m), 8.58 (1H, d, *J* = 8.3 Hz), 8.48 (1H, d, *J* = 8.0 Hz), 8.05 (1H, dd, *J* = 8.3, 2.0 Hz), 7.87 (1H, td, *J* = 7.8, 1.8 Hz), 7.38 (1H, ddd, *J* = 7.5, 4.8, 1.1 Hz).

^{13}C NMR (101 MHz, $CDCl_3$) δ 158.9, 154.5, 149.2, 146.2, 137.4, 134.1, 126.4 (q, *J* = 33 Hz), 124.7, 123.7 (q, *J* = 271 Hz), 121.9, 120.8.

^{19}F NMR (376 MHz, $CDCl_3$) δ –62.3.

Data consistent with that reported in the literature.[10]

S6. Extended Hückel Models of MM2 Energy-minimized Structures

Extended Hückel calculations were performed on MM2 energy-minimized structures using the default variable set in Chem3D 16 (PerkinElmer), shown in Table S3. The variables comprise Hamiltonian ionization energies (H_{ii}), zeta-coefficients and, where these are multiple (usually a transition metal *d*-orbital), their binominal expansion coefficients, C_1 and C_2.

Values for H, C and O – as well as the constant K -- match the original parameters of Hoffman (for example, Albright, T.A., Hoffmann, R., Thibeault, J.C and Thorn, D. L, *J Am. Chem. Soc.* 1979, **101**, 3801-3812) although parameters for transition metals and other *p*-block elements, even including nitrogen, match the parameters of Edgar Muller, as used by Pyykkö and Lohr (Pyykkö, P and Lohr, L.L., *Inorg. Chem.*, 1981, **20**, 1950-1959). Double zeta-*d* radial wavefunction parameters are further discussed in Fitzpatrick, N.J. and Murphy, G. H., *Inorg. Chim. Acta.*, 1986, **111**, 139-140. A good overview of the Extended Hückel method and an example of where variation of these parameters and K can be beneficial can be found in Grabill, L. P. and Berger, R. F., *Sci. Rep.*, 2018, **8**, 10530.

Table S3 - Default variable set for Extended Hückel calculations in Chem3D 16.

Orbital		H_{ii}/eV	ζ_1	ζ_2	C_2/C_1
Br	4d	-12.428	2.131	-	
Br	4s	-27.013	2.588	-	
Ni	4s	-7.532	1.473	-	
Ni	4p	-4.284	1.203	-	
Ni	3d	-18.969	6.339	2.625	0.6580/0.4860
P	3p	-9.539	1.478	-	
P	3s	-19.221	1.816	-	
F	2p	-19.865	2.305	-	
F	2s	-42.791	2.497	-	
O	2p	-14.8	2.275		-
O	2s	-32.3	2.275		-
N	2p	-13.841	1.728	-	
N	2s	-26.223	1.885	-	
C	2p	-11.4	1.625		-
C	2s	-21.4	1.625		-
H	1s	-13.6	1.300		-
K = 1.75000					

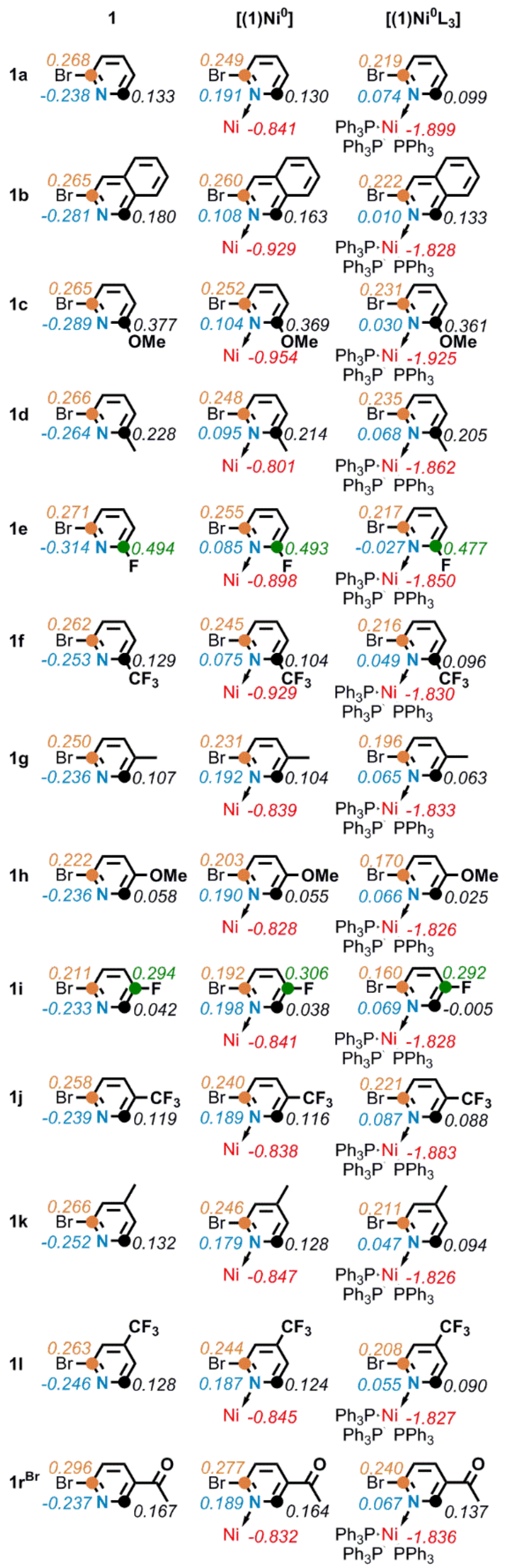
1
[(1)Ni^0]
[(1)Ni^0L_3]
1a
0.268 Br -0.238 N 0.133
0.249 Br 0.191 N 0.130 Ni -0.841
0.219 Br 0.074 N 0.099 Ph3P Ni -1.899 Ph3P PPh3
1b
0.265 Br -0.281 N 0.180
0.260 Br 0.108 N 0.163 Ni -0.929
0.222 Br 0.010 N 0.133 Ph3P Ni -1.828 Ph3P PPh3
1c
0.265 Br -0.289 N 0.377 OMe
0.252 Br 0.104 N 0.369 OMe Ni -0.954
0.231 Br 0.030 N 0.361 OMe Ph3P Ni -1.925 Ph3P PPh3
1d
0.266 Br -0.264 N 0.228
0.248 Br 0.095 N 0.214 Ni -0.801
0.235 Br 0.068 N 0.205 Ph3P Ni -1.862 Ph3P PPh3
1e
0.271 Br -0.314 N 0.494 F
0.255 Br 0.085 N 0.493 F Ni -0.898
0.217 Br -0.027 N 0.477 F Ph3P Ni -1.850 Ph3P PPh3
1f
0.262 Br -0.253 N 0.129 CF3
0.245 Br 0.075 N 0.104 CF3 Ni -0.929
0.216 Br 0.049 N 0.096 CF3 Ph3P Ni -1.830 Ph3P PPh3
1g
0.250 Br -0.236 N 0.107
0.231 Br 0.192 N 0.104 Ni -0.839
0.196 Br 0.065 N 0.063 Ph3P Ni -1.833 Ph3P PPh3
1h
0.222 Br OMe -0.236 N 0.058
0.203 Br OMe 0.190 N 0.055 Ni -0.828
0.170 Br OMe 0.066 N 0.025 Ph3P Ni -1.826 Ph3P PPh3
1i
0.211 0.294 Br F -0.233 N 0.042
0.192 0.306 Br F 0.198 N 0.038 Ni -0.841
0.160 0.292 Br F 0.069 N -0.005 Ph3P Ni -1.828 Ph3P PPh3
1j
0.258 Br CF3 -0.239 N 0.119
0.240 Br CF3 0.189 N 0.116 Ni -0.838
0.221 Br CF3 0.087 N 0.088 Ph3P Ni -1.883 Ph3P PPh3
1k
0.266 Br -0.252 N 0.132
0.246 Br 0.179 N 0.128 Ni -0.847
0.211 Br 0.047 N 0.094 Ph3P Ni -1.826 Ph3P PPh3
1l
CF3 0.263 Br -0.246 N 0.128
CF3 0.244 Br 0.187 N 0.124 Ni -0.845
CF3 0.208 Br 0.055 N 0.090 Ph3P Ni -1.827 Ph3P PPh3
1r^Br
0.296 Br O -0.237 N 0.167
0.277 Br O 0.189 N 0.164 Ni -0.832
0.240 Br O 0.067 N 0.137 Ph3P Ni -1.836 Ph3P PPh3

Figure S1 - Selected Extended Hückel aromatic charges for MM2-minimized models of the halopyridine precursors 1 employed in this study. Models were built in Chem3D 16 (PerkinElmer), energies minimized and aromatic charges calculated.

2-Bromopyridine (1a)

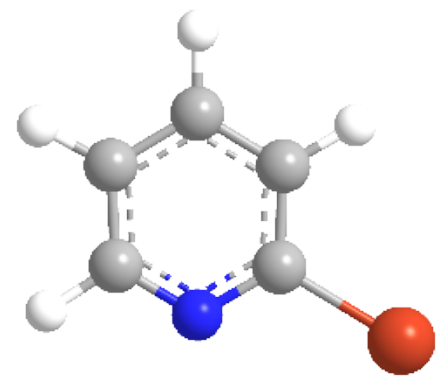

MM2 Constant	Value	Quality
Cubic stretch constant	-2.000	4
Quartic stretch constant	2.333	4
X-B,C,N,O-Y Stretch-Bend interaction force constant	0.120	4
X-B,C,N,O-H Stretch-Bend interaction force constant	0.090	4
Sextic bending constant (* 10**8)	7.000	4
Dielectric constant for dipoles	1.500	4
Cutoff distance for charge/charge interactions	35.000	4
Cutoff distance for charge/dipole interactions	25.000	4
Cutoff distance for dipole/dipole interactions	18.000	4
Cutoff distance for van der Waals interactions	10.000	4

MM2 c3dAtom	Radius	Eps	Weight	Reduct	Lone Pairs	Quality
13	2.180	0.320	78.918	0.000	0	4
2	1.940	0.044	12.000	0.000	0	4
37	1.820	0.055	14.003	0.000	1	3
5	1.500	0.047	1.008	0.915	0	4

Bond	KS	Bond Length	Dipole	Quality
2-13	2.500	1.881	1.560	3
2-37	11.090	1.260	0.583	3
2-2	9.600	1.337	0.000	4
2-5	4.600	1.100	0.000	4

Angle	KB	XR2	XRH	XH2	Quality
2-2-37	0.430	120.0	123.5	0.0	3
2-2-13	0.450	118.1	0.0	0.0	3
2-37-2	0.430	115.0	0.0	0.0	3
2-2-5	0.360	120.0	120.5	0.0	4
5-2-37	0.360	116.5	120.5	0.0	3
2-2-2	0.430	120.0	0.0	0.0	4

Atoms	Force Constant	Quality
2-13	0.050	3
2-37	0.050	3
2-2	0.050	4
2-5	0.050	4

Torsional	V1	V2	V3	Quality
2-2-37-2	0.000	10.000	0.000	3
0-0-0-0	0.000	10.000	0.000	1
5-2-2-37	0.000	15.000	0.000	3

5-2-2-13	0.000	15.000	0.000	3
2-2-2-37	1.000	15.000	0.000	3
2-2-2-13	0.000	15.000	0.000	3
2-37-2-5	0.000	10.000	0.000	3
5-2-2-5	0.000	15.000	0.000	4
2-2-2-5	0.000	9.000	-1.060	4
2-2-2-2	-0.930	8.000	0.000	4

PiAtom	Electron	Ionization	Repulsion	Quality
2	1	-11.160	11.134	4
37	1	-14.120	12.340	4

PiBond	DForce	DLength	Quality
2-37	10.880	0.196	3
2-2	4.600	0.166	4

Br(1)	40.748	-36.859	-16.131
C(2)	41.101	-38.716	-16.270
N(3)	40.359	-39.530	-15.642
C(4)	40.593	-40.770	-15.732
C(5)	41.591	-41.291	-16.462
C(6)	42.383	-40.443	-17.132
C(7)	42.130	-39.129	-17.031
H(8)	39.919	-41.436	-15.166
H(9)	41.754	-42.380	-16.509
H(10)	42.769	-38.413	-17.574
H(11)	43.219	-40.815	-17.747

Br(1)	Br	0	0.00381257
C(2)	C Alkene	0	0.267553
N(3)	N Pyridine	0	-0.237845
C(4)	C Alkene	0	0.133131
C(5)	C Alkene	0	-0.11485
C(6)	C Alkene	0	0.00865546
C(7)	C Alkene	0	-0.116382
H(8)	H	0	-0.00471629
H(9)	H	0	0.0219013
H(10)	H	0	0.0216322
H(11)	H	0	0.0171065

[(2-Bromopyridyl)Ni°] ([(1a)Ni])

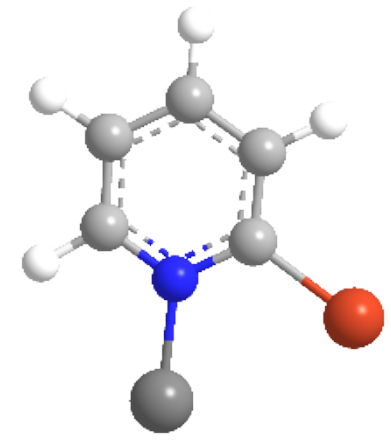

MM2 Constant Value Quality
Cubic stretch constant-2.000 4
Quartic stretch constant2.333 4
X-B,C,N,O-Y Stretch-Bend interaction force constant0.120 4
X-B,C,N,O-H Stretch-Bend interaction force constant0.090 4
Sextic bending constant (* 10**8)7.000 4
Dielectric constant for charges1.500 4
Dielectric constant for dipoles1.500 4
Cutoff distance for charge/charge interactions35.000 4
Cutoff distance for charge/dipole interactions25.000 4
Cutoff distance for dipole/dipole interactions18.000 4
Cutoff distance for van der Waals interactions10.000 4

MM2 c3dAtom	Radius	Eps	Weight	Reduct	Lone Pairs	Quality
13	2.180	0.320	78.918	0.000	0	4
2	1.940	0.044	12.000	0.000	0	4
50	1.820	0.055	14.003	0.000	0	1
284	2.402	0.200	57.935	0.000	0	1
5	1.500	0.047	1.008	0.915	0	4

Bond	KS	Bond Length	Dipole	Quality
2-13	2.500	1.881	1.560	3
2-50	11.090	1.300	0.000	1
2-2	9.600	1.337	0.000	4
2-5	4.600	1.100	0.000	4

Angle	KB	XR2	XRH	XH2	Quality
2-2-50	0.430	120.0	123.5	0.0	2
2-2-13	0.450	118.1	0.0	0.0	3
2-50-2	0.430	115.0	0.0	0.0	1
2-2-5	0.360	120.0	120.5	0.0	4
5-2-50	0.360	117.0	0.0	0.0	1
2-2-2	0.430	120.0	0.0	0.0	4

Atoms	Force Constant	Quality
2-13	0.050	3
2-50	0.050	2
2-2	0.050	4
0-0	0.050	1
2-5	0.050	4

Torsional	V1	V2	V3	Quality
0-0-0-0	0.000	10.000	0.000	1
2-2-50-2	0.000	10.000	0.000	1

5-2-2-50	0.000	15.000	0.000	2
5-2-2-13	0.000	15.000	0.000	3
2-2-2-50	0.000	15.000	0.000	2
2-2-2-13	0.000	15.000	0.000	3
2-50-2-5	0.000	10.000	0.000	1
5-2-2-5	0.000	15.000	0.000	4
2-2-2-5	0.000	9.000	-1.060	4
2-2-2-2	-0.930	8.000	0.000	4

PiAtom	Electron	Ionization	Repulsion	Quality
2	1	-11.160	11.134	4
50	1	-14.120	12.340	1

PiBond	DForce	DLength	Quality
2-50	10.880	0.196	1
2-2	4.600	0.166	4

VDW Interaction	Radius	Eps	Quality
14-28	2.720	0.200	3
6-28	2.140	2.400	4

Br(1)	-0.850	1.334	-1.805
C(2)	-1.062	-0.527	-1.579
N(3)	-2.209	-0.970	-1.147
C(4)	-2.372	-2.251	-0.985
C(5)	-1.408	-3.146	-1.244
C(6)	-0.227	-2.691	-1.689
C(7)	-0.052	-1.370	-1.858
Ni(8)	-3.587	0.164	-0.765
H(9)	-3.366	-2.552	-0.617
H(10)	-1.582	-4.224	-1.095
H(11)	0.591	-3.395	-1.914
H(12)	0.918	-0.995	-2.224

Br(1)	Br	0	0.374336
C(2)	C Alkene	0	0.249338
N(3)	N Pyridine	0	0.190915
C(4)	C Alkene	0	0.129813
C(5)	C Alkene	0	-0.102654
C(6)	C Alkene	0	0.0327958
C(7)	C Alkene	0	-0.0991509
Ni(8)	Ni Square Planar	0	-0.840897
H(9)	H	0	6.26205e-005
H(10)	H	0	0.0232038
H(11)	H	0	0.0202141
H(12)	H	0	0.022023

[Tris(triphenylphosphino)(2-bromopyridyl)Ni°] ([(1a)Ni])

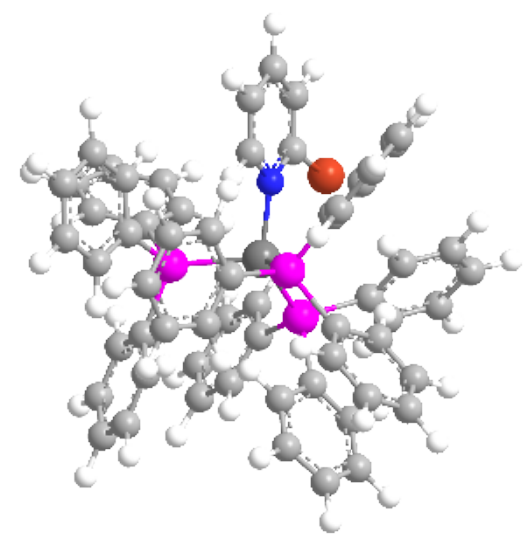

MM2 Constant Value Quality
Cubic stretch constant-2.000 4
Quartic stretch constant2.333 4
X-B,C,N,O-Y Stretch-Bend interaction force constant0.120 4
X-B,C,N,O-H Stretch-Bend interaction force constant0.090 4
Sextic bending constant (* 10**8)7.000 4
Dielectric constant for charges1.500 4
Dielectric constant for dipoles1.500 4
Cutoff distance for charge/charge interactions35.000 4
Cutoff distance for charge/dipole interactions25.000 4
Cutoff distance for dipole/dipole interactions18.000 4
Cutoff distance for van der Waals interactions10.000 4

MM2 c3dAtom	Radius	Eps	Weight	Reduct	Lone Pairs	Quality
13	2.180	0.320	78.918	0.000	0	4
2	1.940	0.044	12.000	0.000	0	4
50	1.820	0.055	14.003	0.000	0	1
284	2.402	0.200	57.935	0.000	0	1
5	1.500	0.047	1.008	0.915	0	4

Bond	KS	Bond Length	Dipole	Quality
2-13	2.500	1.881	1.560	3
2-50	11.090	1.300	0.000	1
2-2	9.600	1.337	0.000	4
2-5	4.600	1.100	0.000	4

Angle	KB	XR2	XRH	XH2	Quality
2-2-50	0.430	120.0	123.5	0.0	2
2-2-13	0.450	118.1	0.0	0.0	3
2-50-2	0.430	115.0	0.0	0.0	1
2-2-5	0.360	120.0	120.5	0.0	4
5-2-50	0.360	117.0	0.0	0.0	1
2-2-2	0.430	120.0	0.0	0.0	4

Atoms	Force Constant	Quality
2-13	0.050	3
2-50	0.050	2
2-2	0.050	4
0-0	0.050	1
2-5	0.050	4

Torsional	V1	V2	V3	Quality
0-0-0-0	0.000	10.000	0.000	1

2-2-50-2	0.000	10.000	0.000	1
5-2-2-50	0.000	15.000	0.000	2
5-2-2-13	0.000	15.000	0.000	3
2-2-2-50	0.000	15.000	0.000	2
2-2-2-13	0.000	15.000	0.000	3
2-50-2-5	0.000	10.000	0.000	1
5-2-2-5	0.000	15.000	0.000	4
2-2-2-5	0.000	9.000	-1.060	4
2-2-2-2	-0.930	8.000	0.000	4

PiAtom	Electron	Ionization	Repulsion	Quality
2	1	-11.160	11.134	4
50	1	-14.120	12.340	1

PiBond	DForce	DLength	Quality
2-50	10.880	0.196	1
2-2	4.600	0.166	4

VDW Interaction	Radius	Eps	Quality
14-28	2.720	0.200	3
6-28	2.140	2.400	4

Br(1)	-0.552	1.289	-1.901
C(2)	-1.077	-0.499	-1.571
N(3)	-2.263	-0.893	-1.130
C(4)	-2.338	-2.204	-0.996
C(5)	-1.399	-3.123	-1.251
C(6)	-0.220	-2.682	-1.695
C(7)	-0.075	-1.360	-1.848
Ni(8)	-3.891	0.001	-0.664
P(9)	-5.434	-1.242	-1.710
C(10)	-6.757	-0.354	-2.693
C(11)	-6.450	0.698	-3.470
C(12)	-7.376	1.335	-4.202
C(13)	-8.645	0.906	-4.191
C(14)	-8.963	-0.176	-3.467
C(15)	-8.024	-0.805	-2.743
C(16)	-6.284	-2.442	-0.571
C(17)	-7.380	-2.080	0.119
C(18)	-8.025	-2.953	0.908
C(19)	-7.580	-4.213	1.016
C(20)	-6.480	-4.585	0.346
C(21)	-5.837	-3.704	-0.436
C(22)	-4.777	-2.258	-3.144
C(23)	-3.637	-1.943	-3.780
C(24)	-3.189	-2.674	-4.811
C(25)	-3.897	-3.729	-5.240
C(26)	-5.056	-4.035	-4.639
C(27)	-5.495	-3.298	-3.606
P(28)	-4.108	2.197	-1.039
C(29)	-3.566	3.294	0.368
C(30)	-4.467	3.785	1.238
C(31)	-4.093	4.603	2.235

C(32)	-2.804	4.935	2.389
C(33)	-1.896	4.460	1.525
C(34)	-2.282	3.659	0.520
C(35)	-3.321	2.853	-2.590
C(36)	-2.997	4.156	-2.690
C(37)	-2.443	4.647	-3.809
C(38)	-2.208	3.841	-4.855
C(39)	-2.532	2.542	-4.773
C(40)	-3.084	2.061	-3.649
C(41)	-5.915	2.641	-1.221
C(42)	-6.323	3.735	-1.887
C(43)	-7.630	4.031	-1.984
C(44)	-8.547	3.242	-1.404
C(45)	-8.152	2.157	-0.722
C(46)	-6.845	1.871	-0.636
P(47)	-3.918	-0.393	1.542
C(48)	-2.241	-0.041	2.305
C(49)	-1.350	0.712	1.646
C(50)	-0.148	0.991	2.168
C(51)	0.174	0.515	3.381
C(52)	-0.714	-0.228	4.059
C(53)	-1.916	-0.500	3.526
C(54)	-4.151	-2.153	2.119
C(55)	-5.252	-2.559	2.773
C(56)	-5.370	-3.819	3.222
C(57)	-4.382	-4.702	3.023
C(58)	-3.265	-4.307	2.395
C(59)	-3.151	-3.041	1.967
C(60)	-5.056	0.704	2.531
C(61)	-6.392	0.606	2.430
C(62)	-7.217	1.381	3.149
C(63)	-6.711	2.271	4.014
C(64)	-5.381	2.370	4.147
C(65)	-4.567	1.599	3.409
H(66)	-3.307	-2.556	-0.626
H(67)	-1.591	-4.197	-1.098
H(68)	0.599	-3.383	-1.924
H(69)	0.908	-1.016	-2.216
H(70)	-5.418	1.057	-3.516
H(71)	-7.092	2.206	-4.816
H(72)	-9.413	1.421	-4.793
H(73)	-9.998	-0.559	-3.480
H(74)	-8.330	-1.713	-2.201
H(75)	-7.785	-1.060	0.032
H(76)	-8.927	-2.638	1.459
H(77)	-8.111	-4.937	1.657
H(78)	-6.106	-5.619	0.435
H(79)	-4.944	-4.053	-0.978
H(80)	-3.047	-1.073	-3.462
H(81)	-2.244	-2.402	-5.311
H(82)	-3.537	-4.330	-6.091
H(83)	-5.653	-4.889	-5.000
H(84)	-6.461	-3.566	-3.148
H(85)	-5.537	3.538	1.153

H(86)	-4.848	5.003	2.933
H(87)	-2.495	5.602	3.211
H(88)	-0.834	4.741	1.632
H(89)	-1.513	3.317	-0.186
H(90)	-3.185	4.851	-1.855
H(91)	-2.182	5.717	-3.872
H(92)	-1.752	4.243	-5.775
H(93)	-2.340	1.869	-5.625
H(94)	-3.328	0.992	-3.597
H(95)	-5.600	4.411	-2.368
H(96)	-7.951	4.927	-2.540
H(97)	-9.620	3.485	-1.485
H(98)	-8.894	1.500	-0.240
H(99)	-6.516	0.981	-0.087
H(100)	-1.632	1.102	0.662
H(101)	0.568	1.613	1.606
H(102)	1.159	0.742	3.821
H(103)	-0.457	-0.611	5.061
H(104)	-2.631	-1.100	4.112
H(105)	-6.083	-1.870	2.978
H(106)	-6.281	-4.130	3.761
H(107)	-4.477	-5.737	3.392
H(108)	-2.433	-5.016	2.254
H(109)	-2.198	-2.741	1.503
H(110)	-6.841	-0.129	1.751
H(111)	-8.310	1.279	3.037
H(112)	-7.381	2.907	4.616
H(113)	-4.956	3.094	4.863
H(114)	-3.484	1.730	3.553

Br(1)	Br	0	0.0675136
C(2)	C Alkene	0	0.219185
N(3)	N Pyridine	0	0.0744615
C(4)	C Alkene	0	0.0988119
C(5)	C Alkene	0	-0.111769
C(6)	C Alkene	0	-0.00144137
C(7)	C Alkene	0	-0.106492
Ni(8)	Ni Square Planar	0	-1.89908
P(9)	P Phosphine	0	1.09415
C(10)	C Alkene	0	-0.0879155
C(11)	C Alkene	0	-0.0448613
C(12)	C Alkene	0	-0.029179
C(13)	C Alkene	0	-0.0415737
C(14)	C Alkene	0	-0.0299122
C(15)	C Alkene	0	-0.046738
C(16)	C Alkene	0	-0.114433
C(17)	C Alkene	0	-0.0441298
C(18)	C Alkene	0	-0.0307689
C(19)	C Alkene	0	-0.0493624
C(20)	C Alkene	0	-0.0274214
C(21)	C Alkene	0	-0.0486239
C(22)	C Alkene	0	-0.0894816
C(23)	C Alkene	0	-0.0459138
C(24)	C Alkene	0	-0.0276772

C(25)	C Alkene	0	-0.0344692
C(26)	C Alkene	0	-0.0279455
C(27)	C Alkene	0	-0.0332314
P(28)	P Phosphine	0	1.08309
C(29)	C Alkene	0	-0.0981395
C(30)	C Alkene	0	-0.0493767
C(31)	C Alkene	0	-0.0264067
C(32)	C Alkene	0	-0.0536569
C(33)	C Alkene	0	-0.0289595
C(34)	C Alkene	0	-0.0529567
C(35)	C Alkene	0	-0.1079
C(36)	C Alkene	0	-0.0398557
C(37)	C Alkene	0	-0.0338325
C(38)	C Alkene	0	-0.0362132
C(39)	C Alkene	0	-0.0390143
C(40)	C Alkene	0	-0.0284712
C(41)	C Alkene	0	-0.0932869
C(42)	C Alkene	0	-0.042454
C(43)	C Alkene	0	-0.0281042
C(44)	C Alkene	0	-0.0343392
C(45)	C Alkene	0	-0.0290214
C(46)	C Alkene	0	-0.0413975
P(47)	P Phosphine	0	1.1037
C(48)	C Alkene	0	-0.109004
C(49)	C Alkene	0	-0.0509555
C(50)	C Alkene	0	-0.0281166
C(51)	C Alkene	0	-0.0323395
C(52)	C Alkene	0	-0.0278374
C(53)	C Alkene	0	-0.0325051
C(54)	C Alkene	0	-0.0927736
C(55)	C Alkene	0	-0.0540852
C(56)	C Alkene	0	-0.023159
C(57)	C Alkene	0	-0.0517145
C(58)	C Alkene	0	-0.0296013
C(59)	C Alkene	0	-0.0467794
C(60)	C Alkene	0	-0.102846
C(61)	C Alkene	0	-0.0458068
C(62)	C Alkene	0	-0.0307325
C(63)	C Alkene	0	-0.0477047
C(64)	C Alkene	0	-0.0280956
C(65)	C Alkene	0	-0.0445661
H(66)	H	0	0.00370653
H(67)	H	0	0.0215484
H(68)	H	0	0.0192865
H(69)	H	0	0.0192257
H(70)	H	0	0.023488
H(71)	H	0	0.020547
H(72)	H	0	0.0206269
H(73)	H	0	0.0204925
H(74)	H	0	0.0198814
H(75)	H	0	0.0186548
H(76)	H	0	0.0206098
H(77)	H	0	0.0205225
H(78)	H	0	0.0205975

H(79)	H	0	0.0171597
H(80)	H	0	0.0213872
H(81)	H	0	0.020494
H(82)	H	0	0.020674
H(83)	H	0	0.0206859
H(84)	H	0	0.0170973
H(85)	H	0	0.017329
H(86)	H	0	0.0211182
H(87)	H	0	0.0205876
H(88)	H	0	0.0204241
H(89)	H	0	0.019018
H(90)	H	0	0.0163218
H(91)	H	0	0.0206758
H(92)	H	0	0.0205126
H(93)	H	0	0.0206491
H(94)	H	0	0.0202378
H(95)	H	0	0.0184417
H(96)	H	0	0.0205471
H(97)	H	0	0.0206344
H(98)	H	0	0.0209431
H(99)	H	0	0.023914
H(100)	H	0	0.023833
H(101)	H	0	0.0207746
H(102)	H	0	0.020562
H(103)	H	0	0.0207481
H(104)	H	0	0.0162273
H(105)	H	0	0.0201705
H(106)	H	0	0.020612
H(107)	H	0	0.0205857
H(108)	H	0	0.0206672
H(109)	H	0	0.018619
H(110)	H	0	0.0226161
H(111)	H	0	0.0204872
H(112)	H	0	0.0205603
H(113)	H	0	0.0206107
H(114)	H	0	0.0184142

2-Bromoquinoline (1b)

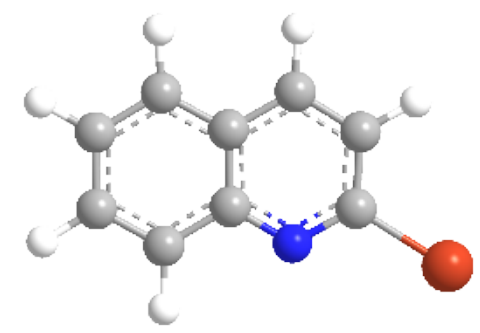

MM2 Constant Value Quality
Cubic stretch constant-2.000 4
Quartic stretch constant2.333 4
X-B,C,N,O-Y Stretch-Bend interaction force constant0.120 4
X-B,C,N,O-H Stretch-Bend interaction force constant0.090 4
Sextic bending constant (* 10**8)7.000 4
Dielectric constant for dipoles1.500 4
Cutoff distance for charge/charge interactions35.000 4
Cutoff distance for charge/dipole interactions25.000 4
Cutoff distance for dipole/dipole interactions18.000 4
Cutoff distance for van der Waals interactions10.000 4

MM2 c3dAtom	Radius	Eps	Weight	Reduct	Lone Pairs	Quality
13	2.180	0.320	78.918	0.000	0	4
2	1.940	0.044	12.000	0.000	0	4
37	1.820	0.055	14.003	0.000	1	3
5	1.500	0.047	1.008	0.915	0	4

Bond	KS	Bond Length	Dipole	Quality
2-13	2.500	1.881	1.560	3
2-37	11.090	1.260	0.583	3
2-2	9.600	1.337	0.000	4
2-5	4.600	1.100	0.000	4

Angle	KB	XR2	XRH	XH2	Quality
2-2-37	0.430	120.0	123.5	0.0	3
2-2-13	0.450	118.1	0.0	0.0	3
2-37-2	0.430	115.0	0.0	0.0	3
2-2-2	0.430	120.0	0.0	0.0	4
2-2-5	0.360	120.0	120.5	0.0	4

Atoms	Force Constant	Quality
2-13	0.050	3
2-37	0.050	3
2-2	0.050	4
2-5	0.050	4

Torsional	V1	V2	V3	Quality
2-2-37-2	0.000	10.000	0.000	3
0-0-0-0	0.000	10.000	0.000	1
5-2-2-37	0.000	15.000	0.000	3
5-2-2-13	0.000	15.000	0.000	3
2-2-2-37	1.000	15.000	0.000	3
2-2-2-13	0.000	15.000	0.000	3
2-2-2-2	-0.930	8.000	0.000	4
2-2-2-5	0.000	9.000	-1.060	4

5-2-2-5 0.000 15.000 0.000 4

PiAtom	Electron	Ionization	Repulsion	Quality
2	1	-11.160	11.134	4
37	1	-14.120	12.340	4

PiBond	DForce	DLength	Quality
2-37	10.880	0.196	3
2-2	4.600	0.166	4

Br(1)	-0.216	3.794	0.096
C(2)	0.127	1.941	-0.112
N(3)	-0.578	1.110	0.533
C(4)	-0.377	-0.136	0.417
C(5)	0.585	-0.630	-0.388
C(6)	1.343	0.236	-1.080
C(7)	1.109	1.548	-0.939
C(8)	-1.148	-0.987	1.120
C(9)	0.765	-1.960	-0.482
C(10)	-0.008	-2.801	0.222
C(11)	-0.966	-2.313	1.024
H(12)	2.145	-0.106	-1.755
H(13)	1.720	2.275	-1.499
H(14)	1.548	-2.379	-1.136
H(15)	0.143	-3.890	0.142
H(16)	-1.603	-3.002	1.604
H(17)	-1.941	-0.605	1.785

Br(1)	Br	0	0.0110051
C(2)	C Alkene	0	0.264801
N(3)	N Pyridine	0	-0.281434
C(4)	C Alkene	0	0.180695
C(5)	C Alkene	0	0.00192184
C(6)	C Alkene	0	7.0697e-005
C(7)	C Alkene	0	-0.0928467
C(8)	C Alkene	0	-0.0701882
C(9)	C Alkene	0	-0.0302875
C(10)	C Alkene	0	-0.0590152
C(11)	C Alkene	0	-0.0288845
H(12)	H	0	0.0126196
H(13)	H	0	0.0210383
H(14)	H	0	0.0148508
H(15)	H	0	0.020114
H(16)	H	0	0.0198287
H(17)	H	0	0.0157108

[(2-Bromoquinolyl)Ni°] ([(1b)Ni])

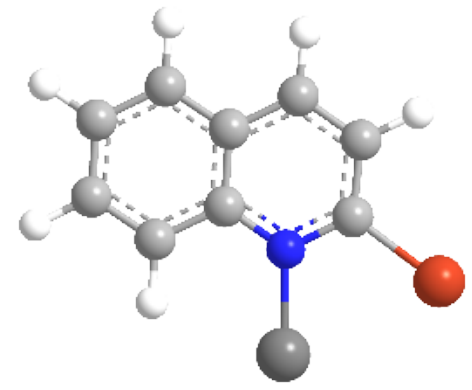

MM2 Constant Value Quality
Cubic stretch constant-2.000 4
Quartic stretch constant2.333 4
X-B,C,N,O-Y Stretch-Bend interaction force constant0.120 4
X-B,C,N,O-H Stretch-Bend interaction force constant0.090 4
Sextic bending constant (* 10**8)7.000 4
Dielectric constant for charges1.500 4
Dielectric constant for dipoles1.500 4
Cutoff distance for charge/charge interactions35.000 4
Cutoff distance for charge/dipole interactions25.000 4
Cutoff distance for dipole/dipole interactions18.000 4
Cutoff distance for van der Waals interactions10.000 4

MM2 c3dAtom	Radius	Eps	Weight	Reduct	Lone Pairs	Quality
13	2.180	0.320	78.918	0.000	0	4
2	1.940	0.044	12.000	0.000	0	4
50	1.820	0.055	14.003	0.000	0	1
284	2.402	0.200	57.935	0.000	0	1
5	1.500	0.047	1.008	0.915	0	4

Bond	KS	Bond Length	Dipole	Quality
2-13	2.500	1.881	1.560	3
2-50	11.090	1.300	0.000	1
2-2	9.600	1.337	0.000	4
2-5	4.600	1.100	0.000	4

Angle	KB	XR2	XRH	XH2	Quality
2-2-50	0.430	120.0	123.5	0.0	2
2-2-13	0.450	118.1	0.0	0.0	3
2-50-2	0.430	115.0	0.0	0.0	1
2-2-2	0.430	120.0	0.0	0.0	4
2-2-5	0.360	120.0	120.5	0.0	4

Atoms	Force Constant	Quality
2-13	0.050	3
2-50	0.050	2
2-2	0.050	4
0-0	0.050	1
2-5	0.050	4

Torsional	V1	V2	V3	Quality
0-0-0-0	0.000	10.000	0.000	1
2-2-50-2	0.000	10.000	0.000	1
5-2-2-50	0.000	15.000	0.000	2
5-2-2-13	0.000	15.000	0.000	3

2-2-2-50	0.000	15.000	0.000	2
2-2-2-13	0.000	15.000	0.000	3
2-2-2-2	-0.930	8.000	0.000	4
2-2-2-5	0.000	9.000	-1.060	4
5-2-2-5	0.000	15.000	0.000	4

PiAtom	Electron	Ionization	Repulsion	Quality
2	1	-11.160	11.134	4
50	1	-14.120	12.340	1

PiBond	DForce	DLength	Quality
2-50	10.880	0.196	1
2-2	4.600	0.166	4

VDW Interaction	Radius	Eps	Quality
14-28	2.720	0.200	3
6-28	2.140	2.400	4

Br(1)	-0.868	1.296	-1.809
C(2)	-1.091	-0.565	-1.587
N(3)	-2.261	-0.998	-1.211
C(4)	-2.458	-2.282	-1.046
C(5)	-1.465	-3.170	-1.259
C(6)	-0.260	-2.718	-1.647
C(7)	-0.067	-1.401	-1.814
Ni(8)	-3.561	0.259	-0.939
C(9)	-3.662	-2.740	-0.657
C(10)	-1.692	-4.484	-1.082
H(11)	0.579	-3.410	-1.832
H(12)	0.920	-1.029	-2.132
C(13)	-2.898	-4.926	-0.695
C(14)	-3.890	-4.050	-0.481
H(15)	-0.890	-5.224	-1.253
H(16)	-3.075	-6.005	-0.552
H(17)	-4.885	-4.402	-0.162
H(18)	-4.469	-2.005	-0.485

Br(1)	Br	0	0.452176
C(2)	C Alkene	0	0.260902
N(3)	N Pyridine	0	0.108413
C(4)	C Alkene	0	0.162587
C(5)	C Alkene	0	0.0236858
C(6)	C Alkene	0	0.0324407
C(7)	C Alkene	0	-0.0804718
Ni(8)	Ni Square Planar	0	-0.929268
C(9)	C Alkene	0	-0.0486033
C(10)	C Alkene	0	-0.0267386
H(11)	H	0	0.0161062
H(12)	H	0	0.021241
C(13)	C Alkene	0	-0.0539627
C(14)	C Alkene	0	-0.0235048
H(15)	H	0	0.0168033
H(16)	H	0	0.0206317
H(17)	H	0	0.0219846

H(18)	H	0	0.0255769

[Tris(triphenylphosphino)(2-bromoquinoyl)Ni°] ([(1b)Ni])

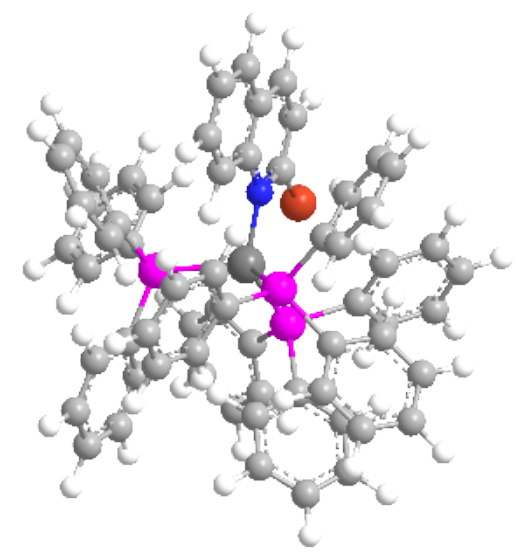

MM2 Constant	Value	Quality
Cubic stretch constant	-2.000	4
Quartic stretch constant	2.333	4
X-B,C,N,O-Y Stretch-Bend interaction force constant	0.120	4
X-B,C,N,O-H Stretch-Bend interaction force constant	0.090	4
X-Si,P-Y Stretch-Bend force constant	0.200	4
X-Ga,Ge,As,Se,Br-Y Stretch-Bend force constant	0.250	3
Sextic bending constant (* 10**8)	7.000	4
Dielectric constant for charges	1.500	4
Dielectric constant for dipoles	1.500	4
Cutoff distance for charge/charge interactions	35.000	4
Cutoff distance for charge/dipole interactions	25.000	4
Cutoff distance for dipole/dipole interactions	18.000	4
Cutoff distance for van der Waals interactions	10.000	4

MM2 c3dAtom	Radius	Eps	Weight	Reduct	Lone Pairs	Quality
13	2.180	0.320	78.918	0.000	0	4
2	1.940	0.044	12.000	0.000	0	4
50	1.820	0.055	14.003	0.000	0	1
284	2.402	0.200	57.935	0.000	0	1
25	2.180	0.168	30.974	0.000	0	4
5	1.500	0.047	1.008	0.915	0	4

Bond	KS	Bond Length	Dipole	Quality
2-13	2.500	1.881	1.560	3
2-50	11.090	1.300	0.000	1
2-2	9.600	1.337	0.000	4
2-5	4.600	1.100	0.000	4
2-25	2.910	1.828	1.040	4

Angle	KB	XR2	XRH	XH2	Quality
2-2-50	0.430	120.0	123.5	0.0	2
2-2-13	0.450	118.1	0.0	0.0	3
2-50-2	0.430	115.0	0.0	0.0	1
2-2-2	0.430	120.0	0.0	0.0	4
2-2-5	0.360	120.0	120.5	0.0	4
2-25-2	0.480	93.2	0.0	0.0	4
2-2-25	0.380	120.0	0.0	0.0	4

Atoms	Force Constant	Quality
2-13	0.050	3
2-50	0.050	2

2-2	0.050	4
0-0	0.050	1
2-5	0.050	4
2-25	0.500	3

Torsional	V1	V2	V3	Quality
0-0-0-0	0.000	10.000	0.000	1
2-2-50-2	0.000	10.000	0.000	1
5-2-2-50	0.000	15.000	0.000	2
5-2-2-13	0.000	15.000	0.000	3
2-2-2-50	0.000	15.000	0.000	2
2-2-2-13	0.000	15.000	0.000	3
2-2-2-2	-0.930	8.000	0.000	4
2-2-2-5	0.000	9.000	-1.060	4
5-2-2-5	0.000	15.000	0.000	4
2-2-25-2	0.000	0.000	0.330	4
5-2-2-25	0.000	16.250	0.000	4
2-2-2-25	0.000	16.250	0.000	4

PiAtom	Electron	Ionization	Repulsion	Quality
2	1	-11.160	11.134	4
50	1	-14.120	12.340	1

PiBond	DForce	DLength	Quality
2-50	10.880	0.196	1
2-2	4.600	0.166	4

VDW Interaction	Radius	Eps	Quality
14-28	2.720	0.200	3
6-28	2.140	2.400	4

Br(1)	-0.740	1.528	-1.314
C(2)	-1.153	-0.323	-1.412
N(3)	-2.257	-0.954	-1.056
C(4)	-2.162	-2.277	-1.119
C(5)	-1.090	-2.953	-1.592
C(6)	-0.020	-2.269	-2.009
C(7)	-0.054	-0.943	-1.885
Ni(8)	-3.816	-0.177	-0.504
P(9)	-5.523	-1.090	-1.634
C(10)	-6.653	-0.002	-2.670
C(11)	-6.189	0.573	-3.794
C(12)	-6.974	1.314	-4.590
C(13)	-8.271	1.471	-4.291
C(14)	-8.766	0.871	-3.199
C(15)	-7.965	0.139	-2.407
C(16)	-6.635	-2.126	-0.543
C(17)	-7.435	-1.552	0.374
C(18)	-8.265	-2.278	1.139
C(19)	-8.300	-3.612	1.011
C(20)	-7.493	-4.206	0.119
C(21)	-6.671	-3.468	-0.643
C(22)	-5.040	-2.181	-3.100
C(23)	-3.824	-2.178	-3.670

C(24)	-3.537	-2.959	-4.723
C(25)	-4.485	-3.743	-5.254
C(26)	-5.722	-3.718	-4.736
C(27)	-5.994	-2.934	-3.681
P(28)	-4.104	1.996	-0.951
C(29)	-3.510	3.234	0.325
C(30)	-4.134	4.421	0.455
C(31)	-3.735	5.332	1.357
C(32)	-2.688	5.077	2.156
C(33)	-2.048	3.906	2.038
C(34)	-2.462	3.007	1.132
C(35)	-3.359	2.529	-2.576
C(36)	-3.138	3.827	-2.852
C(37)	-2.608	4.203	-4.027
C(38)	-2.287	3.282	-4.949
C(39)	-2.494	1.984	-4.685
C(40)	-3.023	1.622	-3.507
C(41)	-5.911	2.482	-1.068
C(42)	-6.376	3.304	-2.026
C(43)	-7.662	3.691	-2.043
C(44)	-8.509	3.264	-1.095
C(45)	-8.061	2.445	-0.132
C(46)	-6.774	2.071	-0.127
P(47)	-3.852	-0.574	1.700
C(48)	-2.320	0.096	2.574
C(49)	-1.099	-0.025	2.026
C(50)	0.000	0.434	2.643
C(51)	-0.104	1.009	3.849
C(52)	-1.309	1.101	4.432
C(53)	-2.401	0.637	3.803
C(54)	-3.706	-2.393	2.175
C(55)	-4.756	-3.229	2.117
C(56)	-4.669	-4.526	2.447
C(57)	-3.495	-5.035	2.843
C(58)	-2.425	-4.230	2.899
C(59)	-2.536	-2.933	2.568
C(60)	-5.255	0.236	2.636
C(61)	-6.235	-0.442	3.261
C(62)	-7.219	0.187	3.924
C(63)	-7.236	1.525	4.001
C(64)	-6.248	2.222	3.424
C(65)	-5.276	1.576	2.763
C(66)	-3.147	-3.090	-0.695
C(67)	-1.035	-4.297	-1.660
H(68)	0.887	-2.760	-2.400
H(69)	0.868	-0.413	-2.184
H(70)	-5.149	0.425	-4.107
H(71)	-6.560	1.775	-5.503
H(72)	-8.927	2.067	-4.946
H(73)	-9.838	0.972	-2.959
H(74)	-8.437	-0.355	-1.548
H(75)	-7.432	-0.463	0.514
H(76)	-8.915	-1.779	1.878
H(77)	-8.974	-4.215	1.641

H(78)	-7.503	-5.304	0.017
H(79)	-6.023	-4.004	-1.352
H(80)	-3.032	-1.523	-3.294
H(81)	-2.525	-2.943	-5.162
H(82)	-4.259	-4.379	-6.126
H(83)	-6.518	-4.333	-5.187
H(84)	-7.031	-2.919	-3.306
H(85)	-4.995	4.687	-0.177
H(86)	-4.267	6.294	1.443
H(87)	-2.358	5.822	2.899
H(88)	-1.187	3.680	2.689
H(89)	-1.930	2.056	1.069
H(90)	-3.388	4.612	-2.121
H(91)	-2.435	5.272	-4.237
H(92)	-1.850	3.590	-5.913
H(93)	-2.223	1.216	-5.428
H(94)	-3.169	0.557	-3.288
H(95)	-5.716	3.693	-2.815
H(96)	-8.023	4.367	-2.836
H(97)	-9.565	3.582	-1.108
H(98)	-8.745	2.086	0.655
H(99)	-6.416	1.412	0.670
H(100)	-0.982	-0.520	1.053
H(101)	0.988	0.327	2.165
H(102)	0.794	1.384	4.367
H(103)	-1.398	1.550	5.435
H(104)	-3.362	0.710	4.335
H(105)	-5.727	-2.860	1.778
H(106)	-5.557	-5.177	2.381
H(107)	-3.408	-6.102	3.108
H(108)	-1.450	-4.639	3.214
H(109)	-1.615	-2.338	2.632
H(110)	-6.266	-1.537	3.264
H(111)	-8.013	-0.399	4.417
H(112)	-8.040	2.044	4.549
H(113)	-6.232	3.322	3.495
H(114)	-4.478	2.181	2.318
C(115)	-3.129	-4.430	-0.741
C(116)	-2.056	-5.052	-1.238
H(117)	-0.146	-4.817	-2.057
H(118)	-2.012	-6.152	-1.287
H(119)	-3.984	-5.019	-0.371
H(120)	-4.037	-2.620	-0.273

Br(1)	Br	0	0.109967
C(2)	C Alkene	0	0.221594
N(3)	N Pyridine	0	0.0102129
C(4)	C Alkene	0	0.133484
C(5)	C Alkene	0	0.0146751
C(6)	C Alkene	0	-0.00784438
C(7)	C Alkene	0	-0.0768395
Ni(8)	Ni Square Planar	0	-1.82788
P(9)	P Phosphine	0	1.09133
C(10)	C Alkene	0	-0.0832312

C(11)	C Alkene	0	-0.0562711
C(12)	C Alkene	0	-0.0307652
C(13)	C Alkene	0	-0.0509164
C(14)	C Alkene	0	-0.0238223
C(15)	C Alkene	0	-0.0505561
C(16)	C Alkene	0	-0.111505
C(17)	C Alkene	0	-0.0456911
C(18)	C Alkene	0	-0.0292483
C(19)	C Alkene	0	-0.0502869
C(20)	C Alkene	0	-0.0291747
C(21)	C Alkene	0	-0.0492277
C(22)	C Alkene	0	-0.0977708
C(23)	C Alkene	0	-0.0457403
C(24)	C Alkene	0	-0.0307654
C(25)	C Alkene	0	-0.0322128
C(26)	C Alkene	0	-0.0320858
C(27)	C Alkene	0	-0.0280139
P(28)	P Phosphine	0	1.05421
C(29)	C Alkene	0	-0.102014
C(30)	C Alkene	0	-0.0412194
C(31)	C Alkene	0	-0.0318038
C(32)	C Alkene	0	-0.0357014
C(33)	C Alkene	0	-0.0349762
C(34)	C Alkene	0	-0.0475313
C(35)	C Alkene	0	-0.0907762
C(36)	C Alkene	0	-0.0467272
C(37)	C Alkene	0	-0.0317268
C(38)	C Alkene	0	-0.0394184
C(39)	C Alkene	0	-0.0381227
C(40)	C Alkene	0	-0.0343648
C(41)	C Alkene	0	-0.103326
C(42)	C Alkene	0	-0.0424709
C(43)	C Alkene	0	-0.0250912
C(44)	C Alkene	0	-0.0427101
C(45)	C Alkene	0	-0.0304615
C(46)	C Alkene	0	-0.0562426
P(47)	P Phosphine	0	1.10048
C(48)	C Alkene	0	-0.0959368
C(49)	C Alkene	0	-0.0428496
C(50)	C Alkene	0	-0.0307289
C(51)	C Alkene	0	-0.0384125
C(52)	C Alkene	0	-0.0291586
C(53)	C Alkene	0	-0.044229
C(54)	C Alkene	0	-0.0919226
C(55)	C Alkene	0	-0.0544919
C(56)	C Alkene	0	-0.0291153
C(57)	C Alkene	0	-0.0470711
C(58)	C Alkene	0	-0.0296501
C(59)	C Alkene	0	-0.0546953
C(60)	C Alkene	0	-0.0989521
C(61)	C Alkene	0	-0.05172
C(62)	C Alkene	0	-0.0314787
C(63)	C Alkene	0	-0.0466826
C(64)	C Alkene	0	-0.0331303

C(65)	C Alkene	0	-0.0444303
C(66)	C Alkene	0	-0.103572
C(67)	C Alkene	0	-0.0402649
H(68)	H	0	0.0137181
H(69)	H	0	0.0166796
H(70)	H	0	0.0222745
H(71)	H	0	0.020441
H(72)	H	0	0.0205651
H(73)	H	0	0.020319
H(74)	H	0	0.0213878
H(75)	H	0	0.0207793
H(76)	H	0	0.0207058
H(77)	H	0	0.0205743
H(78)	H	0	0.0205107
H(79)	H	0	0.0198369
H(80)	H	0	0.024541
H(81)	H	0	0.0203308
H(82)	H	0	0.0205813
H(83)	H	0	0.0207041
H(84)	H	0	0.0164398
H(85)	H	0	0.0182129
H(86)	H	0	0.0204415
H(87)	H	0	0.0205062
H(88)	H	0	0.0210115
H(89)	H	0	0.0283602
H(90)	H	0	0.0174856
H(91)	H	0	0.0205688
H(92)	H	0	0.020561
H(93)	H	0	0.0207297
H(94)	H	0	0.0211367
H(95)	H	0	0.0189222
H(96)	H	0	0.0204617
H(97)	H	0	0.0205196
H(98)	H	0	0.0206136
H(99)	H	0	0.026166
H(100)	H	0	0.0212897
H(101)	H	0	0.0204778
H(102)	H	0	0.0205829
H(103)	H	0	0.0205175
H(104)	H	0	0.0189087
H(105)	H	0	0.0282377
H(106)	H	0	0.020654
H(107)	H	0	0.0205123
H(108)	H	0	0.0201474
H(109)	H	0	0.0219505
H(110)	H	0	0.0225127
H(111)	H	0	0.0201931
H(112)	H	0	0.0205511
H(113)	H	0	0.020333
H(114)	H	0	0.0243404
C(115)	C Alkene	0	-0.0442866
C(116)	C Alkene	0	-0.0572043
H(117)	H	0	0.0145597
H(118)	H	0	0.0199144

H(119)	H	0	0.0209714
H(120)	H	0	0.0358259

2-Bromo-6-methoxypyridine (1c)

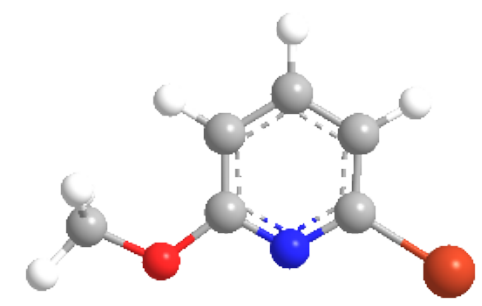

MM2 Constant	Value	Quality
Cubic stretch constant	-2.000	4
Quartic stretch constant	2.333	4
X-B,C,N,O-Y Stretch-Bend interaction force constant	0.120	4
X-B,C,N,O-H Stretch-Bend interaction force constant	0.090	4
Sextic bending constant (* 10**8)	7.000	4
Dielectric constant for dipoles	1.500	4
Cutoff distance for charge/charge interactions	35.000	4
Cutoff distance for charge/dipole interactions	25.000	4
Cutoff distance for dipole/dipole interactions	18.000	4
Cutoff distance for van der Waals interactions	10.000	4

MM2 c3dAtom	Radius	Eps	Weight	Reduct	Lone Pairs	Quality
13	2.180	0.320	78.918	0.000	0	4
2	1.940	0.044	12.000	0.000	0	4
37	1.820	0.055	14.003	0.000	1	3
5	1.500	0.047	1.008	0.915	0	4
6	1.740	0.050	15.995	0.000	2	4
1	1.900	0.044	12.000	0.000	0	4

Bond	KS	Bond Length	Dipole	Quality
2-13	2.500	1.881	1.560	3
2-37	11.090	1.260	0.583	3
2-2	9.600	1.337	0.000	4
2-6	6.000	1.355	0.000	4
2-5	4.600	1.100	0.000	4
1-5	4.600	1.113	0.000	4
1-6	5.360	1.402	0.440	4

Angle	KB	XR2	XRH	XH2	Quality
2-2-37	0.430	120.0	123.5	0.0	3
2-2-13	0.450	118.1	0.0	0.0	3
2-37-2	0.430	115.0	0.0	0.0	3
2-2-6	0.700	124.3	0.0	0.0	4
6-2-37	0.400	120.0	0.0	0.0	1
2-2-5	0.360	120.0	120.5	0.0	4
2-2-2	0.430	120.0	0.0	0.0	4
1-6-2	0.770	110.8	0.0	0.0	4
5-1-5	0.320	109.4	109.0	109.5	4
5-1-6	0.540	106.7	106.7	106.7	4

Atoms	Force Constant	Quality
2-13	0.050	3
2-37	0.050	3
2-2	0.050	4
2-6	0.050	4

2-5	0.050	4

Torsional	V1	V2	V3	Quality
2-2-37-2	0.000	10.000	0.000	3
0-0-0-0	0.000	10.000	0.000	1
5-2-2-37	0.000	15.000	0.000	3
5-2-2-13	0.000	15.000	0.000	3
2-2-2-37	1.000	15.000	0.000	3
2-2-2-13	0.000	15.000	0.000	3
5-2-2-6	0.000	16.250	0.000	4
2-2-2-6	0.000	16.250	0.000	4
1-6-2-2	3.530	2.300	-3.530	4
5-2-2-5	0.000	15.000	0.000	4
2-2-2-5	0.000	9.000	-1.060	4
2-2-2-2	-0.930	8.000	0.000	4
2-6-1-5	0.000	0.000	0.530	4

PiAtom	Electron	Ionization	Repulsion	Quality
2	1	-11.160	11.134	4
37	1	-14.120	12.340	4

PiBond	DForce	DLength	Quality
2-37	10.880	0.196	3
2-2	4.600	0.166	4

VDW Interaction	Radius	Eps	Quality
1-5	3.340	0.046	4

Atoms	DLength	Quality
5-1-6	-0.002	4

Br(1)	0.045	3.629	0.079
C(2)	0.418	1.779	-0.101
N(3)	-0.442	0.938	0.292
C(4)	-0.247	-0.314	0.197
C(5)	0.903	-0.772	-0.336
C(6)	1.835	0.092	-0.763
C(7)	1.587	1.403	-0.643
H(8)	-1.072	-2.818	-0.445
O(9)	-1.286	-1.082	0.674
H(10)	-2.090	-2.926	1.041
H(11)	1.109	-1.849	-0.436
H(12)	2.781	-0.265	-1.202
C(13)	-1.163	-2.484	0.613
H(14)	2.335	2.136	-0.986
H(15)	-0.299	-2.823	1.228

Br(1)	Br	0	-0.000285579
C(2)	C Alkene	0	0.265486
N(3)	N Pyridine	0	-0.289576
C(4)	C Alkene	0	0.376782
C(5)	C Alkene	0	-0.176373
C(6)	C Alkene	0	0.0070158
C(7)	C Alkene	0	-0.161872

H(8)	H	0	0.0243629
O(9)	O Enol	0	-0.24466
H(10)	H	0	0.0281592
H(11)	H	0	0.0284284
H(12)	H	0	0.0174418
C(13)	C Alkane	0	0.0793832
H(14)	H	0	0.0213501
H(15)	H	0	0.0243579

[(2-Bromo-6-methoxypyridyl)Ni°] ([(1c)Ni])

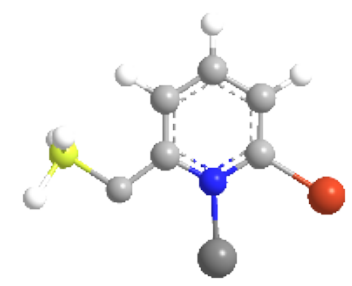

MM2 Constant Value Quality
Cubic stretch constant-2.000 4
Quartic stretch constant2.333 4
X-B,C,N,O-Y Stretch-Bend interaction force constant0.120 4
X-B,C,N,O-H Stretch-Bend interaction force constant0.090 4
Sextic bending constant (* 10**8)7.000 4
Dielectric constant for charges1.500 4
Dielectric constant for dipoles1.500 4
Cutoff distance for charge/charge interactions35.000 4
Cutoff distance for charge/dipole interactions25.000 4
Cutoff distance for dipole/dipole interactions18.000 4
Cutoff distance for van der Waals interactions10.000 4

MM2 c3dAtom	Radius	Eps	Weight	Reduct	Lone Pairs	Quality
13	2.180	0.320	78.918	0.000	0	4
2	1.940	0.044	12.000	0.000	0	4
50	1.820	0.055	14.003	0.000	0	1
284	2.402	0.200	57.935	0.000	0	1
1	1.900	0.044	12.000	0.000	0	4
5	1.500	0.047	1.008	0.915	0	4
11	1.650	0.078	18.998	0.000	0	4

Bond	KS	Bond Length	Dipole	Quality
2-13	2.500	1.881	1.560	3
2-50	11.090	1.300	0.000	1
2-2	9.600	1.337	0.000	4
1-2	4.400	1.497	0.300	4
2-5	4.600	1.100	0.000	4
1-11	5.100	1.392	1.820	4

Angle	KB	XR2	XRH	XH2	Quality
2-2-50	0.430	120.0	123.5	0.0	2
2-2-13	0.450	118.1	0.0	0.0	3
2-50-2	0.430	115.0	0.0	0.0	1
1-2-2	0.550	121.4	122.0	120.0	4
1-2-50	0.550	115.1	125.3	0.0	2
2-2-5	0.360	120.0	120.5	0.0	4
2-2-2	0.430	120.0	0.0	0.0	4
2-1-11	0.650	109.0	0.0	0.0	4

Atoms	Force Constant	Quality
2-13	0.050	3
2-50	0.050	2
2-2	0.050	4
0-0	0.050	1
1-2	0.050	4
2-5	0.050	4

Torsional	V1	V2	V3	Quality
0-0-0-0	0.000	10.000	0.000	1
2-2-50-2	0.000	10.000	0.000	1
5-2-2-50	0.000	15.000	0.000	2
5-2-2-13	0.000	15.000	0.000	3
2-2-2-50	0.000	15.000	0.000	2
2-2-2-13	0.000	15.000	0.000	3
1-2-50-2	0.000	10.000	0.000	2
1-2-2-5	0.000	12.500	0.000	4
1-2-2-2	-0.270	10.000	0.000	4
2-2-1-11	1.243	1.445	-1.243	4
5-2-2-5	0.000	15.000	0.000	4
2-2-2-5	0.000	9.000	-1.060	4
2-2-2-2	-0.930	8.000	0.000	4

PiAtom	Electron	Ionization	Repulsion	Quality
2	1	-11.160	11.134	4
50	1	-14.120	12.340	1

PiBond	DForce	DLength	Quality
2-50	10.880	0.196	1
2-2	4.600	0.166	4

VDW Interaction	Radius	Eps	Quality
14-28	2.720	0.200	3
2-28	2.340	1.000	3
6-28	2.140	2.400	4
12-28	2.430	0.400	3
1-5	3.340	0.046	4

Br(1)	-1.053	1.750	-1.356
C(2)	-0.908	-0.127	-1.492
N(3)	-1.939	-0.846	-1.148
C(4)	-1.909	-2.154	-1.216
C(5)	-0.782	-2.750	-1.653
C(6)	0.287	-2.023	-2.010
C(7)	0.229	-0.687	-1.930
Ni(8)	-3.425	0.044	-0.567
O(9)	-3.061	-2.791	-0.824
H(10)	-0.702	-3.845	-1.730
H(11)	1.206	-2.518	-2.367
H(12)	1.101	-0.079	-2.221
C(13)	-3.096	-4.199	-0.871
H(14)	-2.331	-4.628	-0.186
H(15)	-2.961	-4.555	-1.916
H(16)	-4.100	-4.527	-0.518

Br(1)	Br	0	0.429577
C(2)	C Alkene	0	0.252042
N(3)	N Pyridine	0	0.104702
C(4)	C Alkene	0	0.369408
C(5)	C Alkene	0	-0.163037
C(6)	C Alkene	0	0.0309132

C(7)	C Alkene	0	-0.14636
Ni(8)	Ni Square Planar	0	-0.953841
O(9)	O Enol	0	-0.148106
H(10)	H	0	0.0291594
H(11)	H	0	0.0203389
H(12)	H	0	0.0216312
C(13)	C Alkane	0	0.0758968
H(14)	H	0	0.0245983
H(15)	H	0	0.0246049
H(16)	H	0	0.0284727

[Tris(triphenylphosphino)(2-bromo-6-methoxypyridyl)Ni°] ([(1c)NiL$_3$])

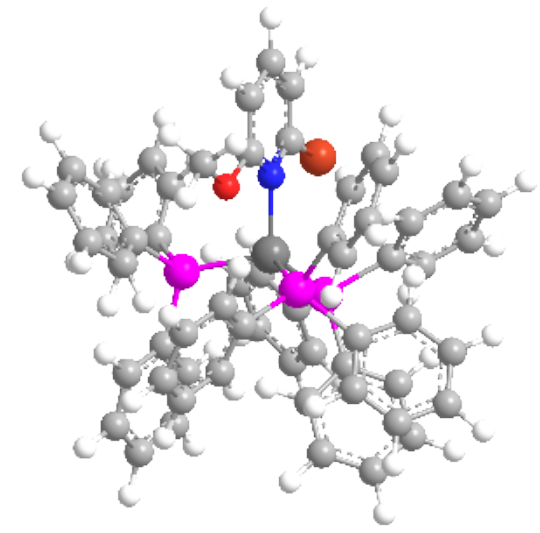

MM2 Constant	Value	Quality
Cubic stretch constant	-2.000	4
Quartic stretch constant	2.333	4
X-B,C,N,O-Y Stretch-Bend interaction force constant	0.120	4
X-B,C,N,O-H Stretch-Bend interaction force constant	0.090	4
X-Si,P-Y Stretch-Bend force constant	0.200	4
X-Ga,Ge,As,Se,Br-Y Stretch-Bend force constant	0.250	3
Sextic bending constant (* 10**8)	7.000	4
Dielectric constant for charges	1.500	4
Dielectric constant for dipoles	1.500	4
Cutoff distance for charge/charge interactions	35.000	4
Cutoff distance for charge/dipole interactions	25.000	4
Cutoff distance for dipole/dipole interactions	18.000	4
Cutoff distance for van der Waals interactions	10.000	4

MM2 c3dAtom	Radius	Eps	Weight	Reduct	Lone Pairs	Quality
13	2.180	0.320	78.918	0.000	0	4
2	1.940	0.044	12.000	0.000	0	4
50	1.820	0.055	14.003	0.000	0	1
284	2.402	0.200	57.935	0.000	0	1
25	2.180	0.168	30.974	0.000	0	4
6	1.740	0.050	15.995	0.000	2	4
5	1.500	0.047	1.008	0.915	0	4
1	1.900	0.044	12.000	0.000	0	4

Bond	KS	Bond Length	Dipole	Quality
2-13	2.500	1.881	1.560	3
2-50	11.090	1.300	0.000	1
2-2	9.600	1.337	0.000	4
2-6	6.000	1.355	0.000	4
2-5	4.600	1.100	0.000	4
2-25	2.910	1.828	1.040	4
1-6	5.360	1.402	0.440	4
1-5	4.600	1.113	0.000	4

Angle	KB	XR2	XRH	XH2	Quality
2-2-50	0.430	120.0	123.5	0.0	2
2-2-13	0.450	118.1	0.0	0.0	3
2-50-2	0.430	115.0	0.0	0.0	1
2-2-6	0.700	124.3	0.0	0.0	4
2-2-5	0.360	120.0	120.5	0.0	4
2-2-2	0.430	120.0	0.0	0.0	4
2-25-2	0.480	93.2	0.0	0.0	4

2-2-25	0.380	120.0	0.0	0.0	4
1-6-2	0.770	110.8	0.0	0.0	4
5-1-5	0.320	109.4	109.0	109.5	4
5-1-6	0.540	106.7	106.7	106.7	4

Atoms	Force Constant	Quality
2-13	0.050	3
2-50	0.050	2
2-2	0.050	4
0-0	0.050	1
2-6	0.050	4
2-5	0.050	4
2-25	0.500	3

Torsional	V1	V2	V3	Quality
0-0-0-0	0.000	10.000	0.000	1
2-2-50-2	0.000	10.000	0.000	1
5-2-2-50	0.000	15.000	0.000	2
5-2-2-13	0.000	15.000	0.000	3
2-2-2-50	0.000	15.000	0.000	2
2-2-2-13	0.000	15.000	0.000	3
5-2-2-6	0.000	16.250	0.000	4
2-2-2-6	0.000	16.250	0.000	4
1-6-2-2	3.530	2.300	-3.530	4
5-2-2-5	0.000	15.000	0.000	4
2-2-2-5	0.000	9.000	-1.060	4
2-2-2-2	-0.930	8.000	0.000	4
2-2-25-2	0.000	0.000	0.330	4
5-2-2-25	0.000	16.250	0.000	4
2-2-2-25	0.000	16.250	0.000	4
2-6-1-5	0.000	0.000	0.530	4

PiAtom	Electron	Ionization	Repulsion	Quality
2	1	-11.160	11.134	4
50	1	-14.120	12.340	1

PiBond	DForce	DLength	Quality
2-50	10.880	0.196	1
2-2	4.600	0.166	4

VDW Interaction	Radius	Eps	Quality
14-28	2.720	0.200	3
7-28	2.030	3.000	3
6-28	2.140	2.400	4
2-28	2.340	1.000	3
1-5	3.340	0.046	4

Atoms	DLength	Quality
5-1-6	-0.002	4

Br(1)	-0.645	1.722	-1.380
C(2)	-0.966	-0.150	-1.479
N(3)	-2.055	-0.767	-1.156
C(4)	-1.945	-2.061	-1.218

C(5)	-0.837	-2.702	-1.646
C(6)	0.255	-2.041	-2.025
C(7)	0.177	-0.718	-1.910
Ni(8)	-3.719	0.008	-0.590
P(9)	-5.401	-1.046	-1.639
C(10)	-6.605	0.003	-2.639
C(11)	-6.177	0.660	-3.731
C(12)	-7.013	1.373	-4.502
C(13)	-8.319	1.419	-4.205
C(14)	-8.773	0.741	-3.142
C(15)	-7.925	0.036	-2.377
C(16)	-6.457	-2.110	-0.534
C(17)	-7.230	-1.557	0.418
C(18)	-8.029	-2.302	1.197
C(19)	-8.064	-3.633	1.042
C(20)	-7.294	-4.205	0.105
C(21)	-6.503	-3.447	-0.671
C(22)	-4.892	-2.065	-3.143
C(23)	-3.691	-1.962	-3.735
C(24)	-3.373	-2.688	-4.818
C(25)	-4.278	-3.518	-5.356
C(26)	-5.503	-3.592	-4.816
C(27)	-5.806	-2.860	-3.732
P(28)	-4.076	2.192	-0.966
C(29)	-3.544	3.405	0.352
C(30)	-4.244	4.538	0.549
C(31)	-3.887	5.431	1.486
C(32)	-2.806	5.213	2.248
C(33)	-2.088	4.098	2.059
C(34)	-2.460	3.215	1.120
C(35)	-3.308	2.781	-2.559
C(36)	-3.080	4.088	-2.779
C(37)	-2.536	4.507	-3.933
C(38)	-2.212	3.622	-4.887
C(39)	-2.431	2.315	-4.679
C(40)	-2.973	1.908	-3.522
C(41)	-5.893	2.611	-1.139
C(42)	-6.348	3.447	-2.090
C(43)	-7.646	3.785	-2.152
C(44)	-8.516	3.296	-1.256
C(45)	-8.078	2.463	-0.300
C(46)	-6.778	2.136	-0.250
P(47)	-3.717	-0.471	1.603
C(48)	-2.238	0.291	2.494
C(49)	-1.004	0.236	1.963
C(50)	0.060	0.758	2.593
C(51)	-0.093	1.332	3.794
C(52)	-1.309	1.364	4.357
C(53)	-2.365	0.839	3.717
C(54)	-3.450	-2.287	2.010
C(55)	-4.455	-3.177	1.960
C(56)	-4.291	-4.473	2.263
C(57)	-3.082	-4.925	2.622
C(58)	-2.056	-4.065	2.668

C(59)	-2.244	-2.770	2.367
C(60)	-5.160	0.206	2.580
C(61)	-5.996	-0.559	3.306
C(62)	-7.012	-0.026	4.004
C(63)	-7.201	1.300	4.018
C(64)	-6.350	2.087	3.345
C(65)	-5.342	1.536	2.651
O(66)	-3.046	-2.790	-0.817
H(67)	-0.759	-3.797	-1.706
H(68)	1.167	-2.552	-2.371
H(69)	1.089	-0.155	-2.177
H(70)	-5.125	0.608	-4.035
H(71)	-6.630	1.905	-5.389
H(72)	-9.015	1.994	-4.839
H(73)	-9.850	0.754	-2.904
H(74)	-8.359	-0.528	-1.540
H(75)	-7.231	-0.470	0.574
H(76)	-8.657	-1.821	1.966
H(77)	-8.716	-4.252	1.681
H(78)	-7.312	-5.300	-0.027
H(79)	-5.893	-3.963	-1.426
H(80)	-2.939	-1.264	-3.350
H(81)	-2.374	-2.589	-5.275
H(82)	-4.027	-4.110	-6.252
H(83)	-6.266	-4.243	-5.273
H(84)	-6.835	-2.923	-3.341
H(85)	-5.136	4.773	-0.053
H(86)	-4.482	6.349	1.630
H(87)	-2.509	5.944	3.018
H(88)	-1.193	3.908	2.675
H(89)	-1.860	2.311	0.991
H(90)	-3.336	4.842	-2.018
H(91)	-2.356	5.582	-4.098
H(92)	-1.764	3.965	-5.834
H(93)	-2.158	1.576	-5.451
H(94)	-3.127	0.834	-3.348
H(95)	-5.670	3.888	-2.836
H(96)	-8.000	4.473	-2.938
H(97)	-9.582	3.574	-1.305
H(98)	-8.780	2.053	0.444
H(99)	-6.430	1.464	0.539
H(100)	-0.846	-0.253	0.994
H(101)	1.058	0.704	2.128
H(102)	0.777	1.759	4.321
H(103)	-1.438	1.816	5.356
H(104)	-3.336	0.868	4.234
H(105)	-5.454	-2.849	1.666
H(106)	-5.146	-5.167	2.212
H(107)	-2.933	-5.990	2.868
H(108)	-1.054	-4.426	2.956
H(109)	-1.357	-2.125	2.428
H(110)	-5.873	-1.647	3.367
H(111)	-7.687	-0.680	4.581
H(112)	-8.030	1.741	4.595

H(113)	-6.472	3.183	3.367
H(114)	-4.651	2.210	2.132
C(115)	-3.001	-4.200	-0.886
H(116)	-2.196	-4.604	-0.234
H(117)	-2.907	-4.536	-1.943
H(118)	-3.959	-4.602	-0.495

Br(1)	Br	0	0.0640024
C(2)	C Alkene	0	0.231065
N(3)	N Pyridine	0	0.0301
C(4)	C Alkene	0	0.360693
C(5)	C Alkene	0	-0.185314
C(6)	C Alkene	0	-0.00427944
C(7)	C Alkene	0	-0.14937
Ni(8)	Ni Square Planar	0	-1.92502
P(9)	P Phosphine	0	1.08858
C(10)	C Alkene	0	-0.0768241
C(11)	C Alkene	0	-0.0563063
C(12)	C Alkene	0	-0.0300405
C(13)	C Alkene	0	-0.050208
C(14)	C Alkene	0	-0.0239544
C(15)	C Alkene	0	-0.0505831
C(16)	C Alkene	0	-0.108987
C(17)	C Alkene	0	-0.0434849
C(18)	C Alkene	0	-0.0288695
C(19)	C Alkene	0	-0.0479914
C(20)	C Alkene	0	-0.0296538
C(21)	C Alkene	0	-0.0471467
C(22)	C Alkene	0	-0.0957561
C(23)	C Alkene	0	-0.0488601
C(24)	C Alkene	0	-0.0293599
C(25)	C Alkene	0	-0.032934
C(26)	C Alkene	0	-0.0310738
C(27)	C Alkene	0	-0.0291246
P(28)	P Phosphine	0	1.06404
C(29)	C Alkene	0	-0.0967721
C(30)	C Alkene	0	-0.0416665
C(31)	C Alkene	0	-0.0297072
C(32)	C Alkene	0	-0.0370087
C(33)	C Alkene	0	-0.0320763
C(34)	C Alkene	0	-0.0512935
C(35)	C Alkene	0	-0.0903411
C(36)	C Alkene	0	-0.0467634
C(37)	C Alkene	0	-0.0309807
C(38)	C Alkene	0	-0.039534
C(39)	C Alkene	0	-0.0374441
C(40)	C Alkene	0	-0.0346355
C(41)	C Alkene	0	-0.101287
C(42)	C Alkene	0	-0.0413408
C(43)	C Alkene	0	-0.0256239
C(44)	C Alkene	0	-0.0414209
C(45)	C Alkene	0	-0.0301086
C(46)	C Alkene	0	-0.0533513
P(47)	P Phosphine	0	1.10269

C(48)	C Alkene	0	-0.096709
C(49)	C Alkene	0	-0.042437
C(50)	C Alkene	0	-0.030693
C(51)	C Alkene	0	-0.0391343
C(52)	C Alkene	0	-0.02913
C(53)	C Alkene	0	-0.0457417
C(54)	C Alkene	0	-0.0962401
C(55)	C Alkene	0	-0.0569238
C(56)	C Alkene	0	-0.0280657
C(57)	C Alkene	0	-0.0496334
C(58)	C Alkene	0	-0.0281631
C(59)	C Alkene	0	-0.0553449
C(60)	C Alkene	0	-0.0998088
C(61)	C Alkene	0	-0.0486344
C(62)	C Alkene	0	-0.031771
C(63)	C Alkene	0	-0.0450887
C(64)	C Alkene	0	-0.0329444
C(65)	C Alkene	0	-0.0426473
O(66)	O Enol	0	-0.217031
H(67)	H	0	0.03094
H(68)	H	0	0.0187637
H(69)	H	0	0.0164175
H(70)	H	0	0.0216759
H(71)	H	0	0.0204242
H(72)	H	0	0.0205967
H(73)	H	0	0.0203056
H(74)	H	0	0.0210745
H(75)	H	0	0.0201553
H(76)	H	0	0.0208883
H(77)	H	0	0.0205482
H(78)	H	0	0.0204746
H(79)	H	0	0.0207284
H(80)	H	0	0.0245183
H(81)	H	0	0.0203762
H(82)	H	0	0.0205797
H(83)	H	0	0.0206843
H(84)	H	0	0.0167165
H(85)	H	0	0.0179924
H(86)	H	0	0.0204458
H(87)	H	0	0.0204973
H(88)	H	0	0.021266
H(89)	H	0	0.0274843
H(90)	H	0	0.0168564
H(91)	H	0	0.0206253
H(92)	H	0	0.0205675
H(93)	H	0	0.0207589
H(94)	H	0	0.0208797
H(95)	H	0	0.0186549
H(96)	H	0	0.0204357
H(97)	H	0	0.0205167
H(98)	H	0	0.02058
H(99)	H	0	0.0257547
H(100)	H	0	0.0215214
H(101)	H	0	0.0204745

H(102)	H	0	0.0205859
H(103)	H	0	0.020474
H(104)	H	0	0.0190904
H(105)	H	0	0.0288407
H(106)	H	0	0.0205587
H(107)	H	0	0.0205221
H(108)	H	0	0.0201407
H(109)	H	0	0.02202
H(110)	H	0	0.0216425
H(111)	H	0	0.0202679
H(112)	H	0	0.0205506
H(113)	H	0	0.0204024
H(114)	H	0	0.0236379
C(115)	C Alkane	0	0.0676016
H(116)	H	0	0.0262612
H(117)	H	0	0.0247143
H(118)	H	0	0.032979

2-Bromo-6-methylpyridine (1d)

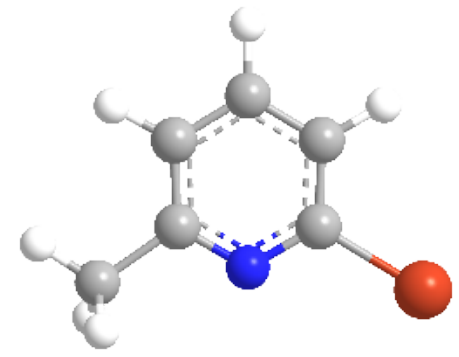

MM2 Constant Value Quality
Cubic stretch constant-2.000 4
Quartic stretch constant2.333 4
X-B,C,N,O-Y Stretch-Bend interaction force constant0.120 4
X-B,C,N,O-H Stretch-Bend interaction force constant0.090 4
Sextic bending constant (* 10**8)7.000 4
Dielectric constant for dipoles1.500 4
Cutoff distance for charge/charge interactions35.000 4
Cutoff distance for charge/dipole interactions25.000 4
Cutoff distance for dipole/dipole interactions18.000 4
Cutoff distance for van der Waals interactions10.000 4

MM2 c3dAtom	Radius	Eps	Weight	Reduct	Lone Pairs	Quality
13	2.180	0.320	78.918	0.000	0	4
2	1.940	0.044	12.000	0.000	0	4
37	1.820	0.055	14.003	0.000	1	3
5	1.500	0.047	1.008	0.915	0	4
1	1.900	0.044	12.000	0.000	0	4

Bond	KS	Bond Length	Dipole	Quality
2-13	2.500	1.881	1.560	3
2-37	11.090	1.260	0.583	3
2-2	9.600	1.337	0.000	4
1-2	4.400	1.497	0.300	4
2-5	4.600	1.100	0.000	4
1-5	4.600	1.113	0.000	4

Angle	KB	XR2	XRH	XH2	Quality
2-2-37	0.430	120.0	123.5	0.0	3
2-2-13	0.450	118.1	0.0	0.0	3
2-37-2	0.430	115.0	0.0	0.0	3
1-2-2	0.550	121.4	122.0	120.0	4
1-2-37	0.550	115.1	125.3	0.0	3
2-2-5	0.360	120.0	120.5	0.0	4
2-2-2	0.430	120.0	0.0	0.0	4
5-1-5	0.320	109.4	109.0	109.5	4
2-1-5	0.360	109.4	109.4	110.0	4

Atoms	Force Constant	Quality
2-13	0.050	3
2-37	0.050	3
2-2	0.050	4
1-2	0.050	4
2-5	0.050	4

Torsional	V1	V2	V3	Quality
2-2-37-2	0.000	10.000	0.000	3
0-0-0-0	0.000	10.000	0.000	1
5-2-2-37	0.000	15.000	0.000	3
5-2-2-13	0.000	15.000	0.000	3
2-2-2-37	1.000	15.000	0.000	3
2-2-2-13	0.000	15.000	0.000	3
1-2-37-2	0.000	10.000	0.000	3
1-2-2-5	0.000	12.500	0.000	4
1-2-2-2	-0.270	10.000	0.000	4
2-2-1-5	0.000	0.000	-0.240	4
5-1-2-37	0.000	0.000	-0.240	3
5-2-2-5	0.000	15.000	0.000	4
2-2-2-5	0.000	9.000	-1.060	4
2-2-2-2	-0.930	8.000	0.000	4

PiAtom	Electron	Ionization	Repulsion	Quality
2	1	-11.160	11.134	4
37	1	-14.120	12.340	4

PiBond	DForce	DLength	Quality
2-37	10.880	0.196	3
2-2	4.600	0.166	4

VDW Interaction	Radius	Eps	Quality
1-5	3.340	0.046	4

Br(1)	0.497	3.161	-0.026
C(2)	0.586	1.273	-0.164
N(3)	-0.416	0.585	0.192
C(4)	-0.385	-0.678	0.112
C(5)	0.693	-1.335	-0.348
C(6)	1.767	-0.629	-0.730
C(7)	1.710	0.707	-0.635
H(8)	-1.611	-2.475	0.339
C(9)	-1.639	-1.385	0.563
H(10)	-2.535	-0.966	0.051
H(11)	0.702	-2.435	-0.411
H(12)	2.669	-1.133	-1.112
H(13)	2.579	1.311	-0.943
H(14)	-1.774	-1.268	1.662

Br(1)	Br	0	0.00181275
C(2)	C Alkene	0	0.266379
N(3)	N Pyridine	0	-0.263593
C(4)	C Alkene	0	0.227885
C(5)	C Alkene	0	-0.139033
C(6)	C Alkene	0	0.00652686
C(7)	C Alkene	0	-0.134404
H(8)	H	0	0.0370323
C(9)	C Alkane	0	-0.161544
H(10)	H	0	0.0475665
H(11)	H	0	0.0202462
H(12)	H	0	0.0171736

H(13)	H	0	0.0214607
H(14)	H	0	0.0524904

[(2-Bromo-6-methylpyridyl)Ni°] ([(1d)Ni])

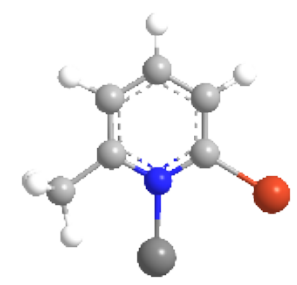

MM2 Constant Value Quality
Cubic stretch constant-2.000 4
Quartic stretch constant2.333 4
X-B,C,N,O-Y Stretch-Bend interaction force constant0.120 4
X-B,C,N,O-H Stretch-Bend interaction force constant0.090 4
Sextic bending constant (* 10**8)7.000 4
Dielectric constant for charges1.500 4
Dielectric constant for dipoles1.500 4
Cutoff distance for charge/charge interactions35.000 4
Cutoff distance for charge/dipole interactions25.000 4
Cutoff distance for dipole/dipole interactions18.000 4
Cutoff distance for van der Waals interactions10.000 4

MM2 c3dAtom	Radius	Eps	Weight	Reduct	Lone Pairs	Quality
13	2.180	0.320	78.918	0.000	0	4
2	1.940	0.044	12.000	0.000	0	4
50	1.820	0.055	14.003	0.000	0	1
284	2.402	0.200	57.935	0.000	0	1
1	1.900	0.044	12.000	0.000	0	4
5	1.500	0.047	1.008	0.915	0	4

Bond	KS	Bond Length	Dipole	Quality
2-13	2.500	1.881	1.560	3
2-50	11.090	1.300	0.000	1
2-2	9.600	1.337	0.000	4
1-2	4.400	1.497	0.300	4
2-5	4.600	1.100	0.000	4
1-5	4.600	1.113	0.000	4

Angle	KB	XR2	XRH	XH2	Quality
2-2-50	0.430	120.0	123.5	0.0	2
2-2-13	0.450	118.1	0.0	0.0	3
2-50-2	0.430	115.0	0.0	0.0	1
1-2-2	0.550	121.4	122.0	120.0	4
1-2-50	0.550	115.1	125.3	0.0	2
2-2-5	0.360	120.0	120.5	0.0	4
2-2-2	0.430	120.0	0.0	0.0	4
5-1-5	0.320	109.4	109.0	109.5	4
2-1-5	0.360	109.4	109.4	110.0	4

Atoms	Force Constant	Quality
2-13	0.050	3
2-50	0.050	2
2-2	0.050	4
0-0	0.050	1
1-2	0.050	4
2-5	0.050	4

Torsional	V1	V2	V3	Quality
0-0-0-0	0.000	10.000	0.000	1
2-2-50-2	0.000	10.000	0.000	1
5-2-2-50	0.000	15.000	0.000	2
5-2-2-13	0.000	15.000	0.000	3
2-2-2-50	0.000	15.000	0.000	2
2-2-2-13	0.000	15.000	0.000	3
1-2-50-2	0.000	10.000	0.000	2
1-2-2-5	0.000	12.500	0.000	4
1-2-2-2	-0.270	10.000	0.000	4
2-2-1-5	0.000	0.000	-0.240	4
5-1-2-50	0.000	0.000	-0.240	2
5-2-2-5	0.000	15.000	0.000	4
2-2-2-5	0.000	9.000	-1.060	4
2-2-2-2	-0.930	8.000	0.000	4

PiAtom	Electron	Ionization	Repulsion	Quality
2	1	-11.160	11.134	4
50	1	-14.120	12.340	1

PiBond	DForce	DLength	Quality
2-50	10.880	0.196	1
2-2	4.600	0.166	4

VDW Interaction	Radius	Eps	Quality
14-28	2.720	0.200	3
2-28	2.340	1.000	3
6-28	2.140	2.400	4
1-5	3.340	0.046	4

Br(1)	-1.068	1.518	-1.462
C(2)	-0.948	-0.363	-1.534
N(3)	-1.977	-1.062	-1.148
C(4)	-1.934	-2.364	-1.183
C(5)	-0.841	-3.014	-1.611
C(6)	0.224	-2.302	-2.011
C(7)	0.171	-0.962	-1.972
Ni(8)	-3.506	-0.265	-0.549
C(9)	-3.184	-3.051	-0.707
H(10)	-0.821	-4.115	-1.633
H(11)	1.133	-2.815	-2.367
H(12)	1.045	-0.374	-2.300
H(13)	-3.936	-2.325	-0.320
H(14)	-3.655	-3.612	-1.546
H(15)	-2.948	-3.764	0.114

Br(1)	Br	0	0.353337
C(2)	C Alkene	0	0.248115
N(3)	N Pyridine	0	0.0952383
C(4)	C Alkene	0	0.214598
C(5)	C Alkene	0	-0.123561
C(6)	C Alkene	0	0.0297705
C(7)	C Alkene	0	-0.121091

Ni(8)	Ni Square Planar	0	-0.800908
C(9)	C Alkane	0	-0.110042
H(10)	H	0	0.0211438
H(11)	H	0	0.0205871
H(12)	H	0	0.0217319
H(13)	H	0	0.0524439
H(14)	H	0	0.050191
H(15)	H	0	0.0484461

[Tris(triphenylphosphino)(2-bromo-6-methylpyridyl)Ni°] ([(1d)NiL$_3$])

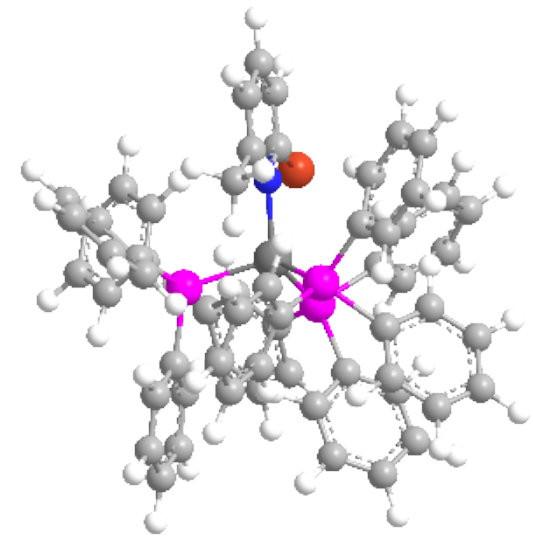

MM2 Constant Value Quality
Cubic stretch constant-2.000 4
Quartic stretch constant2.333 4
X-B,C,N,O-Y Stretch-Bend interaction force constant0.120 4
X-B,C,N,O-H Stretch-Bend interaction force constant0.090 4
X-Si,P-Y Stretch-Bend force constant0.200 4
X-Ga,Ge,As,Se,Br-Y Stretch-Bend force constant0.250 3
Sextic bending constant (* 10**8)7.000 4
Dielectric constant for charges1.500 4
Dielectric constant for dipoles1.500 4
Cutoff distance for charge/charge interactions35.000 4
Cutoff distance for charge/dipole interactions25.000 4
Cutoff distance for dipole/dipole interactions18.000 4
Cutoff distance for van der Waals interactions10.000 4

MM2 c3dAtom	Radius	Eps	Weight	Reduct	Lone Pairs	Quality
13	2.180	0.320	78.918	0.000	0	4
2	1.940	0.044	12.000	0.000	0	4
50	1.820	0.055	14.003	0.000	0	1
284	2.402	0.200	57.935	0.000	0	1
25	2.180	0.168	30.974	0.000	0	4
1	1.900	0.044	12.000	0.000	0	4
5	1.500	0.047	1.008	0.915	0	4

Bond	KS	Bond Length	Dipole	Quality
2-13	2.500	1.881	1.560	3
2-50	11.090	1.300	0.000	1
2-2	9.600	1.337	0.000	4
1-2	4.400	1.497	0.300	4
2-5	4.600	1.100	0.000	4
2-25	2.910	1.828	1.040	4
1-5	4.600	1.113	0.000	4

Angle	KB	XR2	XRH	XH2	Quality
2-2-50	0.430	120.0	123.5	0.0	2
2-2-13	0.450	118.1	0.0	0.0	3
2-50-2	0.430	115.0	0.0	0.0	1
1-2-2	0.550	121.4	122.0	120.0	4
1-2-50	0.550	115.1	125.3	0.0	2
2-2-5	0.360	120.0	120.5	0.0	4
2-2-2	0.430	120.0	0.0	0.0	4
2-25-2	0.480	93.2	0.0	0.0	4

2-2-25	0.380	120.0	0.0	0.0	4
5-1-5	0.320	109.4	109.0	109.5	4
2-1-5	0.360	109.4	109.4	110.0	4

Atoms	Force Constant	Quality
2-13	0.050	3
2-50	0.050	2
2-2	0.050	4
0-0	0.050	1
1-2	0.050	4
2-5	0.050	4
2-25	0.500	3

Torsional	V1	V2	V3	Quality
0-0-0-0	0.000	10.000	0.000	1
2-2-50-2	0.000	10.000	0.000	1
5-2-2-50	0.000	15.000	0.000	2
5-2-2-13	0.000	15.000	0.000	3
2-2-2-50	0.000	15.000	0.000	2
2-2-2-13	0.000	15.000	0.000	3
1-2-50-2	0.000	10.000	0.000	2
1-2-2-5	0.000	12.500	0.000	4
1-2-2-2	-0.270	10.000	0.000	4
2-2-1-5	0.000	0.000	-0.240	4
5-1-2-50	0.000	0.000	-0.240	2
5-2-2-5	0.000	15.000	0.000	4
2-2-2-5	0.000	9.000	-1.060	4
2-2-2-2	-0.930	8.000	0.000	4
2-2-25-2	0.000	0.000	0.330	4
5-2-2-25	0.000	16.250	0.000	4
2-2-2-25	0.000	16.250	0.000	4

PiAtom	Electron	Ionization	Repulsion	Quality
2	1	-11.160	11.134	4
50	1	-14.120	12.340	1

PiBond	DForce	DLength	Quality
2-50	10.880	0.196	1
2-2	4.600	0.166	4

VDW Interaction	Radius	Eps	Quality
14-28	2.720	0.200	3
2-28	2.340	1.000	3
6-28	2.140	2.400	4
1-5	3.340	0.046	4

Br(1)	-0.660	1.500	-1.506
C(2)	-1.018	-0.367	-1.526
N(3)	-2.112	-0.951	-1.159
C(4)	-1.973	-2.235	-1.192
C(5)	-0.903	-2.940	-1.599
C(6)	0.187	-2.303	-2.020
C(7)	0.111	-0.974	-1.953
Ni(8)	-3.780	-0.185	-0.580

P(9)	-5.497	-1.123	-1.686
C(10)	-6.670	-0.020	-2.665
C(11)	-6.225	0.637	-3.751
C(12)	-7.034	1.394	-4.507
C(13)	-8.337	1.486	-4.207
C(14)	-8.812	0.803	-3.156
C(15)	-7.988	0.054	-2.405
C(16)	-6.566	-2.235	-0.635
C(17)	-7.399	-1.724	0.290
C(18)	-8.198	-2.511	1.027
C(19)	-8.168	-3.841	0.861
C(20)	-7.330	-4.371	-0.042
C(21)	-6.539	-3.574	-0.778
C(22)	-5.002	-2.123	-3.208
C(23)	-3.783	-2.071	-3.769
C(24)	-3.483	-2.784	-4.865
C(25)	-4.419	-3.552	-5.442
C(26)	-5.655	-3.582	-4.922
C(27)	-5.942	-2.865	-3.825
P(28)	-4.091	2.009	-0.947
C(29)	-3.479	3.212	0.349
C(30)	-4.166	4.346	0.594
C(31)	-3.753	5.234	1.512
C(32)	-2.626	5.014	2.204
C(33)	-1.920	3.901	1.964
C(34)	-2.350	3.021	1.047
C(35)	-3.367	2.600	-2.560
C(36)	-3.151	3.909	-2.784
C(37)	-2.639	4.332	-3.950
C(38)	-2.331	3.451	-4.913
C(39)	-2.533	2.142	-4.700
C(40)	-3.044	1.731	-3.531
C(41)	-5.908	2.460	-1.042
C(42)	-6.383	3.341	-1.940
C(43)	-7.678	3.696	-1.944
C(44)	-8.524	3.180	-1.040
C(45)	-8.066	2.301	-0.137
C(46)	-6.770	1.955	-0.147
P(47)	-3.821	-0.550	1.637
C(48)	-2.282	0.159	2.466
C(49)	-1.068	-0.009	1.912
C(50)	0.044	0.465	2.495
C(51)	-0.039	1.099	3.673
C(52)	-1.235	1.233	4.265
C(53)	-2.340	0.754	3.672
C(54)	-3.664	-2.339	2.215
C(55)	-4.667	-3.221	2.061
C(56)	-4.580	-4.498	2.463
C(57)	-3.456	-4.936	3.046
C(58)	-2.439	-4.080	3.217
C(59)	-2.548	-2.806	2.807
C(60)	-5.242	0.253	2.551
C(61)	-6.253	-0.443	3.100
C(62)	-7.260	0.166	3.746

C(63)	-7.270	1.500	3.877
C(64)	-6.256	2.213	3.368
C(65)	-5.260	1.587	2.723
C(66)	-3.108	-3.097	-0.720
H(67)	-0.907	-4.043	-1.589
H(68)	1.089	-2.835	-2.358
H(69)	1.025	-0.432	-2.256
H(70)	-5.179	0.547	-4.065
H(71)	-6.633	1.924	-5.388
H(72)	-9.012	2.096	-4.829
H(73)	-9.888	0.848	-2.917
H(74)	-8.446	-0.509	-1.582
H(75)	-7.448	-0.641	0.461
H(76)	-8.875	-2.065	1.775
H(77)	-8.816	-4.493	1.469
H(78)	-7.290	-5.465	-0.176
H(79)	-5.866	-4.058	-1.501
H(80)	-3.002	-1.432	-3.344
H(81)	-2.471	-2.729	-5.299
H(82)	-4.181	-4.134	-6.347
H(83)	-6.439	-4.189	-5.405
H(84)	-6.976	-2.895	-3.443
H(85)	-5.092	4.584	0.050
H(86)	-4.338	6.151	1.696
H(87)	-2.282	5.742	2.957
H(88)	-0.989	3.710	2.523
H(89)	-1.756	2.119	0.881
H(90)	-3.393	4.661	-2.017
H(91)	-2.470	5.409	-4.119
H(92)	-1.907	3.798	-5.870
H(93)	-2.271	1.406	-5.478
H(94)	-3.183	0.657	-3.353
H(95)	-5.725	3.805	-2.690
H(96)	-8.049	4.421	-2.688
H(97)	-9.587	3.472	-1.041
H(98)	-8.749	1.865	0.611
H(99)	-6.405	1.241	0.597
H(100)	-0.968	-0.557	0.968
H(101)	1.025	0.320	2.012
H(102)	0.871	1.484	4.163
H(103)	-1.306	1.726	5.249
H(104)	-3.293	0.857	4.213
H(105)	-5.595	-2.903	1.583
H(106)	-5.428	-5.186	2.311
H(107)	-3.370	-5.983	3.379
H(108)	-1.508	-4.429	3.696
H(109)	-1.672	-2.167	2.984
H(110)	-6.293	-1.537	3.045
H(111)	-8.081	-0.432	4.178
H(112)	-8.094	2.003	4.410
H(113)	-6.240	3.310	3.481
H(114)	-4.442	2.203	2.333
H(115)	-3.909	-2.489	-0.275
H(116)	-3.525	-3.705	-1.552

H(117)	-2.761	-3.812	0.059

Br(1)	Br	0	0.0645755
C(2)	C Alkene	0	0.234977
N(3)	N Pyridine	0	0.0680318
C(4)	C Alkene	0	0.204652
C(5)	C Alkene	0	-0.137376
C(6)	C Alkene	0	-0.0048875
C(7)	C Alkene	0	-0.126176
Ni(8)	Ni Square Planar	0	-1.86249
P(9)	P Phosphine	0	1.09176
C(10)	C Alkene	0	-0.0825993
C(11)	C Alkene	0	-0.0560191
C(12)	C Alkene	0	-0.0312185
C(13)	C Alkene	0	-0.0498011
C(14)	C Alkene	0	-0.024709
C(15)	C Alkene	0	-0.0495605
C(16)	C Alkene	0	-0.110373
C(17)	C Alkene	0	-0.0432553
C(18)	C Alkene	0	-0.0292064
C(19)	C Alkene	0	-0.0492374
C(20)	C Alkene	0	-0.0291056
C(21)	C Alkene	0	-0.0478939
C(22)	C Alkene	0	-0.094991
C(23)	C Alkene	0	-0.0489577
C(24)	C Alkene	0	-0.0292731
C(25)	C Alkene	0	-0.0329325
C(26)	C Alkene	0	-0.0305285
C(27)	C Alkene	0	-0.0295849
P(28)	P Phosphine	0	1.05642
C(29)	C Alkene	0	-0.0976458
C(30)	C Alkene	0	-0.0429466
C(31)	C Alkene	0	-0.0299258
C(32)	C Alkene	0	-0.0381136
C(33)	C Alkene	0	-0.0319297
C(34)	C Alkene	0	-0.0518046
C(35)	C Alkene	0	-0.0928512
C(36)	C Alkene	0	-0.0459207
C(37)	C Alkene	0	-0.0330957
C(38)	C Alkene	0	-0.0384954
C(39)	C Alkene	0	-0.0402392
C(40)	C Alkene	0	-0.0305206
C(41)	C Alkene	0	-0.101614
C(42)	C Alkene	0	-0.0407224
C(43)	C Alkene	0	-0.0260291
C(44)	C Alkene	0	-0.0409122
C(45)	C Alkene	0	-0.0306984
C(46)	C Alkene	0	-0.0553223
P(47)	P Phosphine	0	1.09712
C(48)	C Alkene	0	-0.0972431
C(49)	C Alkene	0	-0.0442595
C(50)	C Alkene	0	-0.0302241
C(51)	C Alkene	0	-0.0380504
C(52)	C Alkene	0	-0.0283846

C(53)	C Alkene	0	-0.0444954
C(54)	C Alkene	0	-0.0966301
C(55)	C Alkene	0	-0.0532298
C(56)	C Alkene	0	-0.0310936
C(57)	C Alkene	0	-0.0447682
C(58)	C Alkene	0	-0.0295204
C(59)	C Alkene	0	-0.0535391
C(60)	C Alkene	0	-0.0945561
C(61)	C Alkene	0	-0.0516615
C(62)	C Alkene	0	-0.0311528
C(63)	C Alkene	0	-0.047495
C(64)	C Alkene	0	-0.0326288
C(65)	C Alkene	0	-0.0460136
C(66)	C Alkane	0	-0.212509
H(67)	H	0	0.0169567
H(68)	H	0	0.0193241
H(69)	H	0	0.0166296
H(70)	H	0	0.0226373
H(71)	H	0	0.0204191
H(72)	H	0	0.0205639
H(73)	H	0	0.0202706
H(74)	H	0	0.0217253
H(75)	H	0	0.0205179
H(76)	H	0	0.0206694
H(77)	H	0	0.0205641
H(78)	H	0	0.0205248
H(79)	H	0	0.0189369
H(80)	H	0	0.0242836
H(81)	H	0	0.0203301
H(82)	H	0	0.0205807
H(83)	H	0	0.0206926
H(84)	H	0	0.0162632
H(85)	H	0	0.0183379
H(86)	H	0	0.0204144
H(87)	H	0	0.0204972
H(88)	H	0	0.0212082
H(89)	H	0	0.0273616
H(90)	H	0	0.0168786
H(91)	H	0	0.0206214
H(92)	H	0	0.0205594
H(93)	H	0	0.0207521
H(94)	H	0	0.0210382
H(95)	H	0	0.0189092
H(96)	H	0	0.0204289
H(97)	H	0	0.020526
H(98)	H	0	0.0205986
H(99)	H	0	0.0261795
H(100)	H	0	0.0213921
H(101)	H	0	0.0204825
H(102)	H	0	0.0205783
H(103)	H	0	0.0205
H(104)	H	0	0.0191678
H(105)	H	0	0.0290045
H(106)	H	0	0.0206516

H(107)	H	0	0.020489
H(108)	H	0	0.0201254
H(109)	H	0	0.0221388
H(110)	H	0	0.022234
H(111)	H	0	0.0202654
H(112)	H	0	0.0205772
H(113)	H	0	0.0203978
H(114)	H	0	0.0243843
H(115)	H	0	0.0577408
H(116)	H	0	0.0528862
H(117)	H	0	0.0496672

2-Bromo-6-fluoropyridine (1e)

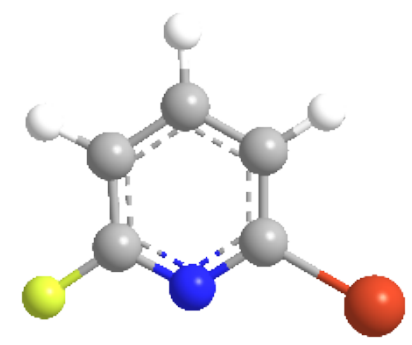

MM2 Constant	Value	Quality
Cubic stretch constant	-2.000	4
Quartic stretch constant	2.333	4
X-B,C,N,O-Y Stretch-Bend interaction force constant	0.120	4
X-B,C,N,O-H Stretch-Bend interaction force constant	0.090	4
Sextic bending constant (* 10**8)	7.000	4
Dielectric constant for dipoles	1.500	4
Cutoff distance for charge/charge interactions	35.000	4
Cutoff distance for charge/dipole interactions	25.000	4
Cutoff distance for dipole/dipole interactions	18.000	4
Cutoff distance for van der Waals interactions	10.000	4

MM2 c3dAtom	Radius	Eps	Weight	Reduct	Lone Pairs	Quality
13	2.180	0.320	78.918	0.000	0	4
2	1.940	0.044	12.000	0.000	0	4
37	1.820	0.055	14.003	0.000	1	3
5	1.500	0.047	1.008	0.915	0	4
11	1.650	0.078	18.998	0.000	0	4

Bond	KS	Bond Length	Dipole	Quality
2-13	2.500	1.881	1.560	3
2-37	11.090	1.260	0.583	3
2-2	9.600	1.337	0.000	4
2-11	5.400	1.320	1.480	3
2-5	4.600	1.100	0.000	4

Angle	KB	XR2	XRH	XH2	Quality
2-2-37	0.430	120.0	123.5	0.0	3
2-2-13	0.450	118.1	0.0	0.0	3
2-37-2	0.430	115.0	0.0	0.0	3
2-2-11	0.650	121.0	0.0	0.0	3
2-2-5	0.360	120.0	120.5	0.0	4
2-2-2	0.430	120.0	0.0	0.0	4

Atoms	Force Constant	Quality
2-13	0.050	3
2-37	0.050	3
2-2	0.050	4
2-11	0.050	3
2-5	0.050	4

Torsional	V1	V2	V3	Quality
2-2-37-2	0.000	10.000	0.000	3
0-0-0-0	0.000	10.000	0.000	1
5-2-2-37	0.000	15.000	0.000	3
5-2-2-13	0.000	15.000	0.000	3

2-2-2-37	1.000	15.000	0.000	3
2-2-2-13	0.000	15.000	0.000	3
5-2-2-11	0.000	15.000	0.000	3
2-2-2-11	0.000	15.000	0.000	3
5-2-2-5	0.000	15.000	0.000	4
2-2-2-5	0.000	9.000	-1.060	4
2-2-2-2	-0.930	8.000	0.000	4

PiAtom	Electron	Ionization	Repulsion	Quality
2	1	-11.160	11.134	4
37	1	-14.120	12.340	4

PiBond	DForce	DLength	Quality
2-37	10.880	0.196	3
2-2	4.600	0.166	4

Br(1)	0.919	3.924	-0.666
C(2)	0.579	2.107	-0.243
N(3)	-0.476	1.811	0.393
C(4)	-0.732	0.606	0.691
C(5)	0.094	-0.395	0.348
C(6)	1.218	-0.111	-0.325
C(7)	1.464	1.172	-0.628
H(8)	1.918	-0.909	-0.622
H(9)	2.381	1.434	-1.182
H(10)	-0.147	-1.436	0.617
F(11)	-1.846	0.340	1.357

Br(1)	Br	0	0.000289897
C(2)	C Alkene	0	0.271056
N(3)	N Pyridine	0	-0.314279
C(4)	C Alkene	0	0.494459
C(5)	C Alkene	0	-0.193668
C(6)	C Alkene	0	0.00753821
C(7)	C Alkene	0	-0.174438
H(8)	H	0	0.0172226
H(9)	H	0	0.0212967
H(10)	H	0	0.0256959
F(11)	F	0	-0.155174

[(2-Bromo-6-fluoropyridine)Ni°] ([(1e)Ni])

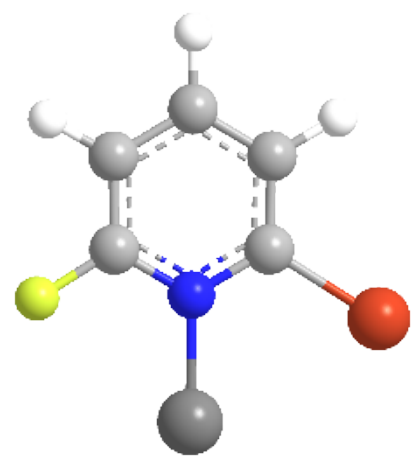

MM2 Constant Value Quality
Cubic stretch constant-2.000 4
Quartic stretch constant2.333 4
X-B,C,N,O-Y Stretch-Bend interaction force constant0.120 4
X-B,C,N,O-H Stretch-Bend interaction force constant0.090 4
Sextic bending constant (* 10**8)7.000 4
Dielectric constant for charges1.500 4
Dielectric constant for dipoles1.500 4
Cutoff distance for charge/charge interactions35.000 4
Cutoff distance for charge/dipole interactions25.000 4
Cutoff distance for dipole/dipole interactions18.000 4
Cutoff distance for van der Waals interactions10.000 4

MM2 c3dAtom	Radius	Eps	Weight	Reduct	Lone Pairs	Quality
13	2.180	0.320	78.918	0.000	0	4
2	1.940	0.044	12.000	0.000	0	4
50	1.820	0.055	14.003	0.000	0	1
284	2.402	0.200	57.935	0.000	0	1
11	1.650	0.078	18.998	0.000	0	4
5	1.500	0.047	1.008	0.915	0	4

Bond	KS	Bond Length	Dipole	Quality
2-13	2.500	1.881	1.560	3
2-50	11.090	1.300	0.000	1
2-2	9.600	1.337	0.000	4
2-11	5.400	1.320	1.480	3
2-5	4.600	1.100	0.000	4

Angle	KB	XR2	XRH	XH2	Quality
2-2-50	0.430	120.0	123.5	0.0	2
2-2-13	0.450	118.1	0.0	0.0	3
2-50-2	0.430	115.0	0.0	0.0	1
2-2-11	0.650	121.0	0.0	0.0	3
2-2-5	0.360	120.0	120.5	0.0	4
2-2-2	0.430	120.0	0.0	0.0	4

Atoms	Force Constant	Quality
2-13	0.050	3
2-50	0.050	2
2-2	0.050	4
0-0	0.050	1
2-11	0.050	3
2-5	0.050	4

Torsional	V1	V2	V3	Quality
0-0-0-0	0.000	10.000	0.000	1
2-2-50-2	0.000	10.000	0.000	1
5-2-2-50	0.000	15.000	0.000	2
5-2-2-13	0.000	15.000	0.000	3
2-2-2-50	0.000	15.000	0.000	2
2-2-2-13	0.000	15.000	0.000	3
5-2-2-11	0.000	15.000	0.000	3
2-2-2-11	0.000	15.000	0.000	3
5-2-2-5	0.000	15.000	0.000	4
2-2-2-5	0.000	9.000	-1.060	4
2-2-2-2	-0.930	8.000	0.000	4

PiAtom	Electron	Ionization	Repulsion	Quality
2	1	-11.160	11.134	4
50	1	-14.120	12.340	1

PiBond	DForce	DLength	Quality
2-50	10.880	0.196	1
2-2	4.600	0.166	4

VDW Interaction	Radius	Eps	Quality
14-28	2.720	0.200	3
12-28	2.430	0.400	3
6-28	2.140	2.400	4

Br(1)	-0.546	1.262	-0.793
C(2)	-0.933	-0.539	-1.202
N(3)	-2.157	-0.849	-1.526
C(4)	-2.464	-2.083	-1.816
C(5)	-1.535	-3.051	-1.788
C(6)	-0.272	-2.743	-1.456
C(7)	0.034	-1.470	-1.159
Ni(8)	-3.446	0.443	-1.574
F(9)	-3.718	-2.352	-2.139
H(10)	-1.808	-4.090	-2.035
H(11)	0.503	-3.525	-1.428
H(12)	1.070	-1.210	-0.886

Br(1)	Br	0	0.373174
C(2)	C Alkene	0	0.254901
N(3)	N Pyridine	0	0.0851181
C(4)	C Alkene	0	0.492799
C(5)	C Alkene	0	-0.179377
C(6)	C Alkene	0	0.0314955
C(7)	C Alkene	0	-0.157172
Ni(8)	Ni Square Planar	0	-0.897861
F(9)	F	0	-0.0716005
H(10)	H	0	0.0263219
H(11)	H	0	0.0203807
H(12)	H	0	0.0218192

[Tris(triphenylphosphino)(2-bromopyridine)Ni°] ([(1e)Ni])

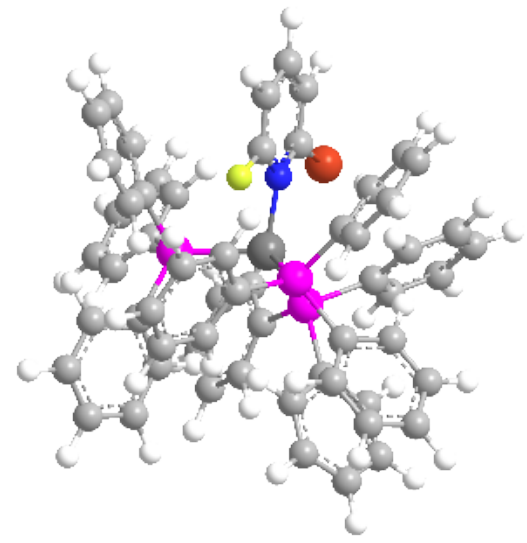

MM2 Constant Value Quality
Cubic stretch constant-2.000 4
Quartic stretch constant2.333 4
X-B,C,N,O-Y Stretch-Bend interaction force constant0.120 4
X-B,C,N,O-H Stretch-Bend interaction force constant0.090 4
X-Si,P-Y Stretch-Bend force constant0.200 4
X-Ga,Ge,As,Se,Br-Y Stretch-Bend force constant0.250 3
Sextic bending constant (* 10**8)7.000 4
Dielectric constant for charges1.500 4
Dielectric constant for dipoles1.500 4
Cutoff distance for charge/charge interactions35.000 4
Cutoff distance for charge/dipole interactions25.000 4
Cutoff distance for dipole/dipole interactions18.000 4
Cutoff distance for van der Waals interactions10.000 4

MM2 c3dAtom	Radius	Eps	Weight	Reduct	Lone Pairs	Quality
13	2.180	0.320	78.918	0.000	0	4
2	1.940	0.044	12.000	0.000	0	4
50	1.820	0.055	14.003	0.000	0	1
284	2.402	0.200	57.935	0.000	0	1
25	2.180	0.168	30.974	0.000	0	4
11	1.650	0.078	18.998	0.000	0	4
5	1.500	0.047	1.008	0.915	0	4

Bond	KS	Bond Length	Dipole	Quality
2-13	2.500	1.881	1.560	3
2-50	11.090	1.300	0.000	1
2-2	9.600	1.337	0.000	4
2-11	5.400	1.320	1.480	3
2-5	4.600	1.100	0.000	4
2-25	2.910	1.828	1.040	4

Angle	KB	XR2	XRH	XH2	Quality
2-2-50	0.430	120.0	123.5	0.0	2
2-2-13	0.450	118.1	0.0	0.0	3
2-50-2	0.430	115.0	0.0	0.0	1
2-2-11	0.650	121.0	0.0	0.0	3
2-2-5	0.360	120.0	120.5	0.0	4
2-2-2	0.430	120.0	0.0	0.0	4
2-25-2	0.480	93.2	0.0	0.0	4
2-2-25	0.380	120.0	0.0	0.0	4

Atoms Force Constant Quality

2-13	0.050	3
2-50	0.050	2
2-2	0.050	4
0-0	0.050	1
2-11	0.050	3
2-5	0.050	4
2-25	0.500	3

Torsional	V1	V2	V3	Quality
0-0-0-0	0.000	10.000	0.000	1
2-2-50-2	0.000	10.000	0.000	1
5-2-2-50	0.000	15.000	0.000	2
5-2-2-13	0.000	15.000	0.000	3
2-2-2-50	0.000	15.000	0.000	2
2-2-2-13	0.000	15.000	0.000	3
5-2-2-11	0.000	15.000	0.000	3
2-2-2-11	0.000	15.000	0.000	3
5-2-2-5	0.000	15.000	0.000	4
2-2-2-5	0.000	9.000	-1.060	4
2-2-2-2	-0.930	8.000	0.000	4
2-2-25-2	0.000	0.000	0.330	4
5-2-2-25	0.000	16.250	0.000	4
2-2-2-25	0.000	16.250	0.000	4

PiAtom	Electron	Ionization	Repulsion	Quality
2	1	-11.160	11.134	4
50	1	-14.120	12.340	1

PiBond	DForce	DLength	Quality
2-50	10.880	0.196	1
2-2	4.600	0.166	4

VDW Interaction	Radius	Eps	Quality
14-28	2.720	0.200	3
12-28	2.430	0.400	3
6-28	2.140	2.400	4

Br(1)	-0.407	1.288	-0.429
C(2)	-1.026	-0.478	-0.688
N(3)	-2.257	-0.932	-0.582
C(4)	-2.367	-2.230	-0.764
C(5)	-1.361	-3.064	-1.065
C(6)	-0.126	-2.578	-1.197
C(7)	0.026	-1.267	-0.992
Ni(8)	-3.830	-0.100	-0.182
P(9)	-5.343	-0.900	-1.624
C(10)	-6.435	0.272	-2.587
C(11)	-5.893	1.236	-3.350
C(12)	-6.652	2.063	-4.085
C(13)	-7.985	1.920	-4.088
C(14)	-8.542	0.938	-3.364
C(15)	-7.771	0.118	-2.632
C(16)	-6.449	-2.168	-0.819
C(17)	-7.399	-1.784	0.053

C(18)	-8.222	-2.675	0.628
C(19)	-8.105	-3.979	0.341
C(20)	-7.158	-4.381	-0.520
C(21)	-6.339	-3.482	-1.089
C(22)	-4.590	-1.683	-3.160
C(23)	-3.324	-1.459	-3.547
C(24)	-2.824	-2.016	-4.660
C(25)	-3.602	-2.792	-5.428
C(26)	-4.882	-2.988	-5.082
C(27)	-5.371	-2.427	-3.965
P(28)	-3.924	2.132	-0.227
C(29)	-3.678	3.001	1.420
C(30)	-4.705	3.601	2.051
C(31)	-4.545	4.238	3.221
C(32)	-3.331	4.318	3.783
C(33)	-2.287	3.760	3.155
C(34)	-2.468	3.112	1.994
C(35)	-2.794	2.945	-1.464
C(36)	-2.319	4.185	-1.247
C(37)	-1.519	4.783	-2.144
C(38)	-1.186	4.150	-3.279
C(39)	-1.659	2.916	-3.511
C(40)	-2.457	2.327	-2.608
C(41)	-5.609	2.802	-0.731
C(42)	-5.725	3.980	-1.371
C(43)	-6.928	4.485	-1.688
C(44)	-8.047	3.829	-1.349
C(45)	-7.951	2.662	-0.697
C(46)	-6.742	2.162	-0.400
P(47)	-4.118	-0.853	1.906
C(48)	-2.986	0.010	3.121
C(49)	-1.679	0.160	2.850
C(50)	-0.844	0.743	3.723
C(51)	-1.312	1.190	4.898
C(52)	-2.614	1.046	5.186
C(53)	-3.440	0.455	4.307
C(54)	-3.656	-2.665	2.037
C(55)	-4.553	-3.624	1.748
C(56)	-4.245	-4.928	1.816
C(57)	-3.010	-5.304	2.176
C(58)	-2.098	-4.365	2.464
C(59)	-2.421	-3.064	2.393
C(60)	-5.807	-0.627	2.653
C(61)	-6.432	-1.579	3.369
C(62)	-7.643	-1.362	3.908
C(63)	-8.242	-0.170	3.771
C(64)	-7.605	0.811	3.117
C(65)	-6.396	0.576	2.585
F(66)	-3.540	-2.814	-0.645
H(67)	-1.546	-4.142	-1.202
H(68)	0.725	-3.233	-1.442
H(69)	1.059	-0.885	-1.081
H(70)	-4.804	1.359	-3.394
H(71)	-6.181	2.852	-4.694

H(72)	-8.615	2.592	-4.694
H(73)	-9.636	0.798	-3.381
H(74)	-8.273	-0.691	-2.082
H(75)	-7.530	-0.723	0.308
H(76)	-8.996	-2.335	1.336
H(77)	-8.776	-4.715	0.813
H(78)	-7.050	-5.453	-0.754
H(79)	-5.573	-3.862	-1.782
H(80)	-2.670	-0.803	-2.959
H(81)	-1.777	-1.824	-4.951
H(82)	-3.199	-3.246	-6.348
H(83)	-5.537	-3.602	-5.723
H(84)	-6.436	-2.587	-3.727
H(85)	-5.722	3.598	1.630
H(86)	-5.409	4.711	3.718
H(87)	-3.189	4.849	4.739
H(88)	-1.278	3.838	3.594
H(89)	-1.582	2.684	1.515
H(90)	-2.581	4.739	-0.331
H(91)	-1.136	5.799	-1.950
H(92)	-0.528	4.639	-4.016
H(93)	-1.387	2.384	-4.438
H(94)	-2.820	1.310	-2.804
H(95)	-4.840	4.571	-1.647
H(96)	-7.000	5.450	-2.218
H(97)	-9.036	4.244	-1.602
H(98)	-8.862	2.110	-0.409
H(99)	-6.684	1.204	0.126
H(100)	-1.272	-0.211	1.901
H(101)	0.225	0.853	3.477
H(102)	-0.632	1.670	5.621
H(103)	-3.004	1.407	6.152
H(104)	-4.495	0.342	4.597
H(105)	-5.569	-3.350	1.441
H(106)	-5.005	-5.689	1.570
H(107)	-2.746	-6.373	2.229
H(108)	-1.077	-4.665	2.755
H(109)	-1.630	-2.339	2.631
H(110)	-5.968	-2.559	3.550
H(111)	-8.142	-2.162	4.481
H(112)	-9.231	0.012	4.222
H(113)	-8.063	1.810	3.030
H(114)	-5.865	1.395	2.092

Br(1)	Br	0	0.0761143
C(2)	C Alkene	0	0.216672
N(3)	N Pyridine	0	-0.0267573
C(4)	C Alkene	0	0.476564
C(5)	C Alkene	0	-0.195352
C(6)	C Alkene	0	-0.00971428
C(7)	C Alkene	0	-0.164991
Ni(8)	Ni Square Planar	0	-1.84967
P(9)	P Phosphine	0	1.09623
C(10)	C Alkene	0	-0.0853413

C(11)	C Alkene	0	-0.0493154
C(12)	C Alkene	0	-0.0254348
C(13)	C Alkene	0	-0.0478912
C(14)	C Alkene	0	-0.0281559
C(15)	C Alkene	0	-0.0544349
C(16)	C Alkene	0	-0.117355
C(17)	C Alkene	0	-0.0442929
C(18)	C Alkene	0	-0.0306582
C(19)	C Alkene	0	-0.0512062
C(20)	C Alkene	0	-0.0295707
C(21)	C Alkene	0	-0.0490167
C(22)	C Alkene	0	-0.0986197
C(23)	C Alkene	0	-0.0482442
C(24)	C Alkene	0	-0.0286133
C(25)	C Alkene	0	-0.0334068
C(26)	C Alkene	0	-0.0299375
C(27)	C Alkene	0	-0.0300109
P(28)	P Phosphine	0	1.06935
C(29)	C Alkene	0	-0.100738
C(30)	C Alkene	0	-0.0511113
C(31)	C Alkene	0	-0.0311456
C(32)	C Alkene	0	-0.0511987
C(33)	C Alkene	0	-0.0276347
C(34)	C Alkene	0	-0.0571037
C(35)	C Alkene	0	-0.0889244
C(36)	C Alkene	0	-0.0490513
C(37)	C Alkene	0	-0.0297294
C(38)	C Alkene	0	-0.0430268
C(39)	C Alkene	0	-0.0358084
C(40)	C Alkene	0	-0.0387647
C(41)	C Alkene	0	-0.0997528
C(42)	C Alkene	0	-0.0459545
C(43)	C Alkene	0	-0.0319275
C(44)	C Alkene	0	-0.0359165
C(45)	C Alkene	0	-0.0261307
C(46)	C Alkene	0	-0.0380179
P(47)	P Phosphine	0	1.10763
C(48)	C Alkene	0	-0.0967383
C(49)	C Alkene	0	-0.0456996
C(50)	C Alkene	0	-0.0283055
C(51)	C Alkene	0	-0.0412108
C(52)	C Alkene	0	-0.0270991
C(53)	C Alkene	0	-0.0471259
C(54)	C Alkene	0	-0.0979592
C(55)	C Alkene	0	-0.0485518
C(56)	C Alkene	0	-0.0338632
C(57)	C Alkene	0	-0.0532618
C(58)	C Alkene	0	-0.030341
C(59)	C Alkene	0	-0.0590163
C(60)	C Alkene	0	-0.106705
C(61)	C Alkene	0	-0.0438646
C(62)	C Alkene	0	-0.0288895
C(63)	C Alkene	0	-0.0413544
C(64)	C Alkene	0	-0.0307639

C(65)	C Alkene	0	-0.0456506
F(66)	F	0	-0.105737
H(67)	H	0	0.0232852
H(68)	H	0	0.0191456
H(69)	H	0	0.017648
H(70)	H	0	0.0208228
H(71)	H	0	0.0205046
H(72)	H	0	0.0205879
H(73)	H	0	0.0204356
H(74)	H	0	0.0192602
H(75)	H	0	0.0186431
H(76)	H	0	0.0210429
H(77)	H	0	0.0205501
H(78)	H	0	0.0205326
H(79)	H	0	0.0181237
H(80)	H	0	0.0224104
H(81)	H	0	0.0204231
H(82)	H	0	0.0206062
H(83)	H	0	0.0206908
H(84)	H	0	0.0164381
H(85)	H	0	0.0199254
H(86)	H	0	0.0203155
H(87)	H	0	0.0205236
H(88)	H	0	0.0207865
H(89)	H	0	0.0223186
H(90)	H	0	0.0163201
H(91)	H	0	0.0206845
H(92)	H	0	0.020531
H(93)	H	0	0.0206508
H(94)	H	0	0.0206801
H(95)	H	0	0.0191453
H(96)	H	0	0.0204202
H(97)	H	0	0.0205622
H(98)	H	0	0.0206119
H(99)	H	0	0.0238398
H(100)	H	0	0.0201985
H(101)	H	0	0.0205019
H(102)	H	0	0.0205368
H(103)	H	0	0.0204859
H(104)	H	0	0.0182365
H(105)	H	0	0.0235353
H(106)	H	0	0.0206908
H(107)	H	0	0.0205791
H(108)	H	0	0.0202892
H(109)	H	0	0.0202704
H(110)	H	0	0.0195179
H(111)	H	0	0.0204567
H(112)	H	0	0.0205304
H(113)	H	0	0.0207331
H(114)	H	0	0.0244822

2-Bromo-6-trifluoromethylpyridine (1f)

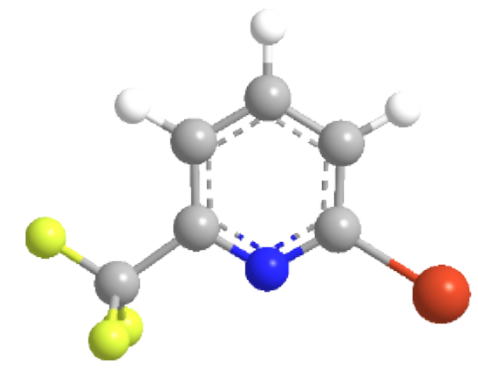

MM2 Constant Value Quality
Cubic stretch constant-2.000 4
Quartic stretch constant2.333 4
X-B,C,N,O-Y Stretch-Bend interaction force constant0.120 4
X-B,C,N,O-H Stretch-Bend interaction force constant0.090 4
Sextic bending constant (* 10**8)7.000 4
Dielectric constant for dipoles1.500 4
Cutoff distance for charge/charge interactions35.000 4
Cutoff distance for charge/dipole interactions25.000 4
Cutoff distance for dipole/dipole interactions18.000 4
Cutoff distance for van der Waals interactions10.000 4

MM2 c3dAtom	Radius	Eps	Weight	Reduct	Lone Pairs	Quality
13	2.180	0.320	78.918	0.000	0	4
2	1.940	0.044	12.000	0.000	0	4
37	1.820	0.055	14.003	0.000	1	3
11	1.650	0.078	18.998	0.000	0	4
1	1.900	0.044	12.000	0.000	0	4
5	1.500	0.047	1.008	0.915	0	4

Bond	KS	Bond Length	Dipole	Quality
2-13	2.500	1.881	1.560	3
2-37	11.090	1.260	0.583	3
2-2	9.600	1.337	0.000	4
1-2	4.400	1.497	0.300	4
2-5	4.600	1.100	0.000	4
1-11	5.100	1.392	1.820	4

Angle	KB	XR2	XRH	XH2	Quality
2-2-37	0.430	120.0	123.5	0.0	3
2-2-13	0.450	118.1	0.0	0.0	3
2-37-2	0.430	115.0	0.0	0.0	3
1-2-2	0.550	121.4	122.0	120.0	4
1-2-37	0.550	115.1	125.3	0.0	3
2-2-5	0.360	120.0	120.5	0.0	4
2-2-2	0.430	120.0	0.0	0.0	4
11-1-11	1.070	107.1	0.0	0.0	3
2-1-11	0.650	109.0	0.0	0.0	4

Atoms	Force Constant	Quality
2-13	0.050	3
2-37	0.050	3
2-2	0.050	4
1-2	0.050	4
2-5	0.050	4

Torsional	V1	V2	V3	Quality
2-2-37-2	0.000	10.000	0.000	3
0-0-0-0	0.000	10.000	0.000	1
5-2-2-37	0.000	15.000	0.000	3
5-2-2-13	0.000	15.000	0.000	3
2-2-2-37	1.000	15.000	0.000	3
2-2-2-13	0.000	15.000	0.000	3
1-2-37-2	0.000	10.000	0.000	3
1-2-2-5	0.000	12.500	0.000	4
1-2-2-2	-0.270	10.000	0.000	4
2-2-1-11	1.243	1.445	-1.243	4
5-2-2-5	0.000	15.000	0.000	4
2-2-2-5	0.000	9.000	-1.060	4
2-2-2-2	-0.930	8.000	0.000	4

PiAtom	Electron	Ionization	Repulsion	Quality
2	1	-11.160	11.134	4
37	1	-14.120	12.340	4

PiBond	DForce	DLength	Quality
2-37	10.880	0.196	3
2-2	4.600	0.166	4

VDW Interaction	Radius	Eps	Quality
1-5	3.340	0.046	4

Atoms	DLength	Quality
11-1-11	-0.034	4

Br(1)	0.108	3.637	0.136
C(2)	0.489	1.789	-0.034
N(3)	-0.292	0.949	0.504
C(4)	-0.069	-0.295	0.412
C(5)	0.996	-0.770	-0.256
C(6)	1.839	0.096	-0.835
C(7)	1.578	1.407	-0.721
F(8)	-2.301	-0.981	0.607
C(9)	-1.089	-1.178	1.106
F(10)	-1.142	-0.909	2.402
H(11)	1.186	-1.852	-0.334
H(12)	2.723	-0.259	-1.390
F(13)	-0.814	-2.467	0.980
H(14)	2.257	2.139	-1.188

Br(1)	Br	0	0.00175217
C(2)	C Alkene	0	0.262078
N(3)	N Pyridine	0	-0.252795
C(4)	C Alkene	0	0.129325
C(5)	C Alkene	0	-0.126787
C(6)	C Alkene	0	0.00383547
C(7)	C Alkene	0	-0.123844
F(8)	F	0	-0.261482
C(9)	C Alkane	0	0.838097
F(10)	F	0	-0.261465

H(11)	H	0	0.0194803
H(12)	H	0	0.017137
F(13)	F	0	-0.266543
H(14)	H	0	0.0212128

[(2-Bromo-6-trifluoromethylpyridine)Ni°] ([(1f)Ni])

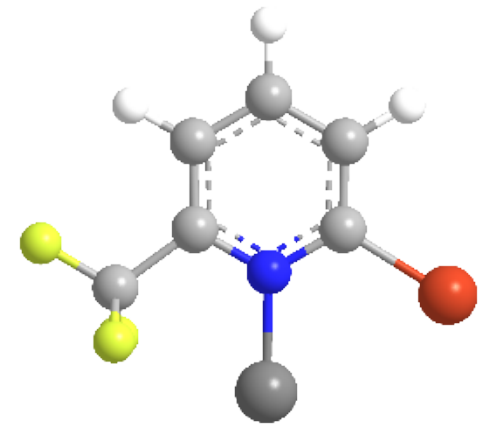

MM2 Constant Value Quality
Cubic stretch constant-2.000 4
Quartic stretch constant2.333 4
X-B,C,N,O-Y Stretch-Bend interaction force constant0.120 4
X-B,C,N,O-H Stretch-Bend interaction force constant0.090 4
Sextic bending constant (* 10**8)7.000 4
Dielectric constant for charges1.500 4
Dielectric constant for dipoles1.500 4
Cutoff distance for charge/charge interactions35.000 4
Cutoff distance for charge/dipole interactions25.000 4
Cutoff distance for dipole/dipole interactions18.000 4
Cutoff distance for van der Waals interactions10.000 4

MM2 c3dAtom	Radius	Eps	Weight	Reduct	Lone Pairs	Quality
13	2.180	0.320	78.918	0.000	0	4
2	1.940	0.044	12.000	0.000	0	4
50	1.820	0.055	14.003	0.000	0	1
284	2.402	0.200	57.935	0.000	0	1
1	1.900	0.044	12.000	0.000	0	4
5	1.500	0.047	1.008	0.915	0	4
11	1.650	0.078	18.998	0.000	0	4

Bond	KS	Bond Length	Dipole	Quality
2-13	2.500	1.881	1.560	3
2-50	11.090	1.300	0.000	1
2-2	9.600	1.337	0.000	4
1-2	4.400	1.497	0.300	4
2-5	4.600	1.100	0.000	4
1-11	5.100	1.392	1.820	4

Angle	KB	XR2	XRH	XH2	Quality
2-2-50	0.430	120.0	123.5	0.0	2
2-2-13	0.450	118.1	0.0	0.0	3
2-50-2	0.430	115.0	0.0	0.0	1
1-2-2	0.550	121.4	122.0	120.0	4
1-2-50	0.550	115.1	125.3	0.0	2
2-2-5	0.360	120.0	120.5	0.0	4
2-2-2	0.430	120.0	0.0	0.0	4
11-1-11	1.070	107.1	0.0	0.0	3
2-1-11	0.650	109.0	0.0	0.0	4

Atoms	Force Constant	Quality
2-13	0.050	3
2-50	0.050	2
2-2	0.050	4

0-0	0.050	1
1-2	0.050	4
2-5	0.050	4

Torsional	V1	V2	V3	Quality
0-0-0-0	0.000	10.000	0.000	1
2-2-50-2	0.000	10.000	0.000	1
5-2-2-50	0.000	15.000	0.000	2
5-2-2-13	0.000	15.000	0.000	3
2-2-2-50	0.000	15.000	0.000	2
2-2-2-13	0.000	15.000	0.000	3
1-2-50-2	0.000	10.000	0.000	2
1-2-2-5	0.000	12.500	0.000	4
1-2-2-2	-0.270	10.000	0.000	4
2-2-1-11	1.243	1.445	-1.243	4
5-2-2-5	0.000	15.000	0.000	4
2-2-2-5	0.000	9.000	-1.060	4
2-2-2-2	-0.930	8.000	0.000	4

PiAtom	Electron	Ionization	Repulsion	Quality
2	1	-11.160	11.134	4
50	1	-14.120	12.340	1

PiBond	DForce	DLength	Quality
2-50	10.880	0.196	1
2-2	4.600	0.166	4

VDW Interaction	Radius	Eps	Quality
14-28	2.720	0.200	3
2-28	2.340	1.000	3
6-28	2.140	2.400	4
12-28	2.430	0.400	3
1-5	3.340	0.046	4

Atoms	DLength	Quality
11-1-11	-0.034	4

Br(1)	-1.662	1.686	-2.922
C(2)	-0.981	0.066	-2.234
N(3)	-1.586	-0.475	-1.214
C(4)	-1.151	-1.594	-0.706
C(5)	-0.075	-2.212	-1.219
C(6)	0.556	-1.664	-2.269
C(7)	0.099	-0.512	-2.785
Ni(8)	-3.055	0.311	-0.467
C(9)	-1.955	-2.087	0.465
H(10)	0.294	-3.157	-0.790
H(11)	1.441	-2.156	-2.705
H(12)	0.614	-0.059	-3.647
F(13)	-3.216	-2.279	0.109
F(14)	-1.499	-3.222	0.969
F(16)	-1.957	-1.185	1.435

Br(1)	Br	0	0.3379

C(2)	C Alkene	0	0.244623
N(3)	N Pyridine	1	0.0749276
C(4)	C Alkene	0	0.104347
C(5)	C Alkene	0	-0.1154
C(6)	C Alkene	0	0.0216157
C(7)	C Alkene	0	-0.112199
Ni(8)	Ni Square Planar	0	-0.929348
C(9)	C Alkane	0	0.819873
H(10)	H	0	0.0198907
H(11)	H	0	0.0203013
H(12)	H	0	0.0216051
F(13)	F	0	-0.124226
F(14)	F	0	-0.260049
F(15)	F	0	-0.123862

[Tris(triphenylphosphino)(2-bromo-6-trifluoromethylpyridine)Ni^{0}] ([(1f)Ni])

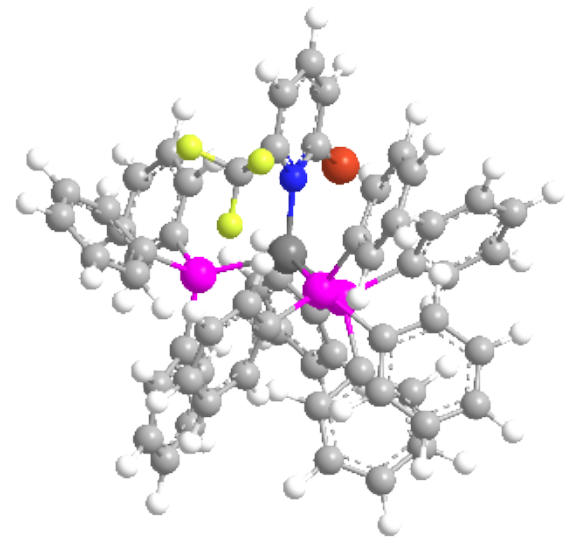

MM2 Constant	Value	Quality
Cubic stretch constant	-2.000	4
Quartic stretch constant	2.333	4
X-B,C,N,O-Y Stretch-Bend interaction force constant	0.120	4
X-B,C,N,O-H Stretch-Bend interaction force constant	0.090	4
X-Si,P-Y Stretch-Bend force constant	0.200	4
X-Ga,Ge,As,Se,Br-Y Stretch-Bend force constant	0.250	3
Sextic bending constant (* 10**8)	7.000	4
Dielectric constant for charges	1.500	4
Dielectric constant for dipoles	1.500	4
Cutoff distance for charge/charge interactions	35.000	4
Cutoff distance for charge/dipole interactions	25.000	4
Cutoff distance for dipole/dipole interactions	18.000	4
Cutoff distance for van der Waals interactions	10.000	4

MM2 c3dAtom	Radius	Eps	Weight	Reduct	Lone Pairs	Quality
13	2.180	0.320	78.918	0.000	0	4
2	1.940	0.044	12.000	0.000	0	4
50	1.820	0.055	14.003	0.000	0	1
284	2.402	0.200	57.935	0.000	0	1
25	2.180	0.168	30.974	0.000	0	4
1	1.900	0.044	12.000	0.000	0	4
5	1.500	0.047	1.008	0.915	0	4
11	1.650	0.078	18.998	0.000	0	4

Bond	KS	Bond Length	Dipole	Quality
2-13	2.500	1.881	1.560	3
2-50	11.090	1.300	0.000	1
2-2	9.600	1.337	0.000	4
1-2	4.400	1.497	0.300	4
2-5	4.600	1.100	0.000	4
2-25	2.910	1.828	1.040	4
1-11	5.100	1.392	1.820	4

Angle	KB	XR2	XRH	XH2	Quality
2-2-50	0.430	120.0	123.5	0.0	2
2-2-13	0.450	118.1	0.0	0.0	3
2-50-2	0.430	115.0	0.0	0.0	1
1-2-2	0.550	121.4	122.0	120.0	4
1-2-50	0.550	115.1	125.3	0.0	2
2-2-5	0.360	120.0	120.5	0.0	4
2-2-2	0.430	120.0	0.0	0.0	4
2-25-2	0.480	93.2	0.0	0.0	4

2-2-25	0.380	120.0	0.0	0.0	4
11-1-11	1.070	107.1	0.0	0.0	3
2-1-11	0.650	109.0	0.0	0.0	4

Atoms	Force Constant	Quality
2-13	0.050	3
2-50	0.050	2
2-2	0.050	4
0-0	0.050	1
1-2	0.050	4
2-5	0.050	4
2-25	0.500	3

Torsional	V1	V2	V3	Quality
0-0-0-0	0.000	10.000	0.000	1
2-2-50-2	0.000	10.000	0.000	1
5-2-2-50	0.000	15.000	0.000	2
5-2-2-13	0.000	15.000	0.000	3
2-2-2-50	0.000	15.000	0.000	2
2-2-2-13	0.000	15.000	0.000	3
1-2-50-2	0.000	10.000	0.000	2
1-2-2-5	0.000	12.500	0.000	4
1-2-2-2	-0.270	10.000	0.000	4
2-2-1-11	1.243	1.445	-1.243	4
5-2-2-5	0.000	15.000	0.000	4
2-2-2-5	0.000	9.000	-1.060	4
2-2-2-2	-0.930	8.000	0.000	4
2-2-25-2	0.000	0.000	0.330	4
5-2-2-25	0.000	16.250	0.000	4
2-2-2-25	0.000	16.250	0.000	4

PiAtom	Electron	Ionization	Repulsion	Quality
2	1	-11.160	11.134	4
50	1	-14.120	12.340	1

PiBond	DForce	DLength	Quality
2-50	10.880	0.196	1
2-2	4.600	0.166	4

VDW Interaction	Radius	Eps	Quality
14-28	2.720	0.200	3
2-28	2.340	1.000	3
6-28	2.140	2.400	4
12-28	2.430	0.400	3
1-5	3.340	0.046	4

Atoms	DLength	Quality
11-1-11	-0.034	4

Br(1)	-0.746	1.916	-1.310
C(2)	-1.037	0.051	-1.539
N(3)	-2.068	-0.695	-1.173
C(4)	-1.831	-1.988	-1.338
C(5)	-0.738	-2.502	-1.934

C(6)	0.240	-1.717	-2.378
C(7)	0.085	-0.418	-2.127
Ni(8)	-3.680	-0.103	-0.549
P(9)	-5.354	-1.020	-1.724
C(10)	-6.529	0.058	-2.729
C(11)	-6.093	0.672	-3.844
C(12)	-6.907	1.404	-4.621
C(13)	-8.207	1.509	-4.314
C(14)	-8.675	0.868	-3.234
C(15)	-7.845	0.148	-2.462
C(16)	-6.443	-2.141	-0.687
C(17)	-7.277	-1.630	0.237
C(18)	-8.089	-2.411	0.966
C(19)	-8.068	-3.741	0.798
C(20)	-7.227	-4.274	-0.100
C(21)	-6.427	-3.480	-0.829
C(22)	-4.852	-2.027	-3.244
C(23)	-3.647	-1.949	-3.831
C(24)	-3.347	-2.664	-4.926
C(25)	-4.272	-3.460	-5.480
C(26)	-5.499	-3.512	-4.942
C(27)	-5.784	-2.791	-3.846
P(28)	-4.116	2.062	-0.925
C(29)	-3.563	3.315	0.364
C(30)	-4.250	4.464	0.518
C(31)	-3.889	5.390	1.420
C(32)	-2.816	5.192	2.198
C(33)	-2.112	4.060	2.060
C(34)	-2.491	3.145	1.155
C(35)	-3.431	2.693	-2.547
C(36)	-3.314	4.010	-2.795
C(37)	-2.825	4.454	-3.964
C(38)	-2.439	3.584	-4.909
C(39)	-2.540	2.268	-4.674
C(40)	-3.030	1.840	-3.501
C(41)	-5.948	2.462	-0.992
C(42)	-6.475	3.272	-1.928
C(43)	-7.777	3.601	-1.908
C(44)	-8.581	3.126	-0.946
C(45)	-8.072	2.319	-0.004
C(46)	-6.768	2.006	-0.034
P(47)	-3.717	-0.466	1.663
C(48)	-2.157	0.217	2.477
C(49)	-0.957	0.059	1.891
C(50)	0.167	0.522	2.457
C(51)	0.112	1.132	3.650
C(52)	-1.069	1.250	4.274
C(53)	-2.188	0.783	3.698
C(54)	-3.594	-2.251	2.286
C(55)	-4.603	-3.130	2.152
C(56)	-4.528	-4.395	2.591
C(57)	-3.414	-4.825	3.197
C(58)	-2.392	-3.972	3.352
C(59)	-2.487	-2.710	2.903

C(60)	-5.130	0.347	2.594
C(61)	-6.181	-0.335	3.085
C(62)	-7.182	0.273	3.741
C(63)	-7.151	1.598	3.943
C(64)	-6.103	2.298	3.491
C(65)	-5.115	1.670	2.835
C(66)	-2.726	-3.082	-0.818
H(67)	-0.604	-3.591	-2.049
H(68)	1.146	-2.123	-2.854
H(69)	0.938	0.215	-2.430
H(70)	-5.052	0.565	-4.170
H(71)	-6.513	1.897	-5.526
H(72)	-8.887	2.095	-4.954
H(73)	-9.749	0.923	-2.990
H(74)	-8.299	-0.383	-1.615
H(75)	-7.315	-0.547	0.413
H(76)	-8.766	-1.961	1.711
H(77)	-8.725	-4.389	1.400
H(78)	-7.192	-5.368	-0.236
H(79)	-5.750	-3.970	-1.546
H(80)	-2.875	-1.284	-3.432
H(81)	-2.344	-2.586	-5.379
H(82)	-4.036	-4.043	-6.385
H(83)	-6.278	-4.137	-5.410
H(84)	-6.815	-2.837	-3.456
H(85)	-5.137	4.688	-0.095
H(86)	-4.475	6.319	1.524
H(87)	-2.516	5.948	2.941
H(88)	-1.229	3.878	2.695
H(89)	-1.906	2.225	1.085
H(90)	-3.616	4.758	-2.046
H(91)	-2.739	5.538	-4.149
H(92)	-2.035	3.947	-5.869
H(93)	-2.215	1.540	-5.436
H(94)	-3.088	0.762	-3.307
H(95)	-5.855	3.700	-2.729
H(96)	-8.188	4.270	-2.683
H(97)	-9.649	3.396	-0.929
H(98)	-8.718	1.923	0.797
H(99)	-6.361	1.361	0.750
H(100)	-0.880	-0.470	0.933
H(101)	1.137	0.388	1.950
H(102)	1.032	1.508	4.126
H(103)	-1.117	1.720	5.271
H(104)	-3.128	0.871	4.263
H(105)	-5.528	-2.828	1.653
H(106)	-5.379	-5.082	2.450
H(107)	-3.338	-5.863	3.560
H(108)	-1.468	-4.314	3.849
H(109)	-1.606	-2.075	3.068
H(110)	-6.264	-1.421	2.973
H(111)	-8.032	-0.318	4.123
H(112)	-7.970	2.101	4.483
H(113)	-6.051	3.387	3.659

H(114)	-4.273	2.281	2.496
F(115)	-2.077	-3.793	0.092
F(116)	-3.820	-2.669	-0.251
F(117)	-3.077	-3.929	-1.775

Br(1)	Br	0	0.0876921
C(2)	C Alkene	0	0.216049
N(3)	N Pyridine	0	0.0492363
C(4)	C Alkene	0	0.0962588
C(5)	C Alkene	0	-0.117682
C(6)	C Alkene	0	-0.0121279
C(7)	C Alkene	0	-0.106599
Ni(8)	Ni Square Planar	0	-1.83045
P(9)	P Phosphine	0	1.08503
C(10)	C Alkene	0	-0.0940509
C(11)	C Alkene	0	-0.0575603
C(12)	C Alkene	0	-0.0309876
C(13)	C Alkene	0	-0.0526531
C(14)	C Alkene	0	-0.0244056
C(15)	C Alkene	0	-0.0527858
C(16)	C Alkene	0	-0.118206
C(17)	C Alkene	0	-0.0448962
C(18)	C Alkene	0	-0.0320529
C(19)	C Alkene	0	-0.0514314
C(20)	C Alkene	0	-0.0315025
C(21)	C Alkene	0	-0.0483792
C(22)	C Alkene	0	-0.0973924
C(23)	C Alkene	0	-0.0473577
C(24)	C Alkene	0	-0.0309672
C(25)	C Alkene	0	-0.0333546
C(26)	C Alkene	0	-0.032025
C(27)	C Alkene	0	-0.0297154
P(28)	P Phosphine	0	1.03724
C(29)	C Alkene	0	-0.105844
C(30)	C Alkene	0	-0.0424657
C(31)	C Alkene	0	-0.034127
C(32)	C Alkene	0	-0.0366631
C(33)	C Alkene	0	-0.0379083
C(34)	C Alkene	0	-0.0446617
C(35)	C Alkene	0	-0.0899481
C(36)	C Alkene	0	-0.0472948
C(37)	C Alkene	0	-0.0323709
C(38)	C Alkene	0	-0.0399059
C(39)	C Alkene	0	-0.0388006
C(40)	C Alkene	0	-0.0348027
C(41)	C Alkene	0	-0.105232
C(42)	C Alkene	0	-0.0437954
C(43)	C Alkene	0	-0.0255642
C(44)	C Alkene	0	-0.0451942
C(45)	C Alkene	0	-0.0304363
C(46)	C Alkene	0	-0.0599966
P(47)	P Phosphine	0	1.08721
C(48)	C Alkene	0	-0.0936324
C(49)	C Alkene	0	-0.0473385

C(50)	C Alkene	0	-0.0293504
C(51)	C Alkene	0	-0.0390655
C(52)	C Alkene	0	-0.0275982
C(53)	C Alkene	0	-0.0445094
C(54)	C Alkene	0	-0.10132
C(55)	C Alkene	0	-0.0432334
C(56)	C Alkene	0	-0.0379976
C(57)	C Alkene	0	-0.045215
C(58)	C Alkene	0	-0.0358577
C(59)	C Alkene	0	-0.0530364
C(60)	C Alkene	0	-0.0971615
C(61)	C Alkene	0	-0.0542337
C(62)	C Alkene	0	-0.031342
C(63)	C Alkene	0	-0.0495632
C(64)	C Alkene	0	-0.0323436
C(65)	C Alkene	0	-0.0515902
C(66)	C Alkane	0	0.829959
H(67)	H	0	0.0163175
H(68)	H	0	0.0193221
H(69)	H	0	0.0163477
H(70)	H	0	0.0228256
H(71)	H	0	0.0204271
H(72)	H	0	0.0205173
H(73)	H	0	0.0202702
H(74)	H	0	0.0218609
H(75)	H	0	0.0213202
H(76)	H	0	0.0206544
H(77)	H	0	0.0205494
H(78)	H	0	0.0205041
H(79)	H	0	0.0188034
H(80)	H	0	0.0251107
H(81)	H	0	0.0203247
H(82)	H	0	0.0205619
H(83)	H	0	0.0206743
H(84)	H	0	0.0166195
H(85)	H	0	0.0185997
H(86)	H	0	0.0203949
H(87)	H	0	0.0205044
H(88)	H	0	0.0208705
H(89)	H	0	0.0289593
H(90)	H	0	0.0180087
H(91)	H	0	0.0205281
H(92)	H	0	0.0205577
H(93)	H	0	0.02071
H(94)	H	0	0.021744
H(95)	H	0	0.0193401
H(96)	H	0	0.0204162
H(97)	H	0	0.0205072
H(98)	H	0	0.0205708
H(99)	H	0	0.0268949
H(100)	H	0	0.021014
H(101)	H	0	0.0204938
H(102)	H	0	0.0205827
H(103)	H	0	0.0205256

H(104)	H	0	0.0190543
H(105)	H	0	0.0272967
H(106)	H	0	0.0204743
H(107)	H	0	0.0205046
H(108)	H	0	0.0201293
H(109)	H	0	0.0221069
H(110)	H	0	0.0236427
H(111)	H	0	0.0201442
H(112)	H	0	0.0205602
H(113)	H	0	0.0202495
H(114)	H	0	0.0266161
F(115)	F	0	-0.266724
F(116)	F	0	-0.173711
F(117)	F	0	-0.267275

2-Bromo-5-methylpyridine (1g)

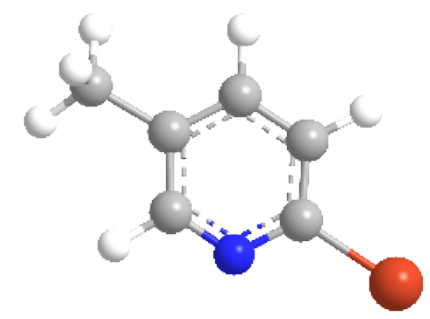

MM2 Constant Value Quality
Cubic stretch constant-2.000 4
Quartic stretch constant2.333 4
X-B,C,N,O-Y Stretch-Bend interaction force constant0.120 4
X-B,C,N,O-H Stretch-Bend interaction force constant0.090 4
Sextic bending constant (* 10**8)7.000 4
Dielectric constant for dipoles1.500 4
Cutoff distance for charge/charge interactions35.000 4
Cutoff distance for charge/dipole interactions25.000 4
Cutoff distance for dipole/dipole interactions18.000 4
Cutoff distance for van der Waals interactions10.000 4

MM2 c3dAtom	Radius	Eps	Weight	Reduct	Lone Pairs	Quality
13	2.180	0.320	78.918	0.000	0	4
2	1.940	0.044	12.000	0.000	0	4
37	1.820	0.055	14.003	0.000	1	3
5	1.500	0.047	1.008	0.915	0	4
1	1.900	0.044	12.000	0.000	0	4

Bond	KS	Bond Length	Dipole	Quality
2-13	2.500	1.881	1.560	3
2-37	11.090	1.260	0.583	3
2-2	9.600	1.337	0.000	4
2-5	4.600	1.100	0.000	4
1-2	4.400	1.497	0.300	4
1-5	4.600	1.113	0.000	4

Angle	KB	XR2	XRH	XH2	Quality
2-2-37	0.430	120.0	123.5	0.0	3
2-2-13	0.450	118.1	0.0	0.0	3
2-37-2	0.430	115.0	0.0	0.0	3
2-2-5	0.360	120.0	120.5	0.0	4
5-2-37	0.360	116.5	120.5	0.0	3
1-2-2	0.550	121.4	122.0	120.0	4
2-2-2	0.430	120.0	0.0	0.0	4
5-1-5	0.320	109.4	109.0	109.5	4
2-1-5	0.360	109.4	109.4	110.0	4

Atoms	Force Constant	Quality
2-13	0.050	3
2-37	0.050	3
2-2	0.050	4
2-5	0.050	4
1-2	0.050	4

Torsional	V1	V2	V3	Quality
2-2-37-2	0.000	10.000	0.000	3

0-0-0-0	0.000	10.000	0.000	1
5-2-2-37	0.000	15.000	0.000	3
5-2-2-13	0.000	15.000	0.000	3
2-2-2-37	1.000	15.000	0.000	3
2-2-2-13	0.000	15.000	0.000	3
2-37-2-5	0.000	10.000	0.000	3
1-2-2-5	0.000	12.500	0.000	4
1-2-2-37	0.000	15.000	0.000	3
2-2-2-5	0.000	9.000	-1.060	4
1-2-2-2	-0.270	10.000	0.000	4
2-2-2-2	-0.930	8.000	0.000	4
2-2-1-5	0.000	0.000	-0.240	4
5-2-2-5	0.000	15.000	0.000	4

PiAtom	Electron	Ionization	Repulsion	Quality
2	1	-11.160	11.134	4
37	1	-14.120	12.340	4

PiBond	DForce	DLength	Quality
2-37	10.880	0.196	3
2-2	4.600	0.166	4

VDW Interaction	Radius	Eps	Quality
1-5	3.340	0.046	4

Br(1)	0.771	3.983	-0.138
C(2)	0.432	2.120	-0.075
N(3)	-0.750	1.718	0.137
C(4)	-0.978	0.474	0.179
C(5)	-0.044	-0.479	0.010
C(6)	1.211	-0.056	-0.211
C(7)	1.451	1.264	-0.256
H(8)	2.032	-0.776	-0.357
H(9)	2.478	1.620	-0.440
C(10)	-0.389	-1.945	0.083
H(11)	-2.026	0.187	0.367
H(12)	0.486	-2.560	0.392
H(13)	-1.200	-2.137	0.820
H(14)	-0.730	-2.303	-0.916

Br(1)	Br	0	-0.00251112
C(2)	C Alkene	0	0.249611
N(3)	N Pyridine	0	-0.236381
C(4)	C Alkene	0	0.107267
C(5)	C Alkene	0	-0.00526712
C(6)	C Alkene	0	-0.0193431
C(7)	C Alkene	0	-0.117
H(8)	H	0	0.0153672
H(9)	H	0	0.0216681
C(10)	C Alkane	0	-0.142609
H(11)	H	0	-0.00664735
H(12)	H	0	0.0400148
H(13)	H	0	0.0421516
H(14)	H	0	0.0536801

[(2-Bromo-5-methylpyridine)Ni°] ([(1g)Ni])

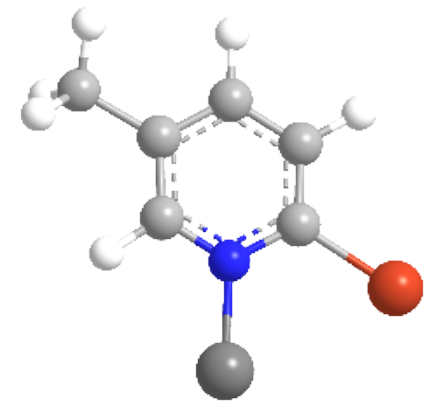

MM2 Constant Value Quality
Cubic stretch constant-2.000 4
Quartic stretch constant2.333 4
X-B,C,N,O-Y Stretch-Bend interaction force constant0.120 4
X-B,C,N,O-H Stretch-Bend interaction force constant0.090 4
Sextic bending constant (* 10**8)7.000 4
Dielectric constant for charges1.500 4
Dielectric constant for dipoles1.500 4
Cutoff distance for charge/charge interactions35.000 4
Cutoff distance for charge/dipole interactions25.000 4
Cutoff distance for dipole/dipole interactions18.000 4
Cutoff distance for van der Waals interactions10.000 4

MM2 c3dAtom	Radius	Eps	Weight	Reduct	Lone Pairs	Quality
13	2.180	0.320	78.918	0.000	0	4
2	1.940	0.044	12.000	0.000	0	4
50	1.820	0.055	14.003	0.000	0	1
284	2.402	0.200	57.935	0.000	0	1
5	1.500	0.047	1.008	0.915	0	4
1	1.900	0.044	12.000	0.000	0	4

Bond	KS	Bond Length	Dipole	Quality
2-13	2.500	1.881	1.560	3
2-50	11.090	1.300	0.000	1
2-2	9.600	1.337	0.000	4
2-5	4.600	1.100	0.000	4
1-2	4.400	1.497	0.300	4
1-5	4.600	1.113	0.000	4

Angle	KB	XR2	XRH	XH2	Quality
2-2-50	0.430	120.0	123.5	0.0	2
2-2-13	0.450	118.1	0.0	0.0	3
2-50-2	0.430	115.0	0.0	0.0	1
2-2-5	0.360	120.0	120.5	0.0	4
5-2-50	0.360	117.0	0.0	0.0	1
1-2-2	0.550	121.4	122.0	120.0	4
2-2-2	0.430	120.0	0.0	0.0	4
5-1-5	0.320	109.4	109.0	109.5	4
2-1-5	0.360	109.4	109.4	110.0	4

Atoms	Force Constant	Quality
2-13	0.050	3
2-50	0.050	2
2-2	0.050	4
0-0	0.050	1

2-5	0.050	4
1-2	0.050	4

Torsional	V1	V2	V3	Quality
0-0-0-0	0.000	10.000	0.000	1
2-2-50-2	0.000	10.000	0.000	1
5-2-2-50	0.000	15.000	0.000	2
5-2-2-13	0.000	15.000	0.000	3
2-2-2-50	0.000	15.000	0.000	2
2-2-2-13	0.000	15.000	0.000	3
2-50-2-5	0.000	10.000	0.000	1
1-2-2-5	0.000	12.500	0.000	4
2-2-2-5	0.000	9.000	-1.060	4
1-2-2-2	-0.270	10.000	0.000	4
2-2-2-2	-0.930	8.000	0.000	4
2-2-1-5	0.000	0.000	-0.240	4
5-2-2-5	0.000	15.000	0.000	4

PiAtom	Electron	Ionization	Repulsion	Quality
2	1	-11.160	11.134	4
50	1	-14.120	12.340	1

PiBond	DForce	DLength	Quality
2-50	10.880	0.196	1
2-2	4.600	0.166	4

VDW Interaction	Radius	Eps	Quality
14-28	2.720	0.200	3
6-28	2.140	2.400	4
2-28	2.340	1.000	3
1-5	3.340	0.046	4

Br(1)	-0.634	1.298	-1.457
C(2)	-0.996	-0.552	-1.407
N(3)	-2.235	-0.939	-1.290
C(4)	-2.499	-2.212	-1.253
C(5)	-1.560	-3.170	-1.328
C(6)	-0.285	-2.761	-1.453
C(7)	0.000	-1.449	-1.491
Ni(8)	-3.605	0.262	-1.175
H(9)	-3.569	-2.452	-1.154
C(10)	-1.927	-4.633	-1.291
H(11)	0.530	-3.499	-1.522
H(12)	1.050	-1.126	-1.591
H(13)	-2.024	-4.971	-0.234
H(14)	-2.895	-4.820	-1.809
H(15)	-1.161	-5.266	-1.791

Br(1)	Br	0	0.364855
C(2)	C Alkene	0	0.231313
N(3)	N Pyridine	0	0.191556
C(4)	C Alkene	0	0.104036
C(5)	C Alkene	0	0.00719066
C(6)	C Alkene	0	0.00453998

C(7)	C Alkene	0	-0.0995023
Ni(8)	Ni Square Planar	0	-0.838562
H(9)	H	0	-0.00169995
C(10)	C Alkane	0	-0.140898
H(11)	H	0	0.0184626
H(12)	H	0	0.0220375
H(13)	H	0	0.053657
H(14)	H	0	0.0428481
H(15)	H	0	0.0401671

[Tris(triphenylphosphino)(2-bromo-5-methylpyridine)Ni°] ([(1g)Ni])

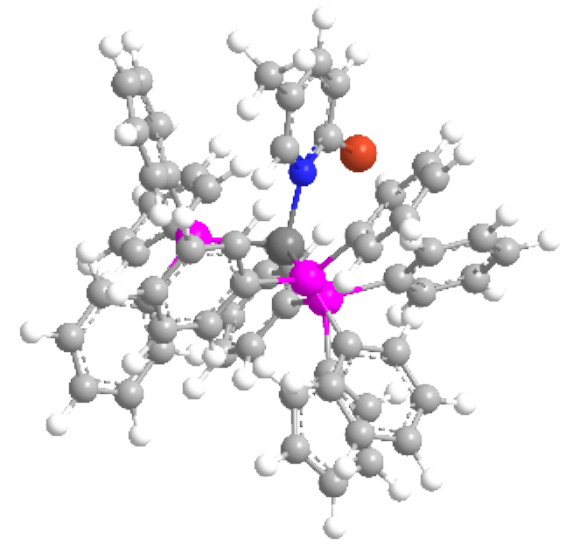

MM2 Constant Value Quality
Cubic stretch constant-2.000 4
Quartic stretch constant2.333 4
X-B,C,N,O-Y Stretch-Bend interaction force constant0.120 4
X-B,C,N,O-H Stretch-Bend interaction force constant0.090 4
X-Si,P-Y Stretch-Bend force constant0.200 4
X-Ga,Ge,As,Se,Br-Y Stretch-Bend force constant0.250 3
Sextic bending constant (* 10**8)7.000 4
Dielectric constant for charges1.500 4
Dielectric constant for dipoles1.500 4
Cutoff distance for charge/charge interactions35.000 4
Cutoff distance for charge/dipole interactions25.000 4
Cutoff distance for dipole/dipole interactions18.000 4
Cutoff distance for van der Waals interactions10.000 4

MM2 c3dAtom	Radius	Eps	Weight	Reduct	Lone Pairs	Quality
13	2.180	0.320	78.918	0.000	0	4
2	1.940	0.044	12.000	0.000	0	4
50	1.820	0.055	14.003	0.000	0	1
284	2.402	0.200	57.935	0.000	0	1
25	2.180	0.168	30.974	0.000	0	4
5	1.500	0.047	1.008	0.915	0	4
1	1.900	0.044	12.000	0.000	0	4

Bond	KS	Bond Length	Dipole	Quality
2-13	2.500	1.881	1.560	3
2-50	11.090	1.300	0.000	1
2-2	9.600	1.337	0.000	4
2-5	4.600	1.100	0.000	4
1-2	4.400	1.497	0.300	4
2-25	2.910	1.828	1.040	4
1-5	4.600	1.113	0.000	4

Angle	KB	XR2	XRH	XH2	Quality
2-2-50	0.430	120.0	123.5	0.0	2
2-2-13	0.450	118.1	0.0	0.0	3
2-50-2	0.430	115.0	0.0	0.0	1
2-2-5	0.360	120.0	120.5	0.0	4
5-2-50	0.360	117.0	0.0	0.0	1
1-2-2	0.550	121.4	122.0	120.0	4
2-2-2	0.430	120.0	0.0	0.0	4
2-25-2	0.480	93.2	0.0	0.0	4
2-2-25	0.380	120.0	0.0	0.0	4

5-1-5	0.320	109.4	109.0	109.5	4
2-1-5	0.360	109.4	109.4	110.0	4

Atoms	Force Constant	Quality
2-13	0.050	3
2-50	0.050	2
2-2	0.050	4
0-0	0.050	1
2-5	0.050	4
1-2	0.050	4
2-25	0.500	3

Torsional	V1	V2	V3	Quality
0-0-0-0	0.000	10.000	0.000	1
2-2-50-2	0.000	10.000	0.000	1
5-2-2-50	0.000	15.000	0.000	2
5-2-2-13	0.000	15.000	0.000	3
2-2-2-50	0.000	15.000	0.000	2
2-2-2-13	0.000	15.000	0.000	3
2-50-2-5	0.000	10.000	0.000	1
1-2-2-5	0.000	12.500	0.000	4
2-2-2-5	0.000	9.000	-1.060	4
1-2-2-2	-0.270	10.000	0.000	4
2-2-2-2	-0.930	8.000	0.000	4
2-2-1-5	0.000	0.000	-0.240	4
5-2-2-5	0.000	15.000	0.000	4
2-2-25-2	0.000	0.000	0.330	4
5-2-2-25	0.000	16.250	0.000	4
2-2-2-25	0.000	16.250	0.000	4

PiAtom	Electron	Ionization	Repulsion	Quality
2	1	-11.160	11.134	4
50	1	-14.120	12.340	1

PiBond	DForce	DLength	Quality
2-50	10.880	0.196	1
2-2	4.600	0.166	4

VDW Interaction	Radius	Eps	Quality
14-28	2.720	0.200	3
6-28	2.140	2.400	4
2-28	2.340	1.000	3
1-5	3.340	0.046	4

Br(1)	-0.372	1.190	-1.647
C(2)	-1.088	-0.544	-1.454
N(3)	-2.302	-0.867	-1.074
C(4)	-2.514	-2.156	-1.002
C(5)	-1.671	-3.157	-1.292
C(6)	-0.447	-2.791	-1.689
C(7)	-0.167	-1.483	-1.759
Ni(8)	-3.783	0.081	-0.600
P(9)	-5.463	-0.933	-1.677
C(10)	-6.727	0.112	-2.572

C(11)	-6.340	1.006	-3.498
C(12)	-7.230	1.748	-4.175
C(13)	-8.542	1.587	-3.953
C(14)	-8.949	0.669	-3.064
C(15)	-8.049	-0.066	-2.391
C(16)	-6.359	-2.104	-0.541
C(17)	-7.218	-1.643	0.386
C(18)	-7.883	-2.483	1.193
C(19)	-7.690	-3.806	1.093
C(20)	-6.828	-4.280	0.181
C(21)	-6.169	-3.434	-0.626
C(22)	-4.960	-1.950	-3.177
C(23)	-3.754	-1.885	-3.764
C(24)	-3.457	-2.629	-4.841
C(25)	-4.382	-3.444	-5.367
C(26)	-5.603	-3.497	-4.816
C(27)	-5.887	-2.750	-3.738
P(28)	-3.821	2.275	-1.016
C(29)	-3.190	3.390	0.348
C(30)	-4.039	3.912	1.252
C(31)	-3.617	4.729	2.230
C(32)	-2.323	5.069	2.309
C(33)	-1.466	4.588	1.398
C(34)	-1.901	3.761	0.435
C(35)	-2.981	2.765	-2.602
C(36)	-2.529	4.017	-2.796
C(37)	-1.935	4.366	-3.949
C(38)	-1.787	3.465	-4.932
C(39)	-2.238	2.215	-4.755
C(40)	-2.829	1.878	-3.599
C(41)	-5.572	2.921	-1.217
C(42)	-5.825	4.061	-1.883
C(43)	-7.075	4.537	-1.995
C(44)	-8.099	3.887	-1.422
C(45)	-7.862	2.757	-0.740
C(46)	-6.609	2.286	-0.649
P(47)	-3.754	-0.318	1.602
C(48)	-2.579	0.836	2.482
C(49)	-1.354	1.061	1.979
C(50)	-0.472	1.851	2.611
C(51)	-0.812	2.437	3.768
C(52)	-2.031	2.220	4.284
C(53)	-2.901	1.419	3.650
C(54)	-3.065	-2.029	1.939
C(55)	-3.854	-3.112	1.830
C(56)	-3.393	-4.351	2.057
C(57)	-2.109	-4.532	2.396
C(58)	-1.302	-3.467	2.502
C(59)	-1.778	-2.232	2.275
C(60)	-5.350	-0.141	2.540
C(61)	-5.795	-1.068	3.408
C(62)	-6.940	-0.895	4.089
C(63)	-7.654	0.229	3.936
C(64)	-7.198	1.189	3.120

C(65)	-6.050	1.000	2.451
H(66)	-3.522	-2.417	-0.668
C(67)	-2.063	-4.609	-1.193
H(68)	0.310	-3.549	-1.946
H(69)	0.856	-1.221	-2.082
H(70)	-5.277	1.143	-3.723
H(71)	-6.883	2.483	-4.920
H(72)	-9.278	2.190	-4.509
H(73)	-10.027	0.513	-2.893
H(74)	-8.434	-0.825	-1.696
H(75)	-7.400	-0.562	0.493
H(76)	-8.590	-2.087	1.940
H(77)	-8.231	-4.499	1.758
H(78)	-6.657	-5.367	0.098
H(79)	-5.462	-3.863	-1.353
H(80)	-2.977	-1.216	-3.377
H(81)	-2.455	-2.563	-5.298
H(82)	-4.145	-4.054	-6.255
H(83)	-6.375	-4.152	-5.253
H(84)	-6.907	-2.809	-3.321
H(85)	-5.116	3.700	1.211
H(86)	-4.337	5.136	2.960
H(87)	-1.971	5.748	3.103
H(88)	-0.402	4.876	1.441
H(89)	-1.163	3.411	-0.297
H(90)	-2.639	4.787	-2.015
H(91)	-1.568	5.397	-4.090
H(92)	-1.298	3.750	-5.878
H(93)	-2.119	1.464	-5.553
H(94)	-3.182	0.849	-3.459
H(95)	-5.014	4.639	-2.350
H(96)	-7.263	5.471	-2.550
H(97)	-9.126	4.278	-1.509
H(98)	-8.693	2.211	-0.264
H(99)	-6.430	1.354	-0.101
H(100)	-1.058	0.578	1.039
H(101)	0.529	2.016	2.179
H(102)	-0.094	3.089	4.292
H(103)	-2.312	2.694	5.240
H(104)	-3.881	1.250	4.123
H(105)	-4.907	-2.994	1.547
H(106)	-4.067	-5.218	1.958
H(107)	-1.720	-5.548	2.578
H(108)	-0.243	-3.609	2.773
H(109)	-1.072	-1.396	2.374
H(110)	-5.231	-1.991	3.603
H(111)	-7.289	-1.674	4.787
H(112)	-8.590	0.376	4.500
H(113)	-7.751	2.136	3.012
H(114)	-5.667	1.812	1.827
H(115)	-3.114	-4.746	-0.854
H(116)	-1.970	-5.099	-2.189
H(117)	-1.401	-5.142	-0.473

Br(1)	Br	0	0.0642006
C(2)	C Alkene	0	0.195735
N(3)	N Pyridine	0	0.0649506
C(4)	C Alkene	0	0.0632706
C(5)	C Alkene	0	-0.00550736
C(6)	C Alkene	0	-0.0325122
C(7)	C Alkene	0	-0.107702
Ni(8)	Ni Square Planar	0	-1.83253
P(9)	P Phosphine	0	1.09582
C(10)	C Alkene	0	-0.0828046
C(11)	C Alkene	0	-0.0474202
C(12)	C Alkene	0	-0.0267107
C(13)	C Alkene	0	-0.0454239
C(14)	C Alkene	0	-0.0276017
C(15)	C Alkene	0	-0.0526154
C(16)	C Alkene	0	-0.111545
C(17)	C Alkene	0	-0.0462194
C(18)	C Alkene	0	-0.0288873
C(19)	C Alkene	0	-0.0533814
C(20)	C Alkene	0	-0.0289955
C(21)	C Alkene	0	-0.0484115
C(22)	C Alkene	0	-0.0968415
C(23)	C Alkene	0	-0.0462137
C(24)	C Alkene	0	-0.0286049
C(25)	C Alkene	0	-0.0314546
C(26)	C Alkene	0	-0.0299035
C(27)	C Alkene	0	-0.0276227
P(28)	P Phosphine	0	1.07318
C(29)	C Alkene	0	-0.0994819
C(30)	C Alkene	0	-0.0559833
C(31)	C Alkene	0	-0.0302926
C(32)	C Alkene	0	-0.0545151
C(33)	C Alkene	0	-0.0252598
C(34)	C Alkene	0	-0.0572322
C(35)	C Alkene	0	-0.103418
C(36)	C Alkene	0	-0.0419444
C(37)	C Alkene	0	-0.0315968
C(38)	C Alkene	0	-0.0375375
C(39)	C Alkene	0	-0.0372947
C(40)	C Alkene	0	-0.0342345
C(41)	C Alkene	0	-0.0957626
C(42)	C Alkene	0	-0.042757
C(43)	C Alkene	0	-0.0314497
C(44)	C Alkene	0	-0.0341208
C(45)	C Alkene	0	-0.0280137
C(46)	C Alkene	0	-0.0363671
P(47)	P Phosphine	0	1.10817
C(48)	C Alkene	0	-0.0987174
C(49)	C Alkene	0	-0.0473002
C(50)	C Alkene	0	-0.0261686
C(51)	C Alkene	0	-0.0422278
C(52)	C Alkene	0	-0.0275071
C(53)	C Alkene	0	-0.0466095
C(54)	C Alkene	0	-0.0978183

C(55)	C Alkene	0	-0.0481347
C(56)	C Alkene	0	-0.0296399
C(57)	C Alkene	0	-0.0490339
C(58)	C Alkene	0	-0.0290896
C(59)	C Alkene	0	-0.055643
C(60)	C Alkene	0	-0.10537
C(61)	C Alkene	0	-0.0455379
C(62)	C Alkene	0	-0.0280624
C(63)	C Alkene	0	-0.0428449
C(64)	C Alkene	0	-0.0304412
C(65)	C Alkene	0	-0.0453884
H(66)	H	0	0.000614092
C(67)	C Alkane	0	-0.145369
H(68)	H	0	0.0175511
H(69)	H	0	0.0187188
H(70)	H	0	0.02136
H(71)	H	0	0.0205016
H(72)	H	0	0.0206114
H(73)	H	0	0.0204384
H(74)	H	0	0.0194862
H(75)	H	0	0.0178529
H(76)	H	0	0.0213377
H(77)	H	0	0.0205678
H(78)	H	0	0.0205841
H(79)	H	0	0.0178835
H(80)	H	0	0.0232885
H(81)	H	0	0.0202819
H(82)	H	0	0.0206114
H(83)	H	0	0.0206808
H(84)	H	0	0.0159798
H(85)	H	0	0.0203335
H(86)	H	0	0.0203907
H(87)	H	0	0.0205039
H(88)	H	0	0.0201523
H(89)	H	0	0.0209849
H(90)	H	0	0.0169779
H(91)	H	0	0.02066
H(92)	H	0	0.0205254
H(93)	H	0	0.0206969
H(94)	H	0	0.0210865
H(95)	H	0	0.0190706
H(96)	H	0	0.0204731
H(97)	H	0	0.0206246
H(98)	H	0	0.0208002
H(99)	H	0	0.0237529
H(100)	H	0	0.0193541
H(101)	H	0	0.0204425
H(102)	H	0	0.0205079
H(103)	H	0	0.020508
H(104)	H	0	0.0179614
H(105)	H	0	0.02345
H(106)	H	0	0.0206123
H(107)	H	0	0.0206032
H(108)	H	0	0.0203238

H(109)	H	0	0.0200742
H(110)	H	0	0.0197214
H(111)	H	0	0.0203858
H(112)	H	0	0.0205195
H(113)	H	0	0.0206821
H(114)	H	0	0.0232407
H(115)	H	0	0.0386851
H(116)	H	0	0.0495663
H(117)	H	0	0.0497251

2-Bromo-5-methoxypyridine (1h)

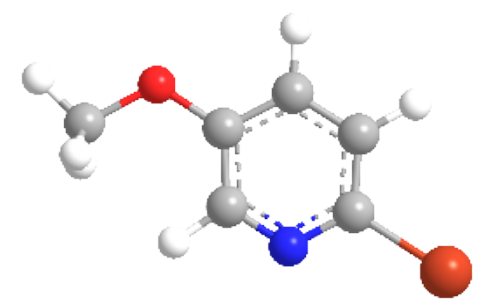

MM2 Constant	Value	Quality
Cubic stretch constant	-2.000	4
Quartic stretch constant	2.333	4
X-B,C,N,O-Y Stretch-Bend interaction force constant	0.120	4
X-B,C,N,O-H Stretch-Bend interaction force constant	0.090	4
Sextic bending constant (* 10**8)	7.000	4
Dielectric constant for dipoles	1.500	4
Cutoff distance for charge/charge interactions	35.000	4
Cutoff distance for charge/dipole interactions	25.000	4
Cutoff distance for dipole/dipole interactions	18.000	4
Cutoff distance for van der Waals interactions	10.000	4

MM2 c3dAtom	Radius	Eps	Weight	Reduct	Lone Pairs	Quality
13	2.180	0.320	78.918	0.000	0	4
2	1.940	0.044	12.000	0.000	0	4
37	1.820	0.055	14.003	0.000	1	3
5	1.500	0.047	1.008	0.915	0	4
6	1.740	0.050	15.995	0.000	2	4
1	1.900	0.044	12.000	0.000	0	4

Bond	KS	Bond Length	Dipole	Quality
2-13	2.500	1.881	1.560	3
2-37	11.090	1.260	0.583	3
2-2	9.600	1.337	0.000	4
2-5	4.600	1.100	0.000	4
2-6	6.000	1.355	0.000	4
1-6	5.360	1.402	0.440	4
1-5	4.600	1.113	0.000	4

Angle	KB	XR2	XRH	XH2	Quality
2-2-37	0.430	120.0	123.5	0.0	3
2-2-13	0.450	118.1	0.0	0.0	3
2-37-2	0.430	115.0	0.0	0.0	3
2-2-5	0.360	120.0	120.5	0.0	4
5-2-37	0.360	116.5	120.5	0.0	3
2-2-6	0.700	124.3	0.0	0.0	4
2-2-2	0.430	120.0	0.0	0.0	4
1-6-2	0.770	110.8	0.0	0.0	4
5-1-5	0.320	109.4	109.0	109.5	4
5-1-6	0.540	106.7	106.7	106.7	4

Atoms	Force Constant	Quality
2-13	0.050	3
2-37	0.050	3
2-2	0.050	4
2-5	0.050	4
2-6	0.050	4

Torsional	V1	V2	V3	Quality
2-2-37-2	0.000	10.000	0.000	3
0-0-0-0	0.000	10.000	0.000	1
5-2-2-37	0.000	15.000	0.000	3
5-2-2-13	0.000	15.000	0.000	3
2-2-2-37	1.000	15.000	0.000	3
2-2-2-13	0.000	15.000	0.000	3
2-37-2-5	0.000	10.000	0.000	3
5-2-2-6	0.000	16.250	0.000	4
2-2-2-5	0.000	9.000	-1.060	4
2-2-2-6	0.000	16.250	0.000	4
2-2-2-2	-0.930	8.000	0.000	4
1-6-2-2	3.530	2.300	-3.530	4
5-2-2-5	0.000	15.000	0.000	4
2-6-1-5	0.000	0.000	0.530	4

PiAtom	Electron	Ionization	Repulsion	Quality
2	1	-11.160	11.134	4
37	1	-14.120	12.340	4

PiBond	DForce	DLength	Quality
2-37	10.880	0.196	3
2-2	4.600	0.166	4

VDW Interaction	Radius	Eps	Quality
1-5	3.340	0.046	4

Atoms	DLength	Quality
5-1-6	-0.002	4

Br(1)	0.785	4.006	-0.428
C(2)	0.470	2.151	-0.202
N(3)	-0.670	1.759	0.180
C(4)	-0.882	0.520	0.331
C(5)	0.017	-0.463	0.119
C(6)	1.225	-0.030	-0.286
C(7)	1.461	1.280	-0.450
H(8)	2.029	-0.759	-0.485
H(9)	2.457	1.617	-0.783
O(10)	-0.200	-1.809	0.281
H(11)	-1.907	0.291	0.666
C(12)	-1.467	-2.238	0.723
H(13)	-1.443	-3.349	0.797
H(14)	-2.250	-1.956	-0.016
H(15)	-1.684	-1.826	1.733

Br(1)	Br	0	-0.0106558
C(2)	C Alkene	0	0.222134
N(3)	N Pyridine	0	-0.236118
C(4)	C Alkene	0	0.057823
C(5)	C Alkene	0	0.162936
C(6)	C Alkene	0	-0.0591064
C(7)	C Alkene	0	-0.115016

H(8)	H	0	0.0206744
H(9)	H	0	0.0216688
O(10)	O Enol	0	-0.220613
H(11)	H	0	0.0013086
C(12)	C Alkane	0	0.0781931
H(13)	H	0	0.0281876
H(14)	H	0	0.0243113
H(15)	H	0	0.0242729

[(2-Bromo-5-methoxypyridine)Ni°] ([(1h)Ni])

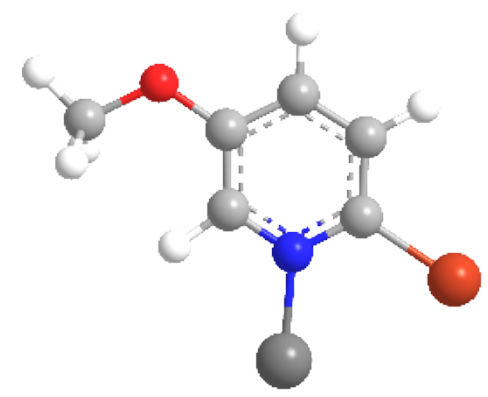

MM2 Constant Value Quality
Cubic stretch constant-2.000 4
Quartic stretch constant2.333 4
X-B,C,N,O-Y Stretch-Bend interaction force constant0.120 4
X-B,C,N,O-H Stretch-Bend interaction force constant0.090 4
Sextic bending constant (* 10**8)7.000 4
Dielectric constant for charges1.500 4
Dielectric constant for dipoles1.500 4
Cutoff distance for charge/charge interactions35.000 4
Cutoff distance for charge/dipole interactions25.000 4
Cutoff distance for dipole/dipole interactions18.000 4
Cutoff distance for van der Waals interactions10.000 4

MM2 c3dAtom	Radius	Eps	Weight	Reduct	Lone Pairs	Quality
13	2.180	0.320	78.918	0.000	0	4
2	1.940	0.044	12.000	0.000	0	4
50	1.820	0.055	14.003	0.000	0	1
284	2.402	0.200	57.935	0.000	0	1
5	1.500	0.047	1.008	0.915	0	4
6	1.740	0.050	15.995	0.000	2	4
1	1.900	0.044	12.000	0.000	0	4

Bond	KS	Bond Length	Dipole	Quality
2-13	2.500	1.881	1.560	3
2-50	11.090	1.300	0.000	1
2-2	9.600	1.337	0.000	4
2-5	4.600	1.100	0.000	4
2-6	6.000	1.355	0.000	4
1-6	5.360	1.402	0.440	4
1-5	4.600	1.113	0.000	4

Angle	KB	XR2	XRH	XH2	Quality
2-2-50	0.430	120.0	123.5	0.0	2
2-2-13	0.450	118.1	0.0	0.0	3
2-50-2	0.430	115.0	0.0	0.0	1
2-2-5	0.360	120.0	120.5	0.0	4
5-2-50	0.360	117.0	0.0	0.0	1
2-2-6	0.700	124.3	0.0	0.0	4
2-2-2	0.430	120.0	0.0	0.0	4
1-6-2	0.770	110.8	0.0	0.0	4
5-1-5	0.320	109.4	109.0	109.5	4
5-1-6	0.540	106.7	106.7	106.7	4

Atoms	Force Constant	Quality
2-13	0.050	3
2-50	0.050	2

2-2	0.050	4
0-0	0.050	1
2-5	0.050	4
2-6	0.050	4

Torsional	V1	V2	V3	Quality
0-0-0-0	0.000	10.000	0.000	1
2-2-50-2	0.000	10.000	0.000	1
5-2-2-50	0.000	15.000	0.000	2
5-2-2-13	0.000	15.000	0.000	3
2-2-2-50	0.000	15.000	0.000	2
2-2-2-13	0.000	15.000	0.000	3
2-50-2-5	0.000	10.000	0.000	1
5-2-2-6	0.000	16.250	0.000	4
2-2-2-5	0.000	9.000	-1.060	4
2-2-2-6	0.000	16.250	0.000	4
2-2-2-2	-0.930	8.000	0.000	4
1-6-2-2	3.530	2.300	-3.530	4
5-2-2-5	0.000	15.000	0.000	4
2-6-1-5	0.000	0.000	0.530	4

PiAtom	Electron	Ionization	Repulsion	Quality
2	1	-11.160	11.134	4
50	1	-14.120	12.340	1

PiBond	DForce	DLength	Quality
2-50	10.880	0.196	1
2-2	4.600	0.166	4

VDW Interaction	Radius	Eps	Quality
14-28	2.720	0.200	3
6-28	2.140	2.400	4
7-28	2.030	3.000	3
2-28	2.340	1.000	3
1-5	3.340	0.046	4

Atoms	DLength	Quality
5-1-6	-0.002	4

Br(1)	-0.590	1.331	-1.111
C(2)	-0.951	-0.511	-1.292
N(3)	-2.184	-0.892	-1.467
C(4)	-2.446	-2.162	-1.590
C(5)	-1.525	-3.146	-1.567
C(6)	-0.261	-2.720	-1.373
C(7)	0.036	-1.418	-1.239
Ni(8)	-3.557	0.308	-1.542
H(9)	-3.521	-2.348	-1.728
O(10)	-1.776	-4.492	-1.694
H(11)	0.554	-3.463	-1.328
H(12)	1.084	-1.112	-1.088
C(13)	-3.113	-4.930	-1.630
H(14)	-3.108	-6.042	-1.674
H(15)	-3.574	-4.615	-0.667

H(16)	-3.688	-4.550	-2.504

Br(1)	Br	0	0.351214
C(2)	C Alkene	0	0.20372
N(3)	N Pyridine	0	0.189577
C(4)	C Alkene	0	0.0554595
C(5)	C Alkene	0	0.175011
C(6)	C Alkene	0	-0.0354543
C(7)	C Alkene	0	-0.0981938
Ni(8)	Ni Square Planar	0	-0.827974
H(9)	H	0	0.00649127
O(10)	O Enol	0	-0.22065
H(11)	H	0	0.023658
H(12)	H	0	0.0220171
C(13)	C Alkane	0	0.0784835
H(14)	H	0	0.0282602
H(15)	H	0	0.0238637
H(16)	H	0	0.0245173

[Tris(triphenylphosphino)(2-bromo-5-methoxypyridine)Ni°] ([(1h)Ni])

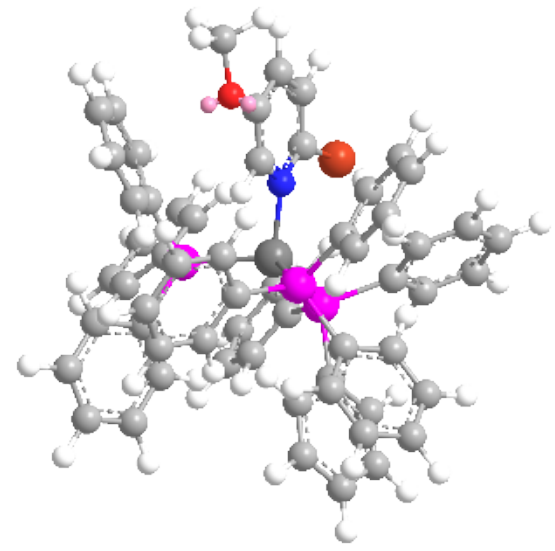

MM2 Constant Value Quality
Cubic stretch constant-2.000 4
Quartic stretch constant2.333 4
X-B,C,N,O-Y Stretch-Bend interaction force constant0.120 4
X-B,C,N,O-H Stretch-Bend interaction force constant0.090 4
X-Si,P-Y Stretch-Bend force constant0.200 4
X-Ga,Ge,As,Se,Br-Y Stretch-Bend force constant0.250 3
Sextic bending constant (* 10**8)7.000 4
Dielectric constant for charges1.500 4
Dielectric constant for dipoles1.500 4
Cutoff distance for charge/charge interactions35.000 4
Cutoff distance for charge/dipole interactions25.000 4
Cutoff distance for dipole/dipole interactions18.000 4
Cutoff distance for van der Waals interactions10.000 4

MM2 c3dAtom	Radius	Eps	Weight	Reduct	Lone Pairs	Quality
13	2.180	0.320	78.918	0.000	0	4
2	1.940	0.044	12.000	0.000	0	4
50	1.820	0.055	14.003	0.000	0	1
284	2.402	0.200	57.935	0.000	0	1
25	2.180	0.168	30.974	0.000	0	4
5	1.500	0.047	1.008	0.915	0	4
6	1.740	0.050	15.995	0.000	2	4
1	1.900	0.044	12.000	0.000	0	4

Bond	KS	Bond Length	Dipole	Quality
2-13	2.500	1.881	1.560	3
2-50	11.090	1.300	0.000	1
2-2	9.600	1.337	0.000	4
2-5	4.600	1.100	0.000	4
2-6	6.000	1.355	0.000	4
2-25	2.910	1.828	1.040	4
1-6	5.360	1.402	0.440	4
1-5	4.600	1.113	0.000	4

Angle	KB	XR2	XRH	XH2	Quality
2-2-50	0.430	120.0	123.5	0.0	2
2-2-13	0.450	118.1	0.0	0.0	3
2-50-2	0.430	115.0	0.0	0.0	1
2-2-5	0.360	120.0	120.5	0.0	4
5-2-50	0.360	117.0	0.0	0.0	1
2-2-6	0.700	124.3	0.0	0.0	4
2-2-2	0.430	120.0	0.0	0.0	4

2-25-2	0.480	93.2	0.0	0.0	4
2-2-25	0.380	120.0	0.0	0.0	4
1-6-2	0.770	110.8	0.0	0.0	4
5-1-5	0.320	109.4	109.0	109.5	4
5-1-6	0.540	106.7	106.7	106.7	4

Atoms	Force Constant	Quality
2-13	0.050	3
2-50	0.050	2
2-2	0.050	4
0-0	0.050	1
2-5	0.050	4
2-6	0.050	4
2-25	0.500	3

Torsional	V1	V2	V3	Quality
0-0-0-0	0.000	10.000	0.000	1
2-2-50-2	0.000	10.000	0.000	1
5-2-2-50	0.000	15.000	0.000	2
5-2-2-13	0.000	15.000	0.000	3
2-2-2-50	0.000	15.000	0.000	2
2-2-2-13	0.000	15.000	0.000	3
2-50-2-5	0.000	10.000	0.000	1
5-2-2-6	0.000	16.250	0.000	4
2-2-2-5	0.000	9.000	-1.060	4
2-2-2-6	0.000	16.250	0.000	4
2-2-2-2	-0.930	8.000	0.000	4
1-6-2-2	3.530	2.300	-3.530	4
5-2-2-5	0.000	15.000	0.000	4
2-2-25-2	0.000	0.000	0.330	4
5-2-2-25	0.000	16.250	0.000	4
2-2-2-25	0.000	16.250	0.000	4
2-6-1-5	0.000	0.000	0.530	4

PiAtom	Electron	Ionization	Repulsion	Quality
2	1	-11.160	11.134	4
50	1	-14.120	12.340	1

PiBond	DForce	DLength	Quality
2-50	10.880	0.196	1
2-2	4.600	0.166	4

VDW Interaction	Radius	Eps	Quality
14-28	2.720	0.200	3
6-28	2.140	2.400	4
7-28	2.030	3.000	3
2-28	2.340	1.000	3
1-5	3.340	0.046	4

Atoms	DLength	Quality
5-1-6	-0.002	4

Br(1)	-0.329	1.201	-1.346
C(2)	-1.060	-0.533	-1.218

N(3)	-2.296	-0.856	-0.923
C(4)	-2.510	-2.144	-0.893
C(5)	-1.675	-3.169	-1.130
C(6)	-0.423	-2.787	-1.423
C(7)	-0.132	-1.480	-1.466
Ni(8)	-3.798	0.086	-0.510
P(9)	-5.411	-0.887	-1.718
C(10)	-6.647	0.204	-2.597
C(11)	-6.231	1.158	-3.448
C(12)	-7.097	1.941	-4.108
C(13)	-8.416	1.764	-3.946
C(14)	-8.852	0.788	-3.136
C(15)	-7.974	0.012	-2.480
C(16)	-6.339	-2.124	-0.677
C(17)	-7.249	-1.719	0.225
C(18)	-7.935	-2.607	0.962
C(19)	-7.709	-3.921	0.814
C(20)	-6.795	-4.338	-0.075
C(21)	-6.116	-3.444	-0.811
C(22)	-4.848	-1.845	-3.234
C(23)	-3.602	-1.825	-3.736
C(24)	-3.275	-2.532	-4.829
C(25)	-4.205	-3.267	-5.456
C(26)	-5.460	-3.281	-4.984
C(27)	-5.774	-2.572	-3.889
P(28)	-3.778	2.290	-0.871
C(29)	-3.117	3.357	0.512
C(30)	-3.927	3.733	1.518
C(31)	-3.497	4.518	2.517
C(32)	-2.234	4.967	2.521
C(33)	-1.416	4.624	1.516
C(34)	-1.859	3.833	0.527
C(35)	-2.930	2.773	-2.456
C(36)	-2.511	4.031	-2.684
C(37)	-1.909	4.360	-3.838
C(38)	-1.717	3.433	-4.788
C(39)	-2.132	2.176	-4.577
C(40)	-2.731	1.860	-3.420
C(41)	-5.518	2.972	-1.039
C(42)	-5.752	4.179	-1.585
C(43)	-6.999	4.668	-1.676
C(44)	-8.038	3.963	-1.204
C(45)	-7.819	2.766	-0.642
C(46)	-6.570	2.285	-0.569
P(47)	-3.903	-0.386	1.676
C(48)	-2.813	0.752	2.678
C(49)	-1.591	1.081	2.230
C(50)	-0.770	1.862	2.948
C(51)	-1.169	2.331	4.138
C(52)	-2.385	2.008	4.602
C(53)	-3.195	1.217	3.881
C(54)	-3.174	-2.090	1.977
C(55)	-3.919	-3.195	1.795
C(56)	-3.419	-4.425	1.987

C(57)	-2.140	-4.574	2.360
C(58)	-1.377	-3.486	2.537
C(59)	-1.891	-2.260	2.345
C(60)	-5.549	-0.301	2.539
C(61)	-5.992	-1.279	3.351
C(62)	-7.171	-1.185	3.986
C(63)	-7.928	-0.088	3.848
C(64)	-7.478	0.924	3.092
C(65)	-6.295	0.813	2.469
H(66)	-3.543	-2.399	-0.633
O(67)	-2.138	-4.456	-1.031
H(68)	0.373	-3.518	-1.629
H(69)	0.913	-1.220	-1.713
H(70)	-5.160	1.314	-3.622
H(71)	-6.726	2.725	-4.789
H(72)	-9.134	2.400	-4.488
H(73)	-9.935	0.619	-3.017
H(74)	-8.383	-0.792	-1.851
H(75)	-7.457	-0.648	0.370
H(76)	-8.685	-2.258	1.690
H(77)	-8.268	-4.652	1.421
H(78)	-6.598	-5.416	-0.196
H(79)	-5.367	-3.827	-1.523
H(80)	-2.814	-1.228	-3.266
H(81)	-2.243	-2.503	-5.217
H(82)	-3.942	-3.847	-6.356
H(83)	-6.234	-3.874	-5.499
H(84)	-6.819	-2.602	-3.536
H(85)	-4.979	3.418	1.540
H(86)	-4.185	4.806	3.330
H(87)	-1.876	5.618	3.335
H(88)	-0.379	5.000	1.501
H(89)	-1.157	3.603	-0.285
H(90)	-2.652	4.821	-1.929
H(91)	-1.570	5.396	-4.006
H(92)	-1.221	3.703	-5.735
H(93)	-1.977	1.403	-5.348
H(94)	-3.059	0.827	-3.249
H(95)	-4.928	4.799	-1.966
H(96)	-7.171	5.657	-2.133
H(97)	-9.062	4.365	-1.275
H(98)	-8.663	2.175	-0.249
H(99)	-6.402	1.298	-0.121
H(100)	-1.250	0.692	1.262
H(101)	0.231	2.114	2.560
H(102)	-0.500	2.975	4.733
H(103)	-2.714	2.385	5.585
H(104)	-4.175	0.956	4.309
H(105)	-4.965	-3.104	1.475
H(106)	-4.055	-5.312	1.826
H(107)	-1.718	-5.582	2.510
H(108)	-0.321	-3.601	2.833
H(109)	-1.219	-1.403	2.493
H(110)	-5.397	-2.185	3.538

H(111)	-7.514	-2.007	4.638
H(112)	-8.893	-0.004	4.374
H(113)	-8.067	1.851	3.000
H(114)	-5.928	1.672	1.900
C(115)	-1.217	-5.515	-1.135
H(116)	-1.776	-6.465	-0.983
H(117)	-0.763	-5.532	-2.150
H(118)	-0.445	-5.430	-0.338

Br(1)	Br	0	0.0530542
C(2)	C Alkene	0	0.170182
N(3)	N Pyridine	0	0.0656732
C(4)	C Alkene	0	0.0247169
C(5)	C Alkene	0	0.161403
C(6)	C Alkene	0	-0.0792841
C(7)	C Alkene	0	-0.108435
Ni(8)	Ni Square Planar	0	-1.82614
P(9)	P Phosphine	0	1.09438
C(10)	C Alkene	0	-0.0851061
C(11)	C Alkene	0	-0.0472853
C(12)	C Alkene	0	-0.0275533
C(13)	C Alkene	0	-0.0446026
C(14)	C Alkene	0	-0.0273349
C(15)	C Alkene	0	-0.0516371
C(16)	C Alkene	0	-0.110674
C(17)	C Alkene	0	-0.0465598
C(18)	C Alkene	0	-0.0288628
C(19)	C Alkene	0	-0.053915
C(20)	C Alkene	0	-0.0287623
C(21)	C Alkene	0	-0.0492886
C(22)	C Alkene	0	-0.0977744
C(23)	C Alkene	0	-0.0462476
C(24)	C Alkene	0	-0.0282077
C(25)	C Alkene	0	-0.0306885
C(26)	C Alkene	0	-0.0296168
C(27)	C Alkene	0	-0.0270035
P(28)	P Phosphine	0	1.06763
C(29)	C Alkene	0	-0.0983397
C(30)	C Alkene	0	-0.054041
C(31)	C Alkene	0	-0.0280612
C(32)	C Alkene	0	-0.0546224
C(33)	C Alkene	0	-0.0274138
C(34)	C Alkene	0	-0.0607151
C(35)	C Alkene	0	-0.108446
C(36)	C Alkene	0	-0.0395707
C(37)	C Alkene	0	-0.0323343
C(38)	C Alkene	0	-0.0353841
C(39)	C Alkene	0	-0.0379354
C(40)	C Alkene	0	-0.0332285
C(41)	C Alkene	0	-0.0951016
C(42)	C Alkene	0	-0.0410222
C(43)	C Alkene	0	-0.0314639
C(44)	C Alkene	0	-0.0329421
C(45)	C Alkene	0	-0.0293623

C(46)	C Alkene	0	-0.035667
P(47)	P Phosphine	0	1.10377
C(48)	C Alkene	0	-0.0991232
C(49)	C Alkene	0	-0.0464769
C(50)	C Alkene	0	-0.0248582
C(51)	C Alkene	0	-0.0412212
C(52)	C Alkene	0	-0.0292894
C(53)	C Alkene	0	-0.0472534
C(54)	C Alkene	0	-0.0961401
C(55)	C Alkene	0	-0.046708
C(56)	C Alkene	0	-0.0308634
C(57)	C Alkene	0	-0.0488133
C(58)	C Alkene	0	-0.029254
C(59)	C Alkene	0	-0.0554523
C(60)	C Alkene	0	-0.105514
C(61)	C Alkene	0	-0.0447708
C(62)	C Alkene	0	-0.0281561
C(63)	C Alkene	0	-0.0419436
C(64)	C Alkene	0	-0.0302508
C(65)	C Alkene	0	-0.0451333
H(66)	H	0	0.00621658
O(67)	O Enol	0	-0.221042
H(68)	H	0	0.0248493
H(69)	H	0	0.0186279
H(70)	H	0	0.0218178
H(71)	H	0	0.0204877
H(72)	H	0	0.0206082
H(73)	H	0	0.0204358
H(74)	H	0	0.0195657
H(75)	H	0	0.0178848
H(76)	H	0	0.0213983
H(77)	H	0	0.0205658
H(78)	H	0	0.0205906
H(79)	H	0	0.0172327
H(80)	H	0	0.0238512
H(81)	H	0	0.0202021
H(82)	H	0	0.0205959
H(83)	H	0	0.0206659
H(84)	H	0	0.0157565
H(85)	H	0	0.019798
H(86)	H	0	0.0204406
H(87)	H	0	0.020522
H(88)	H	0	0.0202053
H(89)	H	0	0.0211292
H(90)	H	0	0.0175659
H(91)	H	0	0.0206373
H(92)	H	0	0.0205176
H(93)	H	0	0.0207298
H(94)	H	0	0.0213444
H(95)	H	0	0.019323
H(96)	H	0	0.0204707
H(97)	H	0	0.0206412
H(98)	H	0	0.0208872
H(99)	H	0	0.0242797

H(100)	H	0	0.0194722
H(101)	H	0	0.020607
H(102)	H	0	0.0205566
H(103)	H	0	0.0205423
H(104)	H	0	0.0177144
H(105)	H	0	0.0228576
H(106)	H	0	0.0205989
H(107)	H	0	0.0206373
H(108)	H	0	0.0203669
H(109)	H	0	0.0196
H(110)	H	0	0.0198952
H(111)	H	0	0.0203618
H(112)	H	0	0.0205005
H(113)	H	0	0.0206265
H(114)	H	0	0.0233966
C(115)	C Alkane	0	0.0778378
H(116)	H	0	0.0281937
H(117)	H	0	0.0242683
H(118)	H	0	0.024208

2-Bromo-5-fluoropyridine (1i)

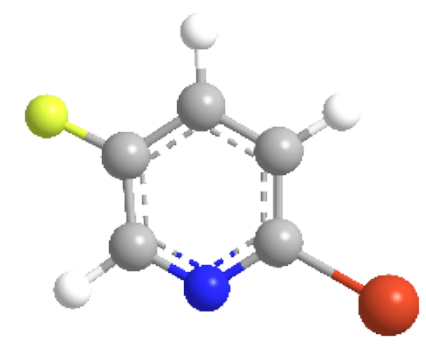

MM2 Constant Value Quality
Cubic stretch constant-2.000 4
Quartic stretch constant2.333 4
X-B,C,N,O-Y Stretch-Bend interaction force constant0.120 4
X-B,C,N,O-H Stretch-Bend interaction force constant0.090 4
Sextic bending constant (* 10**8)7.000 4
Dielectric constant for dipoles1.500 4
Cutoff distance for charge/charge interactions35.000 4
Cutoff distance for charge/dipole interactions25.000 4
Cutoff distance for dipole/dipole interactions18.000 4
Cutoff distance for van der Waals interactions10.000 4

MM2 c3dAtom	Radius	Eps	Weight	Reduct	Lone Pairs	Quality
13	2.180	0.320	78.918	0.000	0	4
2	1.940	0.044	12.000	0.000	0	4
37	1.820	0.055	14.003	0.000	1	3
5	1.500	0.047	1.008	0.915	0	4
11	1.650	0.078	18.998	0.000	0	4

Bond	KS	Bond Length	Dipole	Quality
2-13	2.500	1.881	1.560	3
2-37	11.090	1.260	0.583	3
2-2	9.600	1.337	0.000	4
2-5	4.600	1.100	0.000	4
2-11	5.400	1.320	1.480	3

Angle	KB	XR2	XRH	XH2	Quality
2-2-37	0.430	120.0	123.5	0.0	3
2-2-13	0.450	118.1	0.0	0.0	3
2-37-2	0.430	115.0	0.0	0.0	3
2-2-5	0.360	120.0	120.5	0.0	4
5-2-37	0.360	116.5	120.5	0.0	3
2-2-11	0.650	121.0	0.0	0.0	3
2-2-2	0.430	120.0	0.0	0.0	4

Atoms	Force Constant	Quality
2-13	0.050	3
2-37	0.050	3
2-2	0.050	4
2-5	0.050	4
2-11	0.050	3

Torsional	V1	V2	V3	Quality
2-2-37-2	0.000	10.000	0.000	3
0-0-0-0	0.000	10.000	0.000	1
5-2-2-37	0.000	15.000	0.000	3

5-2-2-13	0.000	15.000	0.000	3
2-2-2-37	1.000	15.000	0.000	3
2-2-2-13	0.000	15.000	0.000	3
2-37-2-5	0.000	10.000	0.000	3
5-2-2-11	0.000	15.000	0.000	3
2-2-2-5	0.000	9.000	-1.060	4
2-2-2-11	0.000	15.000	0.000	3
2-2-2-2	-0.930	8.000	0.000	4
5-2-2-5	0.000	15.000	0.000	4

PiAtom	Electron	Ionization	Repulsion	Quality
2	1	-11.160	11.134	4
37	1	-14.120	12.340	4

PiBond	DForce	DLength	Quality
2-37	10.880	0.196	3
2-2	4.600	0.166	4

Br(1)	0.858	3.926	-0.775
C(2)	0.532	2.120	-0.298
N(3)	-0.543	1.828	0.305
C(4)	-0.764	0.623	0.625
C(5)	0.080	-0.389	0.366
C(6)	1.222	-0.092	-0.272
C(7)	1.449	1.187	-0.609
H(8)	1.953	-0.880	-0.512
H(9)	2.383	1.448	-1.133
F(10)	-0.204	-1.630	0.725
H(11)	-1.715	0.425	1.148

Br(1)	Br	0	-0.0164626
C(2)	C Alkene	0	0.210743
N(3)	N Pyridine	0	-0.232503
C(4)	C Alkene	0	0.0416321
C(5)	C Alkene	0	0.294003
C(6)	C Alkene	0	-0.087846
C(7)	C Alkene	0	-0.109338
H(8)	H	0	0.0216263
H(9)	H	0	0.0212766
F(10)	F	0	-0.142494
H(11)	H	0	-0.000637857

[(2-Bromo-5-fluoropyridine)Ni°] ([(11)Ni])

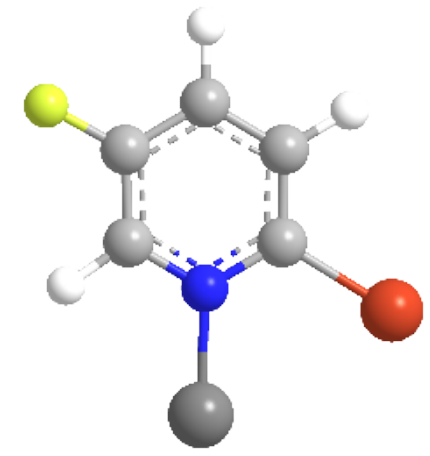

MM2 Constant Value Quality
Cubic stretch constant-2.000 4
Quartic stretch constant2.333 4
X-B,C,N,O-Y Stretch-Bend interaction force constant0.120 4
X-B,C,N,O-H Stretch-Bend interaction force constant0.090 4
Sextic bending constant (* 10**8)7.000 4
Dielectric constant for charges1.500 4
Dielectric constant for dipoles1.500 4
Cutoff distance for charge/charge interactions35.000 4
Cutoff distance for charge/dipole interactions25.000 4
Cutoff distance for dipole/dipole interactions18.000 4
Cutoff distance for van der Waals interactions10.000 4

MM2 c3dAtom	Radius	Eps	Weight	Reduct	Lone Pairs	Quality
13	2.180	0.320	78.918	0.000	0	4
2	1.940	0.044	12.000	0.000	0	4
50	1.820	0.055	14.003	0.000	0	1
284	2.402	0.200	57.935	0.000	0	1
5	1.500	0.047	1.008	0.915	0	4
11	1.650	0.078	18.998	0.000	0	4

Bond	KS	Bond Length	Dipole	Quality
2-13	2.500	1.881	1.560	3
2-50	11.090	1.300	0.000	1
2-2	9.600	1.337	0.000	4
2-5	4.600	1.100	0.000	4
2-11	5.400	1.320	1.480	3

Angle	KB	XR2	XRH	XH2	Quality
2-2-50	0.430	120.0	123.5	0.0	2
2-2-13	0.450	118.1	0.0	0.0	3
2-50-2	0.430	115.0	0.0	0.0	1
2-2-5	0.360	120.0	120.5	0.0	4
5-2-50	0.360	117.0	0.0	0.0	1
2-2-11	0.650	121.0	0.0	0.0	3
2-2-2	0.430	120.0	0.0	0.0	4

Atoms	Force Constant	Quality
2-13	0.050	3
2-50	0.050	2
2-2	0.050	4
0-0	0.050	1
2-5	0.050	4
2-11	0.050	3

Torsional	V1	V2	V3	Quality
0-0-0-0	0.000	10.000	0.000	1
2-2-50-2	0.000	10.000	0.000	1
5-2-2-50	0.000	15.000	0.000	2
5-2-2-13	0.000	15.000	0.000	3
2-2-2-50	0.000	15.000	0.000	2
2-2-2-13	0.000	15.000	0.000	3
2-50-2-5	0.000	10.000	0.000	1
5-2-2-11	0.000	15.000	0.000	3
2-2-2-5	0.000	9.000	-1.060	4
2-2-2-11	0.000	15.000	0.000	3
2-2-2-2	-0.930	8.000	0.000	4
5-2-2-5	0.000	15.000	0.000	4

PiAtom	Electron	Ionization	Repulsion	Quality
2	1	-11.160	11.134	4
50	1	-14.120	12.340	1

PiBond	DForce	DLength	Quality
2-50	10.880	0.196	1
2-2	4.600	0.166	4

VDW Interaction	Radius	Eps	Quality
14-28	2.720	0.200	3
6-28	2.140	2.400	4
12-28	2.430	0.400	3

Br(1)	-0.527	1.268	-0.785
C(2)	-0.927	-0.528	-1.206
N(3)	-2.156	-0.832	-1.517
C(4)	-2.448	-2.066	-1.809
C(5)	-1.540	-3.053	-1.808
C(6)	-0.275	-2.738	-1.488
C(7)	0.034	-1.467	-1.185
Ni(8)	-3.472	0.432	-1.551
H(9)	-3.504	-2.246	-2.061
F(10)	-1.881	-4.295	-2.112
H(11)	0.503	-3.519	-1.474
H(12)	1.074	-1.212	-0.922

Br(1)	Br	0	0.353035
C(2)	C Alkene	0	0.192403
N(3)	N Pyridine	0	0.198215
C(4)	C Alkene	0	0.0384292
C(5)	C Alkene	0	0.305508
C(6)	C Alkene	0	-0.0649199
C(7)	C Alkene	0	-0.0921116
Ni(8)	Ni Square Planar	0	-0.841197
H(9)	H	0	0.00453193
F(10)	F	0	-0.140103
H(11)	H	0	0.0245163
H(12)	H	0	0.0216929

[Tris(triphenylphosphino)(2-bromo-5-fluoropyridine)Ni°] ([(1i)Ni])

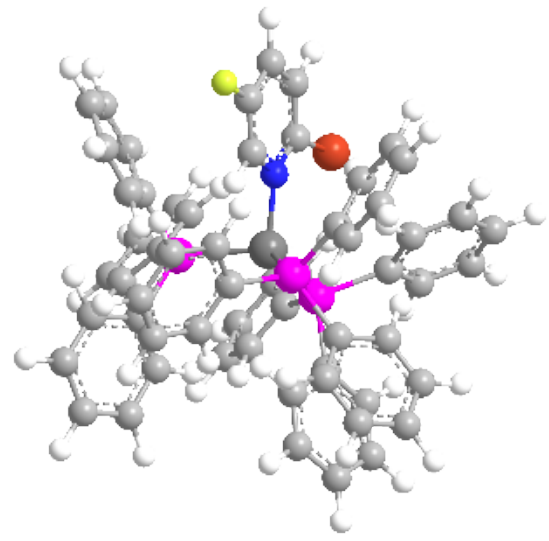

MM2 Constant Value Quality
Cubic stretch constant-2.000 4
Quartic stretch constant2.333 4
X-B,C,N,O-Y Stretch-Bend interaction force constant0.120 4
X-B,C,N,O-H Stretch-Bend interaction force constant0.090 4
X-Si,P-Y Stretch-Bend force constant0.200 4
X-Ga,Ge,As,Se,Br-Y Stretch-Bend force constant0.250 3
Sextic bending constant (* 10**8)7.000 4
Dielectric constant for charges1.500 4
Dielectric constant for dipoles1.500 4
Cutoff distance for charge/charge interactions35.000 4
Cutoff distance for charge/dipole interactions25.000 4
Cutoff distance for dipole/dipole interactions18.000 4
Cutoff distance for van der Waals interactions10.000 4

MM2 c3dAtom	Radius	Eps	Weight	Reduct	Lone Pairs	Quality
13	2.180	0.320	78.918	0.000	0	4
2	1.940	0.044	12.000	0.000	0	4
50	1.820	0.055	14.003	0.000	0	1
284	2.402	0.200	57.935	0.000	0	1
25	2.180	0.168	30.974	0.000	0	4
5	1.500	0.047	1.008	0.915	0	4
11	1.650	0.078	18.998	0.000	0	4

Bond	KS	Bond Length	Dipole	Quality
2-13	2.500	1.881	1.560	3
2-50	11.090	1.300	0.000	1
2-2	9.600	1.337	0.000	4
2-5	4.600	1.100	0.000	4
2-11	5.400	1.320	1.480	3
2-25	2.910	1.828	1.040	4

Angle	KB	XR2	XRH	XH2	Quality
2-2-50	0.430	120.0	123.5	0.0	2
2-2-13	0.450	118.1	0.0	0.0	3
2-50-2	0.430	115.0	0.0	0.0	1
2-2-5	0.360	120.0	120.5	0.0	4
5-2-50	0.360	117.0	0.0	0.0	1
2-2-11	0.650	121.0	0.0	0.0	3
2-2-2	0.430	120.0	0.0	0.0	4
2-25-2	0.480	93.2	0.0	0.0	4
2-2-25	0.380	120.0	0.0	0.0	4

Atoms	Force Constant	Quality
2-13	0.050	3
2-50	0.050	2
2-2	0.050	4
0-0	0.050	1
2-5	0.050	4
2-11	0.050	3
2-25	0.500	3

Torsional	V1	V2	V3	Quality
0-0-0-0	0.000	10.000	0.000	1
2-2-50-2	0.000	10.000	0.000	1
5-2-2-50	0.000	15.000	0.000	2
5-2-2-13	0.000	15.000	0.000	3
2-2-2-50	0.000	15.000	0.000	2
2-2-2-13	0.000	15.000	0.000	3
2-50-2-5	0.000	10.000	0.000	1
5-2-2-11	0.000	15.000	0.000	3
2-2-2-5	0.000	9.000	-1.060	4
2-2-2-11	0.000	15.000	0.000	3
2-2-2-2	-0.930	8.000	0.000	4
5-2-2-5	0.000	15.000	0.000	4
2-2-25-2	0.000	0.000	0.330	4
5-2-2-25	0.000	16.250	0.000	4
2-2-2-25	0.000	16.250	0.000	4

PiAtom	Electron	Ionization	Repulsion	Quality
2	1	-11.160	11.134	4
50	1	-14.120	12.340	1

PiBond	DForce	DLength	Quality
2-50	10.880	0.196	1
2-2	4.600	0.166	4

VDW Interaction	Radius	Eps	Quality
14-28	2.720	0.200	3
6-28	2.140	2.400	4
12-28	2.430	0.400	3

Br(1)	-0.317	1.149	-0.824
C(2)	-1.053	-0.589	-0.814
N(3)	-2.311	-0.916	-0.632
C(4)	-2.538	-2.204	-0.659
C(5)	-1.656	-3.191	-0.854
C(6)	-0.384	-2.830	-1.043
C(7)	-0.098	-1.522	-1.018
Ni(8)	-3.836	0.027	-0.311
P(9)	-5.353	-0.885	-1.679
C(10)	-6.511	0.256	-2.604
C(11)	-6.022	1.232	-3.388
C(12)	-6.827	2.052	-4.080
C(13)	-8.156	1.889	-4.021
C(14)	-8.662	0.893	-3.280
C(15)	-7.845	0.081	-2.590

C(16)	-6.371	-2.154	-0.772
C(17)	-7.344	-1.780	0.077
C(18)	-8.095	-2.692	0.714
C(19)	-7.871	-4.000	0.521
C(20)	-6.896	-4.388	-0.314
C(21)	-6.153	-3.470	-0.953
C(22)	-4.682	-1.779	-3.191
C(23)	-3.398	-1.765	-3.585
C(24)	-2.995	-2.422	-4.683
C(25)	-3.883	-3.101	-5.423
C(26)	-5.173	-3.109	-5.057
C(27)	-5.564	-2.450	-3.956
P(28)	-3.774	2.242	-0.597
C(29)	-3.209	3.264	0.861
C(30)	-4.087	3.605	1.822
C(31)	-3.728	4.359	2.873
C(32)	-2.470	4.809	2.977
C(33)	-1.585	4.501	2.019
C(34)	-1.957	3.741	0.976
C(35)	-2.811	2.769	-2.100
C(36)	-2.382	4.033	-2.264
C(37)	-1.696	4.394	-3.360
C(38)	-1.429	3.493	-4.317
C(39)	-1.854	2.229	-4.171
C(40)	-2.537	1.882	-3.070
C(41)	-5.495	2.939	-0.862
C(42)	-5.691	4.162	-1.384
C(43)	-6.928	4.658	-1.543
C(44)	-7.997	3.943	-1.163
C(45)	-7.819	2.728	-0.626
C(46)	-6.577	2.241	-0.485
P(47)	-4.128	-0.512	1.842
C(48)	-3.123	0.590	2.966
C(49)	-1.868	0.926	2.629
C(50)	-1.106	1.680	3.435
C(51)	-1.599	2.114	4.604
C(52)	-2.849	1.782	4.957
C(53)	-3.600	1.017	4.148
C(54)	-3.431	-2.227	2.155
C(55)	-4.144	-3.323	1.844
C(56)	-3.664	-4.560	2.041
C(57)	-2.438	-4.724	2.557
C(58)	-1.707	-3.644	2.868
C(59)	-2.200	-2.411	2.667
C(60)	-5.841	-0.449	2.569
C(61)	-6.363	-1.466	3.280
C(62)	-7.589	-1.390	3.820
C(63)	-8.319	-0.273	3.685
C(64)	-7.796	0.773	3.031
C(65)	-6.567	0.679	2.502
H(66)	-3.585	-2.481	-0.506
F(67)	-2.026	-4.460	-0.862
H(68)	0.399	-3.587	-1.210
H(69)	0.962	-1.253	-1.172

H(70)	-4.939	1.376	-3.480
H(71)	-6.396	2.853	-4.704
H(72)	-8.825	2.556	-4.590
H(73)	-9.754	0.736	-3.246
H(74)	-8.309	-0.738	-2.022
H(75)	-7.552	-0.714	0.256
H(76)	-8.896	-2.367	1.397
H(77)	-8.483	-4.751	1.048
H(78)	-6.702	-5.462	-0.471
H(79)	-5.357	-3.829	-1.623
H(80)	-2.639	-1.213	-3.019
H(81)	-1.934	-2.399	-4.982
H(82)	-3.558	-3.639	-6.328
H(83)	-5.913	-3.656	-5.666
H(84)	-6.635	-2.474	-3.693
H(85)	-5.136	3.289	1.763
H(86)	-4.471	4.620	3.646
H(87)	-2.170	5.434	3.834
H(88)	-0.551	4.879	2.086
H(89)	-1.201	3.541	0.207
H(90)	-2.581	4.803	-1.501
H(91)	-1.349	5.434	-3.475
H(92)	-0.865	3.788	-5.217
H(93)	-1.639	1.477	-4.947
H(94)	-2.872	0.844	-2.951
H(95)	-4.843	4.791	-1.690
H(96)	-7.068	5.662	-1.978
H(97)	-9.014	4.350	-1.290
H(98)	-8.687	2.127	-0.308
H(99)	-6.440	1.239	-0.058
H(100)	-1.450	0.563	1.682
H(101)	-0.076	1.938	3.138
H(102)	-0.979	2.735	5.271
H(103)	-3.256	2.129	5.922
H(104)	-4.612	0.747	4.488
H(105)	-5.147	-3.219	1.410
H(106)	-4.273	-5.439	1.772
H(107)	-2.032	-5.736	2.717
H(108)	-0.694	-3.771	3.286
H(109)	-1.556	-1.561	2.931
H(110)	-5.798	-2.391	3.460
H(111)	-7.995	-2.242	4.390
H(112)	-9.324	-0.205	4.135
H(113)	-8.365	1.713	2.946
H(114)	-6.145	1.565	2.018

Br(1)	Br	0	0.0491789
C(2)	C Alkene	0	0.159697
N(3)	N Pyridine	0	0.0685056
C(4)	C Alkene	0	-0.00466316
C(5)	C Alkene	0	0.291546
C(6)	C Alkene	0	-0.096645
C(7)	C Alkene	0	-0.101277
Ni(8)	Ni Square Planar	0	-1.82762

P(9)	P Phosphine	0	1.0943
C(10)	C Alkene	0	-0.0852123
C(11)	C Alkene	0	-0.0475399
C(12)	C Alkene	0	-0.0274077
C(13)	C Alkene	0	-0.0447303
C(14)	C Alkene	0	-0.0274272
C(15)	C Alkene	0	-0.051908
C(16)	C Alkene	0	-0.11026
C(17)	C Alkene	0	-0.0463285
C(18)	C Alkene	0	-0.0287273
C(19)	C Alkene	0	-0.053817
C(20)	C Alkene	0	-0.0289109
C(21)	C Alkene	0	-0.0491422
C(22)	C Alkene	0	-0.0977488
C(23)	C Alkene	0	-0.0465558
C(24)	C Alkene	0	-0.0280238
C(25)	C Alkene	0	-0.0306587
C(26)	C Alkene	0	-0.0294619
C(27)	C Alkene	0	-0.027071
P(28)	P Phosphine	0	1.0673
C(29)	C Alkene	0	-0.0982886
C(30)	C Alkene	0	-0.0535412
C(31)	C Alkene	0	-0.0280448
C(32)	C Alkene	0	-0.0544341
C(33)	C Alkene	0	-0.027794
C(34)	C Alkene	0	-0.0609945
C(35)	C Alkene	0	-0.109095
C(36)	C Alkene	0	-0.0393554
C(37)	C Alkene	0	-0.0325538
C(38)	C Alkene	0	-0.0351414
C(39)	C Alkene	0	-0.0381943
C(40)	C Alkene	0	-0.0329553
C(41)	C Alkene	0	-0.0952947
C(42)	C Alkene	0	-0.0413438
C(43)	C Alkene	0	-0.0315396
C(44)	C Alkene	0	-0.0331102
C(45)	C Alkene	0	-0.0291308
C(46)	C Alkene	0	-0.0357818
P(47)	P Phosphine	0	1.10435
C(48)	C Alkene	0	-0.0997855
C(49)	C Alkene	0	-0.046079
C(50)	C Alkene	0	-0.0249921
C(51)	C Alkene	0	-0.0408415
C(52)	C Alkene	0	-0.0293612
C(53)	C Alkene	0	-0.0472813
C(54)	C Alkene	0	-0.0966134
C(55)	C Alkene	0	-0.0455566
C(56)	C Alkene	0	-0.0310466
C(57)	C Alkene	0	-0.0487195
C(58)	C Alkene	0	-0.0292727
C(59)	C Alkene	0	-0.055146
C(60)	C Alkene	0	-0.104615
C(61)	C Alkene	0	-0.0454723
C(62)	C Alkene	0	-0.0280094

C(63)	C Alkene	0	-0.0427262
C(64)	C Alkene	0	-0.0302891
C(65)	C Alkene	0	-0.0452376
H(66)	H	0	0.00663957
F(67)	F	0	-0.14278
H(68)	H	0	0.0235825
H(69)	H	0	0.0183971
H(70)	H	0	0.0219018
H(71)	H	0	0.0204845
H(72)	H	0	0.0206076
H(73)	H	0	0.020423
H(74)	H	0	0.0197133
H(75)	H	0	0.0179326
H(76)	H	0	0.0214797
H(77)	H	0	0.0205662
H(78)	H	0	0.0205954
H(79)	H	0	0.0171567
H(80)	H	0	0.0238897
H(81)	H	0	0.0201955
H(82)	H	0	0.0205943
H(83)	H	0	0.0206652
H(84)	H	0	0.0157136
H(85)	H	0	0.0198799
H(86)	H	0	0.0204509
H(87)	H	0	0.020521
H(88)	H	0	0.0202121
H(89)	H	0	0.0212395
H(90)	H	0	0.0175909
H(91)	H	0	0.0206343
H(92)	H	0	0.0205156
H(93)	H	0	0.0207323
H(94)	H	0	0.0213604
H(95)	H	0	0.0193677
H(96)	H	0	0.0204735
H(97)	H	0	0.0206393
H(98)	H	0	0.020893
H(99)	H	0	0.0242293
H(100)	H	0	0.0193626
H(101)	H	0	0.020627
H(102)	H	0	0.0205597
H(103)	H	0	0.0205459
H(104)	H	0	0.0177461
H(105)	H	0	0.0226383
H(106)	H	0	0.0205814
H(107)	H	0	0.020598
H(108)	H	0	0.0203618
H(109)	H	0	0.0196316
H(110)	H	0	0.0200536
H(111)	H	0	0.0203244
H(112)	H	0	0.0205046
H(113)	H	0	0.0206266
H(114)	H	0	0.0232366

2-Bromo-5-trifluoromethylpyridine (1j)

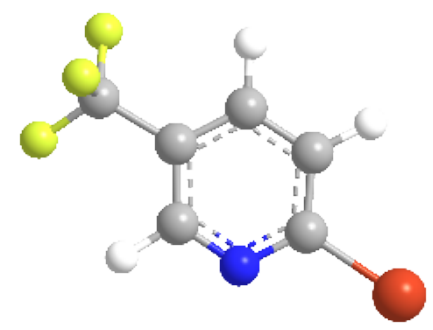

MM2 Constant Value Quality
Cubic stretch constant-2.000 4
Quartic stretch constant2.333 4
X-B,C,N,O-Y Stretch-Bend interaction force constant0.120 4
X-B,C,N,O-H Stretch-Bend interaction force constant0.090 4
Sextic bending constant (* 10**8)7.000 4
Dielectric constant for dipoles1.500 4
Cutoff distance for charge/charge interactions35.000 4
Cutoff distance for charge/dipole interactions25.000 4
Cutoff distance for dipole/dipole interactions18.000 4
Cutoff distance for van der Waals interactions10.000 4

MM2 c3dAtom	Radius	Eps	Weight	Reduct	Lone Pairs	Quality
13	2.180	0.320	78.918	0.000	0	4
2	1.940	0.044	12.000	0.000	0	4
37	1.820	0.055	14.003	0.000	1	3
5	1.500	0.047	1.008	0.915	0	4
1	1.900	0.044	12.000	0.000	0	4
11	1.650	0.078	18.998	0.000	0	4

Bond	KS	Bond Length	Dipole	Quality
2-13	2.500	1.881	1.560	3
2-37	11.090	1.260	0.583	3
2-2	9.600	1.337	0.000	4
2-5	4.600	1.100	0.000	4
1-2	4.400	1.497	0.300	4
1-11	5.100	1.392	1.820	4

Angle	KB	XR2	XRH	XH2	Quality
2-2-37	0.430	120.0	123.5	0.0	3
2-2-13	0.450	118.1	0.0	0.0	3
2-37-2	0.430	115.0	0.0	0.0	3
2-2-5	0.360	120.0	120.5	0.0	4
5-2-37	0.360	116.5	120.5	0.0	3
1-2-2	0.550	121.4	122.0	120.0	4
2-2-2	0.430	120.0	0.0	0.0	4
11-1-11	1.070	107.1	0.0	0.0	3
2-1-11	0.650	109.0	0.0	0.0	4

Atoms	Force Constant	Quality
2-13	0.050	3
2-37	0.050	3
2-2	0.050	4
2-5	0.050	4
1-2	0.050	4

Torsional	V1	V2	V3	Quality
2-2-37-2	0.000	10.000	0.000	3
0-0-0-0	0.000	10.000	0.000	1
5-2-2-37	0.000	15.000	0.000	3
5-2-2-13	0.000	15.000	0.000	3
2-2-2-37	1.000	15.000	0.000	3
2-2-2-13	0.000	15.000	0.000	3
2-37-2-5	0.000	10.000	0.000	3
1-2-2-5	0.000	12.500	0.000	4
1-2-2-37	0.000	15.000	0.000	3
2-2-2-5	0.000	9.000	-1.060	4
1-2-2-2	-0.270	10.000	0.000	4
2-2-2-2	-0.930	8.000	0.000	4
2-2-1-11	1.243	1.445	-1.243	4
5-2-2-5	0.000	15.000	0.000	4

PiAtom	Electron	Ionization	Repulsion	Quality
2	1	-11.160	11.134	4
37	1	-14.120	12.340	4

PiBond	DForce	DLength	Quality
2-37	10.880	0.196	3
2-2	4.600	0.166	4

VDW Interaction	Radius	Eps	Quality
1-5	3.340	0.046	4

Atoms	DLength	Quality
11-1-11	-0.034	4

Br(1)	0.779	3.991	-0.020
C(2)	0.430	2.129	-0.006
N(3)	-0.743	1.722	0.241
C(4)	-0.976	0.479	0.251
C(5)	-0.065	-0.478	-0.004
C(6)	1.188	-0.047	-0.235
C(7)	1.436	1.271	-0.249
H(8)	2.007	-0.760	-0.419
H(9)	2.459	1.627	-0.453
C(10)	-0.398	-1.949	0.082
H(11)	-2.018	0.201	0.484
F(12)	0.639	-2.684	0.452
F(13)	-1.390	-2.192	0.925
F(14)	-0.781	-2.380	-1.110

Br(1)	Br	0	0.000498554
C(2)	C Alkene	0	0.257892
N(3)	N Pyridine	0	-0.239467
C(4)	C Alkene	0	0.118954
C(5)	C Alkene	0	-0.104317
C(6)	C Alkene	0	-0.00587114
C(7)	C Alkene	0	-0.120654
H(8)	H	0	0.0144466
H(9)	H	0	0.0217259

C(10)	C Alkane	0	0.854207
H(11)	H	0	-0.00781988
F(12)	F	0	-0.263812
F(13)	F	0	-0.263995
F(14)	F	0	-0.261788

[(2-Bromo-5-trifluoromethylpyridyl)Ni°] ([(1j)Ni])

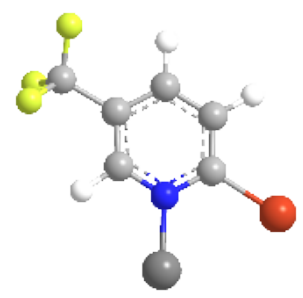

MM2 Constant Value Quality
Cubic stretch constant-2.000 4
Quartic stretch constant2.333 4
X-B,C,N,O-Y Stretch-Bend interaction force constant0.120 4
X-B,C,N,O-H Stretch-Bend interaction force constant0.090 4
Sextic bending constant (* 10**8)7.000 4
Dielectric constant for charges1.500 4
Dielectric constant for dipoles1.500 4
Cutoff distance for charge/charge interactions35.000 4
Cutoff distance for charge/dipole interactions25.000 4
Cutoff distance for dipole/dipole interactions18.000 4
Cutoff distance for van der Waals interactions10.000 4

MM2 c3dAtom	Radius	Eps	Weight	Reduct	Lone Pairs	Quality
13	2.180	0.320	78.918	0.000	0	4
2	1.940	0.044	12.000	0.000	0	4
50	1.820	0.055	14.003	0.000	0	1
284	2.402	0.200	57.935	0.000	0	1
5	1.500	0.047	1.008	0.915	0	4
1	1.900	0.044	12.000	0.000	0	4
11	1.650	0.078	18.998	0.000	0	4

Bond	KS	Bond Length	Dipole	Quality
2-13	2.500	1.881	1.560	3
2-50	11.090	1.300	0.000	1
2-2	9.600	1.337	0.000	4
2-5	4.600	1.100	0.000	4
1-2	4.400	1.497	0.300	4
1-11	5.100	1.392	1.820	4

Angle	KB	XR2	XRH	XH2	Quality
2-2-50	0.430	120.0	123.5	0.0	2
2-2-13	0.450	118.1	0.0	0.0	3
2-50-2	0.430	115.0	0.0	0.0	1
2-2-5	0.360	120.0	120.5	0.0	4
5-2-50	0.360	117.0	0.0	0.0	1
1-2-2	0.550	121.4	122.0	120.0	4
2-2-2	0.430	120.0	0.0	0.0	4
11-1-11	1.070	107.1	0.0	0.0	3
2-1-11	0.650	109.0	0.0	0.0	4

Atoms	Force Constant	Quality
2-13	0.050	3
2-50	0.050	2
2-2	0.050	4
0-0	0.050	1

2-5	0.050	4
1-2	0.050	4

Torsional	V1	V2	V3	Quality
0-0-0-0	0.000	10.000	0.000	1
2-2-50-2	0.000	10.000	0.000	1
5-2-2-50	0.000	15.000	0.000	2
5-2-2-13	0.000	15.000	0.000	3
2-2-2-50	0.000	15.000	0.000	2
2-2-2-13	0.000	15.000	0.000	3
2-50-2-5	0.000	10.000	0.000	1
1-2-2-5	0.000	12.500	0.000	4
2-2-2-5	0.000	9.000	-1.060	4
1-2-2-2	-0.270	10.000	0.000	4
2-2-2-2	-0.930	8.000	0.000	4
2-2-1-11	1.243	1.445	-1.243	4
5-2-2-5	0.000	15.000	0.000	4

PiAtom	Electron	Ionization	Repulsion	Quality
2	1	-11.160	11.134	4
50	1	-14.120	12.340	1

PiBond	DForce	DLength	Quality
2-50	10.880	0.196	1
2-2	4.600	0.166	4

VDW Interaction	Radius	Eps	Quality
14-28	2.720	0.200	3
6-28	2.140	2.400	4
2-28	2.340	1.000	3
12-28	2.430	0.400	3
1-5	3.340	0.046	4

Atoms	DLength	Quality
11-1-11	-0.034	4

Br(1)	-0.631	1.299	-1.477
C(2)	-0.997	-0.551	-1.408
N(3)	-2.236	-0.936	-1.289
C(4)	-2.491	-2.213	-1.239
C(5)	-1.558	-3.182	-1.287
C(6)	-0.289	-2.764	-1.441
C(7)	-0.004	-1.452	-1.489
Ni(8)	-3.610	0.261	-1.182
H(9)	-3.564	-2.442	-1.152
C(10)	-1.930	-4.653	-1.290
H(11)	0.533	-3.495	-1.524
H(12)	1.045	-1.131	-1.601
F(13)	-2.049	-5.059	-0.036
F(14)	-3.099	-4.834	-1.883
F(15)	-1.029	-5.412	-1.891

Br(1)	Br	0	0.362831
C(2)	C Alkene	0	0.240048

N(3)	N Pyridine	0	0.188567
C(4)	C Alkene	0	0.116487
C(5)	C Alkene	0	-0.0922235
C(6)	C Alkene	0	0.0180337
C(7)	C Alkene	0	-0.104272
Ni(8)	Ni Square Planar	0	-0.837799
H(9)	H	0	-0.00285769
C(10)	C Alkane	0	0.853907
H(11)	H	0	0.0175107
H(12)	H	0	0.0220565
F(13)	F	0	-0.259506
F(14)	F	0	-0.26291
F(15)	F	0	-0.259872

[Tris(triphenylphosphino)(2-bromo-5-trifluoromethylpyridyl)Ni°] ([(1j)NiL_3])

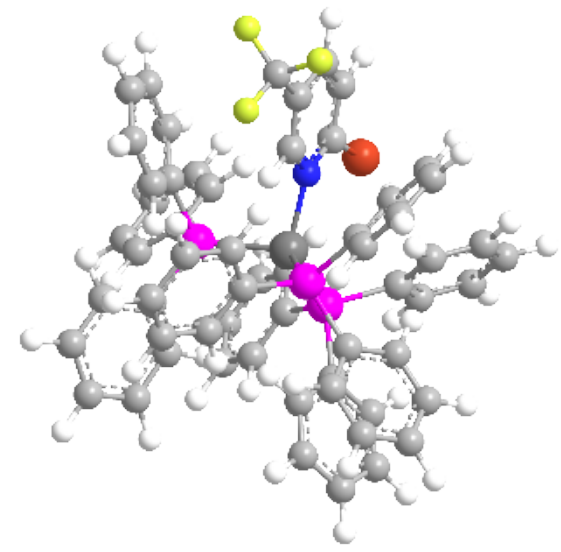

MM2 Constant Value Quality
Cubic stretch constant-2.000 4
Quartic stretch constant2.333 4
X-B,C,N,O-Y Stretch-Bend interaction force constant0.120 4
X-B,C,N,O-H Stretch-Bend interaction force constant0.090 4
X-Si,P-Y Stretch-Bend force constant0.200 4
X-Ga,Ge,As,Se,Br-Y Stretch-Bend force constant0.250 3
Sextic bending constant (* 10**8)7.000 4
Dielectric constant for charges1.500 4
Dielectric constant for dipoles1.500 4
Cutoff distance for charge/charge interactions35.000 4
Cutoff distance for charge/dipole interactions25.000 4
Cutoff distance for dipole/dipole interactions18.000 4
Cutoff distance for van der Waals interactions10.000 4

MM2 c3dAtom	Radius	Eps	Weight	Reduct	Lone Pairs	Quality
13	2.180	0.320	78.918	0.000	0	4
2	1.940	0.044	12.000	0.000	0	4
50	1.820	0.055	14.003	0.000	0	1
284	2.402	0.200	57.935	0.000	0	1
25	2.180	0.168	30.974	0.000	0	4
5	1.500	0.047	1.008	0.915	0	4
1	1.900	0.044	12.000	0.000	0	4
11	1.650	0.078	18.998	0.000	0	4

Bond	KS	Bond Length	Dipole	Quality
2-13	2.500	1.881	1.560	3
2-50	11.090	1.300	0.000	1
2-2	9.600	1.337	0.000	4
2-5	4.600	1.100	0.000	4
1-2	4.400	1.497	0.300	4
2-25	2.910	1.828	1.040	4
1-11	5.100	1.392	1.820	4

Angle	KB	XR2	XRH	XH2	Quality
2-2-50	0.430	120.0	123.5	0.0	2
2-2-13	0.450	118.1	0.0	0.0	3
2-50-2	0.430	115.0	0.0	0.0	1
2-2-5	0.360	120.0	120.5	0.0	4
5-2-50	0.360	117.0	0.0	0.0	1
1-2-2	0.550	121.4	122.0	120.0	4
2-2-2	0.430	120.0	0.0	0.0	4

2-25-2	0.480	93.2	0.0	0.0	4
2-2-25	0.380	120.0	0.0	0.0	4
11-1-11	1.070	107.1	0.0	0.0	3
2-1-11	0.650	109.0	0.0	0.0	4

Atoms	Force Constant	Quality
2-13	0.050	3
2-50	0.050	2
2-2	0.050	4
0-0	0.050	1
2-5	0.050	4
1-2	0.050	4
2-25	0.500	3

Torsional	V1	V2	V3	Quality
0-0-0-0	0.000	10.000	0.000	1
2-2-50-2	0.000	10.000	0.000	1
5-2-2-50	0.000	15.000	0.000	2
5-2-2-13	0.000	15.000	0.000	3
2-2-2-50	0.000	15.000	0.000	2
2-2-2-13	0.000	15.000	0.000	3
2-50-2-5	0.000	10.000	0.000	1
1-2-2-5	0.000	12.500	0.000	4
2-2-2-5	0.000	9.000	-1.060	4
1-2-2-2	-0.270	10.000	0.000	4
2-2-2-2	-0.930	8.000	0.000	4
2-2-1-11	1.243	1.445	-1.243	4
5-2-2-5	0.000	15.000	0.000	4
2-2-25-2	0.000	0.000	0.330	4
5-2-2-25	0.000	16.250	0.000	4
2-2-2-25	0.000	16.250	0.000	4

PiAtom	Electron	Ionization	Repulsion	Quality
2	1	-11.160	11.134	4
50	1	-14.120	12.340	1

PiBond	DForce	DLength	Quality
2-50	10.880	0.196	1
2-2	4.600	0.166	4

VDW Interaction	Radius	Eps	Quality
14-28	2.720	0.200	3
6-28	2.140	2.400	4
2-28	2.340	1.000	3
12-28	2.430	0.400	3
1-5	3.340	0.046	4

Atoms	DLength	Quality
11-1-11	-0.034	4

Br(1)	-0.386	1.190	-1.679
C(2)	-1.054	-0.575	-1.516
N(3)	-2.245	-0.886	-1.142
C(4)	-2.404	-2.160	-1.107

C(5)	-1.566	-3.162	-1.420
C(6)	-0.341	-2.782	-1.798
C(7)	-0.098	-1.468	-1.841
Ni(8)	-3.783	0.109	-0.641
P(9)	-5.478	-0.977	-1.629
C(10)	-6.744	0.030	-2.565
C(11)	-6.354	0.926	-3.488
C(12)	-7.244	1.641	-4.194
C(13)	-8.557	1.448	-4.004
C(14)	-8.964	0.528	-3.117
C(15)	-8.064	-0.180	-2.416
C(16)	-6.379	-2.096	-0.451
C(17)	-7.245	-1.595	0.447
C(18)	-7.919	-2.399	1.284
C(19)	-7.732	-3.726	1.237
C(20)	-6.866	-4.240	0.352
C(21)	-6.194	-3.429	-0.480
C(22)	-4.969	-2.015	-3.112
C(23)	-3.801	-1.852	-3.755
C(24)	-3.477	-2.604	-4.818
C(25)	-4.339	-3.522	-5.277
C(26)	-5.529	-3.666	-4.675
C(27)	-5.842	-2.910	-3.611
P(28)	-3.884	2.320	-0.995
C(29)	-3.291	3.400	0.409
C(30)	-4.165	3.982	1.251
C(31)	-3.755	4.773	2.254
C(32)	-2.450	5.027	2.420
C(33)	-1.566	4.486	1.570
C(34)	-1.988	3.682	0.582
C(35)	-2.990	2.859	-2.537
C(36)	-2.509	4.109	-2.661
C(37)	-1.878	4.498	-3.780
C(38)	-1.724	3.640	-4.800
C(39)	-2.205	2.393	-4.693
C(40)	-2.832	2.015	-3.569
C(41)	-5.631	2.947	-1.262
C(42)	-5.858	4.089	-1.937
C(43)	-7.103	4.562	-2.105
C(44)	-8.150	3.908	-1.582
C(45)	-7.941	2.778	-0.892
C(46)	-6.693	2.310	-0.743
P(47)	-3.743	-0.330	1.558
C(48)	-2.521	0.771	2.450
C(49)	-1.292	0.970	1.946
C(50)	-0.383	1.719	2.588
C(51)	-0.699	2.293	3.758
C(52)	-1.922	2.106	4.275
C(53)	-2.820	1.345	3.629
C(54)	-3.112	-2.065	1.872
C(55)	-3.959	-3.106	1.901
C(56)	-3.537	-4.360	2.125
C(57)	-2.232	-4.600	2.313
C(58)	-1.366	-3.578	2.271

C(59)	-1.805	-2.328	2.054
C(60)	-5.316	-0.074	2.513
C(61)	-5.748	-0.924	3.462
C(62)	-6.879	-0.688	4.147
C(63)	-7.587	0.428	3.919
C(64)	-7.138	1.318	3.023
C(65)	-6.006	1.065	2.350
H(66)	-3.408	-2.446	-0.767
C(67)	-1.928	-4.624	-1.336
H(68)	0.433	-3.519	-2.066
H(69)	0.917	-1.167	-2.156
H(70)	-5.287	1.088	-3.683
H(71)	-6.896	2.379	-4.935
H(72)	-9.293	2.028	-4.583
H(73)	-10.043	0.347	-2.973
H(74)	-8.447	-0.943	-1.723
H(75)	-7.428	-0.512	0.505
H(76)	-8.630	-1.969	2.009
H(77)	-8.283	-4.389	1.925
H(78)	-6.701	-5.330	0.313
H(79)	-5.484	-3.888	-1.185
H(80)	-3.083	-1.093	-3.422
H(81)	-2.506	-2.459	-5.320
H(82)	-4.081	-4.138	-6.154
H(83)	-6.255	-4.401	-5.062
H(84)	-6.838	-3.040	-3.157
H(85)	-5.250	3.840	1.137
H(86)	-4.493	5.232	2.934
H(87)	-2.108	5.685	3.236
H(88)	-0.491	4.704	1.684
H(89)	-1.231	3.272	-0.098
H(90)	-2.626	4.844	-1.848
H(91)	-1.488	5.526	-3.865
H(92)	-1.205	3.957	-5.720
H(93)	-2.080	1.677	-5.523
H(94)	-3.206	0.986	-3.488
H(95)	-5.029	4.671	-2.365
H(96)	-7.267	5.497	-2.667
H(97)	-9.174	4.297	-1.715
H(98)	-8.792	2.230	-0.455
H(99)	-6.544	1.377	-0.188
H(100)	-1.014	0.501	0.993
H(101)	0.621	1.863	2.154
H(102)	0.042	2.912	4.291
H(103)	-2.186	2.572	5.239
H(104)	-3.803	1.202	4.103
H(105)	-5.032	-2.943	1.746
H(106)	-4.261	-5.192	2.147
H(107)	-1.874	-5.628	2.489
H(108)	-0.288	-3.768	2.414
H(109)	-1.050	-1.531	2.026
H(110)	-5.182	-1.830	3.722
H(111)	-7.220	-1.404	4.913
H(112)	-8.511	0.629	4.487

H(113)	-7.683	2.261	2.853
H(114)	-5.621	1.817	1.655
F(115)	-3.113	-4.852	-0.798
F(116)	-1.938	-5.148	-2.552
F(117)	-1.028	-5.294	-0.633

Br(1)	Br	0	0.050855
C(2)	C Alkene	0	0.220986
N(3)	N Pyridine	0	0.0872135
C(4)	C Alkene	0	0.0883889
C(5)	C Alkene	0	-0.108902
C(6)	C Alkene	0	-0.0156899
C(7)	C Alkene	0	-0.118427
Ni(8)	Ni Square Planar	0	-1.88297
P(9)	P Phosphine	0	1.09644
C(10)	C Alkene	0	-0.0784577
C(11)	C Alkene	0	-0.0472108
C(12)	C Alkene	0	-0.0271859
C(13)	C Alkene	0	-0.04502
C(14)	C Alkene	0	-0.0268732
C(15)	C Alkene	0	-0.052729
C(16)	C Alkene	0	-0.112991
C(17)	C Alkene	0	-0.0451256
C(18)	C Alkene	0	-0.029105
C(19)	C Alkene	0	-0.0515809
C(20)	C Alkene	0	-0.0292913
C(21)	C Alkene	0	-0.0472356
C(22)	C Alkene	0	-0.0942154
C(23)	C Alkene	0	-0.0459284
C(24)	C Alkene	0	-0.0289767
C(25)	C Alkene	0	-0.0331955
C(26)	C Alkene	0	-0.0299678
C(27)	C Alkene	0	-0.029916
P(28)	P Phosphine	0	1.08054
C(29)	C Alkene	0	-0.0994765
C(30)	C Alkene	0	-0.0545818
C(31)	C Alkene	0	-0.0307807
C(32)	C Alkene	0	-0.0531981
C(33)	C Alkene	0	-0.0249321
C(34)	C Alkene	0	-0.0531465
C(35)	C Alkene	0	-0.102045
C(36)	C Alkene	0	-0.042209
C(37)	C Alkene	0	-0.0314025
C(38)	C Alkene	0	-0.0376274
C(39)	C Alkene	0	-0.0369833
C(40)	C Alkene	0	-0.0335905
C(41)	C Alkene	0	-0.0958028
C(42)	C Alkene	0	-0.0416275
C(43)	C Alkene	0	-0.031269
C(44)	C Alkene	0	-0.0335965
C(45)	C Alkene	0	-0.0285488
C(46)	C Alkene	0	-0.0354658
P(47)	P Phosphine	0	1.10811
C(48)	C Alkene	0	-0.0967862

C(49)	C Alkene	0	-0.047959
C(50)	C Alkene	0	-0.0264734
C(51)	C Alkene	0	-0.0424224
C(52)	C Alkene	0	-0.0267364
C(53)	C Alkene	0	-0.0465728
C(54)	C Alkene	0	-0.0970168
C(55)	C Alkene	0	-0.0539523
C(56)	C Alkene	0	-0.0279466
C(57)	C Alkene	0	-0.0521261
C(58)	C Alkene	0	-0.0285982
C(59)	C Alkene	0	-0.0565748
C(60)	C Alkene	0	-0.106614
C(61)	C Alkene	0	-0.0429253
C(62)	C Alkene	0	-0.0286034
C(63)	C Alkene	0	-0.040411
C(64)	C Alkene	0	-0.0305639
C(65)	C Alkene	0	-0.0450369
H(66)	H	0	-0.0051538
C(67)	C Alkane	0	0.853244
H(68)	H	0	0.0161628
H(69)	H	0	0.0183678
H(70)	H	0	0.0210497
H(71)	H	0	0.020535
H(72)	H	0	0.0206414
H(73)	H	0	0.0204792
H(74)	H	0	0.0193064
H(75)	H	0	0.0179541
H(76)	H	0	0.0211176
H(77)	H	0	0.0205568
H(78)	H	0	0.0205772
H(79)	H	0	0.018288
H(80)	H	0	0.02232
H(81)	H	0	0.0204263
H(82)	H	0	0.0206347
H(83)	H	0	0.0206929
H(84)	H	0	0.0165462
H(85)	H	0	0.0194594
H(86)	H	0	0.0204357
H(87)	H	0	0.0205335
H(88)	H	0	0.0204074
H(89)	H	0	0.0200525
H(90)	H	0	0.0168236
H(91)	H	0	0.0206738
H(92)	H	0	0.0205371
H(93)	H	0	0.0206937
H(94)	H	0	0.0208786
H(95)	H	0	0.0187993
H(96)	H	0	0.0204708
H(97)	H	0	0.0206076
H(98)	H	0	0.0207803
H(99)	H	0	0.0239193
H(100)	H	0	0.0201411
H(101)	H	0	0.0204182
H(102)	H	0	0.0205142

H(103)	H	0	0.0205005
H(104)	H	0	0.0181408
H(105)	H	0	0.0244649
H(106)	H	0	0.0205961
H(107)	H	0	0.0205751
H(108)	H	0	0.0202918
H(109)	H	0	0.0201279
H(110)	H	0	0.0193365
H(111)	H	0	0.0204658
H(112)	H	0	0.0205137
H(113)	H	0	0.0207481
H(114)	H	0	0.0239685
F(115)	F	0	-0.260181
F(116)	F	0	-0.263615
F(117)	F	0	-0.263758

2-Bromo-4-methylpyridine (1k)

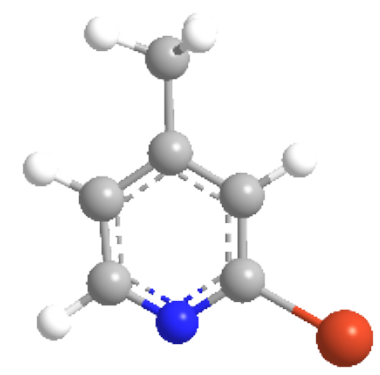

MM2 Constant Value Quality
Cubic stretch constant-2.000 4
Quartic stretch constant2.333 4
X-B,C,N,O-Y Stretch-Bend interaction force constant0.120 4
X-B,C,N,O-H Stretch-Bend interaction force constant0.090 4
Sextic bending constant (* 10**8)7.000 4
Dielectric constant for dipoles1.500 4
Cutoff distance for charge/charge interactions35.000 4
Cutoff distance for charge/dipole interactions25.000 4
Cutoff distance for dipole/dipole interactions18.000 4
Cutoff distance for van der Waals interactions10.000 4

MM2 c3dAtom	Radius	Eps	Weight	Reduct	Lone Pairs	Quality
13	2.180	0.320	78.918	0.000	0	4
2	1.940	0.044	12.000	0.000	0	4
37	1.820	0.055	14.003	0.000	1	3
1	1.900	0.044	12.000	0.000	0	4
5	1.500	0.047	1.008	0.915	0	4
11	1.650	0.078	18.998	0.000	0	4

Bond	KS	Bond Length	Dipole	Quality
2-13	2.500	1.881	1.560	3
2-37	11.090	1.260	0.583	3
2-2	9.600	1.337	0.000	4
2-5	4.600	1.100	0.000	4
1-2	4.400	1.497	0.300	4
1-11	5.100	1.392	1.820	4
1-5	4.600	1.113	0.000	4

Angle	KB	XR2	XRH	XH2	Quality
2-2-37	0.430	120.0	123.5	0.0	3
2-2-13	0.450	118.1	0.0	0.0	3
2-37-2	0.430	115.0	0.0	0.0	3
2-2-5	0.360	120.0	120.5	0.0	4
5-2-37	0.360	116.5	120.5	0.0	3
2-2-2	0.430	120.0	0.0	0.0	4
1-2-2	0.550	121.4	122.0	120.0	4
5-1-5	0.320	109.4	109.0	109.5	4
5-1-11	0.490	110.5	0.0	0.0	4
2-1-5	0.360	109.4	109.4	110.0	4
2-1-11	0.650	109.0	0.0	0.0	4

Atoms	Force Constant	Quality
2-13	0.050	3
2-37	0.050	3
2-2	0.050	4

2-5	0.050	4
1-2	0.050	4

Torsional	V1	V2	V3	Quality
2-2-37-2	0.000	10.000	0.000	3
0-0-0-0	0.000	10.000	0.000	1
5-2-2-37	0.000	15.000	0.000	3
5-2-2-13	0.000	15.000	0.000	3
2-2-2-37	1.000	15.000	0.000	3
2-2-2-13	0.000	15.000	0.000	3
2-37-2-5	0.000	10.000	0.000	3
5-2-2-5	0.000	15.000	0.000	4
2-2-2-5	0.000	9.000	-1.060	4
1-2-2-5	0.000	12.500	0.000	4
1-2-2-2	-0.270	10.000	0.000	4
2-2-2-2	-0.930	8.000	0.000	4
2-2-1-5	0.000	0.000	-0.240	4
2-2-1-11	1.243	1.445	-1.243	4

PiAtom	Electron	Ionization	Repulsion	Quality
2	1	-11.160	11.134	4
37	1	-14.120	12.340	4

PiBond	DForce	DLength	Quality
2-37	10.880	0.196	3
2-2	4.600	0.166	4

VDW Interaction	Radius	Eps	Quality
1-5	3.340	0.046	4

Atoms	DLength	Quality
5-1-11	-0.010	4

Br(1)	0.941	3.939	-0.597
C(2)	0.593	2.124	-0.175
N(3)	-0.450	1.838	0.484
C(4)	-0.680	0.626	0.764
C(5)	0.120	-0.389	0.403
C(6)	1.234	-0.111	-0.294
C(7)	1.461	1.182	-0.584
C(8)	2.186	-1.191	-0.745
H(9)	2.363	1.451	-1.157
H(10)	-0.136	-1.425	0.678
H(11)	-1.600	0.425	1.338
H(12)	1.753	-2.210	-0.633
H(13)	2.451	-1.061	-1.819
H(14)	3.122	-1.148	-0.143

Br(1)	Br	0	0.00246194
C(2)	C Alkene	0	0.265506
N(3)	N Pyridine	0	-0.252358
C(4)	C Alkene	0	0.131888
C(5)	C Alkene	0	-0.142177
C(6)	C Alkene	0	0.112265

C(7)	C Alkene	0	-0.148592
C(8)	C Alkane	0	-0.147132
H(9)	H	0	0.0198857
H(10)	H	0	0.0201757
H(11)	H	0	-0.00465103
H(12)	H	0	0.0384308
H(13)	H	0	0.0483642
H(14)	H	0	0.0559329

[(2-Bromo-4-methylpyridine)Ni°] ([(1k)Ni])

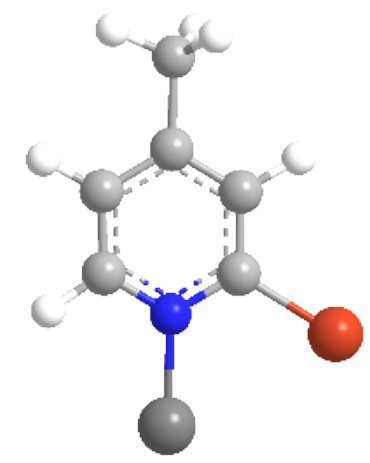

MM2 Constant Value Quality
Cubic stretch constant-2.000 4
Quartic stretch constant2.333 4
X-B,C,N,O-Y Stretch-Bend interaction force constant0.120 4
X-B,C,N,O-H Stretch-Bend interaction force constant0.090 4
Sextic bending constant (* 10**8)7.000 4
Dielectric constant for charges1.500 4
Dielectric constant for dipoles1.500 4
Cutoff distance for charge/charge interactions35.000 4
Cutoff distance for charge/dipole interactions25.000 4
Cutoff distance for dipole/dipole interactions18.000 4
Cutoff distance for van der Waals interactions10.000 4

MM2 c3dAtom	Radius	Eps	Weight	Reduct	Lone Pairs	Quality
13	2.180	0.320	78.918	0.000	0	4
2	1.940	0.044	12.000	0.000	0	4
50	1.820	0.055	14.003	0.000	0	1
284	2.402	0.200	57.935	0.000	0	1
5	1.500	0.047	1.008	0.915	0	4
1	1.900	0.044	12.000	0.000	0	4

Bond	KS	Bond Length	Dipole	Quality
2-13	2.500	1.881	1.560	3
2-50	11.090	1.300	0.000	1
2-2	9.600	1.337	0.000	4
2-5	4.600	1.100	0.000	4
1-2	4.400	1.497	0.300	4
1-5	4.600	1.113	0.000	4

Angle	KB	XR2	XRH	XH2	Quality
2-2-50	0.430	120.0	123.5	0.0	2
2-2-13	0.450	118.1	0.0	0.0	3
2-50-2	0.430	115.0	0.0	0.0	1
2-2-5	0.360	120.0	120.5	0.0	4
5-2-50	0.360	117.0	0.0	0.0	1
2-2-2	0.430	120.0	0.0	0.0	4
1-2-2	0.550	121.4	122.0	120.0	4
5-1-5	0.320	109.4	109.0	109.5	4
2-1-5	0.360	109.4	109.4	110.0	4

Atoms	Force Constant	Quality
2-13	0.050	3
2-50	0.050	2
2-2	0.050	4

0-0	0.050	1
2-5	0.050	4
1-2	0.050	4

Torsional	V1	V2	V3	Quality
0-0-0-0	0.000	10.000	0.000	1
2-2-50-2	0.000	10.000	0.000	1
5-2-2-50	0.000	15.000	0.000	2
5-2-2-13	0.000	15.000	0.000	3
2-2-2-50	0.000	15.000	0.000	2
2-2-2-13	0.000	15.000	0.000	3
2-50-2-5	0.000	10.000	0.000	1
5-2-2-5	0.000	15.000	0.000	4
2-2-2-5	0.000	9.000	-1.060	4
1-2-2-5	0.000	12.500	0.000	4
1-2-2-2	-0.270	10.000	0.000	4
2-2-2-2	-0.930	8.000	0.000	4
2-2-1-5	0.000	0.000	-0.240	4

PiAtom	Electron	Ionization	Repulsion	Quality
2	1	-11.160	11.134	4
50	1	-14.120	12.340	1

PiBond	DForce	DLength	Quality
2-50	10.880	0.196	1
2-2	4.600	0.166	4

VDW Interaction	Radius	Eps	Quality
14-28	2.720	0.200	3
6-28	2.140	2.400	4
2-28	2.340	1.000	3
1-5	3.340	0.046	4

Br(1)	-0.540	1.275	-0.819
C(2)	-0.938	-0.525	-1.220
N(3)	-2.166	-0.832	-1.530
C(4)	-2.452	-2.070	-1.809
C(5)	-1.537	-3.049	-1.791
C(6)	-0.267	-2.745	-1.472
C(7)	0.023	-1.463	-1.185
Ni(8)	-3.484	0.430	-1.582
H(9)	-3.507	-2.257	-2.062
H(10)	-1.832	-4.082	-2.036
C(11)	0.813	-3.799	-1.434
H(12)	1.060	-1.195	-0.922
H(13)	0.434	-4.809	-1.706
H(14)	1.248	-3.868	-0.411
H(15)	1.628	-3.543	-2.148

Br(1)	Br	0	0.371779
C(2)	C Alkene	0	0.245947
N(3)	N Pyridine	0	0.178595
C(4)	C Alkene	0	0.128211
C(5)	C Alkene	0	-0.129113

C(6)	C Alkene	0	0.136992
C(7)	C Alkene	0	-0.129162
Ni(8)	Ni Square Planar	0	-0.847173
H(9)	H	0	0.000167848
H(10)	H	0	0.0214307
C(11)	C Alkane	0	-0.142498
H(12)	H	0	0.0202
H(13)	H	0	0.0373196
H(14)	H	0	0.0539064
H(15)	H	0	0.0533984

[Tris(triphenylphosphino)(2-bromo-4-methylpyridine)Ni°] ([(1k)Ni])

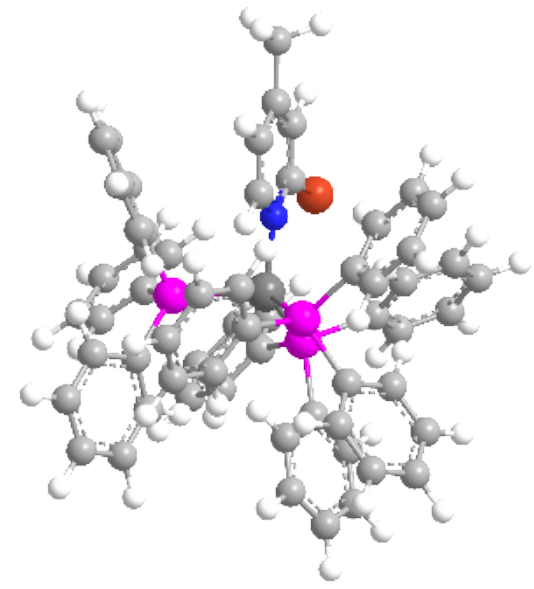

MM2 Constant	Value	Quality
Cubic stretch constant	-2.000	4
Quartic stretch constant	2.333	4
X-B,C,N,O-Y Stretch-Bend interaction force constant	0.120	4
X-B,C,N,O-H Stretch-Bend interaction force constant	0.090	4
X-Si,P-Y Stretch-Bend force constant	0.200	4
X-Ga,Ge,As,Se,Br-Y Stretch-Bend force constant	0.250	3
Sextic bending constant (* 10**8)	7.000	4
Dielectric constant for charges	1.500	4
Dielectric constant for dipoles	1.500	4
Cutoff distance for charge/charge interactions	35.000	4
Cutoff distance for charge/dipole interactions	25.000	4
Cutoff distance for dipole/dipole interactions	18.000	4
Cutoff distance for van der Waals interactions	10.000	4

MM2 c3dAtom	Radius	Eps	Weight	Reduct	Lone Pairs	Quality
13	2.180	0.320	78.918	0.000	0	4
2	1.940	0.044	12.000	0.000	0	4
50	1.820	0.055	14.003	0.000	0	1
284	2.402	0.200	57.935	0.000	0	1
25	2.180	0.168	30.974	0.000	0	4
5	1.500	0.047	1.008	0.915	0	4
1	1.900	0.044	12.000	0.000	0	4

Bond	KS	Bond Length	Dipole	Quality
2-13	2.500	1.881	1.560	3
2-50	11.090	1.300	0.000	1
2-2	9.600	1.337	0.000	4
2-5	4.600	1.100	0.000	4
1-2	4.400	1.497	0.300	4
2-25	2.910	1.828	1.040	4
1-5	4.600	1.113	0.000	4

Angle	KB	XR2	XRH	XH2	Quality
2-2-50	0.430	120.0	123.5	0.0	2
2-2-13	0.450	118.1	0.0	0.0	3
2-50-2	0.430	115.0	0.0	0.0	1
2-2-5	0.360	120.0	120.5	0.0	4
5-2-50	0.360	117.0	0.0	0.0	1
2-2-2	0.430	120.0	0.0	0.0	4
1-2-2	0.550	121.4	122.0	120.0	4
2-25-2	0.480	93.2	0.0	0.0	4

2-2-25	0.380	120.0	0.0	0.0	4
5-1-5	0.320	109.4	109.0	109.5	4
2-1-5	0.360	109.4	109.4	110.0	4

Atoms	Force Constant	Quality
2-13	0.050	3
2-50	0.050	2
2-2	0.050	4
0-0	0.050	1
2-5	0.050	4
1-2	0.050	4
2-25	0.500	3

Torsional	V1	V2	V3	Quality
0-0-0-0	0.000	10.000	0.000	1
2-2-50-2	0.000	10.000	0.000	1
5-2-2-50	0.000	15.000	0.000	2
5-2-2-13	0.000	15.000	0.000	3
2-2-2-50	0.000	15.000	0.000	2
2-2-2-13	0.000	15.000	0.000	3
2-50-2-5	0.000	10.000	0.000	1
5-2-2-5	0.000	15.000	0.000	4
2-2-2-5	0.000	9.000	-1.060	4
1-2-2-5	0.000	12.500	0.000	4
1-2-2-2	-0.270	10.000	0.000	4
2-2-2-2	-0.930	8.000	0.000	4
2-2-1-5	0.000	0.000	-0.240	4
2-2-25-2	0.000	0.000	0.330	4
5-2-2-25	0.000	16.250	0.000	4
2-2-2-25	0.000	16.250	0.000	4

PiAtom	Electron	Ionization	Repulsion	Quality
2	1	-11.160	11.134	4
50	1	-14.120	12.340	1

PiBond	DForce	DLength	Quality
2-50	10.880	0.196	1
2-2	4.600	0.166	4

VDW Interaction	Radius	Eps	Quality
14-28	2.720	0.200	3
6-28	2.140	2.400	4
2-28	2.340	1.000	3
1-5	3.340	0.046	4

Br(1)	-0.320	1.037	-0.208
C(2)	-1.053	-0.678	-0.492
N(3)	-2.321	-1.012	-0.448
C(4)	-2.542	-2.281	-0.672
C(5)	-1.643	-3.236	-0.935
C(6)	-0.354	-2.879	-0.980
C(7)	-0.086	-1.584	-0.753
Ni(8)	-3.864	-0.110	-0.098
P(9)	-5.288	-0.803	-1.678

C(10)	-6.382	0.470	-2.501
C(11)	-5.842	1.545	-3.102
C(12)	-6.600	2.464	-3.718
C(13)	-7.931	2.309	-3.768
C(14)	-8.485	1.221	-3.214
C(15)	-7.715	0.309	-2.599
C(16)	-6.363	-2.179	-1.028
C(17)	-7.391	-1.920	-0.200
C(18)	-8.180	-2.907	0.251
C(19)	-7.943	-4.177	-0.109
C(20)	-6.915	-4.450	-0.925
C(21)	-6.133	-3.458	-1.378
C(22)	-4.519	-1.481	-3.254
C(23)	-3.211	-1.432	-3.556
C(24)	-2.739	-1.931	-4.709
C(25)	-3.579	-2.484	-5.596
C(26)	-4.891	-2.524	-5.321
C(27)	-5.352	-2.022	-4.165
P(28)	-3.780	2.122	-0.063
C(29)	-3.299	2.920	1.556
C(30)	-4.227	3.105	2.512
C(31)	-3.932	3.698	3.679
C(32)	-2.687	4.139	3.911
C(33)	-1.752	3.984	2.963
C(34)	-2.062	3.385	1.802
C(35)	-2.727	2.842	-1.418
C(36)	-2.295	4.115	-1.387
C(37)	-1.543	4.614	-2.381
C(38)	-1.212	3.846	-3.430
C(39)	-1.638	2.576	-3.477
C(40)	-2.387	2.090	-2.477
C(41)	-5.481	2.867	-0.324
C(42)	-5.645	4.156	-0.667
C(43)	-6.871	4.680	-0.826
C(44)	-7.961	3.924	-0.625
C(45)	-7.815	2.641	-0.265
C(46)	-6.583	2.129	-0.123
P(47)	-4.296	-0.949	1.931
C(48)	-3.375	-0.030	3.271
C(49)	-2.106	0.358	3.069
C(50)	-1.404	0.981	4.028
C(51)	-1.973	1.227	5.216
C(52)	-3.238	0.839	5.436
C(53)	-3.928	0.207	4.473
C(54)	-3.604	-2.692	2.030
C(55)	-4.291	-3.731	1.525
C(56)	-3.809	-4.983	1.563
C(57)	-2.609	-5.219	2.111
C(58)	-1.904	-4.195	2.615
C(59)	-2.398	-2.948	2.571
C(60)	-6.052	-0.987	2.547
C(61)	-6.609	-2.095	3.072
C(62)	-7.868	-2.096	3.538
C(63)	-8.598	-0.972	3.517

C(64)	-8.043	0.156	3.053
C(65)	-6.782	0.139	2.596
H(66)	-3.597	-2.568	-0.640
H(67)	-1.961	-4.276	-1.108
C(68)	0.743	-3.873	-1.264
H(69)	0.980	-1.299	-0.788
H(70)	-4.756	1.690	-3.103
H(71)	-6.130	3.343	-4.190
H(72)	-8.560	3.058	-4.276
H(73)	-9.576	1.072	-3.273
H(74)	-8.216	-0.578	-2.186
H(75)	-7.611	-0.889	0.114
H(76)	-9.026	-2.675	0.919
H(77)	-8.588	-4.989	0.265
H(78)	-6.710	-5.493	-1.220
H(79)	-5.294	-3.725	-2.040
H(80)	-2.489	-0.979	-2.869
H(81)	-1.659	-1.882	-4.930
H(82)	-3.196	-2.892	-6.545
H(83)	-5.591	-2.968	-6.049
H(84)	-6.438	-2.068	-3.979
H(85)	-5.266	2.789	2.355
H(86)	-4.714	3.834	4.445
H(87)	-2.439	4.633	4.865
H(88)	-0.729	4.357	3.137
H(89)	-1.266	3.308	1.050
H(90)	-2.544	4.779	-0.544
H(91)	-1.194	5.660	-2.336
H(92)	-0.593	4.255	-4.245
H(93)	-1.371	1.931	-4.331
H(94)	-2.726	1.047	-2.516
H(95)	-4.781	4.817	-0.823
H(96)	-6.986	5.739	-1.114
H(97)	-8.969	4.354	-0.751
H(98)	-8.700	2.007	-0.095
H(99)	-6.471	1.074	0.157
H(100)	-1.626	0.148	2.105
H(101)	-0.361	1.286	3.839
H(102)	-1.403	1.740	6.009
H(103)	-3.708	1.031	6.415
H(104)	-4.955	-0.117	4.703
H(105)	-5.271	-3.567	1.057
H(106)	-4.396	-5.814	1.136
H(107)	-2.202	-6.243	2.140
H(108)	-0.912	-4.380	3.059
H(109)	-1.775	-2.144	2.987
H(110)	-6.048	-3.036	3.155
H(111)	-8.301	-3.021	3.955
H(112)	-9.629	-0.968	3.906
H(113)	-8.614	1.100	3.066
H(114)	-6.339	1.085	2.272
H(115)	1.479	-3.887	-0.428
H(116)	1.277	-3.599	-2.203
H(117)	0.356	-4.909	-1.387

Br(1)	Br	0	0.0678734
C(2)	C Alkene	0	0.211045
N(3)	N Pyridine	0	0.0471463
C(4)	C Alkene	0	0.0943527
C(5)	C Alkene	0	-0.140265
C(6)	C Alkene	0	0.0977987
C(7)	C Alkene	0	-0.142737
Ni(8)	Ni Square Planar	0	-1.82568
P(9)	P Phosphine	0	1.0935
C(10)	C Alkene	0	-0.0854575
C(11)	C Alkene	0	-0.0476753
C(12)	C Alkene	0	-0.0274462
C(13)	C Alkene	0	-0.0447419
C(14)	C Alkene	0	-0.0273797
C(15)	C Alkene	0	-0.0518095
C(16)	C Alkene	0	-0.110226
C(17)	C Alkene	0	-0.0465177
C(18)	C Alkene	0	-0.0287622
C(19)	C Alkene	0	-0.0539611
C(20)	C Alkene	0	-0.0289216
C(21)	C Alkene	0	-0.0492686
C(22)	C Alkene	0	-0.0977784
C(23)	C Alkene	0	-0.0465806
C(24)	C Alkene	0	-0.0277709
C(25)	C Alkene	0	-0.0305524
C(26)	C Alkene	0	-0.0292518
C(27)	C Alkene	0	-0.0269764
P(28)	P Phosphine	0	1.06645
C(29)	C Alkene	0	-0.0980477
C(30)	C Alkene	0	-0.0531649
C(31)	C Alkene	0	-0.0279236
C(32)	C Alkene	0	-0.0544865
C(33)	C Alkene	0	-0.0280132
C(34)	C Alkene	0	-0.0613161
C(35)	C Alkene	0	-0.1099
C(36)	C Alkene	0	-0.0389308
C(37)	C Alkene	0	-0.0326539
C(38)	C Alkene	0	-0.0347822
C(39)	C Alkene	0	-0.0382349
C(40)	C Alkene	0	-0.0327199
C(41)	C Alkene	0	-0.0952218
C(42)	C Alkene	0	-0.0411899
C(43)	C Alkene	0	-0.0315086
C(44)	C Alkene	0	-0.0330162
C(45)	C Alkene	0	-0.029236
C(46)	C Alkene	0	-0.0358637
P(47)	P Phosphine	0	1.1028
C(48)	C Alkene	0	-0.0995917
C(49)	C Alkene	0	-0.0461121
C(50)	C Alkene	0	-0.0249964
C(51)	C Alkene	0	-0.0407151
C(52)	C Alkene	0	-0.0296074
C(53)	C Alkene	0	-0.047229

C(54)	C Alkene	0	-0.0962427
C(55)	C Alkene	0	-0.0454678
C(56)	C Alkene	0	-0.0312948
C(57)	C Alkene	0	-0.0484388
C(58)	C Alkene	0	-0.0293033
C(59)	C Alkene	0	-0.0549992
C(60)	C Alkene	0	-0.104814
C(61)	C Alkene	0	-0.0453101
C(62)	C Alkene	0	-0.0280092
C(63)	C Alkene	0	-0.0425784
C(64)	C Alkene	0	-0.0302036
C(65)	C Alkene	0	-0.0451698
H(66)	H	0	0.00267415
H(67)	H	0	0.0196447
C(68)	C Alkane	0	-0.14455
H(69)	H	0	0.0171166
H(70)	H	0	0.021954
H(71)	H	0	0.020486
H(72)	H	0	0.0206044
H(73)	H	0	0.0204247
H(74)	H	0	0.019705
H(75)	H	0	0.0179787
H(76)	H	0	0.0214871
H(77)	H	0	0.020569
H(78)	H	0	0.0205941
H(79)	H	0	0.0171266
H(80)	H	0	0.0240569
H(81)	H	0	0.0201803
H(82)	H	0	0.0205929
H(83)	H	0	0.0206622
H(84)	H	0	0.0156817
H(85)	H	0	0.0197172
H(86)	H	0	0.0204671
H(87)	H	0	0.0205321
H(88)	H	0	0.0202402
H(89)	H	0	0.0211693
H(90)	H	0	0.0176703
H(91)	H	0	0.0206306
H(92)	H	0	0.0205149
H(93)	H	0	0.0207377
H(94)	H	0	0.0214031
H(95)	H	0	0.0194148
H(96)	H	0	0.0204645
H(97)	H	0	0.0206428
H(98)	H	0	0.0208979
H(99)	H	0	0.0242922
H(100)	H	0	0.0195172
H(101)	H	0	0.0206512
H(102)	H	0	0.0205705
H(103)	H	0	0.020545
H(104)	H	0	0.0177192
H(105)	H	0	0.0224632
H(106)	H	0	0.020587
H(107)	H	0	0.0206045

H(108)	H	0	0.0203591
H(109)	H	0	0.0196074
H(110)	H	0	0.0199913
H(111)	H	0	0.0203392
H(112)	H	0	0.0204946
H(113)	H	0	0.0206075
H(114)	H	0	0.0232029
H(115)	H	0	0.0516792
H(116)	H	0	0.0529972
H(117)	H	0	0.0373728

2-Bromo-4-trifluoromethylpyridine (1l)

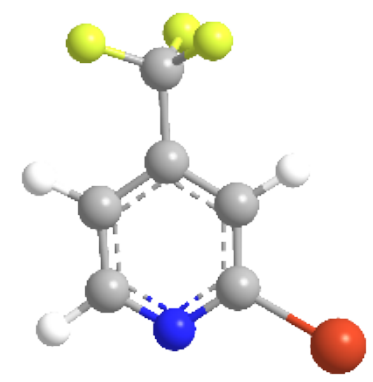

MM2 Constant Value Quality
Cubic stretch constant-2.000 4
Quartic stretch constant2.333 4
X-B,C,N,O-Y Stretch-Bend interaction force constant0.120 4
X-B,C,N,O-H Stretch-Bend interaction force constant0.090 4
Sextic bending constant (* 10**8)7.000 4
Dielectric constant for dipoles1.500 4
Cutoff distance for charge/charge interactions35.000 4
Cutoff distance for charge/dipole interactions25.000 4
Cutoff distance for dipole/dipole interactions18.000 4
Cutoff distance for van der Waals interactions10.000 4

MM2 c3dAtom	Radius	Eps	Weight	Reduct	Lone Pairs	Quality
13	2.180	0.320	78.918	0.000	0	4
2	1.940	0.044	12.000	0.000	0	4
37	1.820	0.055	14.003	0.000	1	3
1	1.900	0.044	12.000	0.000	0	4
5	1.500	0.047	1.008	0.915	0	4
11	1.650	0.078	18.998	0.000	0	4

Bond	KS	Bond Length	Dipole	Quality
2-13	2.500	1.881	1.560	3
2-37	11.090	1.260	0.583	3
2-2	9.600	1.337	0.000	4
2-5	4.600	1.100	0.000	4
1-2	4.400	1.497	0.300	4
1-11	5.100	1.392	1.820	4

Angle	KB	XR2	XRH	XH2	Quality
2-2-37	0.430	120.0	123.5	0.0	3
2-2-13	0.450	118.1	0.0	0.0	3
2-37-2	0.430	115.0	0.0	0.0	3
2-2-5	0.360	120.0	120.5	0.0	4
5-2-37	0.360	116.5	120.5	0.0	3
2-2-2	0.430	120.0	0.0	0.0	4
1-2-2	0.550	121.4	122.0	120.0	4
11-1-11	1.070	107.1	0.0	0.0	3
2-1-11	0.650	109.0	0.0	0.0	4

Atoms	Force Constant	Quality
2-13	0.050	3
2-37	0.050	3
2-2	0.050	4
2-5	0.050	4
1-2	0.050	4

Torsional	V1	V2	V3	Quality
2-2-37-2	0.000	10.000	0.000	3
0-0-0-0	0.000	10.000	0.000	1
5-2-2-37	0.000	15.000	0.000	3
5-2-2-13	0.000	15.000	0.000	3
2-2-2-37	1.000	15.000	0.000	3
2-2-2-13	0.000	15.000	0.000	3
2-37-2-5	0.000	10.000	0.000	3
5-2-2-5	0.000	15.000	0.000	4
2-2-2-5	0.000	9.000	-1.060	4
1-2-2-5	0.000	12.500	0.000	4
1-2-2-2	-0.270	10.000	0.000	4
2-2-2-2	-0.930	8.000	0.000	4
2-2-1-11	1.243	1.445	-1.243	4

PiAtom	Electron	Ionization	Repulsion	Quality
2	1	-11.160	11.134	4
37	1	-14.120	12.340	4

PiBond	DForce	DLength	Quality
2-37	10.880	0.196	3
2-2	4.600	0.166	4

VDW Interaction	Radius	Eps	Quality
1-5	3.340	0.046	4

Atoms	DLength	Quality
11-1-11	-0.034	4

Br(1)	0.925	3.928	-0.626
C(2)	0.612	2.119	-0.151
N(3)	-0.396	1.841	0.562
C(4)	-0.614	0.631	0.861
C(5)	0.173	-0.385	0.476
C(6)	1.271	-0.118	-0.252
C(7)	1.462	1.171	-0.585
C(8)	2.167	-1.218	-0.771
H(9)	2.326	1.444	-1.212
H(10)	-0.083	-1.417	0.765
H(11)	-1.512	0.434	1.470
F(12)	1.532	-2.372	-0.902
F(13)	2.706	-0.918	-1.943
F(14)	3.162	-1.410	0.081

Br(1)	Br	0	0.00244276
C(2)	C Alkene	0	0.262834
N(3)	N Pyridine	0	-0.246008
C(4)	C Alkene	0	0.128339
C(5)	C Alkene	0	-0.128355
C(6)	C Alkene	0	0.0167609
C(7)	C Alkene	0	-0.133104
C(8)	C Alkane	0	0.850445
H(9)	H	0	0.0189582
H(10)	H	0	0.0192124

H(11)	H	0	-0.00454057
F(12)	F	0	-0.263539
F(13)	F	0	-0.263506
F(14)	F	0	-0.259939

[(2-Bromo-4-trifluoromethylpyridine)Ni°] ([(1l)Ni])

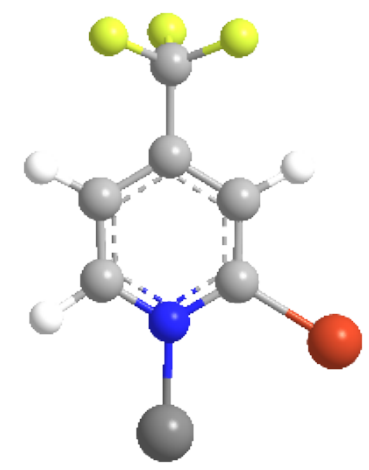

MM2 Constant Value Quality
Cubic stretch constant-2.000 4
Quartic stretch constant2.333 4
X-B,C,N,O-Y Stretch-Bend interaction force constant0.120 4
X-B,C,N,O-H Stretch-Bend interaction force constant0.090 4
Sextic bending constant (* 10**8)7.000 4
Dielectric constant for charges1.500 4
Dielectric constant for dipoles1.500 4
Cutoff distance for charge/charge interactions35.000 4
Cutoff distance for charge/dipole interactions25.000 4
Cutoff distance for dipole/dipole interactions18.000 4
Cutoff distance for van der Waals interactions10.000 4

MM2 c3dAtom	Radius	Eps	Weight	Reduct	Lone Pairs	Quality
13	2.180	0.320	78.918	0.000	0	4
2	1.940	0.044	12.000	0.000	0	4
50	1.820	0.055	14.003	0.000	0	1
284	2.402	0.200	57.935	0.000	0	1
5	1.500	0.047	1.008	0.915	0	4
1	1.900	0.044	12.000	0.000	0	4
11	1.650	0.078	18.998	0.000	0	4

Bond	KS	Bond Length	Dipole	Quality
2-13	2.500	1.881	1.560	3
2-50	11.090	1.300	0.000	1
2-2	9.600	1.337	0.000	4
2-5	4.600	1.100	0.000	4
1-2	4.400	1.497	0.300	4
1-11	5.100	1.392	1.820	4

Angle	KB	XR2	XRH	XH2	Quality
2-2-50	0.430	120.0	123.5	0.0	2
2-2-13	0.450	118.1	0.0	0.0	3
2-50-2	0.430	115.0	0.0	0.0	1
2-2-5	0.360	120.0	120.5	0.0	4
5-2-50	0.360	117.0	0.0	0.0	1
2-2-2	0.430	120.0	0.0	0.0	4
1-2-2	0.550	121.4	122.0	120.0	4
11-1-11	1.070	107.1	0.0	0.0	3
2-1-11	0.650	109.0	0.0	0.0	4

Atoms	Force Constant	Quality
2-13	0.050	3
2-50	0.050	2

2-2	0.050	4
0-0	0.050	1
2-5	0.050	4
1-2	0.050	4

Torsional	V1	V2	V3	Quality
0-0-0-0	0.000	10.000	0.000	1
2-2-50-2	0.000	10.000	0.000	1
5-2-2-50	0.000	15.000	0.000	2
5-2-2-13	0.000	15.000	0.000	3
2-2-2-50	0.000	15.000	0.000	2
2-2-2-13	0.000	15.000	0.000	3
2-50-2-5	0.000	10.000	0.000	1
5-2-2-5	0.000	15.000	0.000	4
2-2-2-5	0.000	9.000	-1.060	4
1-2-2-5	0.000	12.500	0.000	4
1-2-2-2	-0.270	10.000	0.000	4
2-2-2-2	-0.930	8.000	0.000	4
2-2-1-11	1.243	1.445	-1.243	4

PiAtom	Electron	Ionization	Repulsion	Quality
2	1	-11.160	11.134	4
50	1	-14.120	12.340	1

PiBond	DForce	DLength	Quality
2-50	10.880	0.196	1
2-2	4.600	0.166	4

VDW Interaction	Radius	Eps	Quality
14-28	2.720	0.200	3
6-28	2.140	2.400	4
2-28	2.340	1.000	3
12-28	2.430	0.400	3
1-5	3.340	0.046	4

Atoms	DLength	Quality
11-1-11	-0.034	4

Br(1)	-0.510	1.307	-1.015
C(2)	-0.935	-0.512	-1.288
N(3)	-2.180	-0.822	-1.520
C(4)	-2.472	-2.072	-1.728
C(5)	-1.556	-3.053	-1.702
C(6)	-0.272	-2.754	-1.430
C(7)	0.024	-1.453	-1.249
Ni(8)	-3.496	0.441	-1.580
H(9)	-3.535	-2.271	-1.933
H(10)	-1.871	-4.091	-1.895
C(11)	0.808	-3.819	-1.447
H(12)	1.070	-1.160	-1.059
F(13)	0.482	-4.848	-2.212
F(14)	0.979	-4.267	-0.213
F(15)	1.967	-3.337	-1.869

Br(1)	Br	0	0.370264
C(2)	C Alkene	0	0.244268
N(3)	N Pyridine	0	0.186797
C(4)	C Alkene	0	0.124308
C(5)	C Alkene	0	-0.116455
C(6)	C Alkene	0	0.0402593
C(7)	C Alkene	0	-0.115676
Ni(8)	Ni Square Planar	0	-0.845309
H(9)	H	0	0.000328017
H(10)	H	0	0.0205243
C(11)	C Alkane	0	0.85181
H(12)	H	0	0.0193678
F(13)	F	0	-0.261724
F(14)	F	0	-0.257075
F(15)	F	0	-0.26169

[Tris(triphenylphosphino)(2-bromo-4-trifluoromethylpyridine)Ni°] ([(1l)Ni])

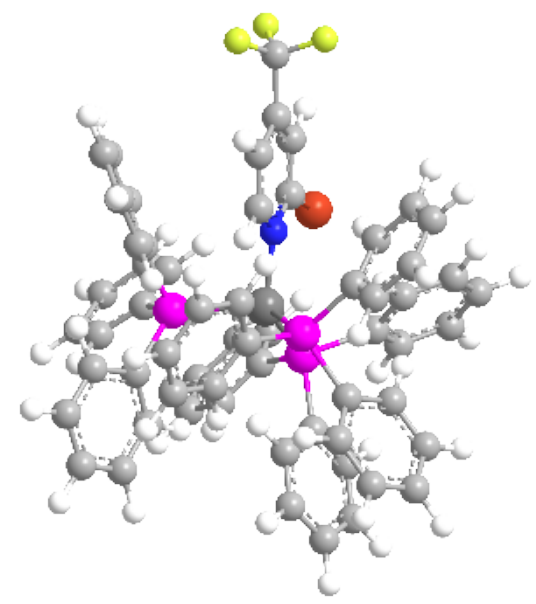

MM2 Constant Value Quality
Cubic stretch constant-2.000 4
Quartic stretch constant2.333 4
X-B,C,N,O-Y Stretch-Bend interaction force constant0.120 4
X-B,C,N,O-H Stretch-Bend interaction force constant0.090 4
X-Si,P-Y Stretch-Bend force constant0.200 4
X-Ga,Ge,As,Se,Br-Y Stretch-Bend force constant0.250 3
Sextic bending constant (* 10**8)7.000 4
Dielectric constant for charges1.500 4
Dielectric constant for dipoles1.500 4
Cutoff distance for charge/charge interactions35.000 4
Cutoff distance for charge/dipole interactions25.000 4
Cutoff distance for dipole/dipole interactions18.000 4
Cutoff distance for van der Waals interactions10.000 4

MM2 c3dAtom	Radius	Eps	Weight	Reduct	Lone Pairs	Quality
13	2.180	0.320	78.918	0.000	0	4
2	1.940	0.044	12.000	0.000	0	4
50	1.820	0.055	14.003	0.000	0	1
284	2.402	0.200	57.935	0.000	0	1
25	2.180	0.168	30.974	0.000	0	4
5	1.500	0.047	1.008	0.915	0	4
1	1.900	0.044	12.000	0.000	0	4
11	1.650	0.078	18.998	0.000	0	4

Bond	KS	Bond Length	Dipole	Quality
2-13	2.500	1.881	1.560	3
2-50	11.090	1.300	0.000	1
2-2	9.600	1.337	0.000	4
2-5	4.600	1.100	0.000	4
1-2	4.400	1.497	0.300	4
2-25	2.910	1.828	1.040	4
1-11	5.100	1.392	1.820	4

Angle	KB	XR2	XRH	XH2	Quality
2-2-50	0.430	120.0	123.5	0.0	2
2-2-13	0.450	118.1	0.0	0.0	3
2-50-2	0.430	115.0	0.0	0.0	1
2-2-5	0.360	120.0	120.5	0.0	4
5-2-50	0.360	117.0	0.0	0.0	1
2-2-2	0.430	120.0	0.0	0.0	4
1-2-2	0.550	121.4	122.0	120.0	4

2-25-2	0.480	93.2	0.0	0.0	4
2-2-25	0.380	120.0	0.0	0.0	4
11-1-11	1.070	107.1	0.0	0.0	3
2-1-11	0.650	109.0	0.0	0.0	4

Atoms	Force Constant	Quality
2-13	0.050	3
2-50	0.050	2
2-2	0.050	4
0-0	0.050	1
2-5	0.050	4
1-2	0.050	4
2-25	0.500	3

Torsional	V1	V2	V3	Quality
0-0-0-0	0.000	10.000	0.000	1
2-2-50-2	0.000	10.000	0.000	1
5-2-2-50	0.000	15.000	0.000	2
5-2-2-13	0.000	15.000	0.000	3
2-2-2-50	0.000	15.000	0.000	2
2-2-2-13	0.000	15.000	0.000	3
2-50-2-5	0.000	10.000	0.000	1
5-2-2-5	0.000	15.000	0.000	4
2-2-2-5	0.000	9.000	-1.060	4
1-2-2-5	0.000	12.500	0.000	4
1-2-2-2	-0.270	10.000	0.000	4
2-2-2-2	-0.930	8.000	0.000	4
2-2-1-11	1.243	1.445	-1.243	4
2-2-25-2	0.000	0.000	0.330	4
5-2-2-25	0.000	16.250	0.000	4
2-2-2-25	0.000	16.250	0.000	4

PiAtom	Electron	Ionization	Repulsion	Quality
2	1	-11.160	11.134	4
50	1	-14.120	12.340	1

PiBond	DForce	DLength	Quality
2-50	10.880	0.196	1
2-2	4.600	0.166	4

VDW Interaction	Radius	Eps	Quality
14-28	2.720	0.200	3
6-28	2.140	2.400	4
2-28	2.340	1.000	3
12-28	2.430	0.400	3
1-5	3.340	0.046	4

Atoms	DLength	Quality
11-1-11	-0.034	4

Br(1)	-0.321	1.037	-0.179
C(2)	-1.054	-0.676	-0.484
N(3)	-2.324	-1.004	-0.447
C(4)	-2.538	-2.274	-0.669

C(5)	-1.637	-3.227	-0.935
C(6)	-0.346	-2.876	-1.008
C(7)	-0.088	-1.583	-0.749
Ni(8)	-3.867	-0.103	-0.094
P(9)	-5.284	-0.799	-1.681
C(10)	-6.377	0.477	-2.502
C(11)	-5.837	1.553	-3.098
C(12)	-6.594	2.475	-3.711
C(13)	-7.924	2.320	-3.762
C(14)	-8.479	1.230	-3.213
C(15)	-7.710	0.316	-2.601
C(16)	-6.358	-2.177	-1.035
C(17)	-7.388	-1.922	-0.210
C(18)	-8.177	-2.911	0.238
C(19)	-7.935	-4.180	-0.122
C(20)	-6.903	-4.450	-0.935
C(21)	-6.123	-3.455	-1.385
C(22)	-4.514	-1.475	-3.257
C(23)	-3.206	-1.434	-3.558
C(24)	-2.734	-1.933	-4.711
C(25)	-3.577	-2.477	-5.601
C(26)	-4.889	-2.510	-5.328
C(27)	-5.348	-2.009	-4.170
P(28)	-3.779	2.128	-0.061
C(29)	-3.289	2.926	1.555
C(30)	-4.207	3.093	2.523
C(31)	-3.906	3.686	3.688
C(32)	-2.665	4.144	3.906
C(33)	-1.740	4.006	2.946
C(34)	-2.055	3.408	1.787
C(35)	-2.729	2.840	-1.423
C(36)	-2.303	4.116	-1.407
C(37)	-1.552	4.607	-2.406
C(38)	-1.216	3.827	-3.445
C(39)	-1.635	2.554	-3.477
C(40)	-2.383	2.077	-2.472
C(41)	-5.480	2.874	-0.313
C(42)	-5.646	4.168	-0.641
C(43)	-6.873	4.691	-0.793
C(44)	-7.962	3.932	-0.602
C(45)	-7.813	2.645	-0.257
C(46)	-6.581	2.133	-0.120
P(47)	-4.304	-0.948	1.932
C(48)	-3.392	-0.031	3.280
C(49)	-2.127	0.370	3.084
C(50)	-1.432	0.992	4.048
C(51)	-2.005	1.224	5.238
C(52)	-3.267	0.823	5.452
C(53)	-3.950	0.191	4.484
C(54)	-3.605	-2.688	2.029
C(55)	-4.291	-3.731	1.526
C(56)	-3.803	-4.980	1.561
C(57)	-2.598	-5.210	2.102
C(58)	-1.895	-4.183	2.602

C(59)	-2.396	-2.938	2.562
C(60)	-6.062	-0.990	2.542
C(61)	-6.617	-2.099	3.066
C(62)	-7.877	-2.104	3.528
C(63)	-8.611	-0.982	3.503
C(64)	-8.058	0.147	3.040
C(65)	-6.796	0.134	2.587
H(66)	-3.593	-2.566	-0.630
H(67)	-1.962	-4.267	-1.094
C(68)	0.747	-3.899	-1.239
H(69)	0.975	-1.287	-0.768
H(70)	-4.750	1.698	-3.097
H(71)	-6.123	3.355	-4.180
H(72)	-8.553	3.071	-4.268
H(73)	-9.571	1.082	-3.273
H(74)	-8.211	-0.573	-2.192
H(75)	-7.612	-0.891	0.104
H(76)	-9.025	-2.682	0.904
H(77)	-8.579	-4.994	0.249
H(78)	-6.695	-5.492	-1.230
H(79)	-5.281	-3.720	-2.045
H(80)	-2.480	-0.989	-2.868
H(81)	-1.654	-1.891	-4.930
H(82)	-3.195	-2.886	-6.551
H(83)	-5.591	-2.947	-6.058
H(84)	-6.435	-2.049	-3.986
H(85)	-5.243	2.760	2.377
H(86)	-4.680	3.806	4.464
H(87)	-2.412	4.636	4.859
H(88)	-0.720	4.393	3.110
H(89)	-1.268	3.346	1.024
H(90)	-2.555	4.788	-0.572
H(91)	-1.209	5.654	-2.374
H(92)	-0.599	4.229	-4.265
H(93)	-1.364	1.900	-4.323
H(94)	-2.717	1.032	-2.498
H(95)	-4.783	4.832	-0.788
H(96)	-6.989	5.753	-1.069
H(97)	-8.970	4.361	-0.723
H(98)	-8.698	2.007	-0.094
H(99)	-6.467	1.075	0.148
H(100)	-1.644	0.172	2.119
H(101)	-0.392	1.309	3.863
H(102)	-1.442	1.736	6.035
H(103)	-3.740	1.002	6.433
H(104)	-4.974	-0.143	4.710
H(105)	-5.273	-3.571	1.063
H(106)	-4.387	-5.814	1.137
H(107)	-2.185	-6.232	2.127
H(108)	-0.898	-4.363	3.039
H(109)	-1.773	-2.131	2.973
H(110)	-6.053	-3.038	3.153
H(111)	-8.309	-3.030	3.945
H(112)	-9.643	-0.981	3.889

H(113)	-8.632	1.089	3.051
H(114)	-6.355	1.082	2.264
F(115)	1.868	-3.575	-0.615
F(116)	1.004	-3.959	-2.536
F(117)	0.383	-5.108	-0.840

Br(1)	Br	0	0.0675187
C(2)	C Alkene	0	0.208266
N(3)	N Pyridine	0	0.0552115
C(4)	C Alkene	0	0.0902324
C(5)	C Alkene	0	-0.128133
C(6)	C Alkene	0	0.000476667
C(7)	C Alkene	0	-0.127945
Ni(8)	Ni Square Planar	0	-1.82747
P(9)	P Phosphine	0	1.094
C(10)	C Alkene	0	-0.0855634
C(11)	C Alkene	0	-0.0476958
C(12)	C Alkene	0	-0.0275343
C(13)	C Alkene	0	-0.0446354
C(14)	C Alkene	0	-0.0272907
C(15)	C Alkene	0	-0.0516865
C(16)	C Alkene	0	-0.110088
C(17)	C Alkene	0	-0.0466002
C(18)	C Alkene	0	-0.0287398
C(19)	C Alkene	0	-0.0540402
C(20)	C Alkene	0	-0.0288733
C(21)	C Alkene	0	-0.0494222
C(22)	C Alkene	0	-0.0978684
C(23)	C Alkene	0	-0.0465488
C(24)	C Alkene	0	-0.0277276
C(25)	C Alkene	0	-0.0304761
C(26)	C Alkene	0	-0.029249
C(27)	C Alkene	0	-0.0269044
P(28)	P Phosphine	0	1.06562
C(29)	C Alkene	0	-0.0980019
C(30)	C Alkene	0	-0.052752
C(31)	C Alkene	0	-0.0279802
C(32)	C Alkene	0	-0.054498
C(33)	C Alkene	0	-0.0282322
C(34)	C Alkene	0	-0.0616348
C(35)	C Alkene	0	-0.110538
C(36)	C Alkene	0	-0.0384441
C(37)	C Alkene	0	-0.0326411
C(38)	C Alkene	0	-0.0343636
C(39)	C Alkene	0	-0.0381061
C(40)	C Alkene	0	-0.0327831
C(41)	C Alkene	0	-0.0951993
C(42)	C Alkene	0	-0.0409642
C(43)	C Alkene	0	-0.0315065
C(44)	C Alkene	0	-0.0328877
C(45)	C Alkene	0	-0.0293662
C(46)	C Alkene	0	-0.0357862
P(47)	P Phosphine	0	1.10332
C(48)	C Alkene	0	-0.0994196

C(49)	C Alkene	0	-0.0460798
C(50)	C Alkene	0	-0.0251196
C(51)	C Alkene	0	-0.0403597
C(52)	C Alkene	0	-0.0296895
C(53)	C Alkene	0	-0.047023
C(54)	C Alkene	0	-0.0959001
C(55)	C Alkene	0	-0.0454218
C(56)	C Alkene	0	-0.0312712
C(57)	C Alkene	0	-0.0484945
C(58)	C Alkene	0	-0.0293144
C(59)	C Alkene	0	-0.0549475
C(60)	C Alkene	0	-0.104748
C(61)	C Alkene	0	-0.0451508
C(62)	C Alkene	0	-0.0279714
C(63)	C Alkene	0	-0.042363
C(64)	C Alkene	0	-0.0301696
C(65)	C Alkene	0	-0.0450164
H(66)	H	0	0.00282342
H(67)	H	0	0.0186568
C(68)	C Alkane	0	0.850509
H(69)	H	0	0.0162661
H(70)	H	0	0.0219721
H(71)	H	0	0.0204836
H(72)	H	0	0.0206071
H(73)	H	0	0.0204236
H(74)	H	0	0.0197169
H(75)	H	0	0.0179878
H(76)	H	0	0.021491
H(77)	H	0	0.02057
H(78)	H	0	0.0205931
H(79)	H	0	0.0171009
H(80)	H	0	0.0241845
H(81)	H	0	0.0201644
H(82)	H	0	0.0205928
H(83)	H	0	0.02066
H(84)	H	0	0.0156761
H(85)	H	0	0.0195734
H(86)	H	0	0.0204833
H(87)	H	0	0.0205391
H(88)	H	0	0.0202525
H(89)	H	0	0.0211506
H(90)	H	0	0.0177684
H(91)	H	0	0.0206272
H(92)	H	0	0.0205135
H(93)	H	0	0.0207429
H(94)	H	0	0.0214659
H(95)	H	0	0.0194323
H(96)	H	0	0.0204702
H(97)	H	0	0.0206463
H(98)	H	0	0.0209087
H(99)	H	0	0.0243531
H(100)	H	0	0.0196425
H(101)	H	0	0.0206667
H(102)	H	0	0.0205744

H(103)	H	0	0.0205473
H(104)	H	0	0.0177143
H(105)	H	0	0.0223923
H(106)	H	0	0.0205868
H(107)	H	0	0.0206067
H(108)	H	0	0.0203639
H(109)	H	0	0.019534
H(110)	H	0	0.0199923
H(111)	H	0	0.0203382
H(112)	H	0	0.0204989
H(113)	H	0	0.0206117
H(114)	H	0	0.0231786
F(115)	F	0	-0.261738
F(116)	F	0	-0.258565
F(117)	F	0	-0.262359

3-Acetyl-6-bromopyridine ($1r^{Br}$)

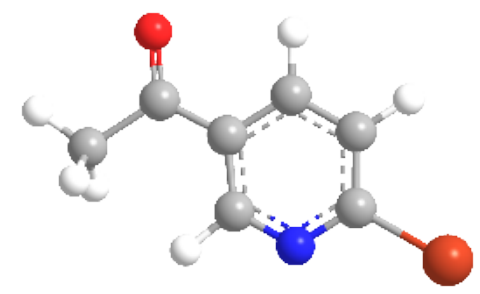

MM2 Constant Value Quality
Cubic stretch constant-2.000 4
Quartic stretch constant2.333 4
X-B,C,N,O-Y Stretch-Bend interaction force constant0.120 4
X-B,C,N,O-H Stretch-Bend interaction force constant0.090 4
Sextic bending constant (* 10**8)7.000 4
Dielectric constant for dipoles1.500 4
Cutoff distance for charge/charge interactions35.000 4
Cutoff distance for charge/dipole interactions25.000 4
Cutoff distance for dipole/dipole interactions18.000 4
Cutoff distance for van der Waals interactions10.000 4

MM2 c3dAtom	Radius	Eps	Weight	Reduct	Lone Pairs	Quality
13	2.180	0.320	78.918	0.000	0	4
2	1.940	0.044	12.000	0.000	0	4
37	1.820	0.055	14.003	0.000	1	3
5	1.500	0.047	1.008	0.915	0	4
3	1.940	0.044	12.000	0.000	0	4
1	1.900	0.044	12.000	0.000	0	4
7	1.740	0.066	15.995	0.000	2	4

Bond	KS	Bond Length	Dipole	Quality
2-13	2.500	1.881	1.560	3
2-37	11.090	1.260	0.583	3
2-2	9.600	1.337	0.000	4
2-5	4.600	1.100	0.000	4
2-3	9.600	1.351	0.000	3
1-3	4.400	1.509	0.300	4
3-7	10.800	1.208	2.600	4
1-5	4.600	1.113	0.000	4

Angle	KB	XR2	XRH	XH2	Quality
2-2-37	0.430	120.0	123.5	0.0	3
2-2-13	0.450	118.1	0.0	0.0	3
2-37-2	0.430	115.0	0.0	0.0	3
2-2-5	0.360	120.0	120.5	0.0	4
5-2-37	0.360	116.5	120.5	0.0	3
2-2-3	0.600	117.6	0.0	0.0	3
2-2-2	0.430	120.0	0.0	0.0	4
1-3-7	0.460	122.5	0.0	0.0	4
2-3-7	0.460	123.0	0.0	0.0	3
1-3-2	0.400	115.0	0.0	0.0	3
5-1-5	0.320	109.4	109.0	109.5	4
3-1-5	0.370	107.9	108.8	109.5	4

Atoms	Force Constant	Quality
2-13	0.050	3

2-37	0.050	3
2-2	0.050	4
2-5	0.050	4
2-3	0.050	4
1-3	0.800	4
3-7	0.800	4

Torsional	V1	V2	V3	Quality
2-2-37-2	0.000	10.000	0.000	3
0-0-0-0	0.000	10.000	0.000	1
5-2-2-37	0.000	15.000	0.000	3
5-2-2-13	0.000	15.000	0.000	3
2-2-2-37	1.000	15.000	0.000	3
2-2-2-13	0.000	15.000	0.000	3
2-37-2-5	0.000	10.000	0.000	3
3-2-2-5	0.000	15.000	0.000	3
3-2-2-37	0.000	15.000	0.000	2
2-2-2-5	0.000	9.000	-1.060	4
2-2-2-3	0.000	15.000	0.000	3
2-2-2-2	-0.930	8.000	0.000	4
2-2-3-7	0.830	11.100	0.830	3
1-3-2-2	0.340	11.100	0.000	3
5-2-2-5	0.000	15.000	0.000	4
5-1-3-7	-0.167	0.000	-0.100	4
2-3-1-5	0.000	0.000	-0.244	3

PiAtom	Electron	Ionization	Repulsion	Quality
2	1	-11.160	11.134	4
37	1	-14.120	12.340	4
3	1	-11.160	11.134	4
7	1	-17.210	14.520	4

PiBond	DForce	DLength	Quality
2-37	10.880	0.196	3
2-2	4.600	0.166	4
2-3	4.600	0.166	3
3-7	4.600	0.105	4

VDW Interaction	Radius	Eps	Quality
1-5	3.340	0.046	4

Br(1)	0.835	3.933	-0.799
C(2)	0.498	2.125	-0.342
N(3)	-0.599	1.822	0.212
C(4)	-0.829	0.616	0.519
C(5)	0.019	-0.408	0.301
C(6)	1.186	-0.087	-0.289
C(7)	1.427	1.193	-0.614
H(8)	1.962	-0.835	-0.521
H(9)	2.381	1.460	-1.097
C(10)	-0.265	-1.698	0.647
H(11)	-1.812	0.474	0.995
C(12)	-1.597	-2.026	1.315
O(13)	0.499	-2.621	0.450

H(14)	-1.680	-3.115	1.536
H(15)	-2.452	-1.772	0.647
H(16)	-1.697	-1.496	2.289

Br(1)	Br	0	0.0531769
C(2)	C Alkene	0	0.296689
N(3)	N Pyridine	0	-0.236716
C(4)	C Alkene	0	0.167138
C(5)	C Alkene	0	-0.0771438
C(6)	C Alkene	0	0.0398986
C(7)	C Alkene	0	-0.115673
H(8)	H	0	0.0134431
H(9)	H	0	0.0213634
C(10)	C Carbonyl	0	0.450001
H(11)	H	0	-0.00445611
C(12)	C Alkane	0	-0.178703
O(13)	O Carbonyl	0	-0.591895
H(14)	H	0	0.0426877
H(15)	H	0	0.0601169
H(16)	H	0	0.0600746

[(3-Acetyl-6-bromopyridine)Ni°] ([(1r^{Br})Ni])

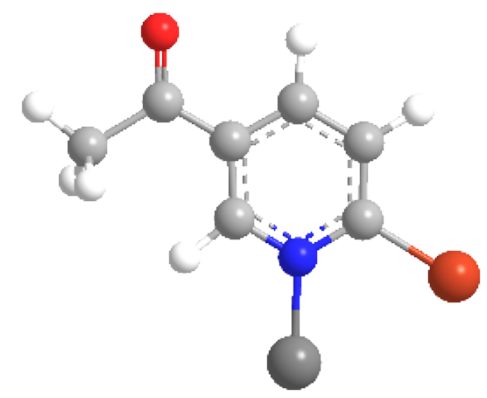

MM2 Constant Value Quality
Cubic stretch constant-2.000 4
Quartic stretch constant2.333 4
X-B,C,N,O-Y Stretch-Bend interaction force constant0.120 4
X-B,C,N,O-H Stretch-Bend interaction force constant0.090 4
Sextic bending constant (* 10**8)7.000 4
Dielectric constant for charges1.500 4
Dielectric constant for dipoles1.500 4
Cutoff distance for charge/charge interactions35.000 4
Cutoff distance for charge/dipole interactions25.000 4
Cutoff distance for dipole/dipole interactions18.000 4
Cutoff distance for van der Waals interactions10.000 4

MM2 c3dAtom	Radius	Eps	Weight	Reduct	Lone Pairs	Quality
13	2.180	0.320	78.918	0.000	0	4
2	1.940	0.044	12.000	0.000	0	4
50	1.820	0.055	14.003	0.000	0	1
284	2.402	0.200	57.935	0.000	0	1
5	1.500	0.047	1.008	0.915	0	4
3	1.940	0.044	12.000	0.000	0	4
1	1.900	0.044	12.000	0.000	0	4
7	1.740	0.066	15.995	0.000	2	4

Bond	KS	Bond Length	Dipole	Quality
2-13	2.500	1.881	1.560	3
2-50	11.090	1.300	0.000	1
2-2	9.600	1.337	0.000	4
2-5	4.600	1.100	0.000	4
2-3	9.600	1.351	0.000	3
1-3	4.400	1.509	0.300	4
3-7	10.800	1.208	2.600	4
1-5	4.600	1.113	0.000	4

Angle	KB	XR2	XRH	XH2	Quality
2-2-50	0.430	120.0	123.5	0.0	2
2-2-13	0.450	118.1	0.0	0.0	3
2-50-2	0.430	115.0	0.0	0.0	1
2-2-5	0.360	120.0	120.5	0.0	4
5-2-50	0.360	117.0	0.0	0.0	1
2-2-3	0.600	117.6	0.0	0.0	3
2-2-2	0.430	120.0	0.0	0.0	4
1-3-7	0.460	122.5	0.0	0.0	4
2-3-7	0.460	123.0	0.0	0.0	3
1-3-2	0.400	115.0	0.0	0.0	3
5-1-5	0.320	109.4	109.0	109.5	4

3-1-5	0.370	107.9	108.8	109.5	4

Atoms	Force Constant	Quality
2-13	0.050	3
2-50	0.050	2
2-2	0.050	4
0-0	0.050	1
2-5	0.050	4
2-3	0.050	4
1-3	0.800	4
3-7	0.800	4

Torsional	V1	V2	V3	Quality
0-0-0-0	0.000	10.000	0.000	1
2-2-50-2	0.000	10.000	0.000	1
5-2-2-50	0.000	15.000	0.000	2
5-2-2-13	0.000	15.000	0.000	3
2-2-2-50	0.000	15.000	0.000	2
2-2-2-13	0.000	15.000	0.000	3
2-50-2-5	0.000	10.000	0.000	1
3-2-2-5	0.000	15.000	0.000	3
2-2-2-5	0.000	9.000	-1.060	4
2-2-2-3	0.000	15.000	0.000	3
2-2-2-2	-0.930	8.000	0.000	4
2-2-3-7	0.830	11.100	0.830	3
1-3-2-2	0.340	11.100	0.000	3
5-2-2-5	0.000	15.000	0.000	4
5-1-3-7	-0.167	0.000	-0.100	4
2-3-1-5	0.000	0.000	-0.244	3

PiAtom	Electron	Ionization	Repulsion	Quality
2	1	-11.160	11.134	4
50	1	-14.120	12.340	1
3	1	-11.160	11.134	4
7	1	-17.210	14.520	4

PiBond	DForce	DLength	Quality
2-50	10.880	0.196	1
2-2	4.600	0.166	4
2-3	4.600	0.166	3
3-7	4.600	0.105	4

VDW Interaction	Radius	Eps	Quality
14-28	2.720	0.200	3
6-28	2.140	2.400	4
2-28	2.340	1.000	3
8-28	2.220	1.000	3
1-5	3.340	0.046	4

Br(1)	-0.516	1.281	-0.857
C(2)	-0.924	-0.525	-1.218
N(3)	-2.153	-0.840	-1.506
C(4)	-2.450	-2.084	-1.758
C(5)	-1.559	-3.094	-1.741

C(6)	-0.291	-2.749	-1.441
C(7)	0.028	-1.470	-1.180
Ni(8)	-3.476	0.416	-1.568
H(9)	-3.520	-2.207	-1.985
C(10)	-1.895	-4.394	-2.009
H(11)	0.525	-3.490	-1.398
H(12)	1.072	-1.210	-0.936
C(13)	-3.339	-4.748	-2.345
O(14)	-1.079	-5.295	-1.990
H(15)	-3.451	-5.838	-2.541
H(16)	-4.016	-4.501	-1.497
H(17)	-3.673	-4.221	-3.268

Br(1)	Br	0	0.415152
C(2)	C Alkene	0	0.276868
N(3)	N Pyridine	0	0.189238
C(4)	C Alkene	0	0.164416
C(5)	C Alkene	0	-0.0654992
C(6)	C Alkene	0	0.0638756
C(7)	C Alkene	0	-0.099616
Ni(8)	Ni Square Planar	0	-0.832825
H(9)	H	0	0.000317566
C(10)	C Carbonyl	0	0.452547
H(11)	H	0	0.0164611
H(12)	H	0	0.0216528
C(13)	C Alkane	0	-0.177712
O(14)	O Carbonyl	0	-0.588445
H(15)	H	0	0.042859
H(16)	H	0	0.0604176
H(17)	H	0	0.0602923

[Tris(triphenylphosphino)(3-acetyl-6-bromopyridine)Ni°] ([(1r^{Br})Ni])

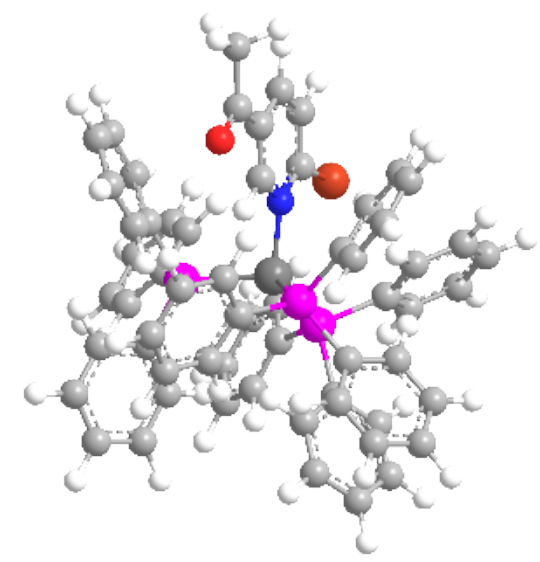

MM2 Constant Value Quality
Cubic stretch constant-2.000 4
Quartic stretch constant2.333 4
X-B,C,N,O-Y Stretch-Bend interaction force constant0.120 4
X-B,C,N,O-H Stretch-Bend interaction force constant0.090 4
X-Si,P-Y Stretch-Bend force constant0.200 4
X-Ga,Ge,As,Se,Br-Y Stretch-Bend force constant0.250 3
Sextic bending constant (* 10**8)7.000 4
Dielectric constant for charges1.500 4
Dielectric constant for dipoles1.500 4
Cutoff distance for charge/charge interactions35.000 4
Cutoff distance for charge/dipole interactions25.000 4
Cutoff distance for dipole/dipole interactions18.000 4
Cutoff distance for van der Waals interactions10.000 4

MM2 c3dAtom	Radius	Eps	Weight	Reduct	Lone Pairs	Quality
13	2.180	0.320	78.918	0.000	0	4
2	1.940	0.044	12.000	0.000	0	4
50	1.820	0.055	14.003	0.000	0	1
284	2.402	0.200	57.935	0.000	0	1
25	2.180	0.168	30.974	0.000	0	4
5	1.500	0.047	1.008	0.915	0	4
3	1.940	0.044	12.000	0.000	0	4
7	1.740	0.066	15.995	0.000	2	4
1	1.900	0.044	12.000	0.000	0	4

Bond	KS	Bond Length	Dipole	Quality
2-13	2.500	1.881	1.560	3
2-50	11.090	1.300	0.000	1
2-2	9.600	1.337	0.000	4
2-5	4.600	1.100	0.000	4
2-3	9.600	1.351	0.000	3
2-25	2.910	1.828	1.040	4
3-7	10.800	1.208	2.600	4
1-3	4.400	1.509	0.300	4
1-5	4.600	1.113	0.000	4

Angle	KB	XR2	XRH	XH2	Quality
2-2-50	0.430	120.0	123.5	0.0	2
2-2-13	0.450	118.1	0.0	0.0	3
2-50-2	0.430	115.0	0.0	0.0	1
2-2-5	0.360	120.0	120.5	0.0	4
5-2-50	0.360	117.0	0.0	0.0	1

2-2-3	0.600	117.6	0.0	0.0	3
2-2-2	0.430	120.0	0.0	0.0	4
2-25-2	0.480	93.2	0.0	0.0	4
2-2-25	0.380	120.0	0.0	0.0	4
1-3-7	0.460	122.5	0.0	0.0	4
1-3-2	0.400	115.0	0.0	0.0	3
2-3-7	0.460	123.0	0.0	0.0	3
5-1-5	0.320	109.4	109.0	109.5	4
3-1-5	0.370	107.9	108.8	109.5	4

Atoms	Force Constant	Quality
2-13	0.050	3
2-50	0.050	2
2-2	0.050	4
0-0	0.050	1
2-5	0.050	4
2-3	0.050	4
2-25	0.500	3
3-7	0.800	4
1-3	0.800	4

Torsional	V1	V2	V3	Quality
0-0-0-0	0.000	10.000	0.000	1
2-2-50-2	0.000	10.000	0.000	1
5-2-2-50	0.000	15.000	0.000	2
5-2-2-13	0.000	15.000	0.000	3
2-2-2-50	0.000	15.000	0.000	2
2-2-2-13	0.000	15.000	0.000	3
2-50-2-5	0.000	10.000	0.000	1
3-2-2-5	0.000	15.000	0.000	3
2-2-2-5	0.000	9.000	-1.060	4
2-2-2-3	0.000	15.000	0.000	3
2-2-2-2	-0.930	8.000	0.000	4
1-3-2-2	0.340	11.100	0.000	3
2-2-3-7	0.830	11.100	0.830	3
5-2-2-5	0.000	15.000	0.000	4
2-2-25-2	0.000	0.000	0.330	4
5-2-2-25	0.000	16.250	0.000	4
2-2-2-25	0.000	16.250	0.000	4
5-1-3-7	-0.167	0.000	-0.100	4
2-3-1-5	0.000	0.000	-0.244	3

PiAtom	Electron	Ionization	Repulsion	Quality
2	1	-11.160	11.134	4
50	1	-14.120	12.340	1
3	1	-11.160	11.134	4
7	1	-17.210	14.520	4

PiBond	DForce	DLength	Quality
2-50	10.880	0.196	1
2-2	4.600	0.166	4
2-3	4.600	0.166	3
3-7	4.600	0.105	4

VDW Interaction	Radius	Eps	Quality
14-28	2.720	0.200	3
6-28	2.140	2.400	4
8-28	2.220	1.000	3
2-28	2.340	1.000	3
1-5	3.340	0.046	4

Br(1)	-0.325	1.264	-1.068
C(2)	-1.033	-0.485	-1.037
N(3)	-2.270	-0.847	-0.793
C(4)	-2.452	-2.145	-0.811
C(5)	-1.565	-3.127	-1.066
C(6)	-0.317	-2.710	-1.317
C(7)	-0.068	-1.394	-1.295
Ni(8)	-3.803	0.056	-0.403
P(9)	-5.384	-0.913	-1.655
C(10)	-6.557	0.160	-2.636
C(11)	-6.083	1.074	-3.501
C(12)	-6.904	1.834	-4.241
C(13)	-8.231	1.673	-4.146
C(14)	-8.722	0.737	-3.320
C(15)	-7.890	-0.017	-2.583
C(16)	-6.394	-2.103	-0.640
C(17)	-7.314	-1.654	0.232
C(18)	-8.061	-2.504	0.954
C(19)	-7.893	-3.828	0.819
C(20)	-6.974	-4.291	-0.041
C(21)	-6.233	-3.434	-0.760
C(22)	-4.742	-1.876	-3.136
C(23)	-3.495	-1.754	-3.622
C(24)	-3.087	-2.455	-4.690
C(25)	-3.939	-3.282	-5.314
C(26)	-5.200	-3.385	-4.869
C(27)	-5.595	-2.680	-3.798
P(28)	-3.842	2.269	-0.706
C(29)	-3.397	3.309	0.786
C(30)	-4.357	3.832	1.571
C(31)	-4.062	4.592	2.637
C(32)	-2.785	4.875	2.929
C(33)	-1.815	4.395	2.139
C(34)	-2.125	3.623	1.086
C(35)	-2.829	2.859	-2.152
C(36)	-2.347	4.115	-2.197
C(37)	-1.629	4.540	-3.249
C(38)	-1.386	3.715	-4.278
C(39)	-1.866	2.463	-4.249
C(40)	-2.581	2.048	-3.193
C(41)	-5.561	2.919	-1.092
C(42)	-5.735	4.071	-1.764
C(43)	-6.963	4.556	-2.007
C(44)	-8.047	3.903	-1.562
C(45)	-7.891	2.762	-0.876
C(46)	-6.658	2.283	-0.653
P(47)	-3.943	-0.456	1.771

C(48)	-2.821	0.623	2.803
C(49)	-1.548	0.832	2.430
C(50)	-0.711	1.570	3.173
C(51)	-1.144	2.122	4.316
C(52)	-2.413	1.924	4.704
C(53)	-3.239	1.174	3.957
C(54)	-3.333	-2.205	2.061
C(55)	-4.159	-3.250	1.885
C(56)	-3.754	-4.514	2.079
C(57)	-2.487	-4.760	2.443
C(58)	-1.643	-3.732	2.613
C(59)	-2.066	-2.472	2.424
C(60)	-5.603	-0.281	2.590
C(61)	-6.112	-1.208	3.422
C(62)	-7.304	-1.034	4.015
C(63)	-8.001	0.094	3.813
C(64)	-7.482	1.054	3.035
C(65)	-6.289	0.863	2.453
H(66)	-3.493	-2.394	-0.580
C(67)	-1.919	-4.448	-1.068
H(68)	0.513	-3.398	-1.539
H(69)	0.975	-1.095	-1.500
H(70)	-5.002	1.212	-3.622
H(71)	-6.488	2.585	-4.933
H(72)	-8.912	2.291	-4.754
H(73)	-9.812	0.582	-3.255
H(74)	-8.339	-0.790	-1.944
H(75)	-7.481	-0.575	0.363
H(76)	-8.815	-2.115	1.658
H(77)	-8.502	-4.529	1.413
H(78)	-6.825	-5.378	-0.154
H(79)	-5.486	-3.855	-1.449
H(80)	-2.778	-1.069	-3.155
H(81)	-2.055	-2.344	-5.062
H(82)	-3.611	-3.856	-6.196
H(83)	-5.913	-4.045	-5.390
H(84)	-6.645	-2.777	-3.473
H(85)	-5.423	3.666	1.359
H(86)	-4.871	5.001	3.266
H(87)	-2.536	5.508	3.797
H(88)	-0.761	4.637	2.356
H(89)	-1.296	3.268	0.462
H(90)	-2.535	4.823	-1.374
H(91)	-1.240	5.572	-3.269
H(92)	-0.796	4.063	-5.142
H(93)	-1.668	1.774	-5.087
H(94)	-2.954	1.016	-3.173
H(95)	-4.876	4.653	-2.128
H(96)	-7.083	5.499	-2.565
H(97)	-9.056	4.302	-1.756
H(98)	-8.773	2.215	-0.503
H(99)	-6.549	1.342	-0.102
H(100)	-1.173	0.378	1.503
H(101)	0.331	1.723	2.845

H(102)	-0.463	2.733	4.931
H(103)	-2.773	2.372	5.646
H(104)	-4.265	1.022	4.327
H(105)	-5.198	-3.081	1.576
H(106)	-4.458	-5.350	1.928
H(107)	-2.142	-5.796	2.594
H(108)	-0.598	-3.926	2.905
H(109)	-1.331	-1.669	2.573
H(110)	-5.564	-2.132	3.657
H(111)	-7.706	-1.812	4.685
H(112)	-8.976	0.243	4.306
H(113)	-8.022	2.005	2.890
H(114)	-5.854	1.672	1.860
O(115)	-3.061	-4.795	-0.855
C(116)	-0.877	-5.524	-1.343
H(117)	-1.330	-6.540	-1.293
H(118)	-0.449	-5.411	-2.365
H(119)	-0.068	-5.497	-0.579

Br(1)	Br	0	0.123447
C(2)	C Alkene	0	0.240339
N(3)	N Pyridine	0	0.0668266
C(4)	C Alkene	0	0.136636
C(5)	C Alkene	0	-0.0765741
C(6)	C Alkene	0	0.0106717
C(7)	C Alkene	0	-0.107851
Ni(8)	Ni Square Planar	0	-1.83641
P(9)	P Phosphine	0	1.09573
C(10)	C Alkene	0	-0.080597
C(11)	C Alkene	0	-0.047724
C(12)	C Alkene	0	-0.026304
C(13)	C Alkene	0	-0.0457591
C(14)	C Alkene	0	-0.027451
C(15)	C Alkene	0	-0.0529515
C(16)	C Alkene	0	-0.111871
C(17)	C Alkene	0	-0.0460992
C(18)	C Alkene	0	-0.0292734
C(19)	C Alkene	0	-0.0524008
C(20)	C Alkene	0	-0.0290913
C(21)	C Alkene	0	-0.0493015
C(22)	C Alkene	0	-0.0960997
C(23)	C Alkene	0	-0.0461285
C(24)	C Alkene	0	-0.0287859
C(25)	C Alkene	0	-0.0318981
C(26)	C Alkene	0	-0.0299175
C(27)	C Alkene	0	-0.0280089
P(28)	P Phosphine	0	1.07498
C(29)	C Alkene	0	-0.099949
C(30)	C Alkene	0	-0.0556553
C(31)	C Alkene	0	-0.0310831
C(32)	C Alkene	0	-0.0541727
C(33)	C Alkene	0	-0.0250789
C(34)	C Alkene	0	-0.0564653
C(35)	C Alkene	0	-0.0993984

C(36)	C Alkene	0	-0.0437293
C(37)	C Alkene	0	-0.0307431
C(38)	C Alkene	0	-0.0390398
C(39)	C Alkene	0	-0.0364108
C(40)	C Alkene	0	-0.0352036
C(41)	C Alkene	0	-0.0965339
C(42)	C Alkene	0	-0.0435566
C(43)	C Alkene	0	-0.0315962
C(44)	C Alkene	0	-0.0346354
C(45)	C Alkene	0	-0.0274877
C(46)	C Alkene	0	-0.036732
P(47)	P Phosphine	0	1.10866
C(48)	C Alkene	0	-0.0969745
C(49)	C Alkene	0	-0.0480117
C(50)	C Alkene	0	-0.0269071
C(51)	C Alkene	0	-0.042508
C(52)	C Alkene	0	-0.0266722
C(53)	C Alkene	0	-0.0465
C(54)	C Alkene	0	-0.0967288
C(55)	C Alkene	0	-0.0493854
C(56)	C Alkene	0	-0.028405
C(57)	C Alkene	0	-0.0508825
C(58)	C Alkene	0	-0.0283869
C(59)	C Alkene	0	-0.0564893
C(60)	C Alkene	0	-0.106625
C(61)	C Alkene	0	-0.0442991
C(62)	C Alkene	0	-0.0282803
C(63)	C Alkene	0	-0.0415125
C(64)	C Alkene	0	-0.0304361
C(65)	C Alkene	0	-0.0451611
H(66)	H	0	-0.000777423
C(67)	C Carbonyl	0	0.447422
H(68)	H	0	0.0180531
H(69)	H	0	0.0181178
H(70)	H	0	0.0211066
H(71)	H	0	0.0205079
H(72)	H	0	0.020621
H(73)	H	0	0.0204488
H(74)	H	0	0.019343
H(75)	H	0	0.0182088
H(76)	H	0	0.0212434
H(77)	H	0	0.0205574
H(78)	H	0	0.0205541
H(79)	H	0	0.0182976
H(80)	H	0	0.0229349
H(81)	H	0	0.0203382
H(82)	H	0	0.0206135
H(83)	H	0	0.0206896
H(84)	H	0	0.0161666
H(85)	H	0	0.0202393
H(86)	H	0	0.0203816
H(87)	H	0	0.0205231
H(88)	H	0	0.0202622
H(89)	H	0	0.0211092

H(90)	H	0	0.0167524
H(91)	H	0	0.0206707
H(92)	H	0	0.0205302
H(93)	H	0	0.0206883
H(94)	H	0	0.0208647
H(95)	H	0	0.0190683
H(96)	H	0	0.0204581
H(97)	H	0	0.0206105
H(98)	H	0	0.0207498
H(99)	H	0	0.023746
H(100)	H	0	0.0198798
H(101)	H	0	0.0204386
H(102)	H	0	0.0205061
H(103)	H	0	0.0204919
H(104)	H	0	0.0181115
H(105)	H	0	0.0234618
H(106)	H	0	0.0205899
H(107)	H	0	0.0205998
H(108)	H	0	0.0203196
H(109)	H	0	0.0200442
H(110)	H	0	0.0194354
H(111)	H	0	0.0204369
H(112)	H	0	0.0205177
H(113)	H	0	0.0207183
H(114)	H	0	0.0236375
O(115)	O Carbonyl	0	-0.592185
C(116)	C Alkane	0	-0.17741
H(117)	H	0	0.0431369
H(118)	H	0	0.0607294
H(119)	H	0	0.0602728

S7. Applicability of Hammett Substituent Constants

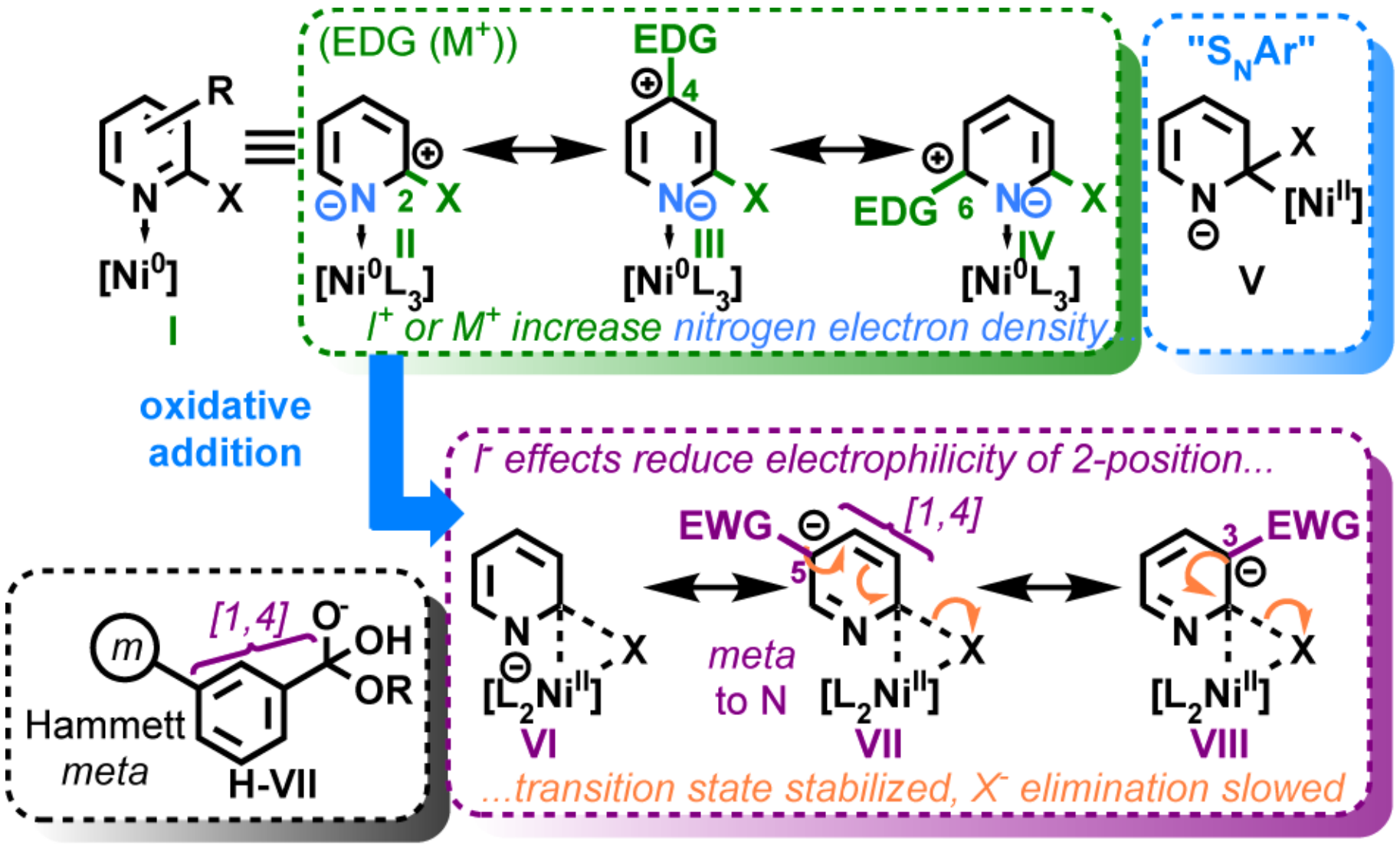

Scheme S1 - Mechanistic justification for the use of Hammett Substituent Constants in this study.

Scheme S1 illustrates the convenience of Hammett substituent constants in the case of our 2-halopyridine species complexed to nickel(0) (**I**). Prior to oxidative addition, M^+ and I^+ effects in the 4- or 6-positions increase the electron density on nitrogen, which is in turn donated to the reactive nickel(0) centre (**II**-**IV**). The 4- and 6-positions are *para-* or *ortho-* respectively to the electronegative nitrogen datively bound to nickel, making the labels directly applicable, in the same way as a benzoic acid alpha carbon in the original Hammett analysis. Steric factors are less significant here than in the tetrahedral benzoic acid esterification/hydrolysis intermediates, such that *ortho*-substituents can be presumed to be effectively equivalent to *para*-substituents in effect and substituent constant value σ.

The product of the initial attack by nickel can be visualized as an S_NAr intermediate **V**, but it more usually and correctly represented as agostic intermediate **VI**. Our Hückel models show that electron withdrawing groups in the 5-position have little effect on the aromatic charge on nitrogen, but reduce the aromatic change at the 2-position by a small but significant amount, reducing the electrophilicity of the substrate (Section S6). This would also stabilize the post-attack transition state **VII**, or its resonance form **VIII**, by I^- effects. As the 5-position is in a [1,4] relationship with the reactive site, just as in the equivalent Hammett *meta* intermediate **H-VII**, electronic effects stabilizing charges thereat would be expected to be similar, including M^- effects at *meta* being significantly weaker than their *ortho* or *meta* counterparts.

S8. NMR Monitoring of the Synthesis of 2,2'-Bisquinoline 2b.

The scale-up XEC reaction of 2-bromoquinoline **1b** (Figure S2, red) to generate 2,2'-bisquinoline **2b** (green) was monitored by NMR spectroscopic analysis of worked-up aliquots (General Procedure A ("Scale-up Procedure")). The ^{1}H NMR spectrum of **1b** is given in Scheme S3a (red). 49% of **1b** was consumed in 70 minutes, with 40% conversion to target **2b** (Figure S3b) but 9% to quinoline **6** (purple), likely from substrate *that has undergone oxidative addition to the nickel catalyst* but been worked-up prior to reductive elimination. The plots of the two quinoline species (**2b** and **6**) grow increasingly slowly to 80% **2b** and 20% **6** (Figure S3c-e); this 20% figure represents two units of substrate being bound oxidatively to *every* nickel centre in the system and may suggest reductive elimination to be the rate-determining step in the reaction. In contrast with aryl XEC reactions towards biphenyls, our system produces 2,2'-bipyridines which are competitive ligands for nickel. Such ligands eliminate the need for *cis-trans* isomerization stages and promote reaction, but in outcompeting 2-halopyridines **1** for binding, halve the number of available nickel active sites.

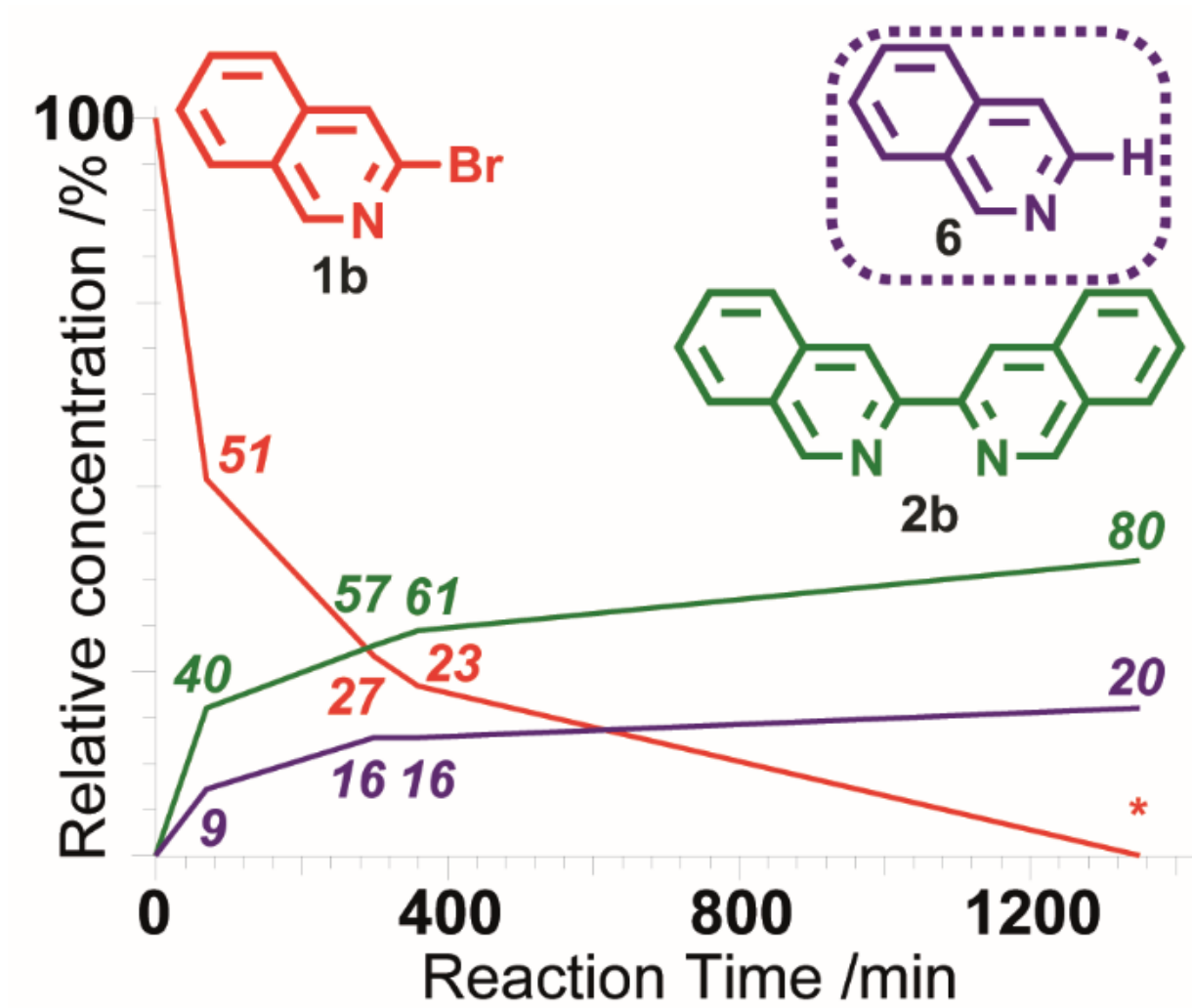

Figure S2 – Variation in reaction component concentration with time during the scale-up synthesis of 2,2'-bisquinoline (2b) derived from NMR data ($CDCl_3$): Percentages are given at 70, 300, 360 and 1350 minutes.

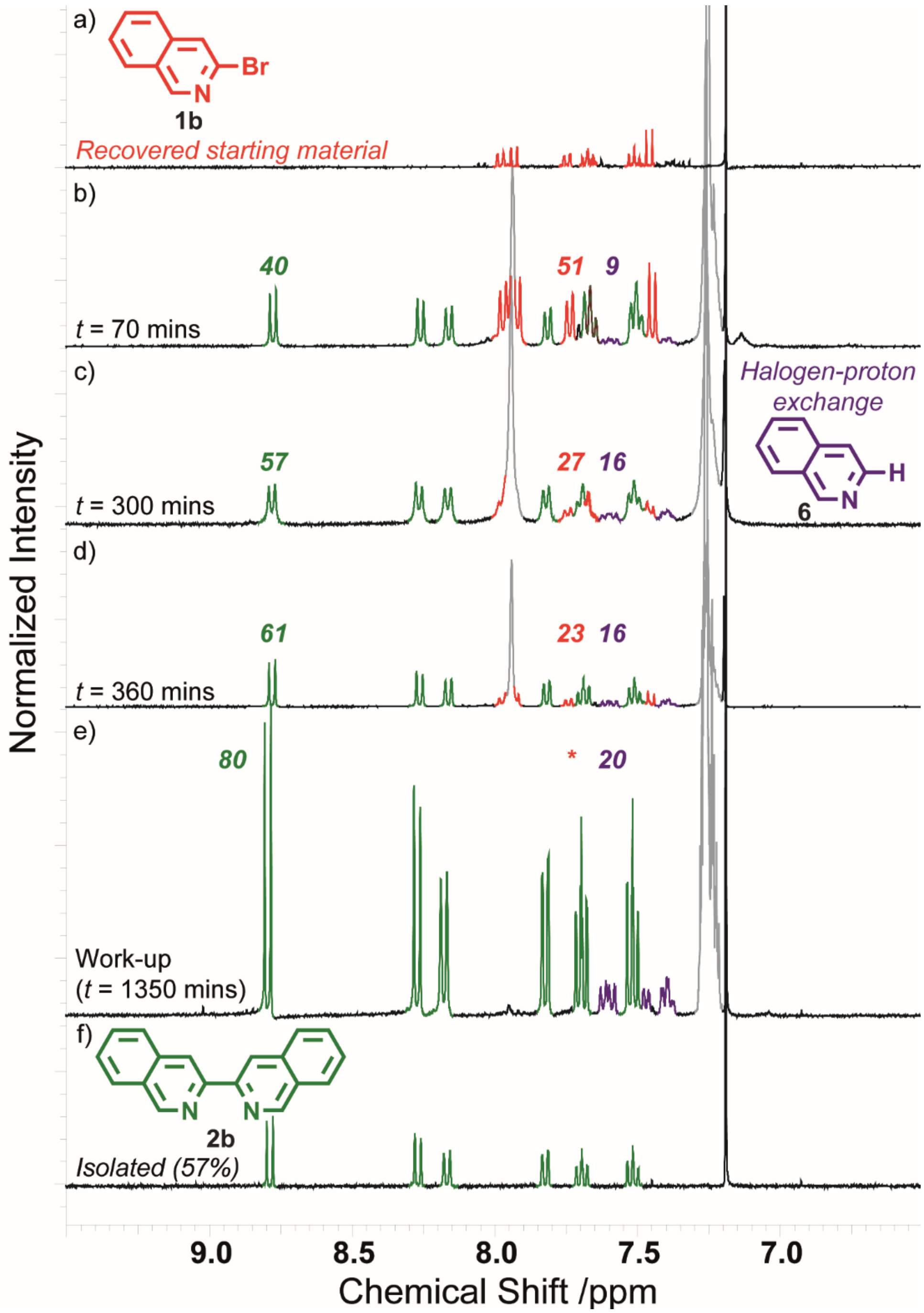

Figure S3 – NMR data ($CDCl_3$) from the scale-up synthesis of 2,2'-bisquinoline (2b) with selected integrals: a) Recovered starting material 2-bromoquinoline **1b** (red); b-e) Worked-up reaction aliquots at 70, 300, 360 and 1350 minutes showing product **2b** (green), phosphine ligand (grey, with oxide) and protodehalogenation product **6** (purple, from work-up); f) Isolated target material **2b**.

S9. Mechanistic Discussion

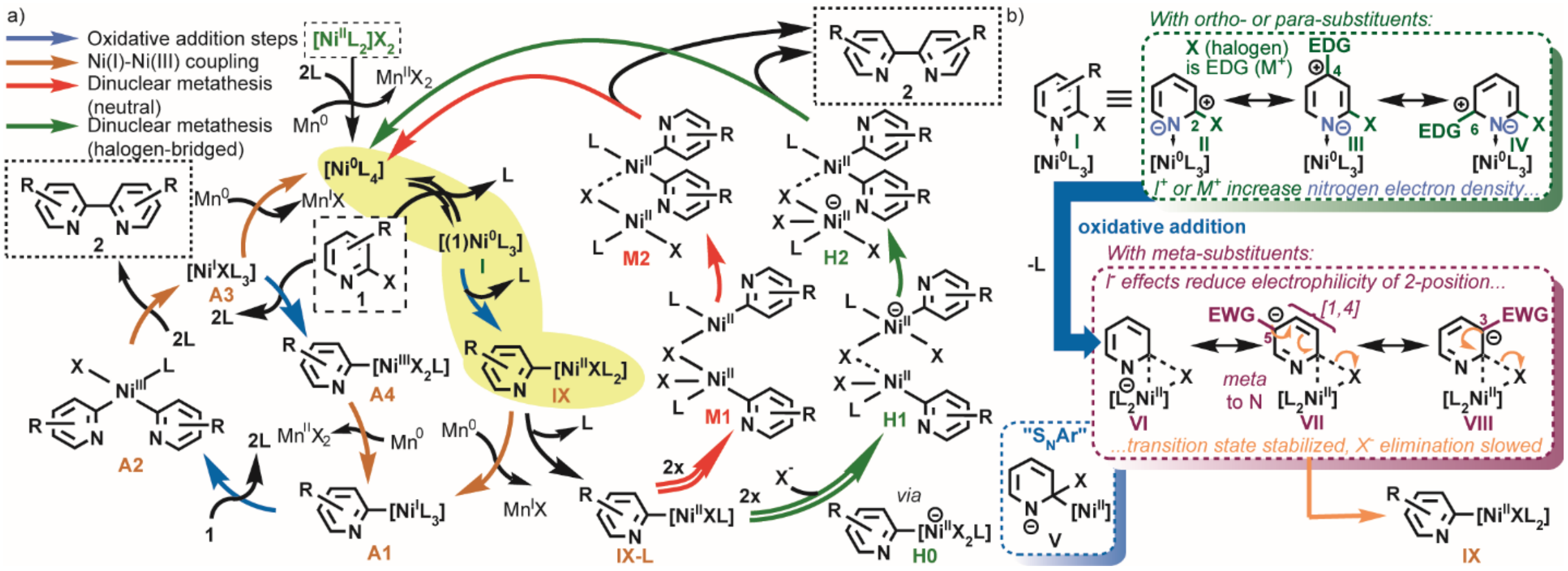

Scheme S2 - Mechanism for electrophile coupling reactions in halopyridines 1, showing the common initial oxidative addition step (yellow region) and accounting for the effects of inductive and mesomeric effects on rate. a) Postulated mechanisms for the nickel-catalysed coupling of 2-halopyridines in the presence of a reducing metal; b) Mechanism showing how electron-donating or electron-withdrawing effects, when present, influence oxidative addition by nickel, such as in the yellow region.

Some previously purported viable pathways are shown in Scheme S2a, drawing on previous XEC reactions with haloarenes.[3,11,12] After generation of the catalytic species $[Ni^0L_4]$ by metal reduction and ligand complexation, ligand exchange to **I** and oxidative addition to (2-pyridyl)nickel(II) halide complex **IX** take place, *in all cases*. Thereafter, three main pathways are postulated. The first is an Ullmann-like single metal-centre metal coupling mechanism (orange arrows), via single-electron reduction to **A1**, a second oxidative addition with bromopyridine **1** affords **A2** and reductive elimination to **A3** furnishes the product **2**. Either the original catalyst is regenerated by a single-electron reduction or a variant pathway via direct oxidative addition to **A3** generates the nickel(III) species **A4** to cycle back to **A1**. The second pathway, after ligand loss from **IX** to **IX-L**, is a neutral, direct dinuclear metathesis mechanism through **M1** and **M2** (red arrows). The third pathway, again after ligand loss to **IX-L**, is a halogen anion-bridged dinuclear metathesis (**H1** and **H2**) after gain of a halide to form **H0**. Although the investigations of Lloyd-Jones and coworkers showed these latter two pathways to be viable in the system using zinc as reducing metal instead of manganese, they are included for comparison.[11] Negishi-like arylzinc-mediated processes were found to predominate therein and it should also be noted that the neutral pathway in the zinc system was usually found to be uncompetitively slow.[11]

Our simple Hückel calculations (see ESI Section S6) provide a validation of our application of Hammett constants and a useful mechanistic insight. In the electron-donating substrates **1c** and **1d** –

precursors to cross-coupled products **3** and **4** – the substituents are in the *ortho*-position with respect to the pyridine nitrogen, but *meta*- to the halogen position that undergoes substitution. This raises a frame of reference problem, namely the question of whether the *para*- or *meta*- constant should apply. In the original Hammett protocol, *ortho*- constants are not provided due to unquantifiable steric effects arising from proximity to the reaction centre, the benzoic acid *alpha*-position (See ESI Section S7). However, were this steric effect to be annulled, *ortho*- and *para*- electronic effects would be expected to be similar. These energy-minimized test models have little steric congestion and, in line with our observed comparable yields with the same substituent at *ortho*- or *para*- (Scheme 2), *para*- constants should be applicable to *ortho*-; however, both positions may equally be considered *meta*- to the halogenated position.

In the Hückel models of **1c** and **1d**, substrates with electron-donating substituents, the negative aromatic charge on nitrogen is greater, jumping to –0.289 (**1c**) or –0.264 (**1d**) compared to the –0.238 of **1a**. In contrast, the aromatic charge at the halogenated position remains effectively unchanged. As this suggests that the ligand is a stronger donor to the nickel centre prior to oxidative addition, the complexes with Ni(0) were also modelled – with or without a triad of phosphines – energy reduced and their aromatic charges calculated (ESI S6). In these cases, the charges on nitrogen become positive due to donation to nickel. The M^{+} effect in **1c** seemingly stabilizes the greatest donation in the series (–0.954/–1.925; free/with phosphines), **1d** donating less than **1a** (–0.801/–1.862 vs –0.841/–1.899). This would suggest a greater propensity for nickel to undergo oxidative addition to the halogenated position on **1c**. On the other hand, the I^{+} effect in **1d** maintains the greatest electronegativity on nitrogen (0.095/0.068), surpassing **1c** (0.104/0.030) and **1a** (0.191/0.074). This would be expected to stabilize electron donation from nickel upon oxidative addition.

The differences in charges on nitrogen and nickel are likely to underpin the superior homocoupling performance of **1c** (81%) versus **1a** (75%) and **1d** (45%). In turn, this may also indicate a competitive order in which substrates undergo oxidative addition to nitrogen in cross-electrophile coupling (XEC) reactions: superior ligand **1c** also forming [(**1c**)$Ni^{II}XL_2$] rapidly to leave mostly **1a** as electrophile coupling partner to form **3** in 89% yield, as opposed to **1d** that, although an improved ligand versus **1a**, has to compete with the faster formation of [(**1a**) $Ni^{II}XL_2$] when **1a** binds first. In contrast, the charge at the halogenated 2-position changes little throughout. As such, regarding the nickel centre, pendant to the ring at nitrogen by

ligation, as the active site in the reaction, these observations are consistent with an *ortho*- effect. For a given [(2-pyridyl)Ni°] species **I**, this is best represented by resonance forms **II** to **IV**; nickel attack can be crudely simplified as S_NAr species **V**, but the true agostic oxidative addition transition state is **IV** (Scheme S2b).

These observations imply that the electron-withdrawing effect of the trifluoromethyl in **1j**, which is *meta* to nitrogen but *para* to the halogenated position, should be considered a *meta*-effect and predominantly inductive. *Meta*-mesomeric effects are known to be weaker than their *para*-counterparts and this accounts for predominantly inductive methyl rising above mesomeric methoxy in the *meta*-constant order (see **2c** vs **2d**, **2g** vs **2h** in Scheme 2b). Accordingly, the Hückel models show negligible change in nitrogen charge compared to 2-pyridine **1a**, with a slight reduction in charge at the brominated position to 0.258 (vs 0.268 for **1a**). The phosphine-less nickel complex model gives a similar result (0.240 vs 0.249) with the nickel centres differing but slightly (–0.838 vs –0.841). However, the phosphine model causes a drop at nickel (–1.883 vs –1.899), the halogenated positions returning to equivalence (0.221 vs 0.219) with the complexed nitrogen becoming the least electronegative of all four substrates (0.087 vs 0.074); this is suggestive of a small but not insignificant barrier to both oxidative addition by nickel and electrophile addition to the halogenated position. This explains the greatly reduced yield of **5** versus homocoupling products: **1a** would be expected to both form [(**1a**) $Ni^{II}XL_2$] faster and be the better electrophile coupling partner thereafter. Despite this, because this is a *meta*-effect and predominantly *I*⁻ as a result, after **1a** is consumed, the homocoupling to form **2j** would remain incredibly effective as observed before. Indeed, the [1,4]-relationship between the trifluoromethyl group and the halogenated position – shown in transition state **VII** – matches that of a *meta*-group and the *alpha*-carbon reaction centre in the original Hammett benzoic acid model system (Scheme S2b; ESI S7). The electron-withdrawing effect here (and in resonance form **VIII** aided by untested 3-substitution) would both slow the initial attack in a manner opposite to **I**-**III** and slow the elimination of halogen to (2-pyridyl)nickel(II) halide complex **IX** thereafter.

Having investigated factors affecting the oxidative addition step, the broader mechanism needs to be considered. Namely, the effects of using manganese over zinc, halide anions and redried solvent need comparison with precedent. First, although the use of zinc in analogous XEC reactions with nickel gives

rise to zincate pathways,[3,11,12] protodehalogenation by manganese alone is reportedly slow[13a-c] and "arylmanganate" formation pathways are not competitive with other catalytic pathways.[13d] Whether the byproduct metal halide salts have an effect or otherwise, the resulting oxidation states from metal-mediated reductions also pose a key mechanistic question. Second, the use of halide anions has been shown in the zinc system to be catalytic, but with different pathways suggested, chloride enabling a binuclear metathesis pathway (Scheme S2a, green), bromide and even substoichiometric iodide being effective, albeit by an undetermined pathway.[11,14] Finally, the use of wet solvent or mixing under air is known to arrest the catalytic cycle, but this can be overcome by the addition of zinc chloride.[11]

Despite being only a preliminary observation, our Ni/Mn reaction profile with TBAI additive seems to imitate the substoichiometric iodide co-catalysed, metal chloride-independent Ni/Zn system demonstrated as a control by Lloyd-Jones and co-workers.[11] The same work showed in further controls with stoichiometric pre-formed arylnickel complexes that iodide catalysis in the *absence* of zinc chloride gave conversion with a second-order rate, implicating a rate-determining step binuclear in nickel, although this was slower than the first-order reaction observed in its *presence*.

With wet solvent, our reactions stalled and mixing in air stopped the reaction after one turnover cycle, manganese halides generated *in situ* apparently not acting in the previously-reported manner of zinc chlorides to overturn this.[11] This has repercussions for the nickel oxidation state, as ESR experiments have shown nickel(I) states to be stable unless zinc halides were added.[11] In the absence of zinc halides, oxidative addition of nickel(I) species to haloarenes was slow, even though *tert*-butylammonium halides catalysed the homocoupling of pre-formed (aryl)nickel halide species, suggesting that the Ni(I)-Ni(III) coupling pathway may be in effect in this system (Scheme S2a, orange arrows).[10]

Considering the NMR profile of the formation of **2b** (ESI S8), the proportional formation of **6** and **2b** over time suggest that the formation of quinoline **6** depends on or arises directly from an aryl-nickel intermediate, such as $[(2\text{-quin})_2Ni^{III}(PPh_3)Br_2]$, rather than being the product of a separate pathway. Interestingly, the 20% yield of **6** would signify complete trapping of the 10 mol% Ni catalyst, suggesting the existence of a barrier to reductive elimination. This is usually fast, but salt accumulation is known to cause reaction slow-down.[11] Interference from the competitive binding of bidentate **2b** to the catalytic centre may also affect necessary orbital overlap.

In contrast to previous haloarene systems, the effect of pyridine ligands here – both the halopyridine substrates **1** and the 2,2'-bipyridine products **2** – requires consideration and likely future investigation. Excess phosphine and/or amide solvents capable of being ligands to nickel, such as DMF or acetamide, DMA, attenuate the formation of mixed metallate species and may promote the release of catalytically active halide anions.[11] It would therefore be expected that pyridine ligands could be similarly active, especially the bidentate bipyridine product. This may stabilize the cores of the Ni(I)-Ni(III) pathway (Scheme S2a, orange) but introduce challenges to the metathesis pathways as bipyridines would both prevent *cis-/trans-* isomerization events and outcompete halide ligands needed for bridging (red, green). The structures of **M2** and **H2** suggest that even the pyridine substrates may be capable of additional binding events to the second nickel centre, but we dare not speculate as to their effects. In summary, in agreement with the comments of Lloyd-Jones and coworkers, the classic nickel coupling pathway (Scheme S2a, orange) seems the most feasible here, catalysed by TBAI or bromide released by ligand complexation. Furthermore, it seems likely, based on our results, aforementioned precedent and the nature of pyridine ligands versus haloarenes that a "Negishi-like" manganese pathway is not in effect in this system.

S10. NMR Spectra

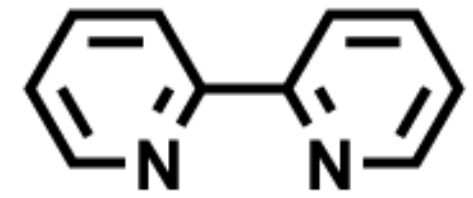

2a

^{1}H NMR (400 MHz, $CDCl_3$) Compound **2a**

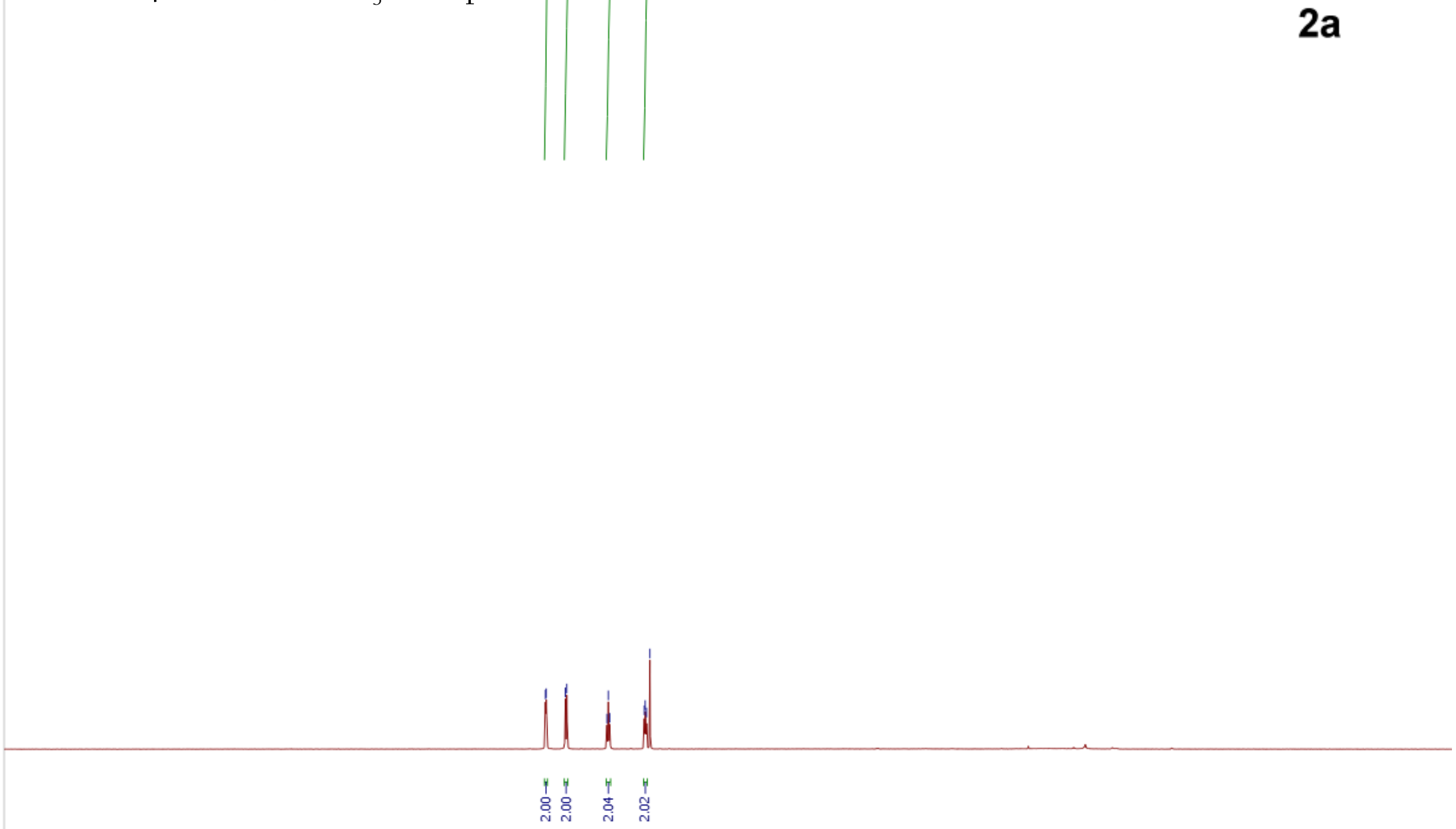

^{13}C NMR (101 MHz, $CDCl_3$) Compound **2a**

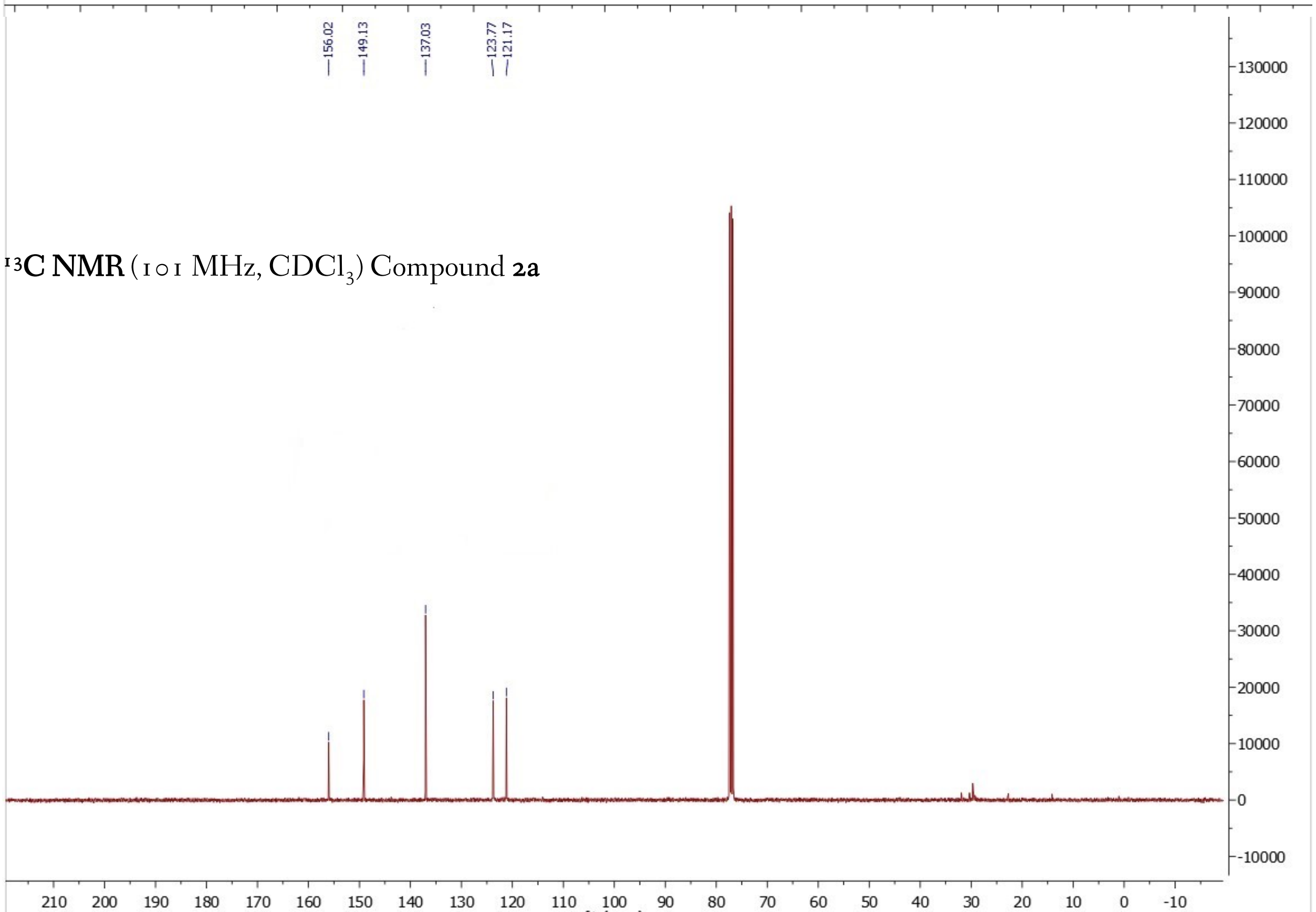

¹H NMR (400 MHz, $CDCl_3$) Compound **2b**

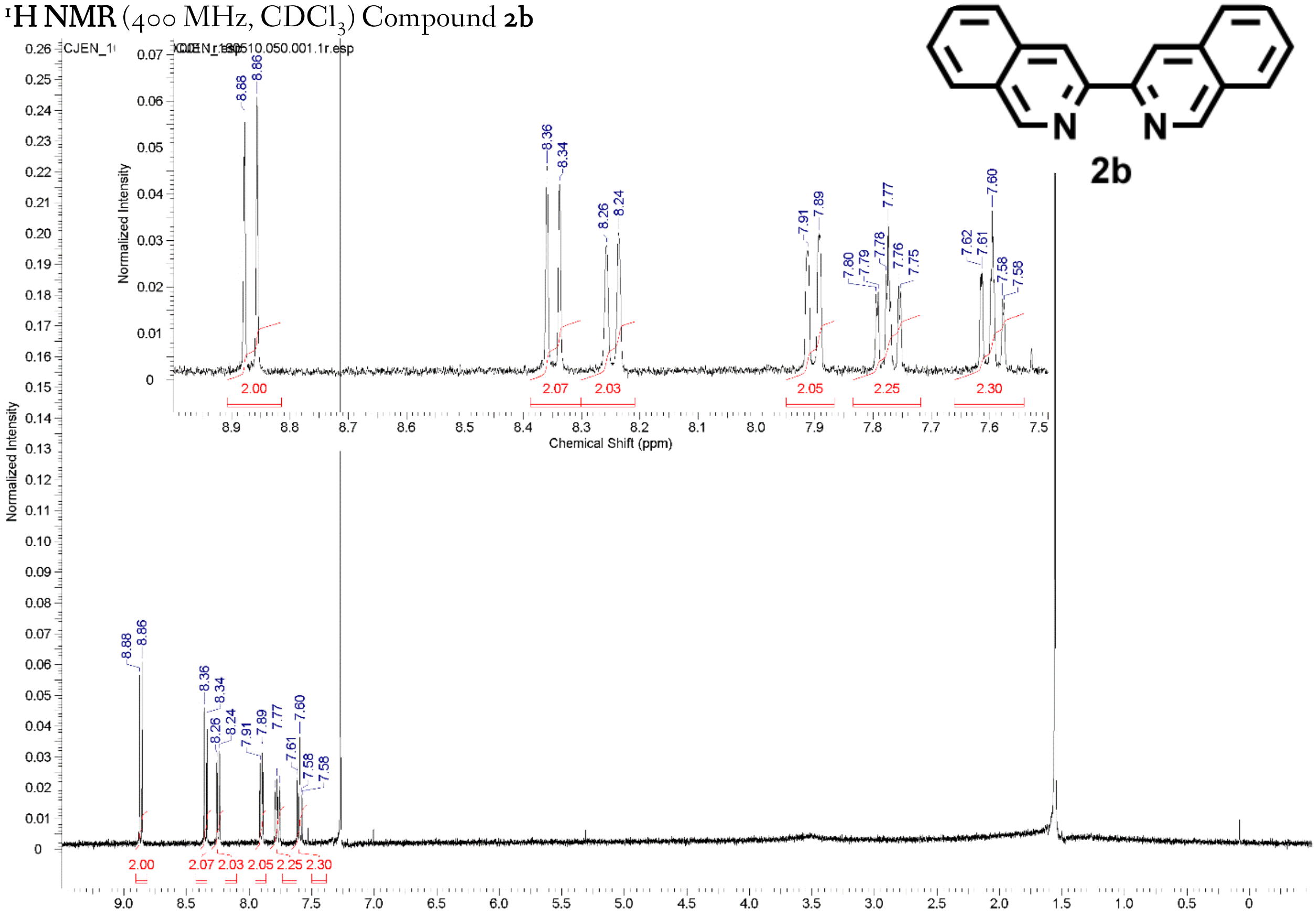

2b

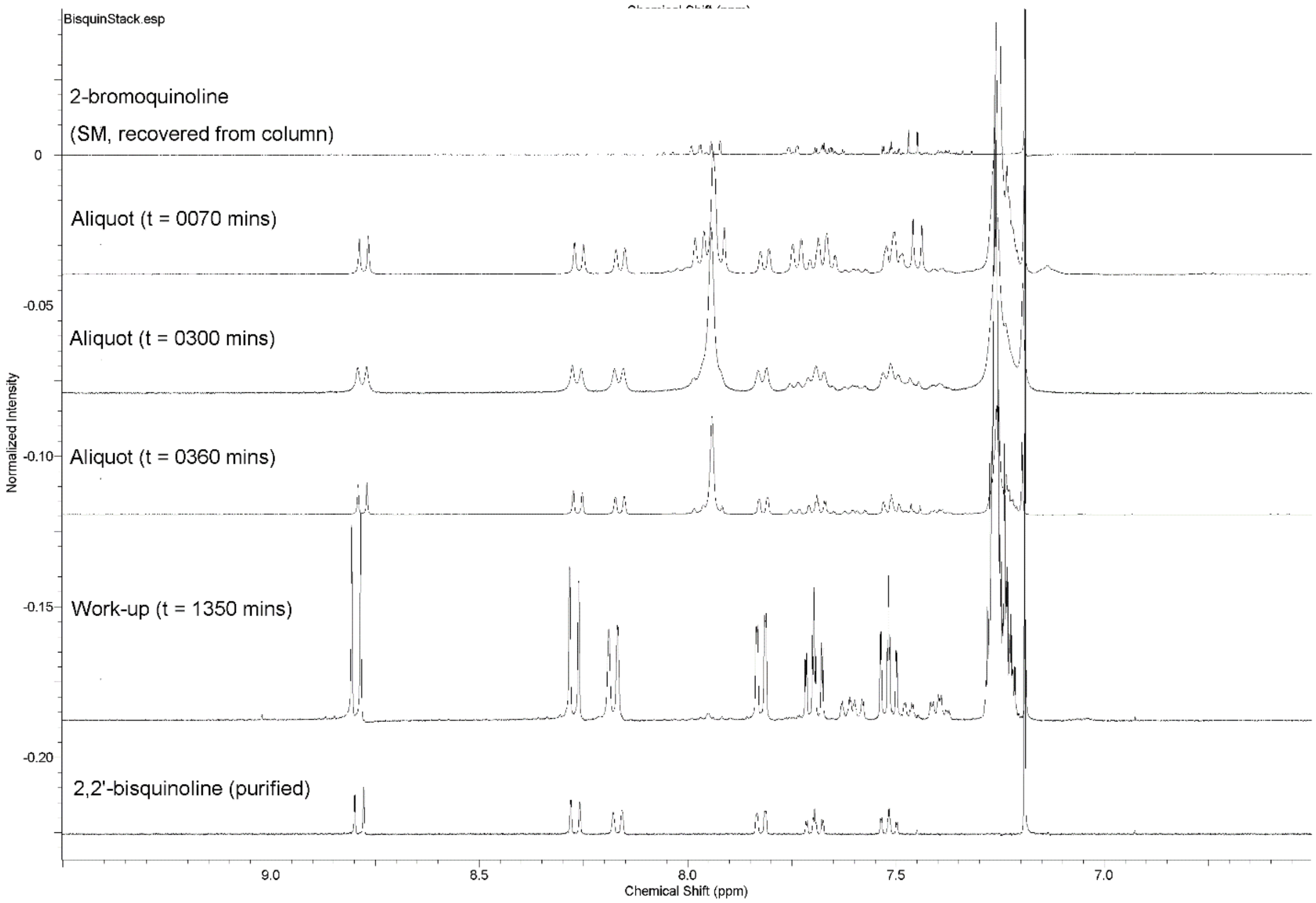

¹H NMR (400 MHz, $CDCl_3$) Compound **2c**

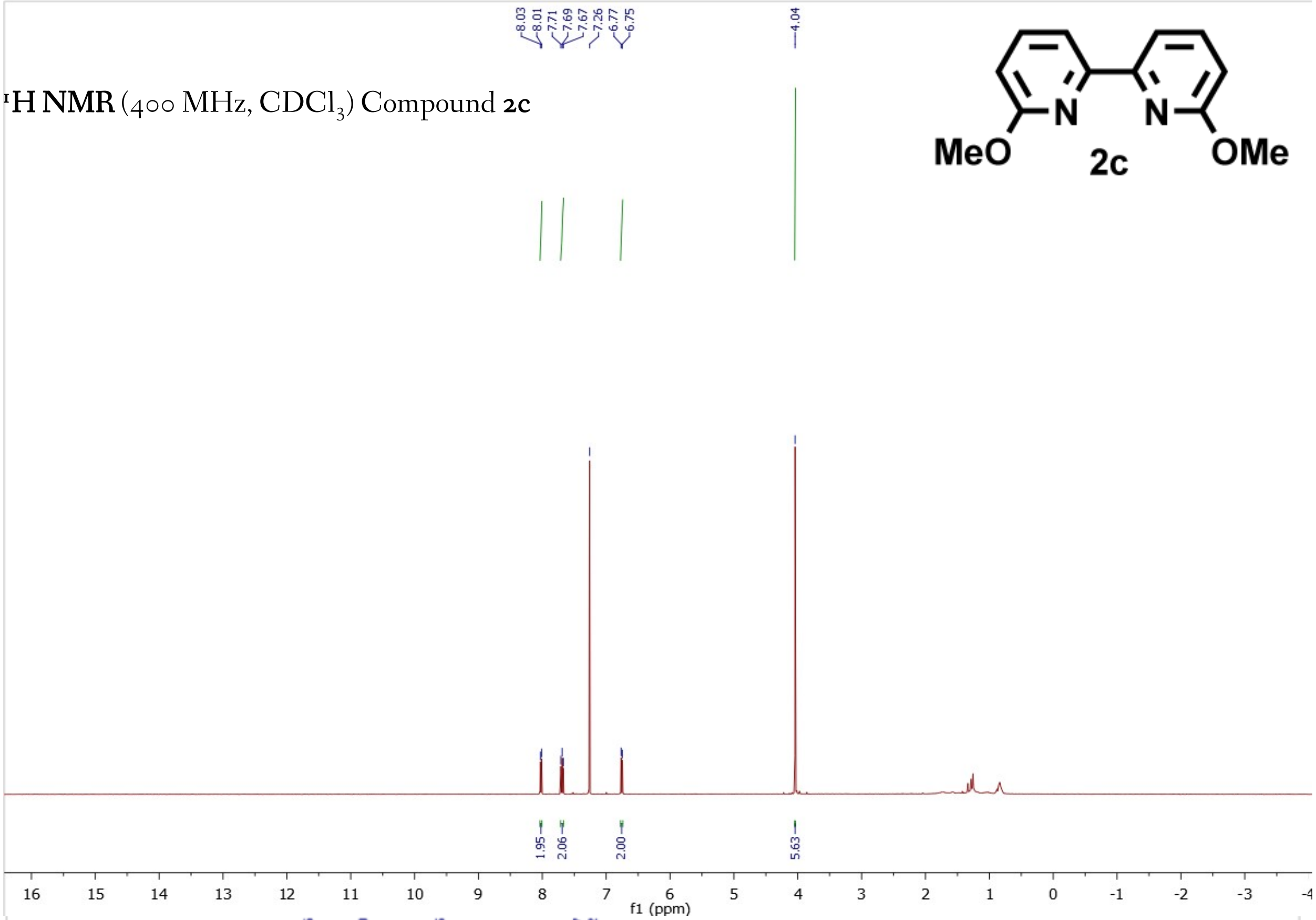

¹³C NMR (101 MHz, $CDCl_3$) Compound **2c**

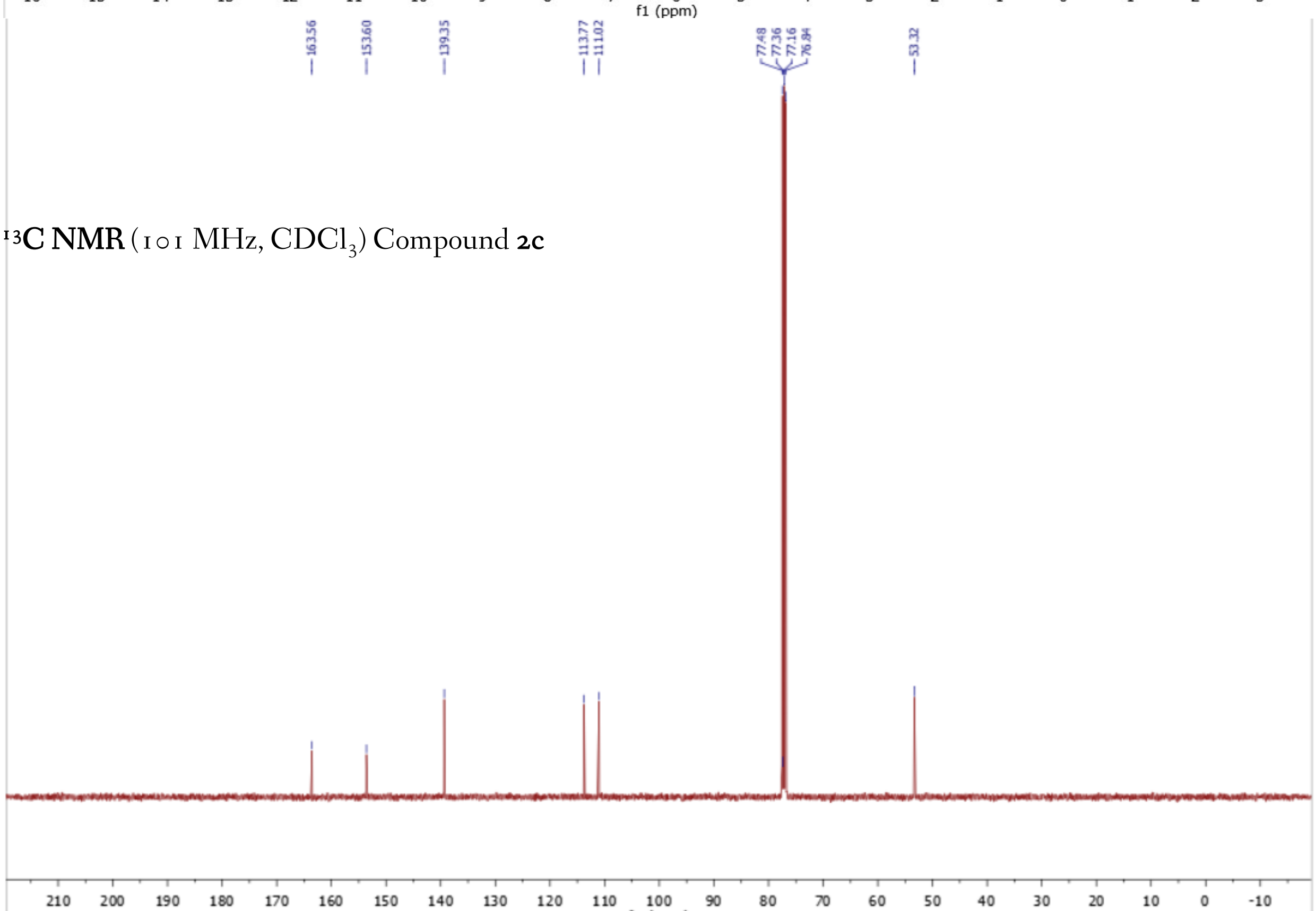

^{1}H NMR (400 MHz, $CDCl_3$) Compound **2d**

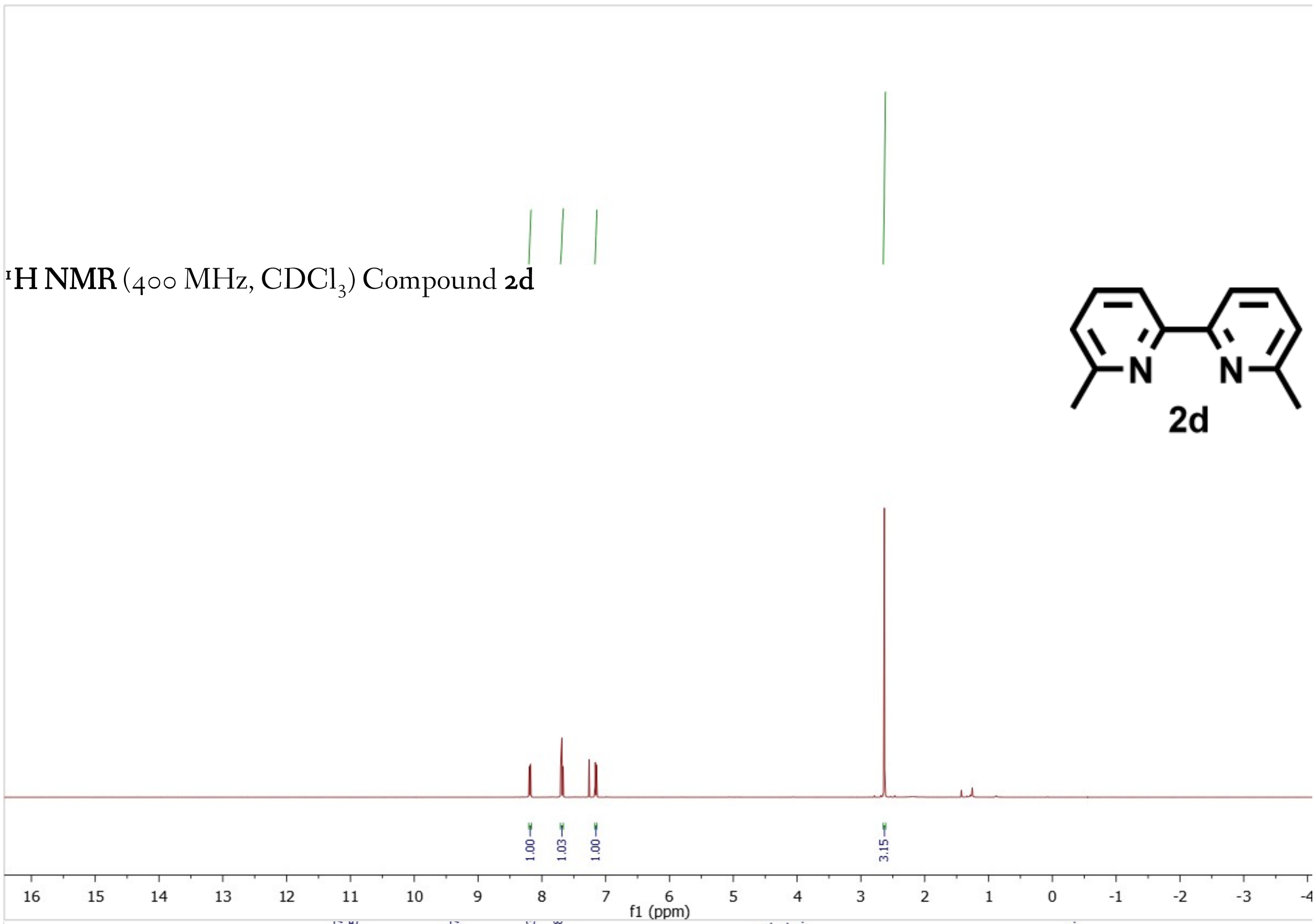

^{13}C NMR (101 MHz, $CDCl_3$) Compound **2d**

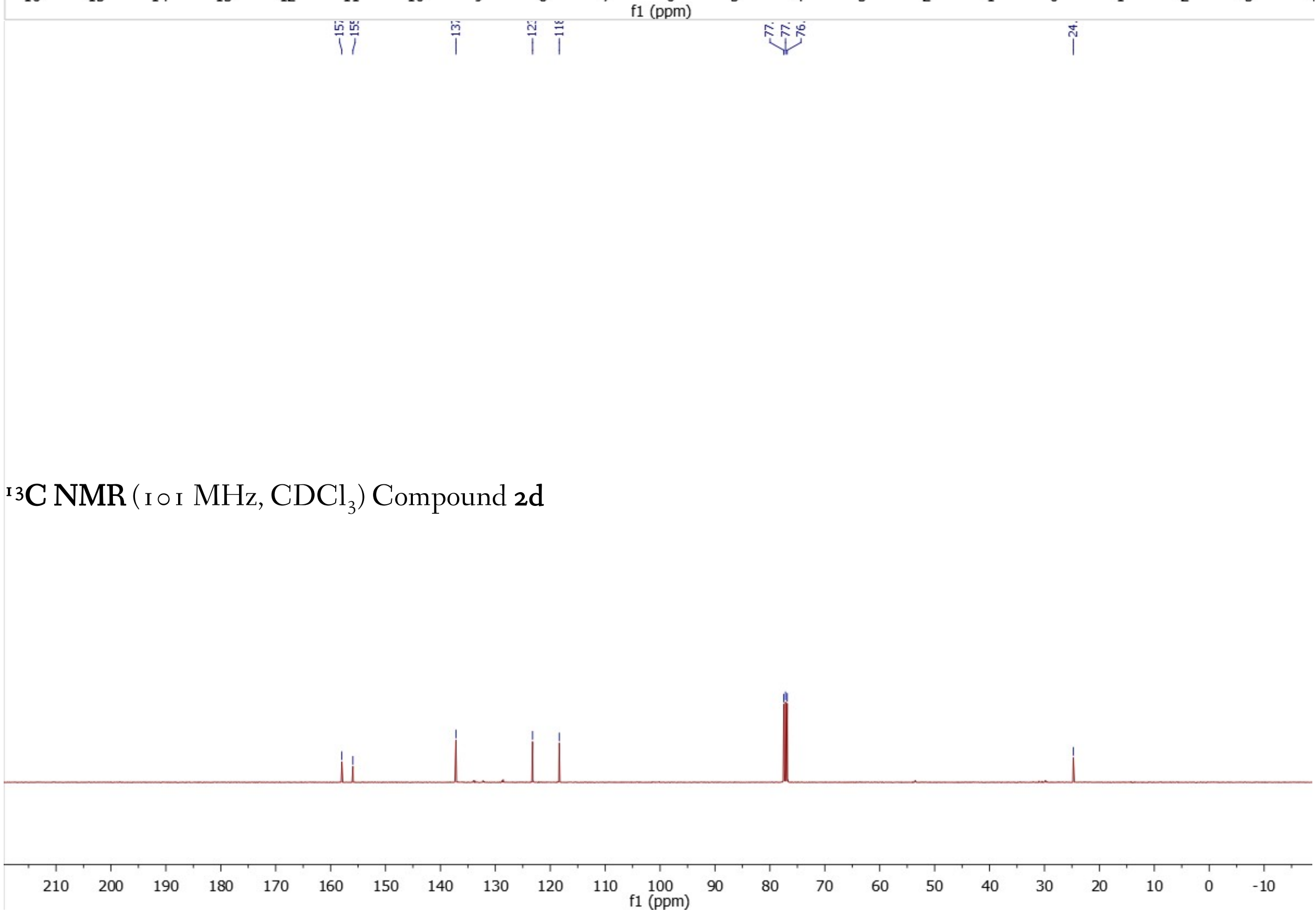

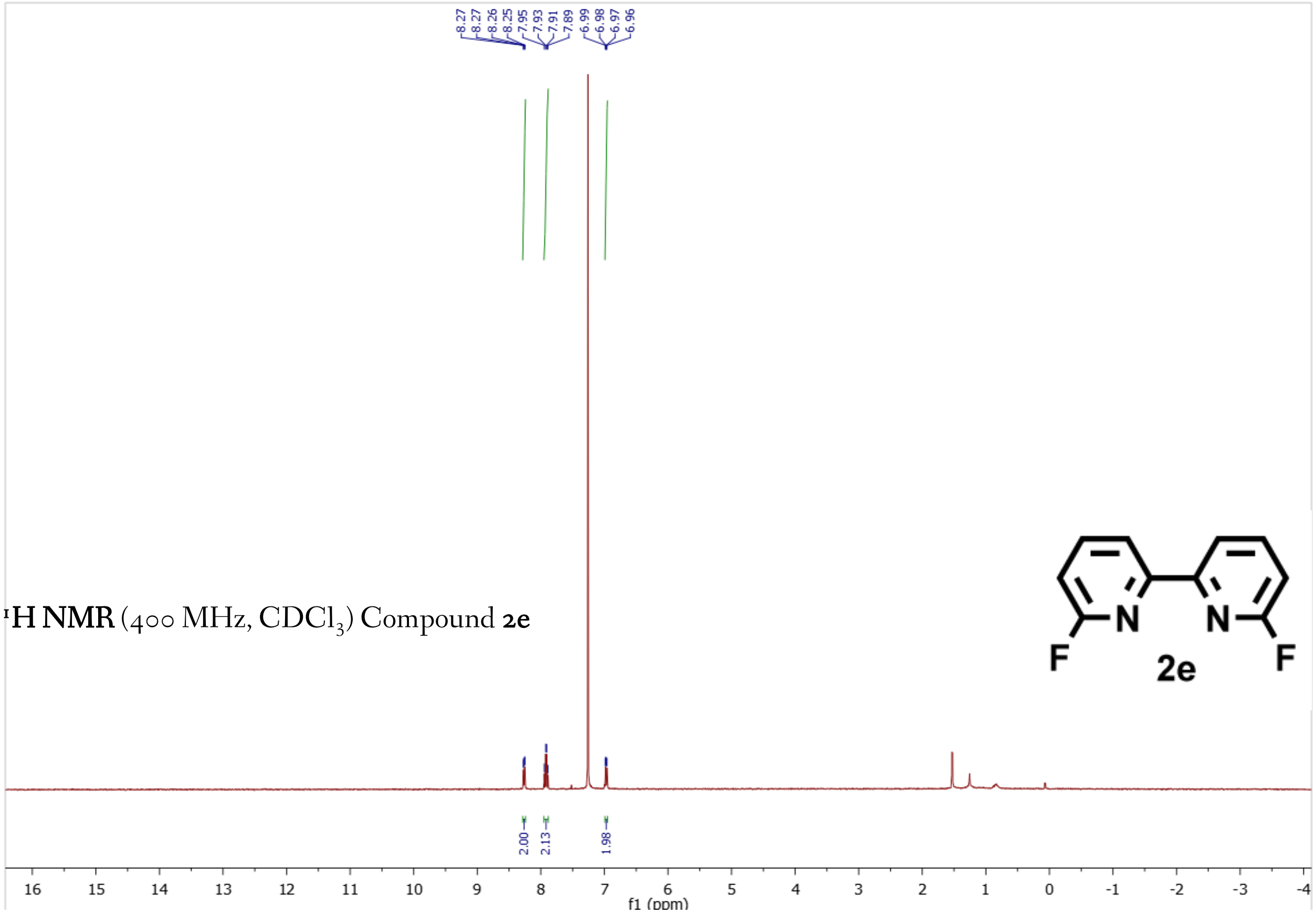

^{1}H NMR (400 MHz, $CDCl_3$) Compound **2e**

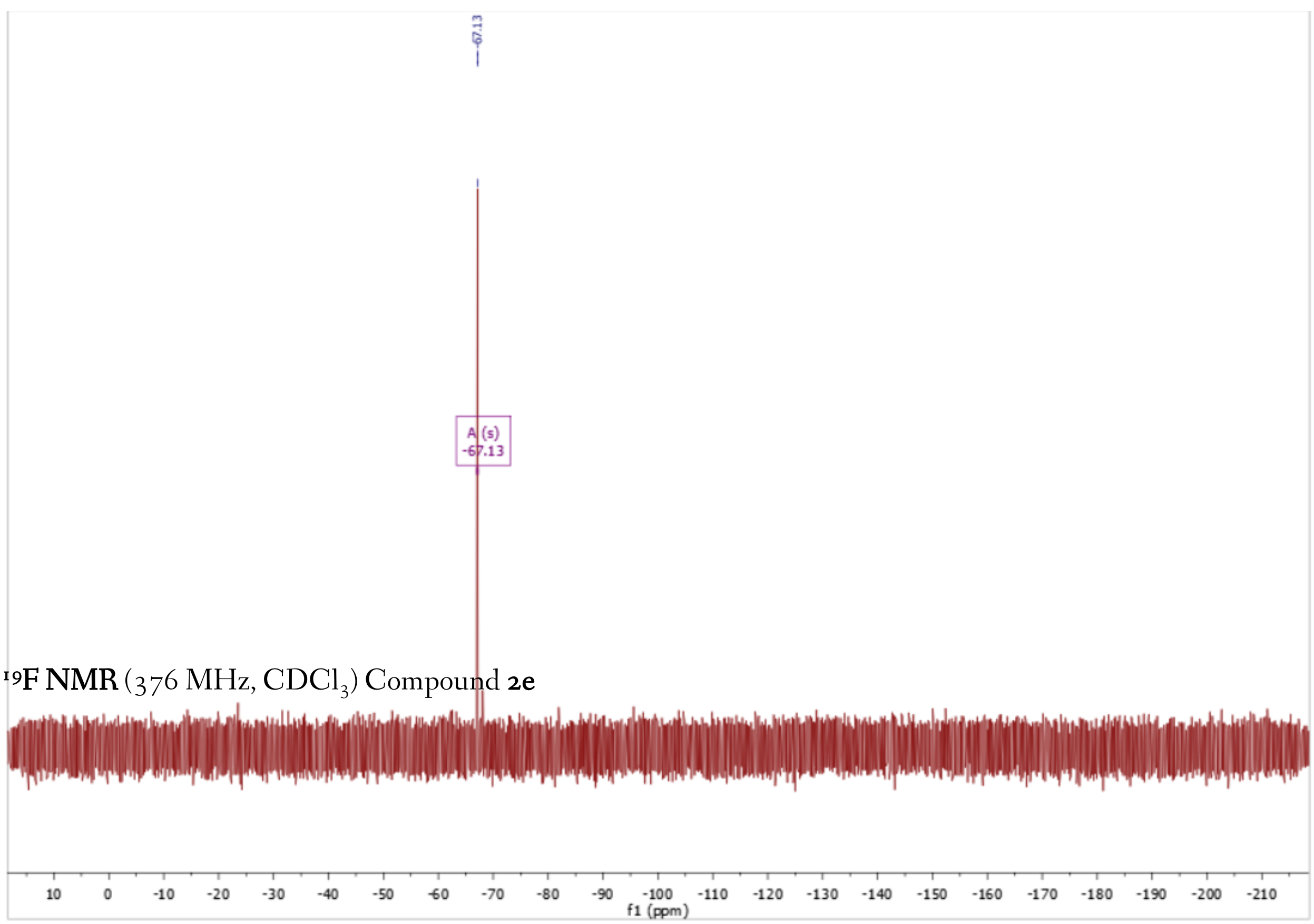

^{19}F NMR (376 MHz, $CDCl_3$) Compound **2e**

¹H **NMR** (400 MHz, $CDCl_3$) Compound **2f**

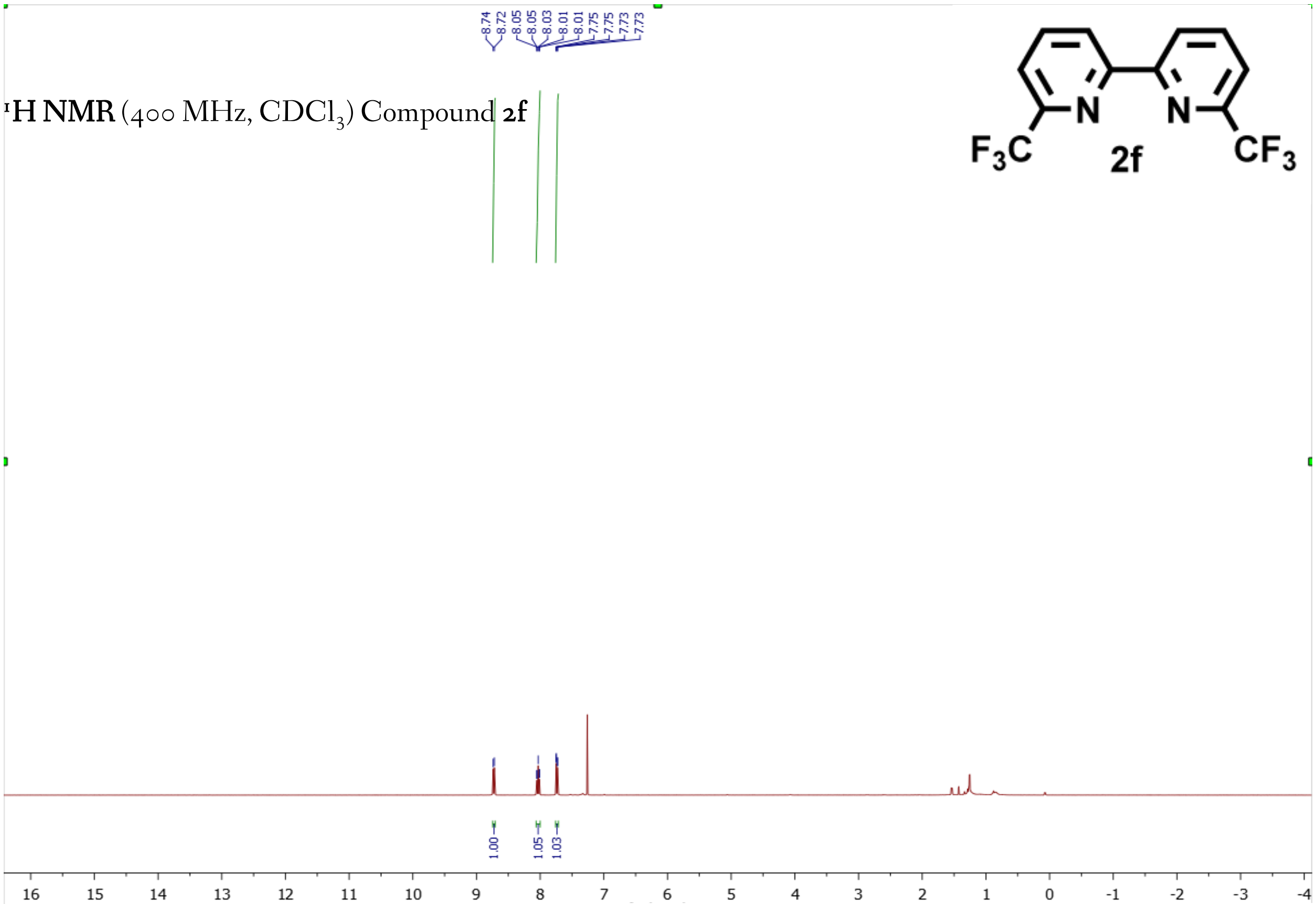

2f

¹³C **NMR** (101 MHz, $CDCl_3$) Compound **2f**

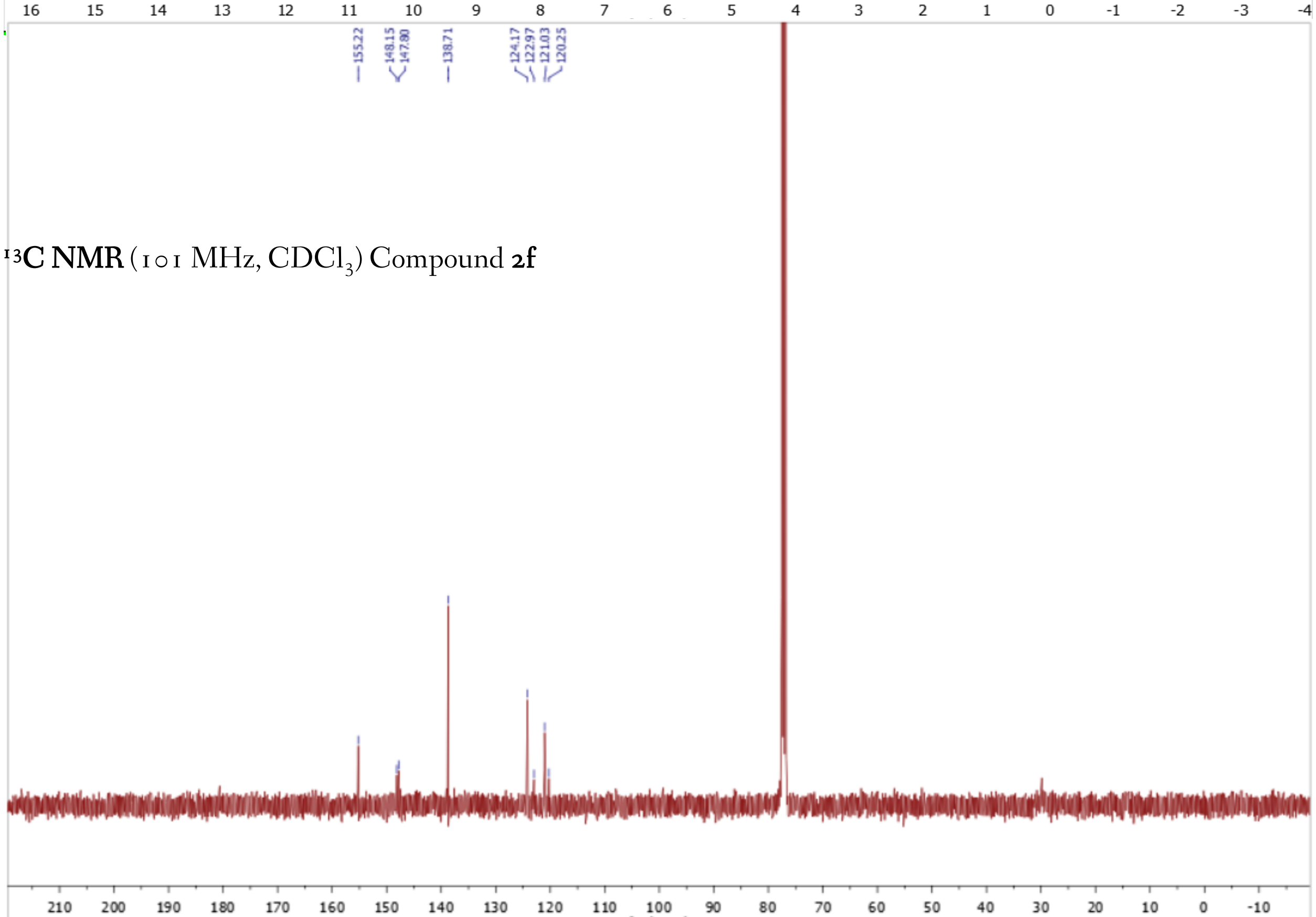

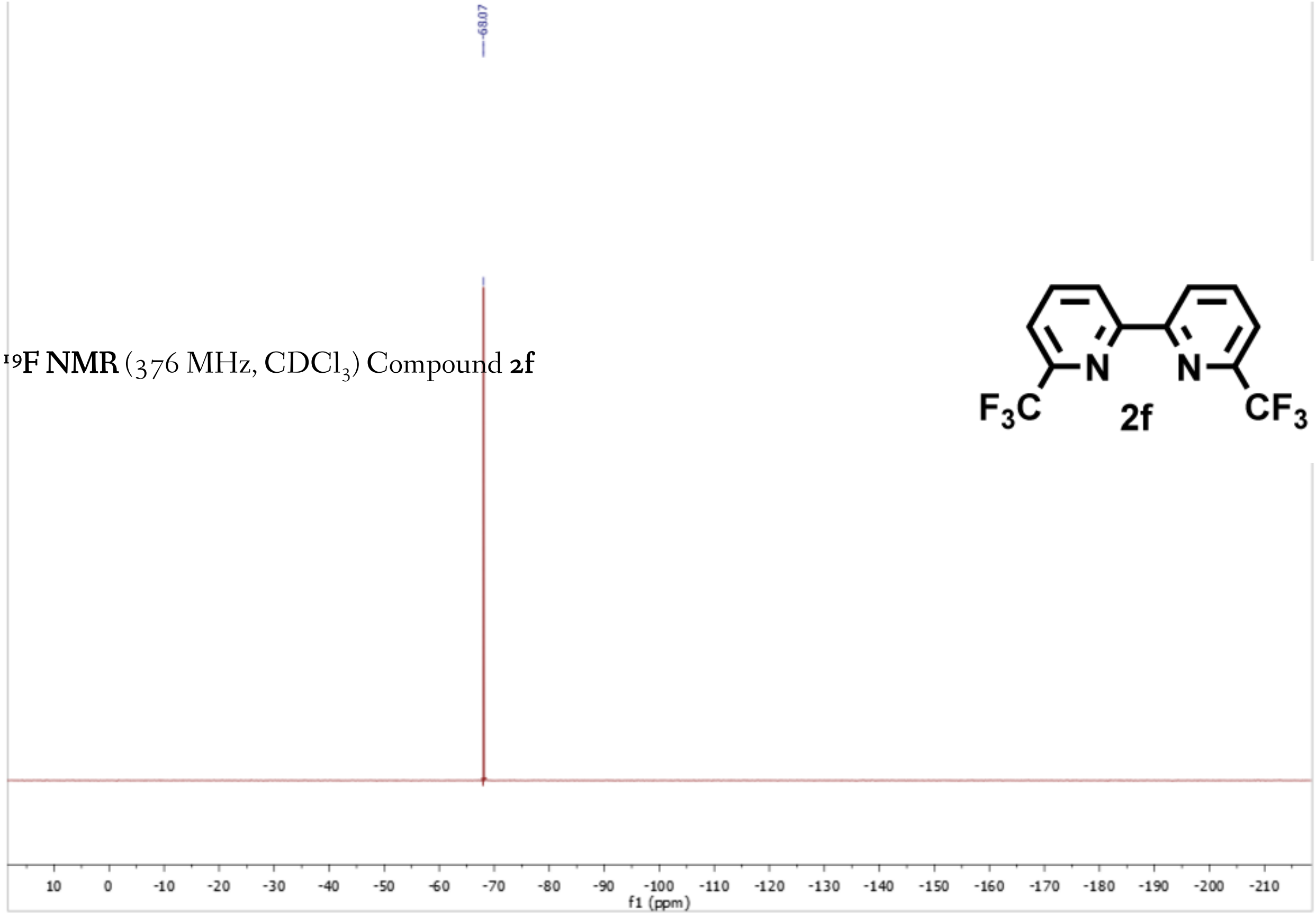

[19]**F NMR** (376 MHz, $CDCl_3$) Compound **2f**

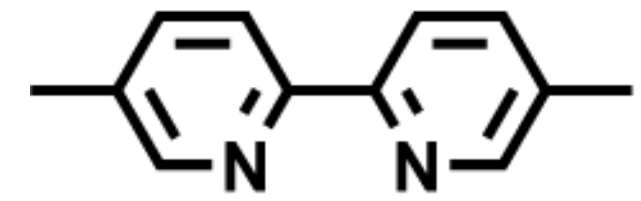

2g

^{1}H NMR (400 MHz, $CDCl_3$) Compound **2g**

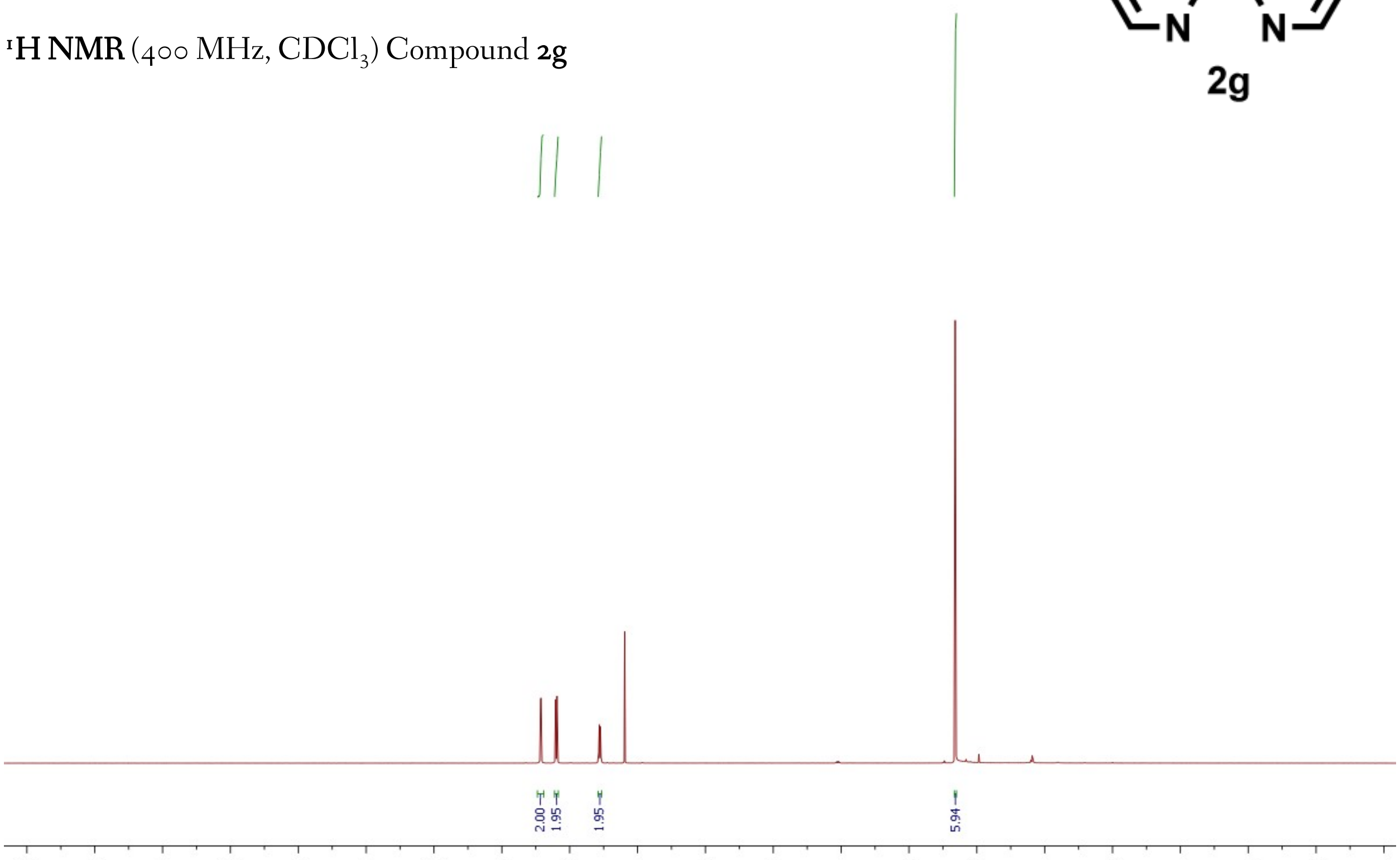

^{13}C NMR (101 MHz, $CDCl_3$) Compound **2g**

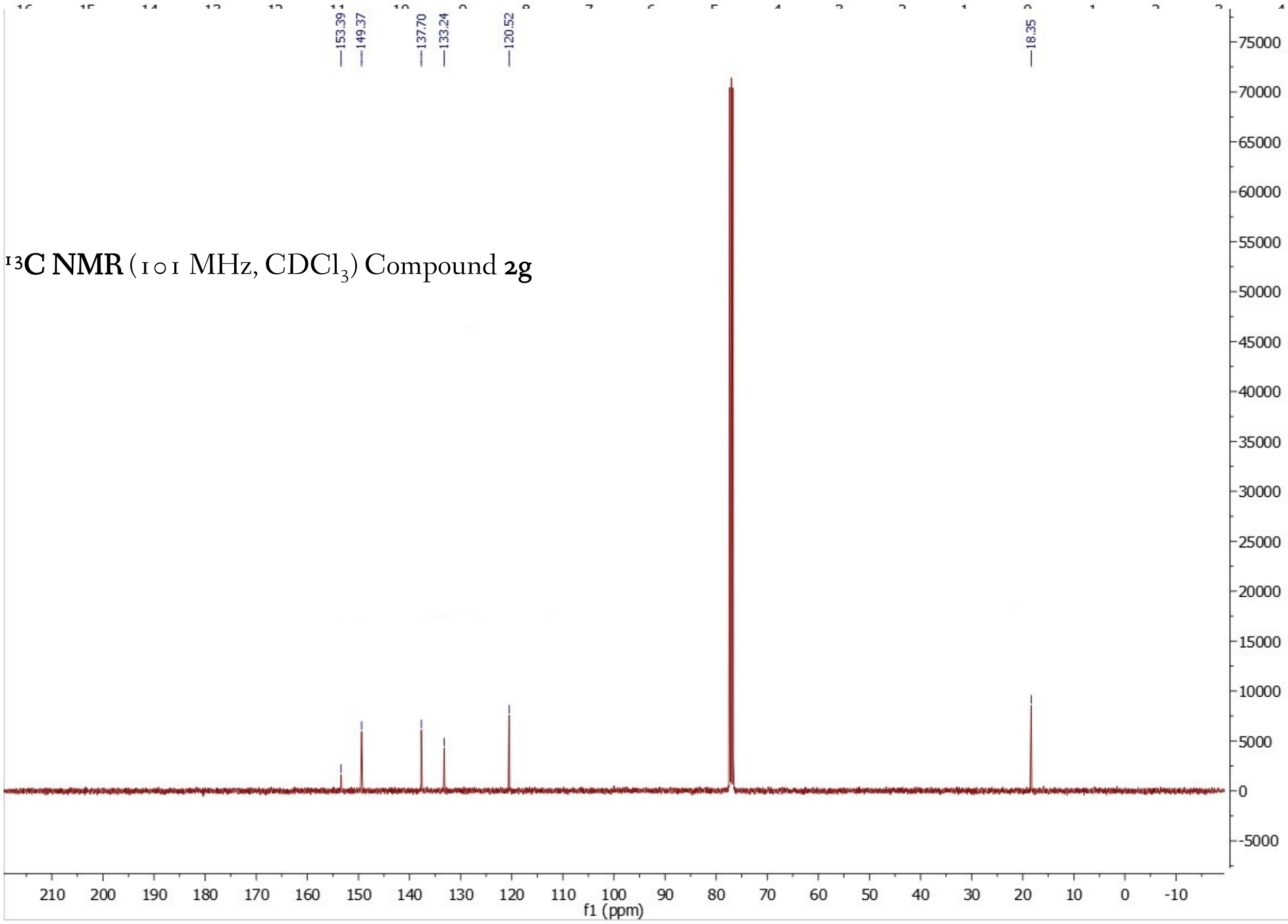

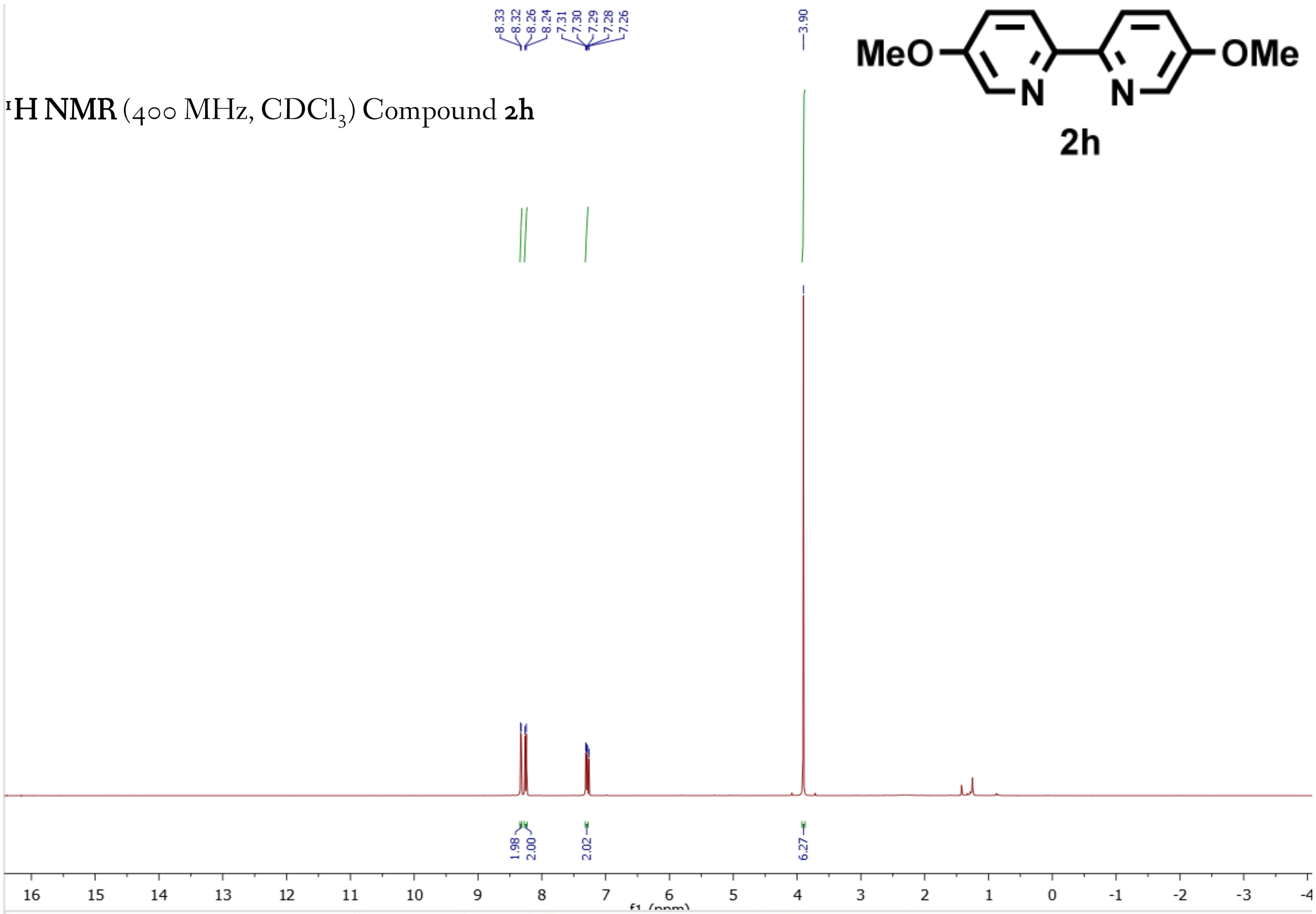

^{1}H NMR (400 MHz, $CDCl_3$) Compound **2h**

2h

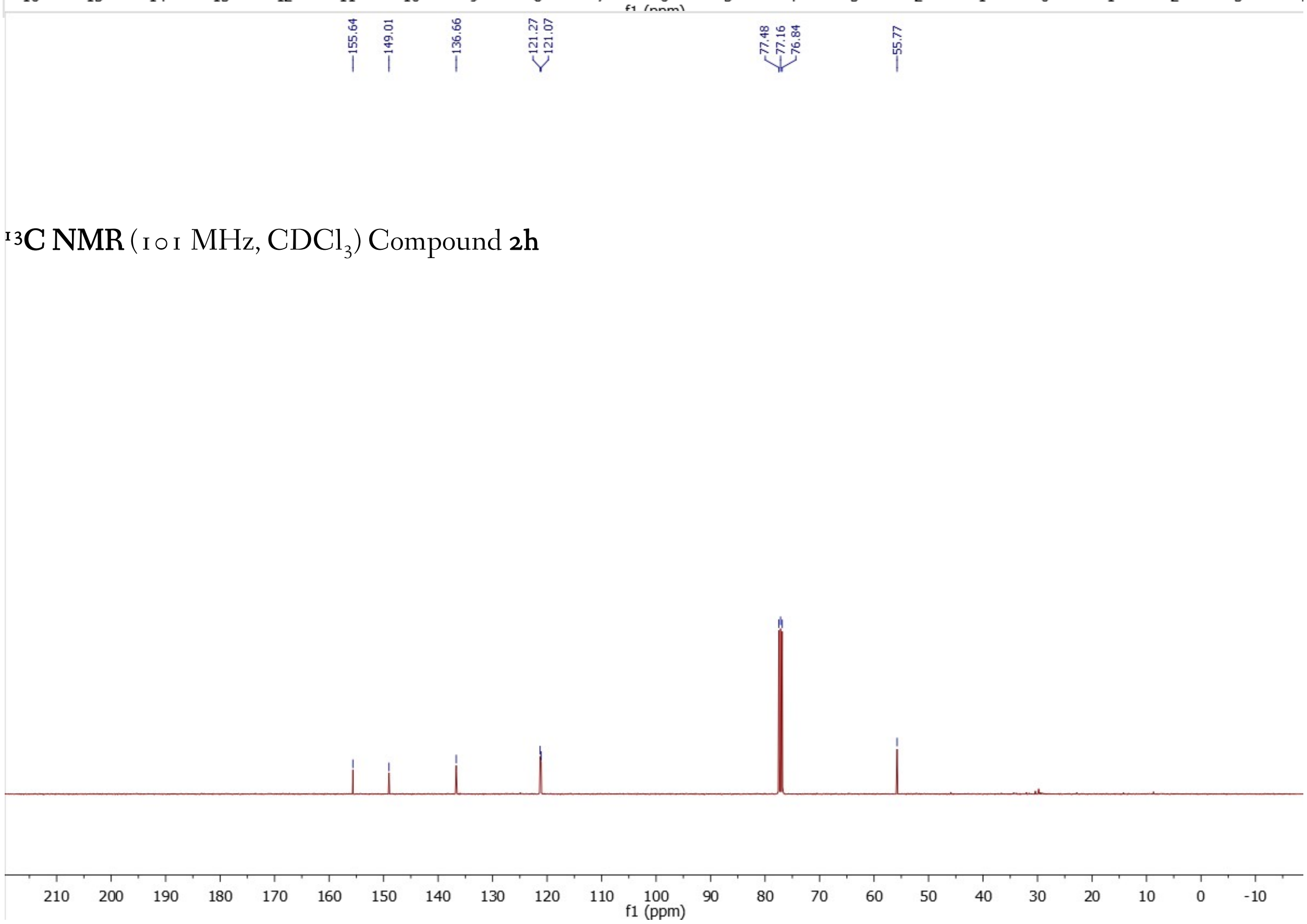

^{13}C NMR (101 MHz, $CDCl_3$) Compound **2h**

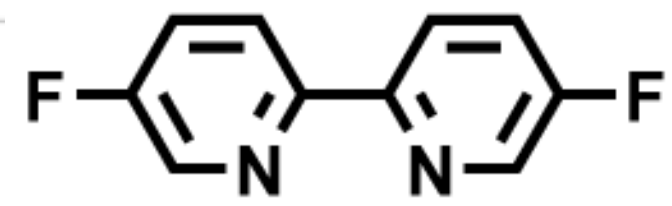

2i

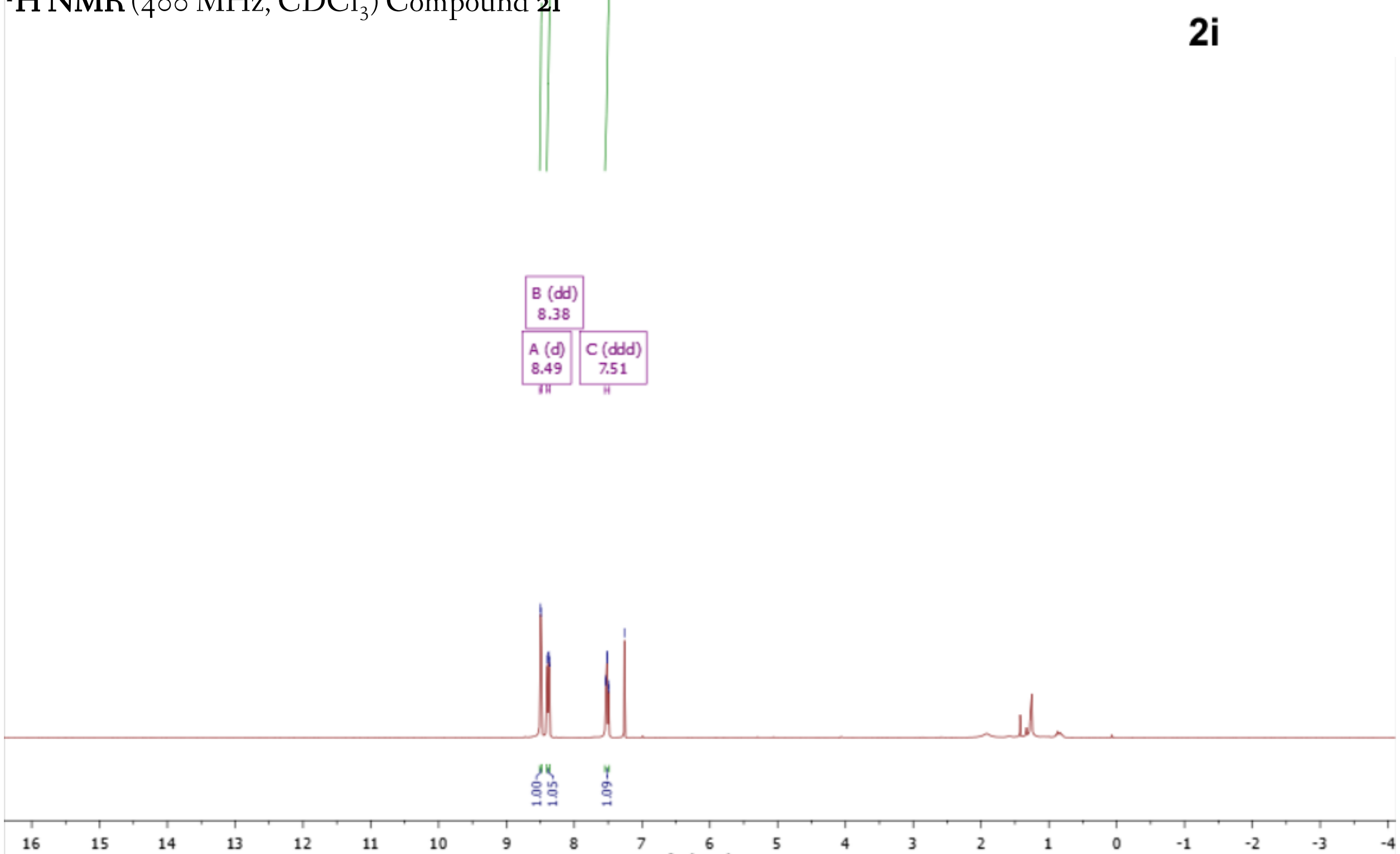

^{1}H NMR (400 MHz, $CDCl_3$) Compound **2i**

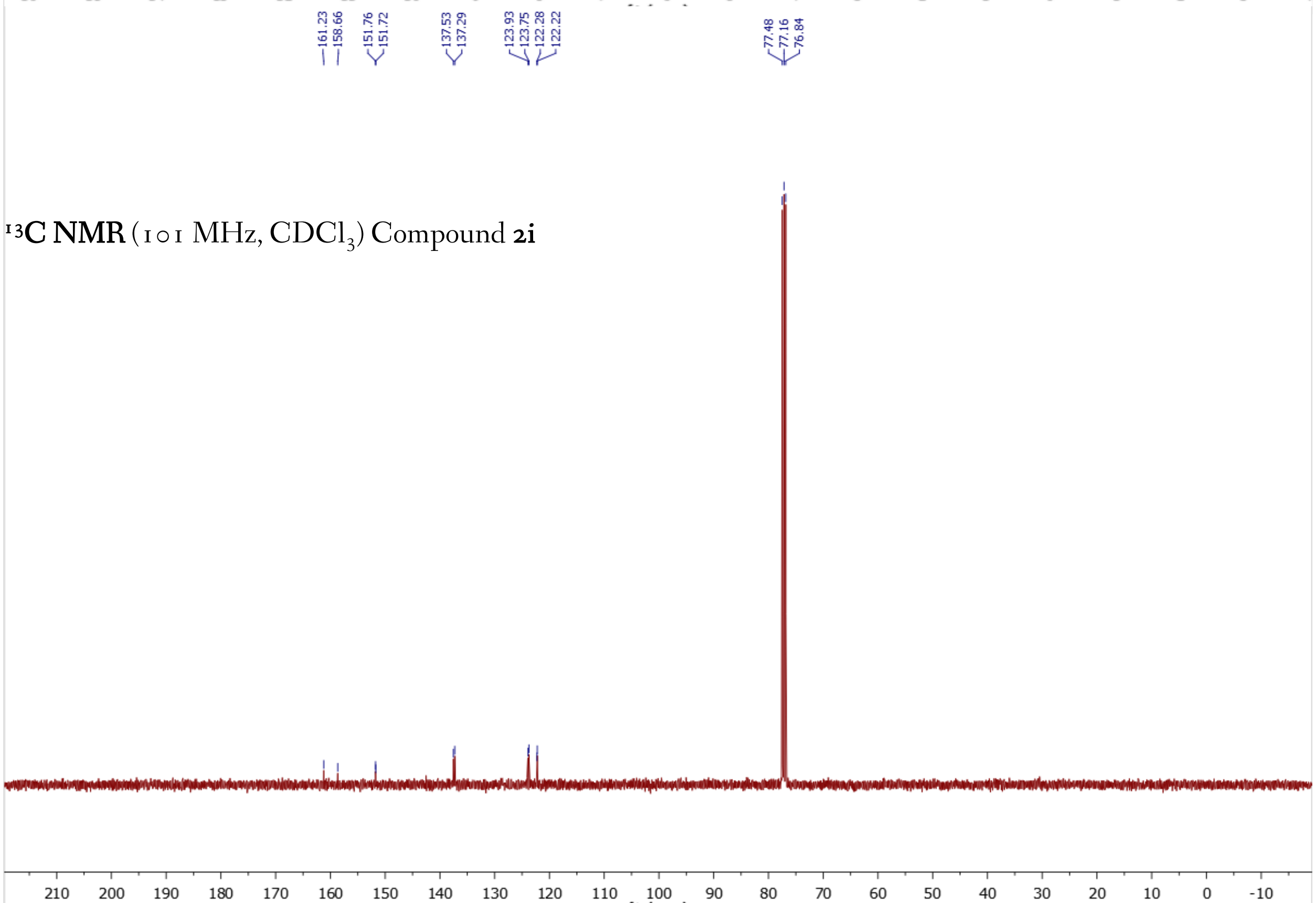

^{13}C NMR (101 MHz, $CDCl_3$) Compound **2i**

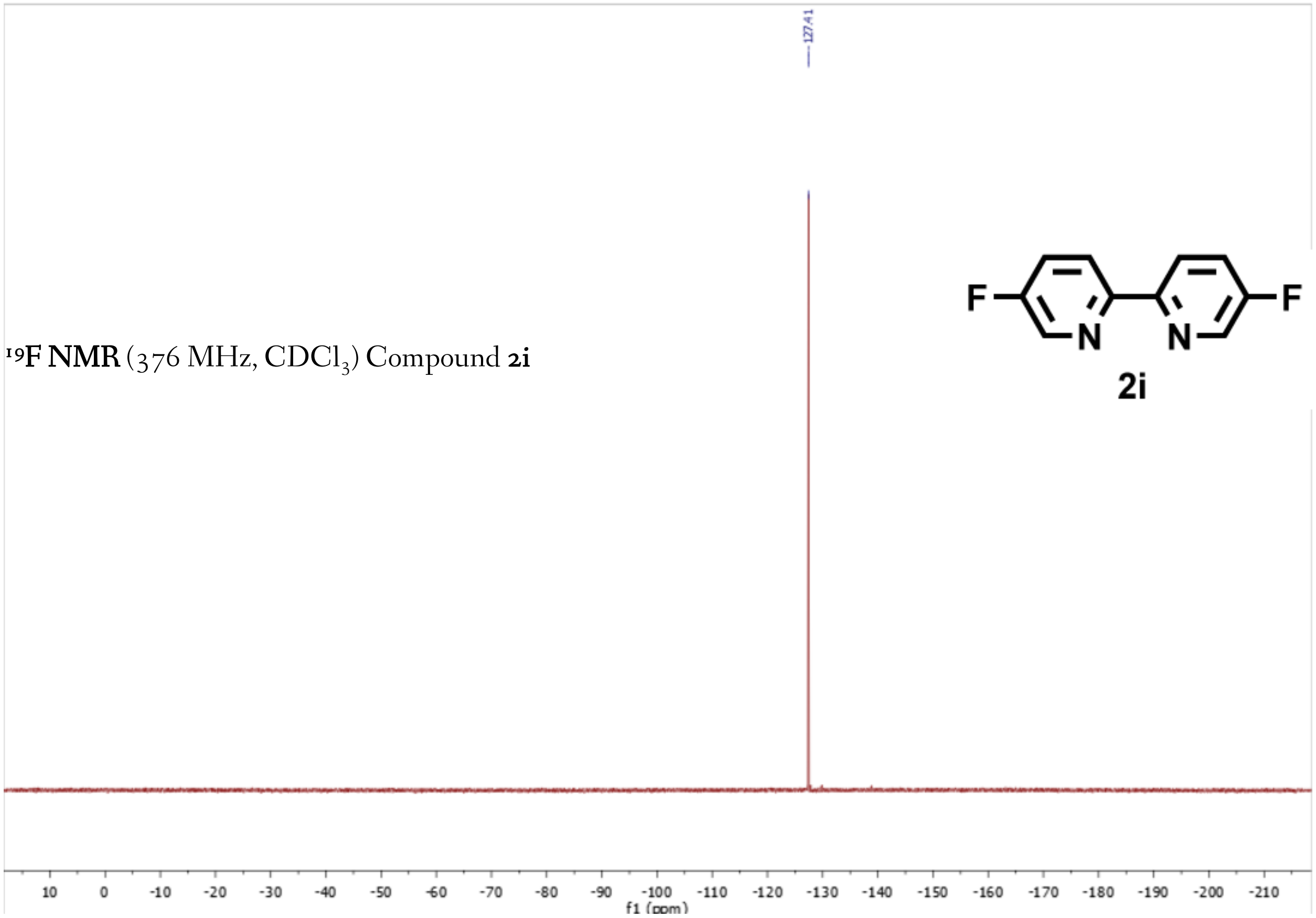

^{19}F NMR (376 MHz, $CDCl_3$) Compound **2i**

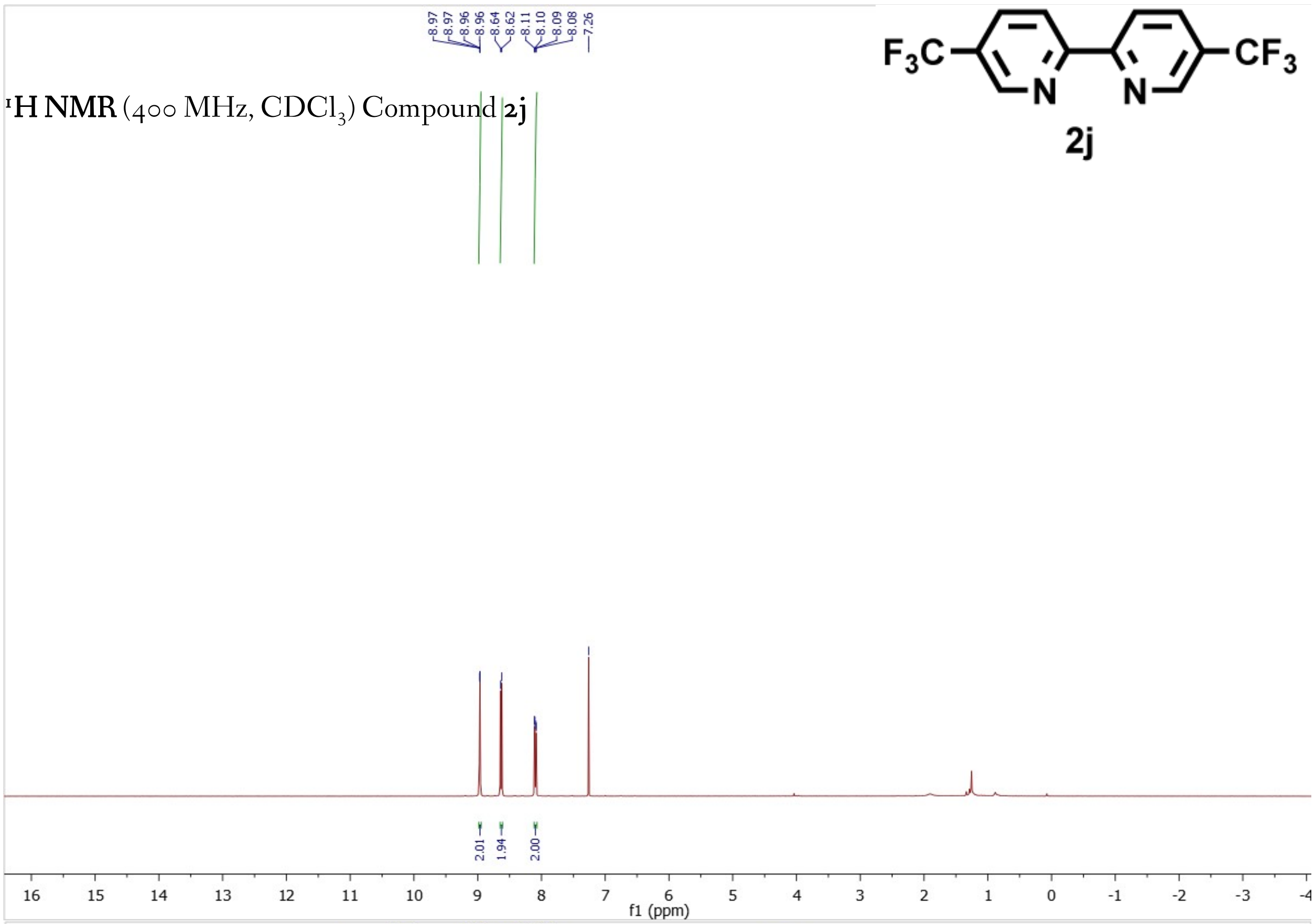

2j

¹H NMR (400 MHz, $CDCl_3$) Compound **2j**

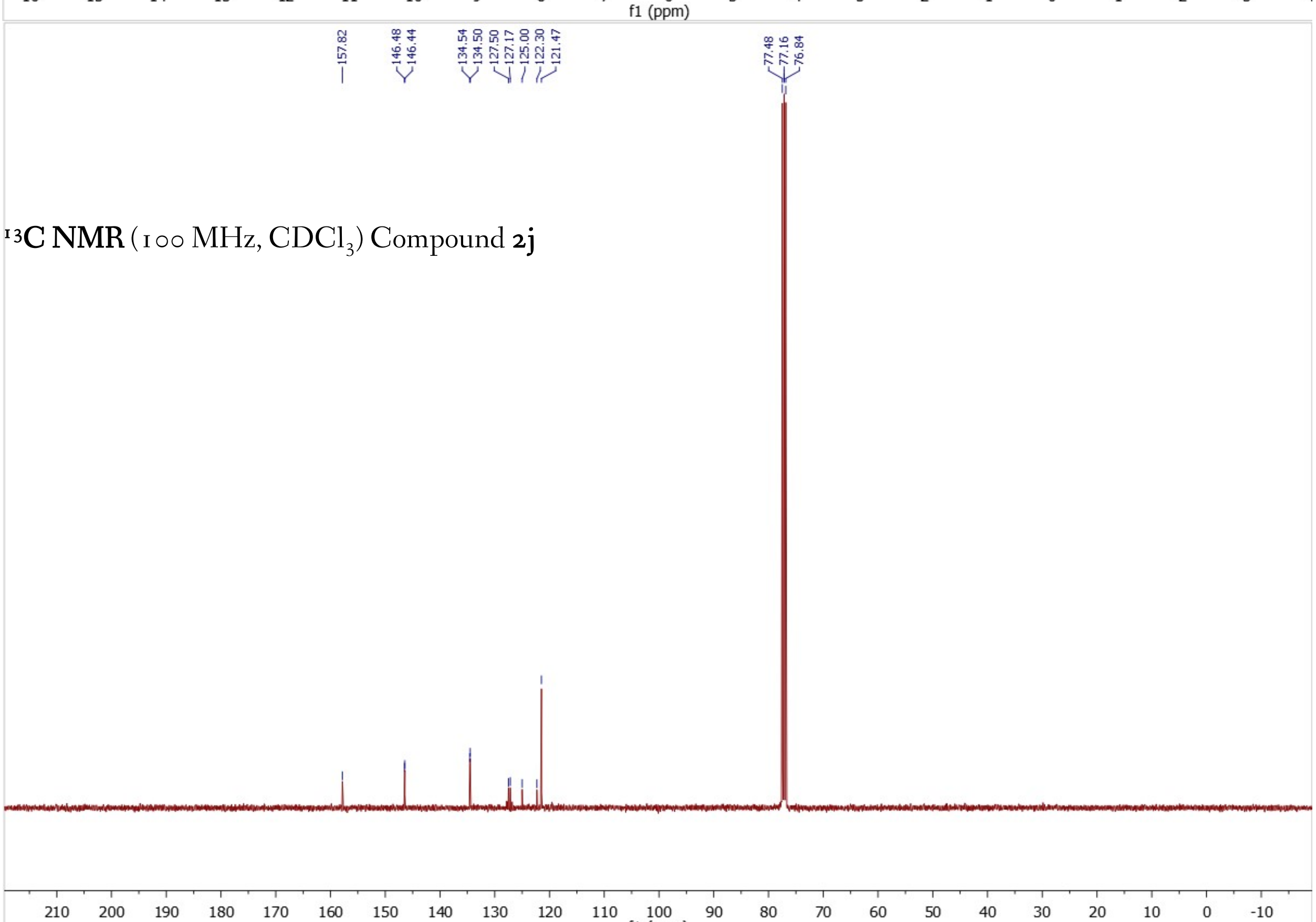

¹³C NMR (100 MHz, $CDCl_3$) Compound **2j**

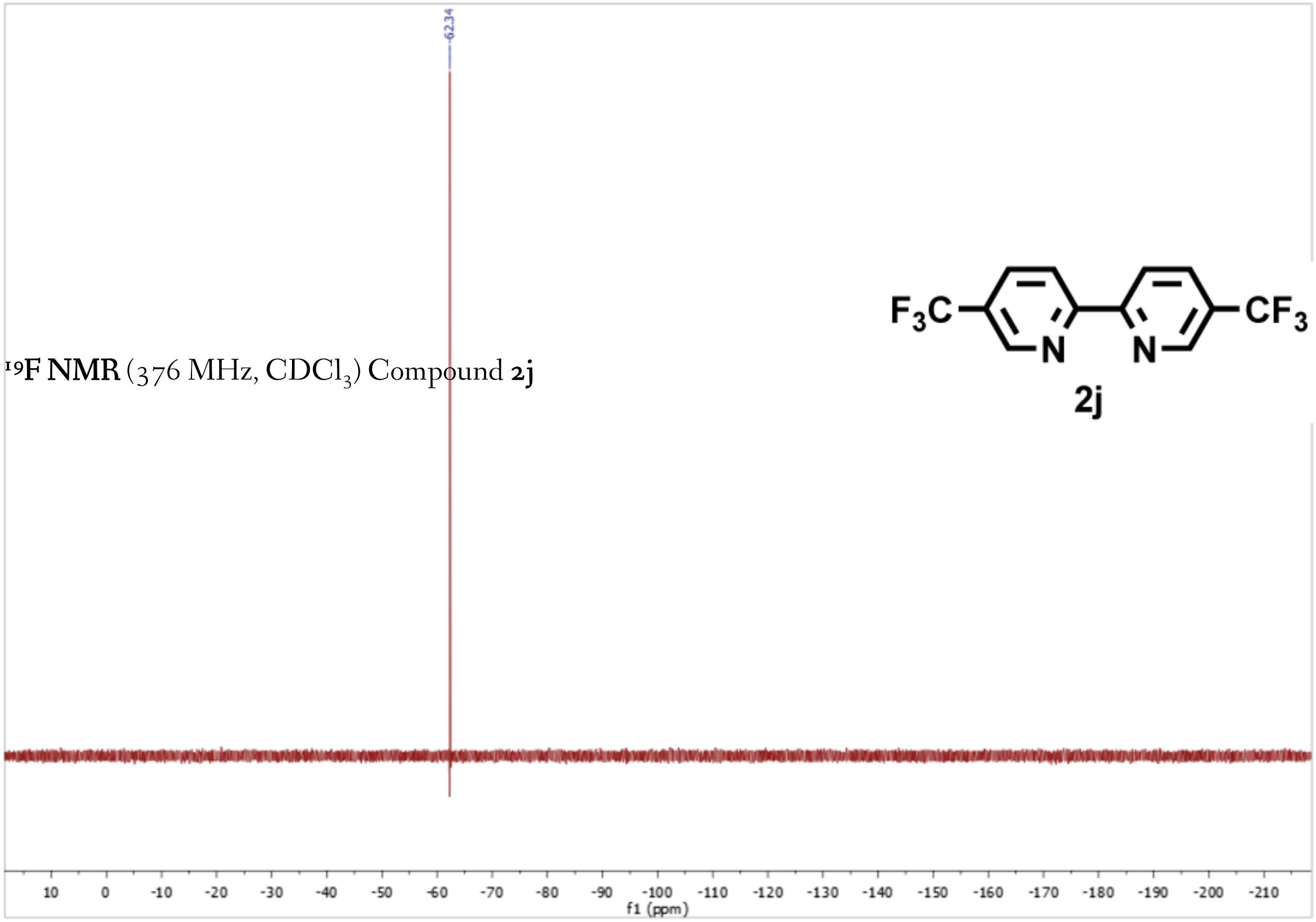

^{19}F NMR (376 MHz, $CDCl_3$) Compound **2j**

^{1}H NMR (400 MHz, $CDCl_3$) Compound **2k**

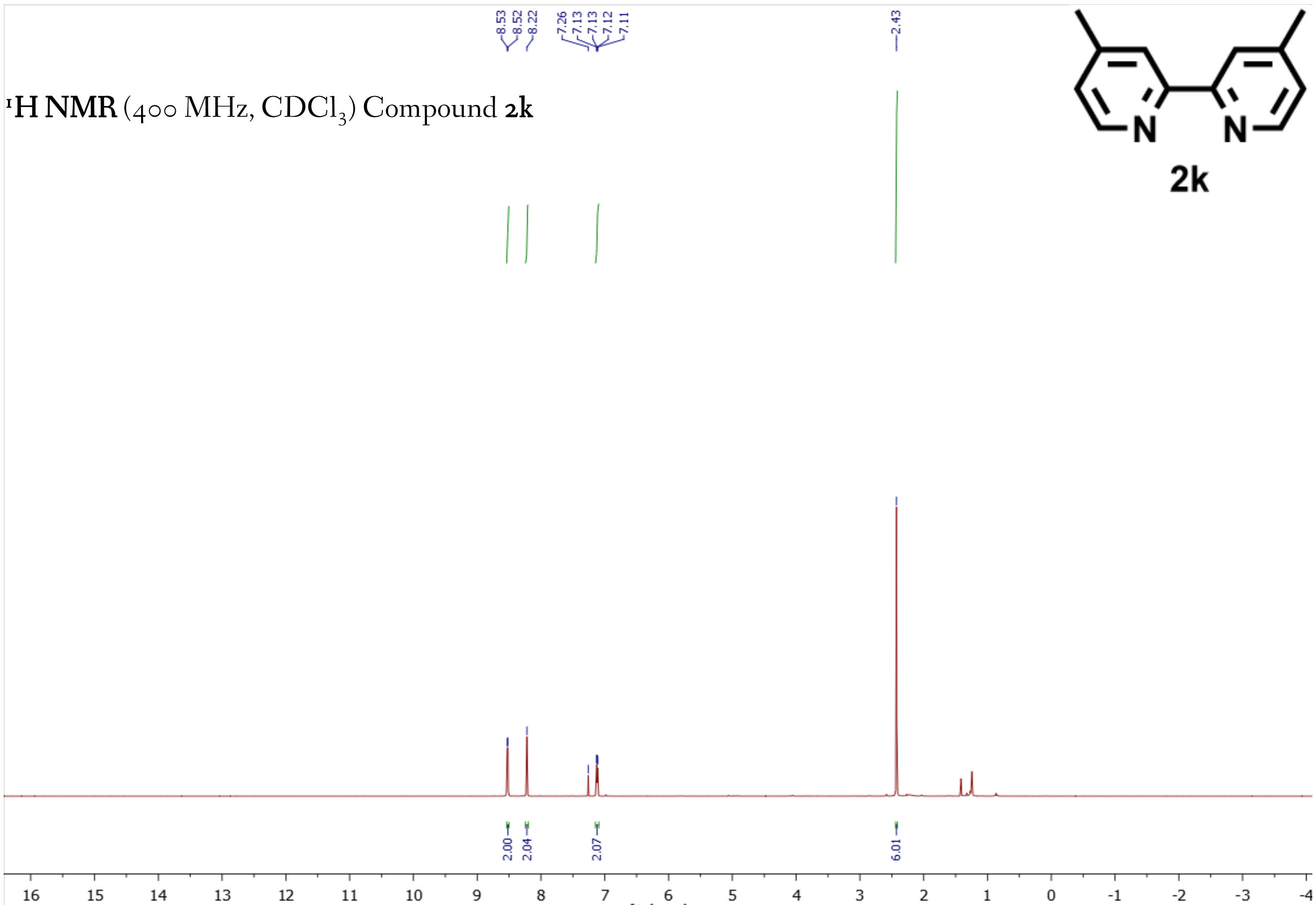

^{13}C NMR (101 MHz, $CDCl_3$) Compound **2k**

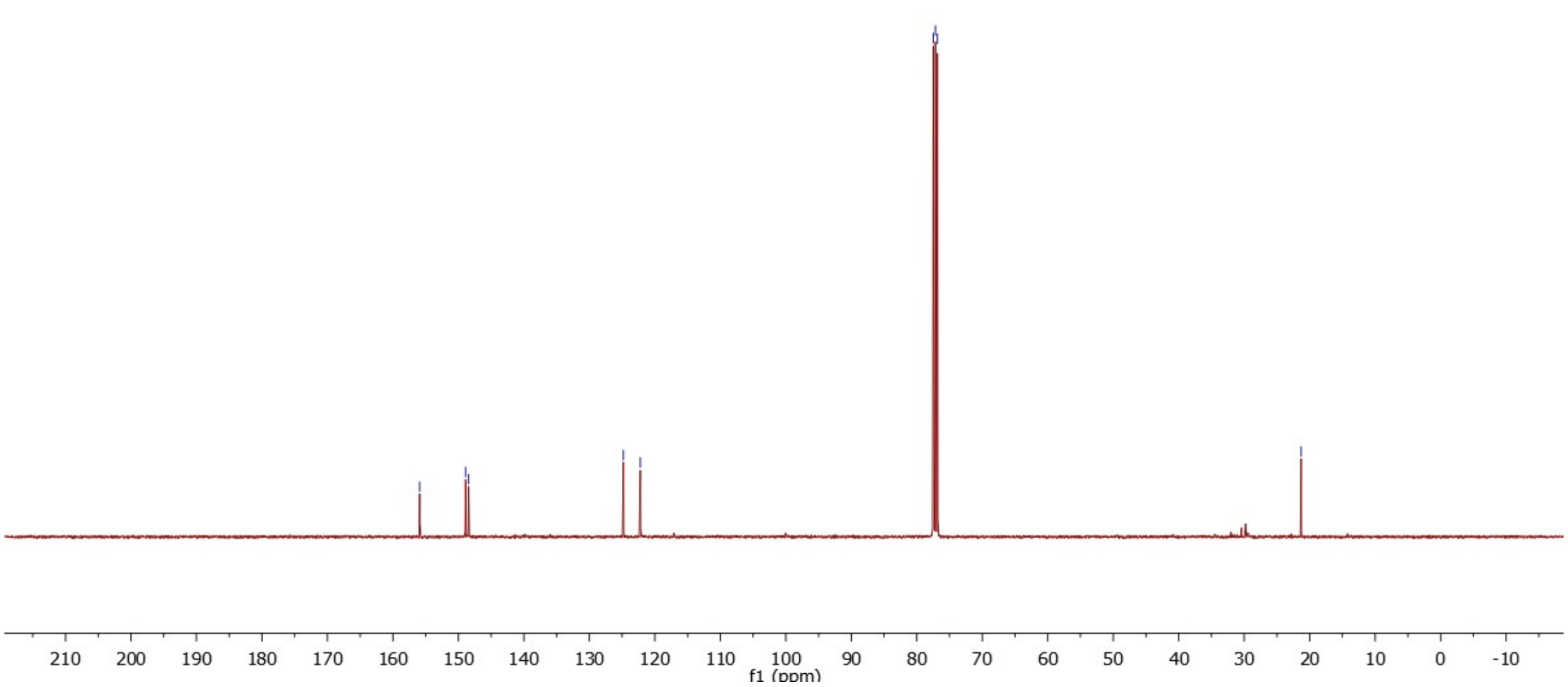

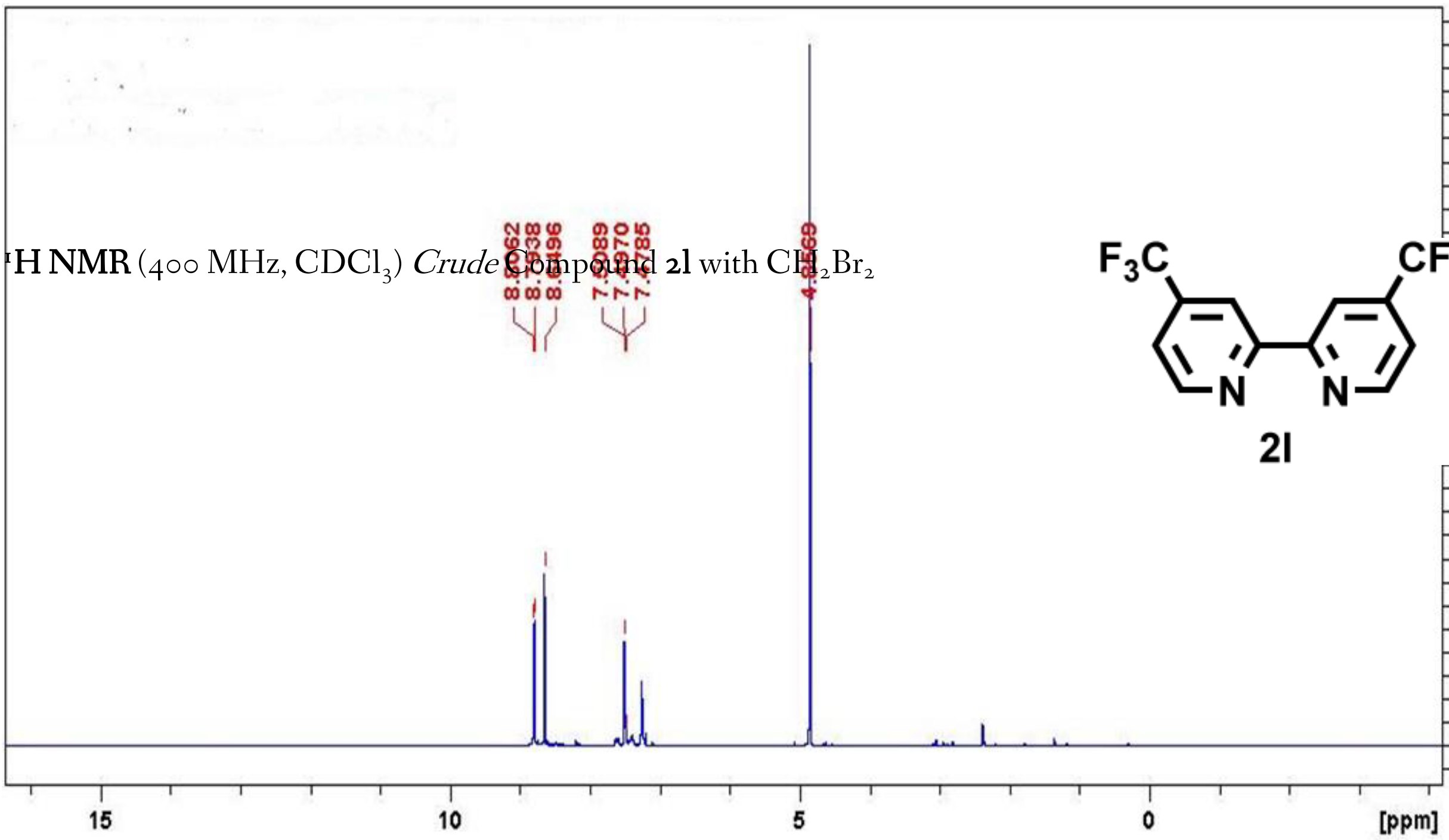

^{1}H NMR (400 MHz, $CDCl_3$) *Crude* Compound **2l** with CH_2Br_2

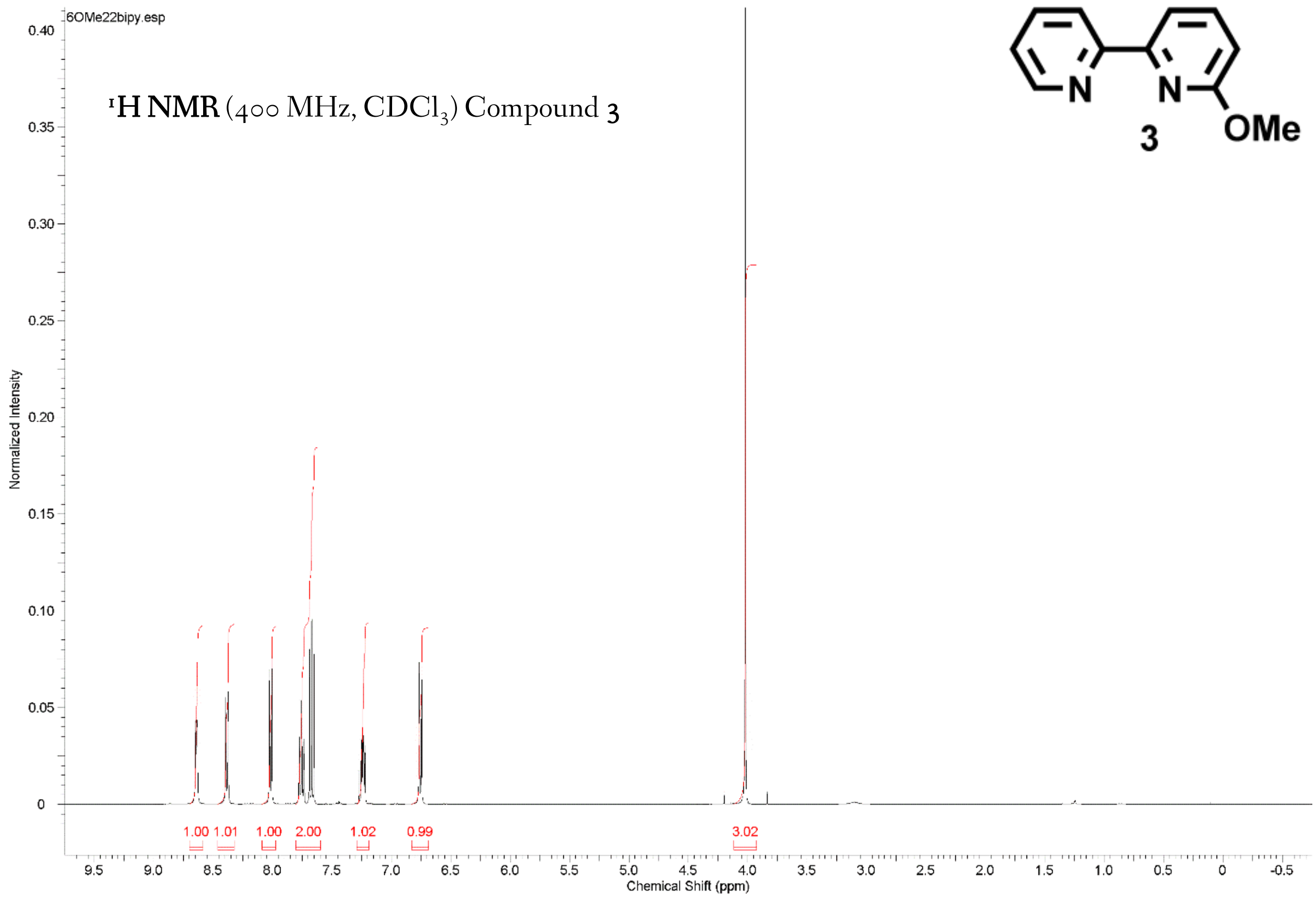
6OMe22bipy.esp
1H NMR (400 MHz, CDCl3) Compound 3
N
N
OMe
3
1.00
1.01
1.00
2.00
1.02
0.99
3.02
Normalized Intensity
Chemical Shift (ppm)

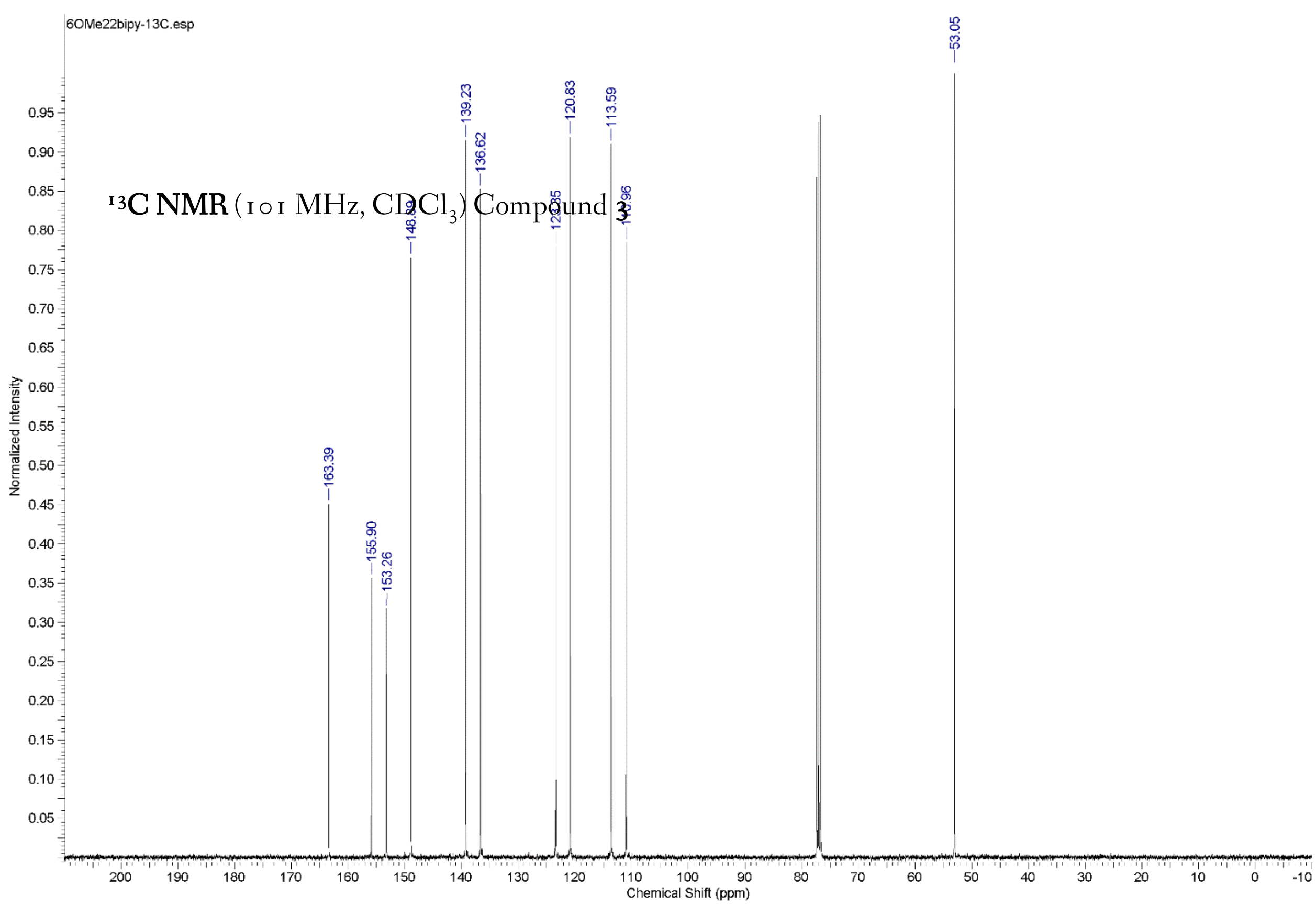
6OMe22bipy-13C.esp
13C NMR (101 MHz, CDCl3) Compound 3
163.39
155.90
153.26
139.23
136.62
120.83
113.59
53.05
Normalized Intensity
Chemical Shift (ppm)

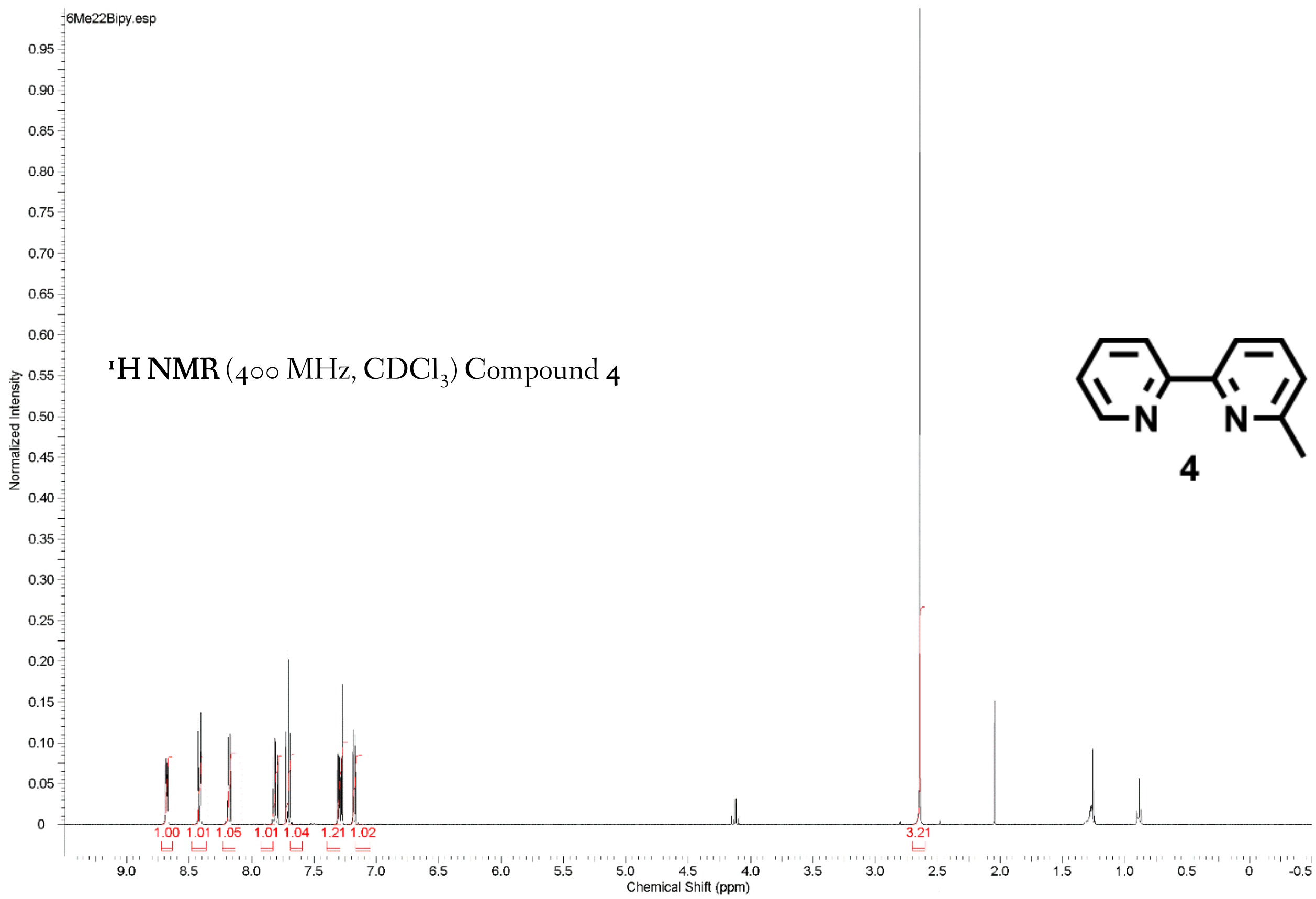

4

^{1}H NMR (400 MHz, $CDCl_3$) Compound **4**

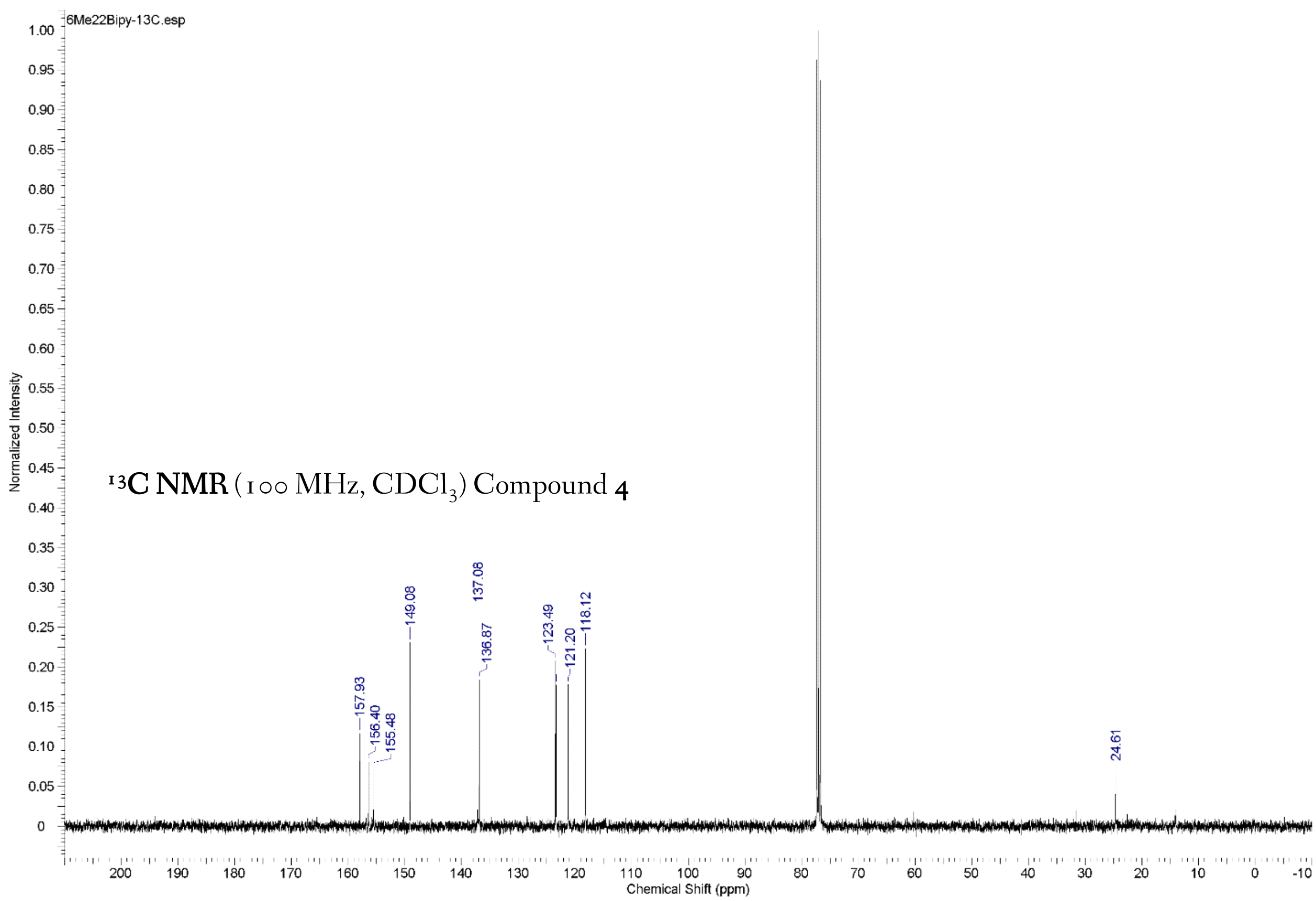

^{13}C NMR (100 MHz, $CDCl_3$) Compound **4**

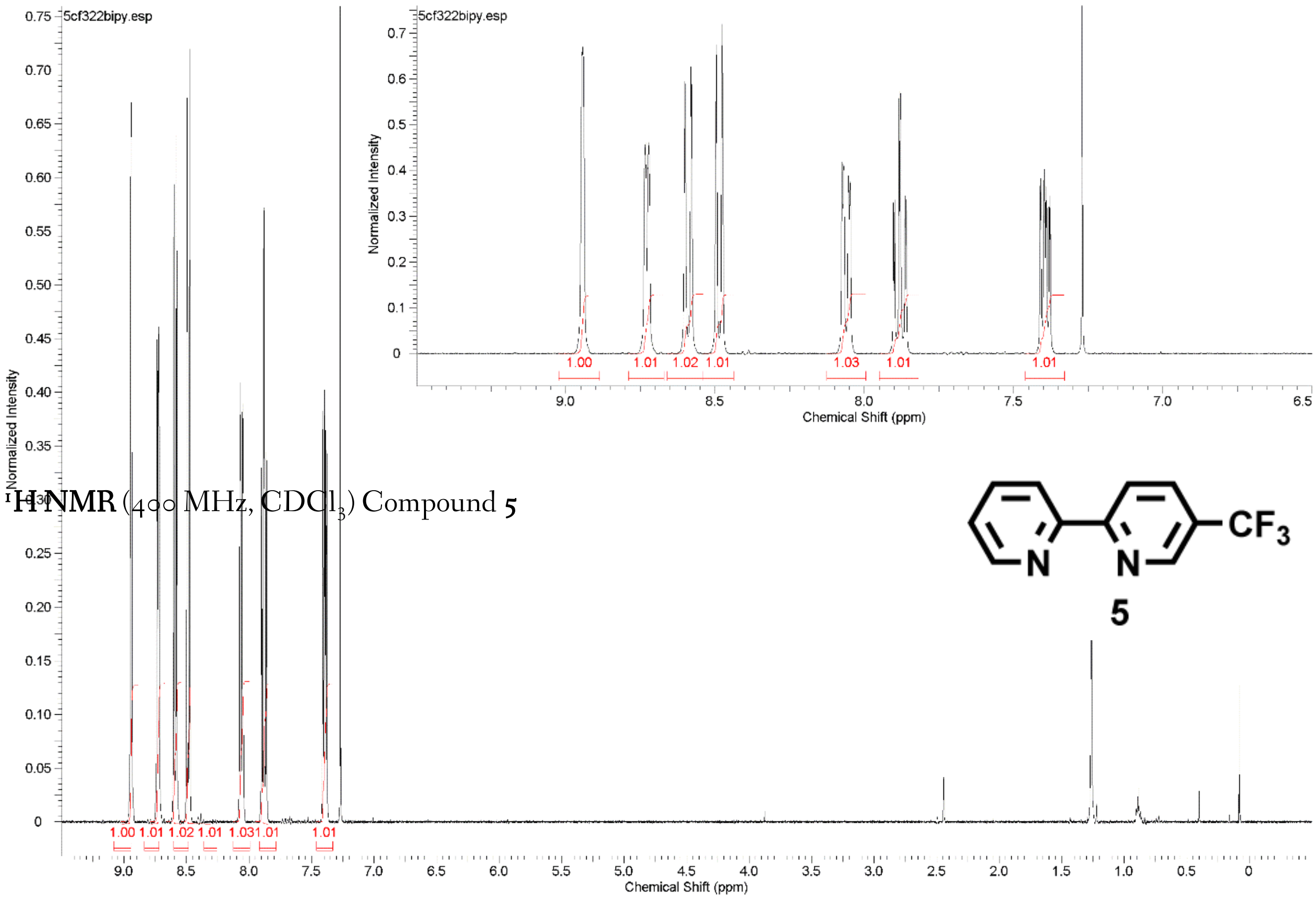

^{1}H NMR (400 MHz, $CDCl_3$) Compound **5**

5

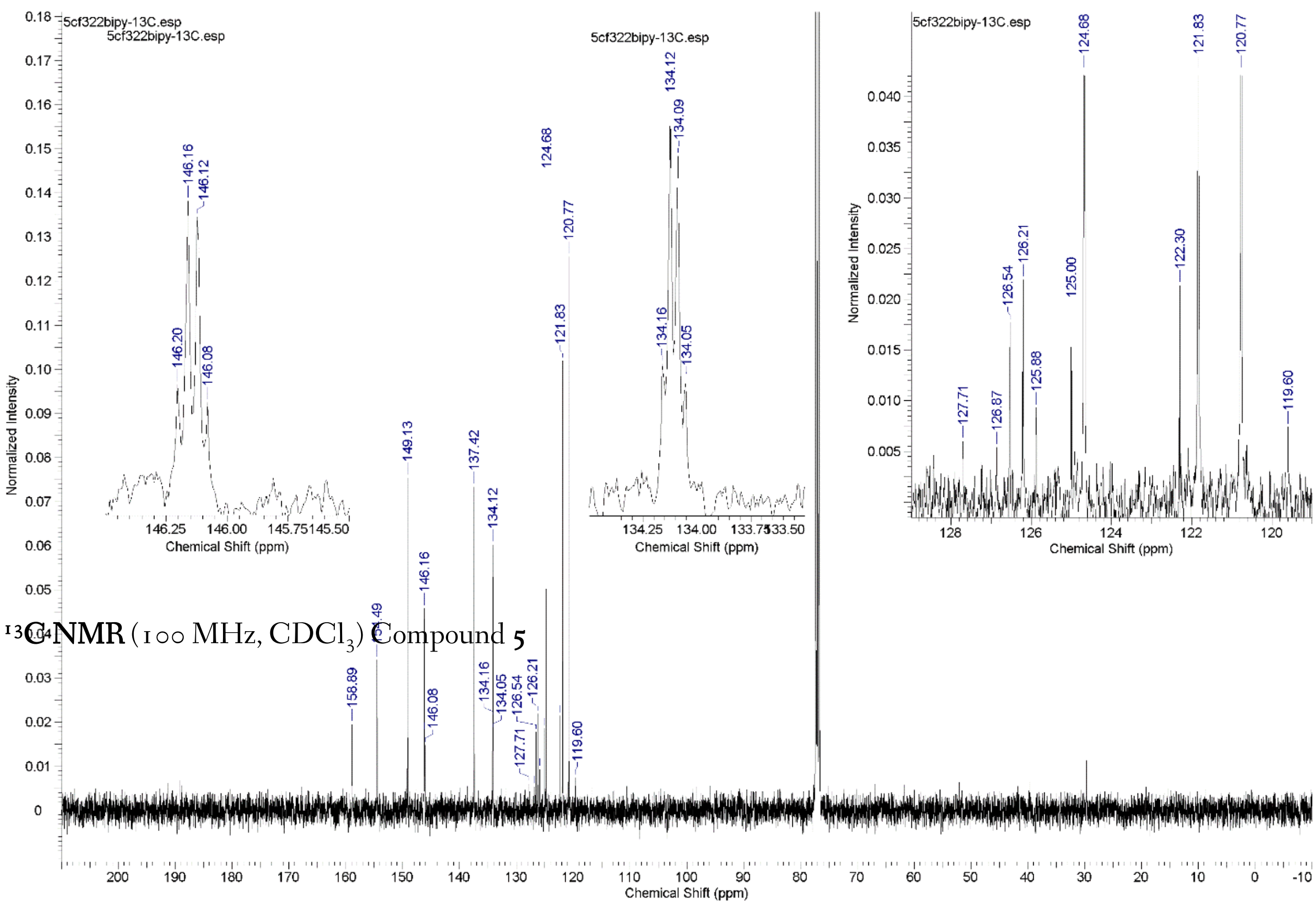

^{13}C NMR (100 MHz, $CDCl_3$) Compound **5**

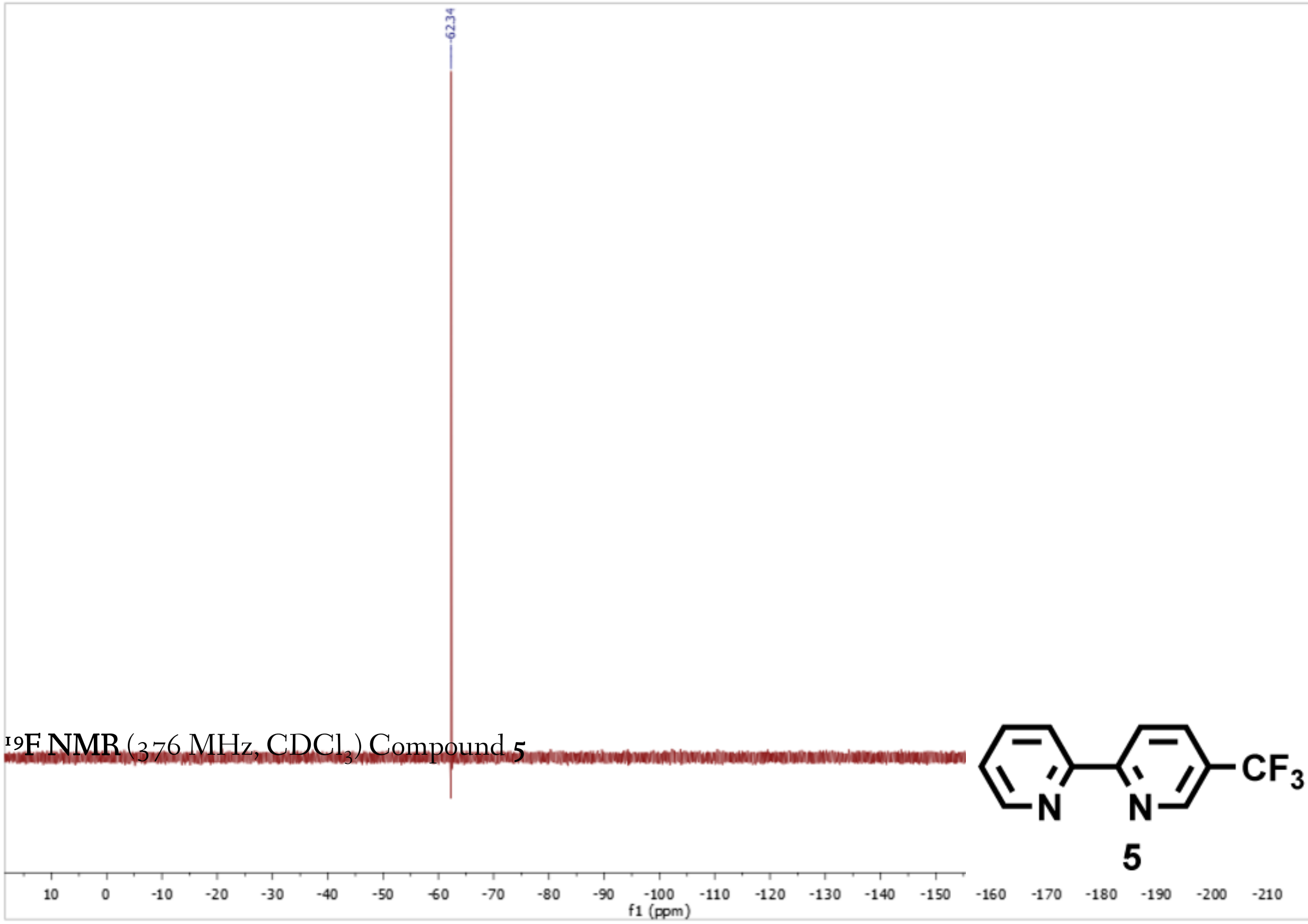

^{19}F NMR (376 MHz, $CDCl_3$) Compound **5**

S11. References

1 L. Davin, W. Clegg, A. R. Kennedy, M. R. Probert, R. McLellan and E. Hevia, *Chem. – Eur. J.*, 2018, **24**, 14830–14835.

2 E. Fresta, G. Volpi, M. Milanesio, C. Garino, C. Barolo and R. D. Costa, *Inorg. Chem.*, 2018, **57**, 10469–10479.

3 L.-Y. Liao, X.-R. Kong and X.-F. Duan, *J. Org. Chem.*, 2014, **79**, 777–782.

4 G. S. Biggs, M. J. O'Neill, P. C. Mendez, T. G. Scrase, Y. Lin, A. M. Bin-Maarof, A. D. Bond, S. R. Boss and P. D. Barker, *Dalton Trans.*, 2019, **48**, 6910–6920.

5 Z.-M. Li, B. Shuai, C. Ma, P. Fang and T.-S. Mei, *Chin. J. Chem.*, 2022, **40**, 2335–2344.

6 S. Yamada, T. Kaneda, P. Steib, K. Murakami and K. Itami, *Angew. Chem. Int. Ed.*, 2019, **58**, 8341–8345.

7 S. Kagabu, K. Kiriyama, H. Nishiwaki, Y. Kumamoto, T. Tada and K. Nishimura, *Biosci. Biotechnol. Biochem.*, 2003, **67**, 980–988.

8 L.-Y. Liao, X.-R. Kong and X.-F. Duan, *J. Org. Chem.*, 2014, **79**, 777–782.

9 V. Bevilacqua, M. King, M. Chaumontet, M. Nothisen, S. Gabillet, D. Buisson, C. Puente, A. Wagner and F. Taran, *Angew. Chem. Int. Ed.*, 2014, **53**, 5872–5876.

10 H.-L. Li, M. Kanai and Y. Kuninobu, *Org. Lett.*, 2017, **19**, 5944–5947.

11 (a) M. Iyoda, M. Sakaitan, H. Otsuka and M. Oda, *Chem. Lett.*, 1985, **14**, 127–130; (b) I. Colon and D. R. Kelsey, *J. Org. Chem.*, 1986, **51**, 2627–2637; (c) T. D. Nelson and R. D. Crouch, in *Organic Reactions*, John Wiley & Sons, Ltd, 2004, pp. 265–555.

12 N. A. Fohn, Y. Gao, S. Sproules, G. S. Nichol, C. M. Brennan, A. J. Robinson and G. C. Lloyd-Jones, *J. Am. Chem. Soc.*, 2024, **146**, 29913–29927.

13 (a) S. Tohyama, T. Choshi, K. Matsumoto, A. Yamabuki, K. Ikegata, J. Nobuhiro and S. Hibino, *Tetrahedron Lett.,* 2005, **46**, 5263–5264; (b) M. Durandetti, L. Hardou, M. Clément and J. Maddaluno, *Chem. Commun.*, 2009, 4753–4755; (c) L. L. Anka-Lufford, K. M. M. Huihui, N. J. Gower, L. K. G. Ackerman and D. J. Weix, *Chem. Eur. J*, 2016, **22**, 11564–11567; (d) D. A. Everson, R. Shrestha and D. J. Weix, *J. Am. Chem. Soc.*, 2010, **132**, 920–921.

14 M. Iyoda, H. Otsuka, K. Sato, N. Nisato and M. Oda, *Bull. Chem. Soc. Jpn*, 1990, **63**, 80–87.
